# Visible light-promoted ring-opening functionalization of unstrained cycloalkanols *via* inert C–C bond scission†
†Electronic supplementary information (ESI) available. See DOI: 10.1039/c8sc01763h
‡
‡Dedicated to Professor Takahiko Akiyama on the occasion of his 60^th^ birthday.


**DOI:** 10.1039/c8sc01763h

**Published:** 2018-06-11

**Authors:** Dongping Wang, Jincheng Mao, Chen Zhu

**Affiliations:** a Key Laboratory of Organic Synthesis of Jiangsu Province , College of Chemistry , Chemical Engineering and Materials Science , Soochow University , 199 Ren-Ai Road , Suzhou , Jiangsu 215123 , China . Email: chzhu@suda.edu.cn; b Key Laboratory of Synthetic Chemistry of Natural Substances , Shanghai Institute of Organic Chemistry , Chinese Academy of Sciences , 345 Lingling Road , Shanghai 200032 , China; c State Key Laboratory of Oil and Gas Reservoir Geology and Exploitation , Southwest Petroleum University , Chengdu 610500 , China

## Abstract

Described herein is a novel, useful, visible light-promoted ring-opening functionalization of unstrained cycloalkanols. Upon scission of an inert cyclic C–C σ-bond, a set of medium- and large-sized rings are readily brominated under mild reaction conditions to afford the corresponding distal bromo-substituted alkyl ketones that are hard to synthesize otherwise. The products are versatile building blocks, which are easily converted to other valuable molecules in one-step operation. This protocol is also applicable to the unprecedented ring-opening cyanation and alkynylation of unstrained cycloalkanols.

## Abstract 2

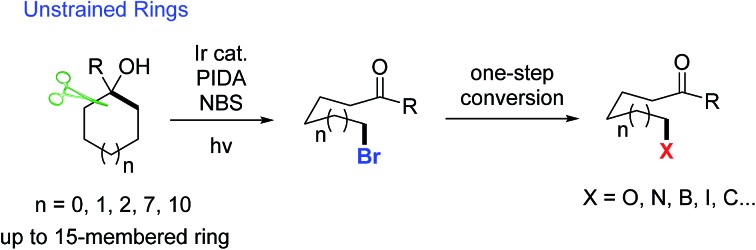
Reported herein is a novel, useful, visible light-promoted ring-opening functionalization of unstrained cycloalkanols.

## Introduction

**Scheme 1:**
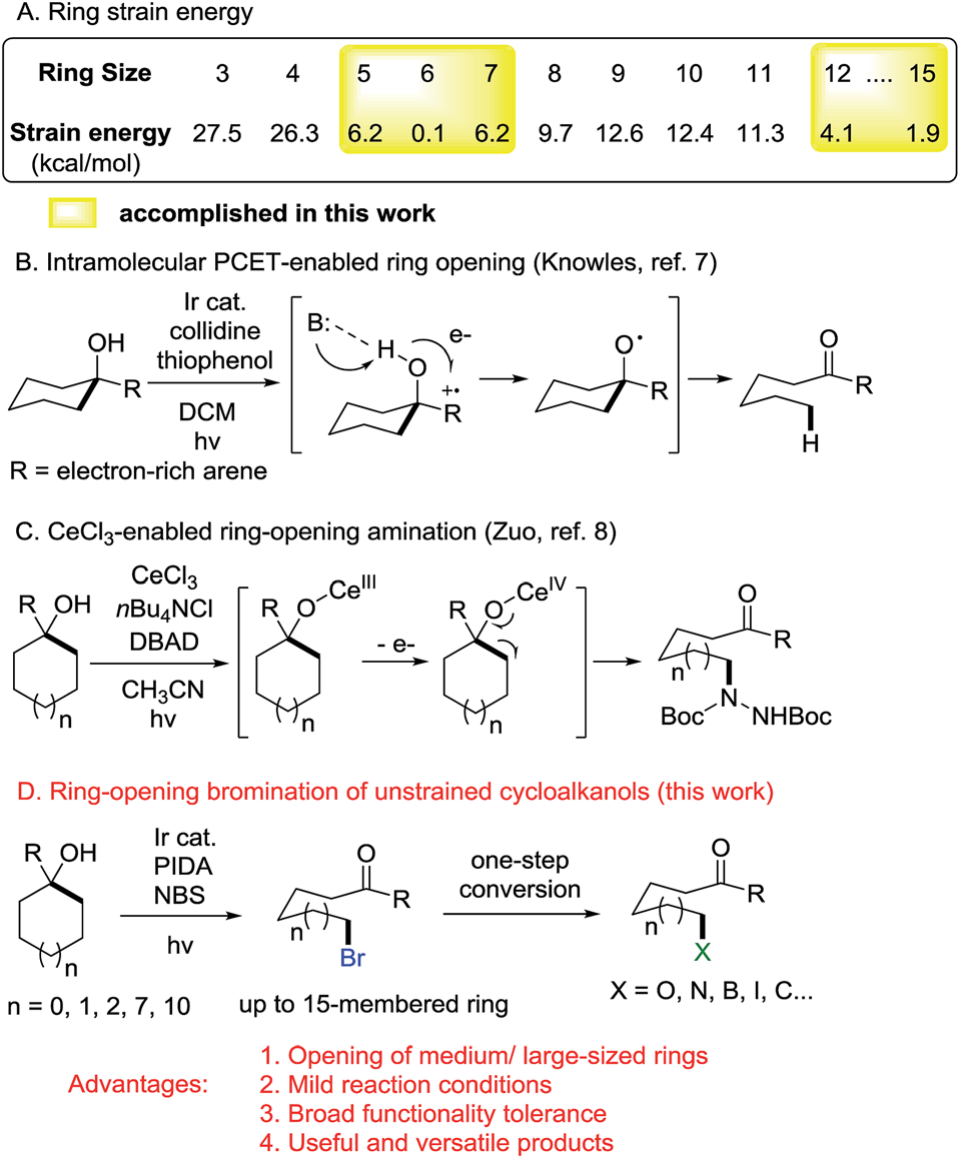
Ring-opening functionalization of unstrained cycloalkanols.

C–C bond activation is always a challenging issue in synthetic chemistry. Over the past few decades, this area has received great attention relying on the cleavage of cyclic C–C bonds.^1^ Strained cycloalkanols such as cyclopropanols and cyclobutanols have emerged as privileged precursors for the preparation of β- and γ-substituted ketones through radical-mediated ring-opening functionalization.^2^,^3^ Recently, we have consecutively reported the ring-opening functionalization of cyclobutanols to construct various chemical bonds, *e.g.*, C–F, C–Cl, C–N, C–S, C–C, *etc.*, based on silver or manganese catalysis.^4^ However, the opening of unstrained rings (in particular, 5–7 and 12–15 membered rings) is always confronted with a formidable challenge. It is mainly attributed to the significantly decreased ring-strain energy (Scheme 1A).^5^ During our research, it was also found that the intramolecular dehydration took place in competition with a ring-opening pathway to suppress the reaction outcome. Therefore, an efficient catalytic protocol should be sought for the ring opening of unstrained cycloalkanols.

Photoredox catalysis has proven to be a powerful tool for the mild generation of an alkyloxy radical that triggers the subsequent ring-opening reactions or other transformations.^6^ Recently, Knowles *et al.* applied intramolecular proton-coupled electron transfer (PCET) to the photocatalytic ring opening of unstrained cycloalkanols.^7^ In this remarkable process, however, electron-rich tertiary alcohols were required to generate the arene radical cation, a key intermediate for the intramolecular PCET process. This somewhat limited the practicality of the protocol (Scheme 1B). Later, Zuo *et al.* disclosed an elegant photocatalytic ring-opening amination of unstrained cycloalkanols using a cerium chloride complex (Scheme 1C).^8^ Beyond this, other types of ring-opening functionalization of unstrained cycloalkanols are still anticipated.

Alkyl bromides are versatile building blocks in synthetic chemistry, which can be easily converted to other valuable molecules through nucleophilic substitution or cross coupling reactions. We considered that the ring-opening bromination of unstrained cycloalkanols would give rise to distally bromo-substituted alkyl ketones which are synthetically valuable but hard to synthesize otherwise. Herein, we provide support for this hypothesis (Scheme 1D). A set of medium- and large-sized rings are readily brominated through the mild cleavage of an inert cyclic C–C σ-bond with the assistance of visible-light irradiation. The newly formed C–Br bonds can function as privileged precursors for many other useful chemical bonds. Moreover, this protocol is also applicable to the unprecedented ring-opening cyanation and alkynylation of unstrained cycloalkanols.

## Results and discussion

At the outset, we implemented the reaction parameter survey with the less strained 1-phenylcyclopentanol **1a** as a model substrate and NBS as a bromine source (Table 1). It was found that the use of biphasic solvents was crucial to the reaction. In the presence of a photoredox catalyst (PC) and a hypervalent iodine reagent, the ring-opening bromination readily proceeded under visible-light irradiation, affording the desired δ-bromo alkyl ketone **2a**. A brief evaluation of solvents indicated that CCl_4_/H_2_O delivered the best yield (entry 4), while the use of PhCF_3_/H_2_O also resulted in a comparable yield (entry 9). The yield was slightly improved to 76% by using PC 2 instead of PC 1 (entry 13). Other than PIDA, hypervalent iodine reagents such as BI-OH, IBX, and DMP were also suitable for the reaction, but led to lower yields (entries 16–18). The amount of H_2_O was important to the reaction outcome. Increasing the volume of H_2_O in biphasic solvents decreased the yield (entry 19), whereas using anhydrous CCl_4_ only led to trace amounts of product **2a** (entry 20). Both PC and PIDA were not indispensable; removing either one still afforded the product in modest yields (entries 21 and 22). It might be suggested that the overall ring-opening process was the outcome of two different pathways. The reaction did not proceed in the absence of both PC and PIDA or in the dark (entries 23 and 24). Finally, the yield of **2a** was further improved to 78% with the use of PhCF_3_/H_2_O as the mixed solvent (entry 25).

**Table 1:** Reaction parameter survey^*a*^

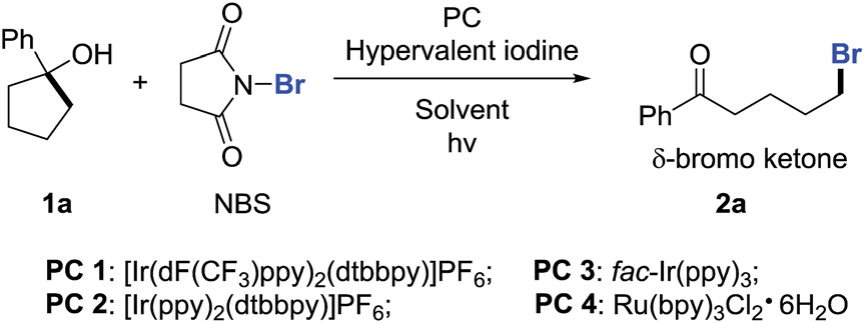				
Entry	Solvent (v/v 20/1)	Photoredox catalyst	Hypervalent iodine	Yield (%)^*b*^
1	DCM/H_2_O	PC 1	PIDA	22
2	DCE/H_2_O	PC 1	PIDA	21
3	CHCl_3_/H_2_O	PC 1	PIDA	15
4	CCl_4_/H_2_O	PC 1	PIDA	74
5	DMF/H_2_O	PC 1	PIDA	0
6	DMSO/H_2_O	PC 1	PIDA	0
7	Acetone/H_2_O	PC 1	PIDA	<5
8	Toluene/H_2_O	PC 1	PIDA	<5
9	PhCF_3_/H_2_O	PC 1	PIDA	68
10	CH_3_CN/H_2_O	PC 1	PIDA	35
11	THF/H_2_O	PC 1	PIDA	0
12	MeOH/H_2_O	PC 1	PIDA	0
13	CCl_4_/H_2_O	PC 2	PIDA	76
14	CCl_4_/H_2_O	PC 3	PIDA	59
15	CCl_4_/H_2_O	PC 4	PIDA	24
16	CCl_4_/H_2_O	PC 2	BI-OH	70
17	CCl_4_/H_2_O	PC 2	IBX	55
18	CCl_4_/H_2_O	PC 2	DMP	65
19^*c*^	CCl_4_/H_2_O	PC 2	PIDA	70
20	CCl_4_	PC 2	PIDA	<5
21	CCl_4_/H_2_O	PC 2	—	53
22	CCl_4_/H_2_O	—	PIDA	45
23	CCl_4_/H_2_O	—	—	0
24^*d*^	CCl_4_/H_2_O	PC 2	PIDA	0
25	PhCF_3_/H_2_O	PC 2	PIDA	78

^*a*^ **1a** (0.2 mmol), NBS (0.3 mmol, 1.5 equiv.), hypervalent iodine (0.4 mmol, 2.0 equiv.), and PC (0.006 mmol, 3 mol%) in mixed solvents (2.0 mL/0.1 mL) at rt, 14 W blue LED irradiation.

^*b*^Yields of isolated products.

^*c*^CCl_4_/H_2_O (2.0 mL/0.2 mL).

^*d*^In the dark.

**Scheme 2:**
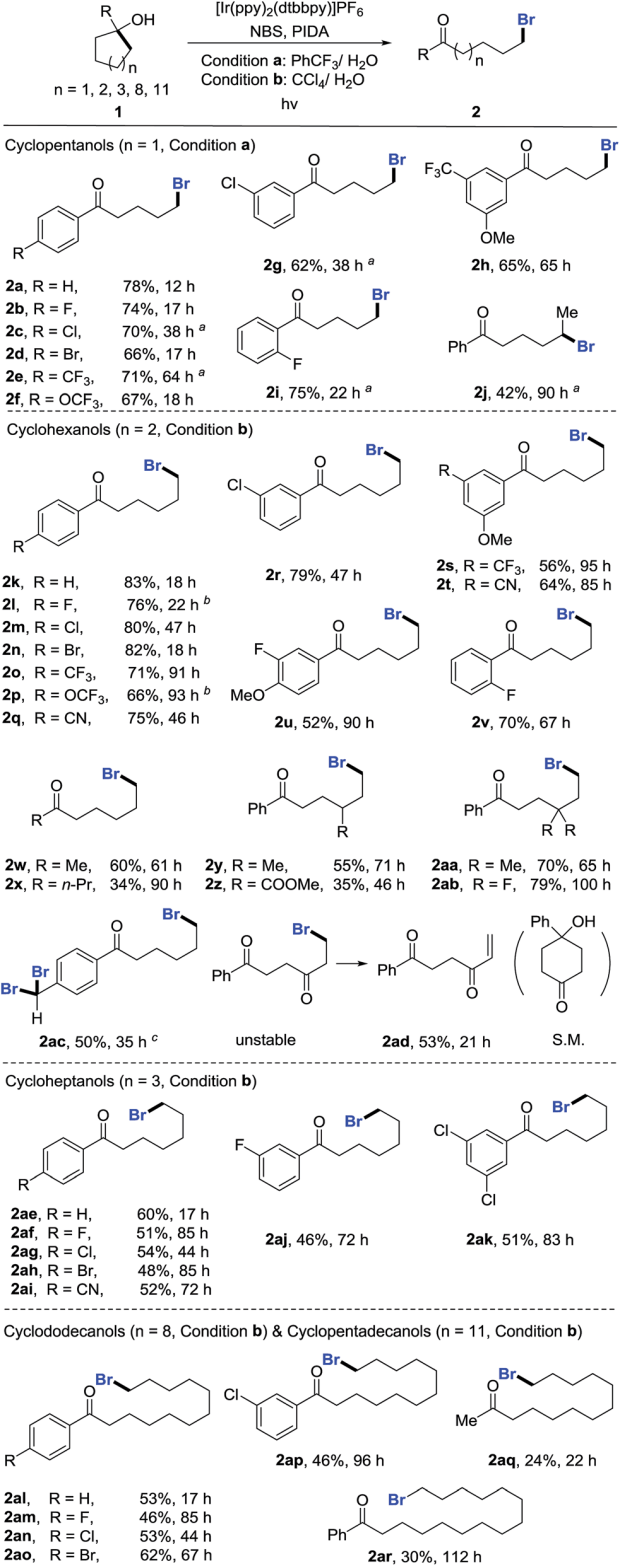
Scope of unstrained cycloalkanols. Reaction conditions: **1** (0.2 mmol), NBS (0.3 mmol, 1.5 equiv.), PIDA (0.4 mmol, 2.0 equiv.), and PC (0.006 mmol, 3 mol%) in mixed solvents (2.0 mL/0.1 mL) at rt, 14 W blue LED irradiation. Yields of isolated products are given. ^*a*^With condition b. ^*b*^[Ir(dF(CF_3_)ppy)_2_(dtbbpy)]PF_6_ as PC. ^*c*^NBS (0.8 mmol, 4.0 equiv.).

With the optimized reaction conditions in hand, we set out to investigate the generality of the protocol (Scheme 2). First, a variety of cyclopentanols were tested. Generally, electron-deficient aryl substituents were preferred for the reaction, leading to δ-bromo alkyl ketones in synthetically useful yields. Halides (**2b–2d**), in particular bromides (**2d**), were well tolerated, providing a platform for the late-stage product manipulation. Positional change (*para*-, *meta*-, and *ortho*-) of substituents did not impede the transformation (**2g–2i**). Although highly electron-rich aryl compounds such as anisole were not suitable due to the electrophilic bromination of the aryl group, it could be overcome by mounting an additional electron-withdrawing group (**2h**). This method also allowed for the preparation of secondary bromides (**2j**). The ring opening of unsymmetric cycloalkanols proceeded in a regioselective manner. However, the synthesis of tertiary bromides failed under the current reaction conditions. The scope of cyclohexanols was investigated more extensively than that of cyclopentanols. Besides the functional groups already examined, other groups such as cyano (**2q** and **2t**) and ester (**2z**) were also compatible to the reaction conditions. In addition to aryl-substituted cyclohexanols, alkyl-substituted cyclohexanols were apt to furnish the elusive distal-bromo dialkyl ketones (**2w** and **2x**). Various substituents could be incorporated into the alkyl chain by using the functionalized cycloalkanol precursors (**2y–2ab**). In the presence of excess NBS, three-fold bromination took place on the toluene-substituted cycloalkanol to give product **2ac** which was brominated at both benzylic and distal positions. The reaction with the cyclohexanol derived from 1,4-cyclohexandione afforded the unsaturated 1,4-diketone *via* dehydrobromination (**2ad**). Likewise, the ring-opening bromination of cycloheptanols bearing various functional groups also readily proceeded, affording the corresponding distal-bromo alkyl ketones in useful yields (**2ae–2ak**). Unfortunately, the ring opening of eight- and ten-membered cycloalkanols failed, leading to complex mixtures. The reason is unclear so far. Remarkably, the opening of large-sized rings such as cyclododecanols and cyclopentadecanols occurred efficiently, yielding a variety of distally brominated alkyl ketones that are hard to synthesize otherwise (**2al–2ar**).

**Scheme 3:**
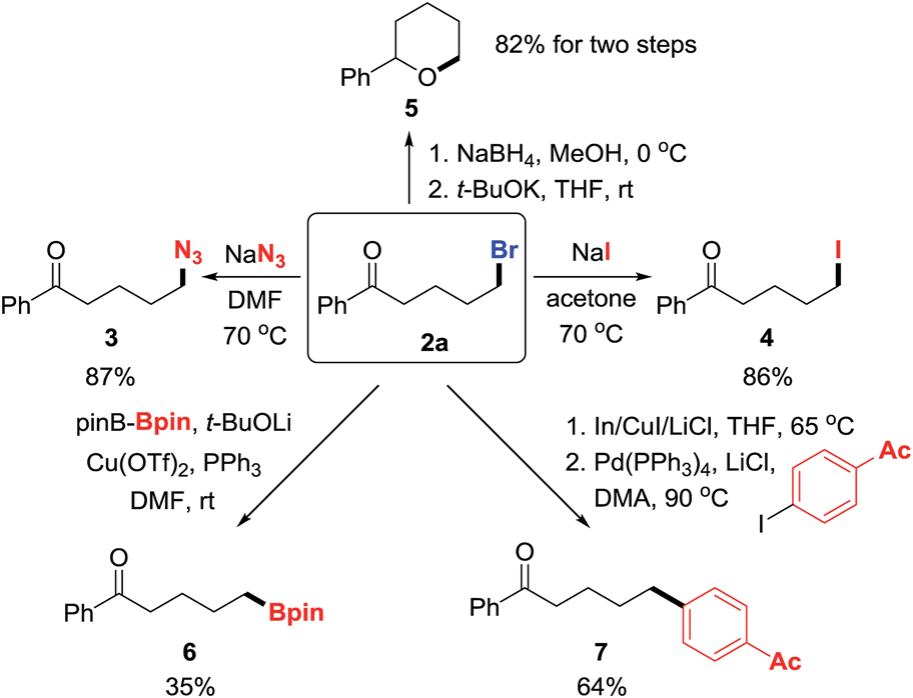
Transformation of products to other useful molecules.

To manifest the utility of the protocol, the products were converted to other valuable molecules *via* nucleophilic substitution or cross-coupling in one or two steps (Scheme 3). First, the bromide in **2a** was easily replaced by azide and iodide, forming new C–N_3_ and C–I bonds in good yields (**3** and **4**).^9^ Then, the ketone in **2a** was reduced to alcohol that intramolecularly attacked the alkyl bromide, leading to tetrahydropyran **5** in two steps with high yield.^10^ Alternatively, the C–Br bond was readily converted to C–B and C–C bonds *via* transition-metal catalyzed cross-coupling reactions, affording synthetically useful building blocks or molecules (**6** and **7**).^11^

**Scheme 4:**
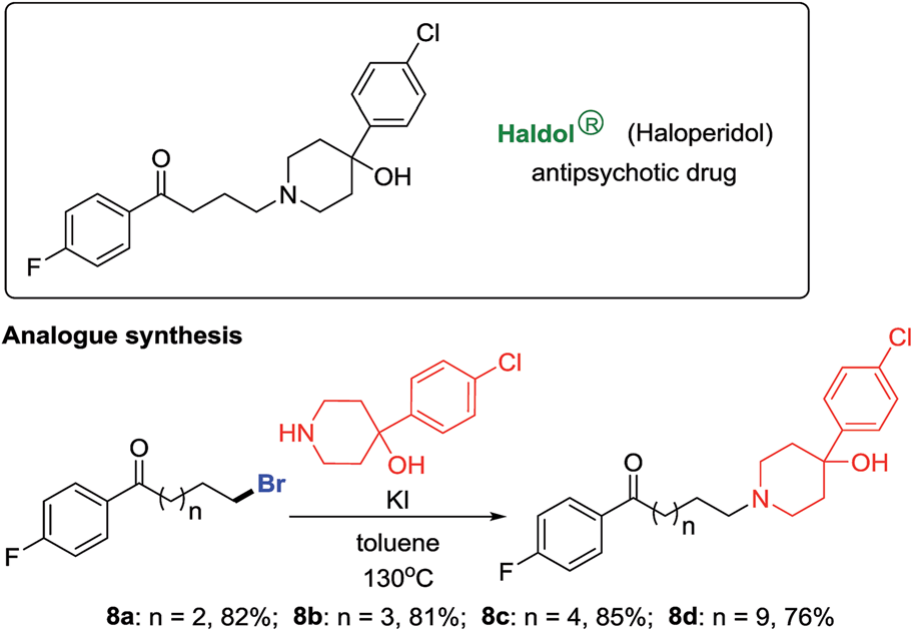
Production of haloperidol analogues.

Haloperidol is a marketed antipsychotic drug extensively used to treat schizophrenia and Tourette's syndrome. After gaining a portfolio of distal-bromo alkyl bromides as precursors, we accomplished the preparation of haloperidol analogues in high yields (**8a–8d**, Scheme 4).^12^ Testing of their biological activities is ongoing.

**Scheme 5:**
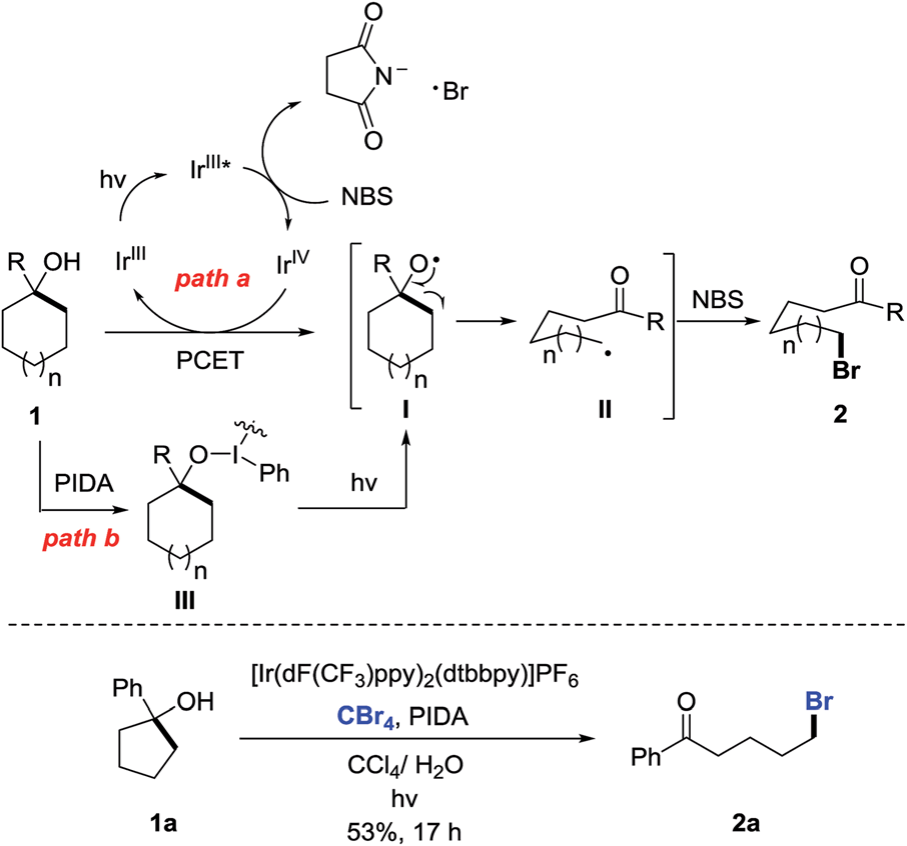
Plausible pathways of ring-opening bromination.

According to the experimental results, the plausible mechanism is depicted (Scheme 5, top). Although the detailed process for the generation of cycloalkoxy radical **I** is not fully understood, the radical **I** might be formed *via* two pathways. First, Stern–Volmer studies disclose that the excited state of Ir^III^ catalyst **PC 2** could be oxidatively quenched by NBS to generate the Ir^IV^ complex (see the ESI†). However, the oxidation potential of this Ir^IV^ complex (*E*_1/2_^IV/III^ = 1.21 V in MeCN *vs.* SCE) is insufficient to oxidize cycloalkanol **1** (*E*_p/2_ > 2.0 V in MeCN *vs.* SCE) to alkoxy radical **I***via* the SET process (for the cyclic voltammetry studies, see the ESI†). Thus, we postulate an intermolecular proton-coupled electron transfer (PCET) process for the generation of alkoxy radical **I** in the presence of an Ir^IV^ complex and weak base such as a succinimide anion (path a).^13^ Alternatively, homolysis of the O–I bond *in situ* formed by the reaction of cycloalkanol **1** with PIDA might also lead to the alkoxy radical **I** (path b).^14^ In either way, the visible-light irradiation is indispensable. The subsequent β-C scission of **I** leads to the ring-opened alkyl radical **II**, which is intercepted by NBS to give the final product **2**. Notably, CBr_4_ is also a competent bromine source *in lieu* of NBS to afford the same brominated product, suggesting the formation of intermediate **II** during the reaction (Scheme 5, bottom).

**Scheme 6:**
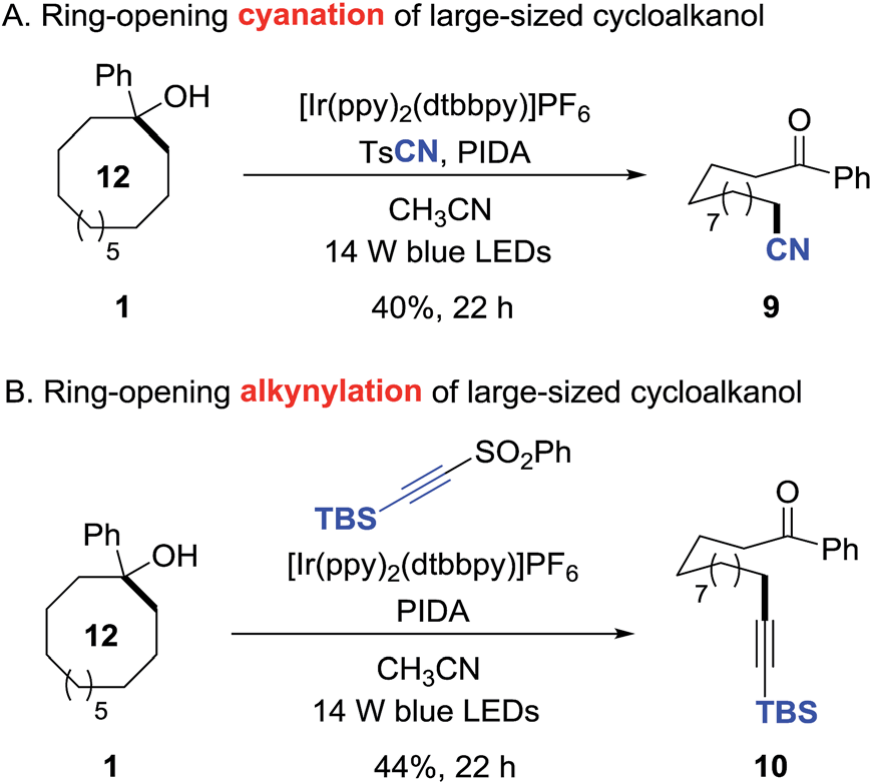
Ring-opening cyanation and alkynylation of unstrained cycloalkanols.

This protocol is also applicable to the challenging ring-opening cyanation and alkynylation of unstrained cycloalkanols.^15^ Under the similar reaction conditions, 1-phenyl cyclododecanol was readily converted to the distally cyano- or alkynyl-substituted alkyl ketones in synthetically useful yields (**9** and **10**), providing a non-trivial approach to construct remote C–C bonds (Scheme 6).

## Conclusions

We have described a novel and efficient visible light-enabled ring-opening functionalization of unstrained cycloalkanols *via* the mild cleavage of cyclic C–C σ-bonds. Ring opening of a variety of cyclopentanols, cyclohexanols, cycloheptanols, cyclododecanols, and cyclopentadecanols readily proceeded to furnish the distally brominated alkyl ketones that often function as precursors of complex molecules in organic and medicinal synthesis. As shown above, this method provides important feedstocks for the production of haloperidol analogues. The newly formed C–Br bonds in products are easily transformed into other valuable chemical bonds, thus illustrating the utility of this method. This protocol is also applicable to the elusive ring-opening cyanation and alkynylation of unstrained cycloalkanols, providing an unusual strategy for the construction of remote C–C bonds.

## Conflicts of interest

There are no conflicts to declare.

## Supplementary Material

Supplementary information
