# Supplementary material for: Visible light-promoted ring-opening functionalization of unstrained cycloalkanols via inert C–C bond scission
Source: Chem Sci. 2018 Jun 11;9(26):5805–9. doi: 10.1039/c8sc01763h (PMC6050598; doi:10.1039/c8sc01763h)
Supplement: Supplementary file 1 [file SC-009-C8SC01763H-s001.pdf]

Electronic Supplementary Material (ESI) for Chemical Science.
This journal is © The Royal Society of Chemistry 2018

Supporting Information

Visible light-Promoted Ring-Opening Functionalization of Unstrained Cycloalkanols via Inert C-C Bond Scission

Dongping Wang,† Jincheng Mao,§ and Chen Zhu*,†,‡

† Key Laboratory of Organic Synthesis of Jiangsu Province, College of Chemistry, Chemical Engineering and Materials Science, Soochow University, 199 Ren-Ai Road, Suzhou, Jiangsu 215123, China

‡ Key Laboratory of Synthesis Chemistry of Natural Substances, Shanghai Institute of Organic Chemistry, Chinese Academy of Science, 345 Lingling Road, Shanghai 200032, China

§ State Key Laboratory of Oil and Gas Reservoir Geology and Exploitation, Southwest Petroleum University, Chengdu 610500, China

Email: chzhu@suda.edu.cn

Table of Contents

1. General experimental details S2
2. General procedure for the ring-opening bromination reactions S2
3. Characterization of new starting materials S2
4. Characterization of products S4
5. Transformation of products S13
6. ^{1}H, ^{13}C, and ^{19}F NMR spectra S17
7. Cyclic voltammograms of **1a** and **1k** S96
8. Emission quenching experiments (Stern–Volmer studies) S98

1. General experimental details

All reactions were maintained under a nitrogen atmosphere unless otherwise stated. Commercially available reagents were used without further purification. Infrared (FT-IR) spectra were recorded on a BRUKER VERTEX 70, ν_{max} in cm^{-1}. ^{1}H-NMR spectra were recorded on a BRUKER AVANCE III HD (400 MHz) spectrometer. Chemical shifts are reported in ppm from tetramethylsilane with the solvent resonance as internal standard ($CDCl_3$: δ 7.26). Data are reported as follows: chemical shift, multiplicity (s = singlet, d = doublet, t = triplet, q = quadruplet, br = broad, m = multiplet), coupling constants (Hz) and integration. ^{13}C-NMR spectra were recorded on a BRUKER AVANCE III HD (100 MHz) spectrometer with complete proton decoupling. Chemical shifts are reported in ppm from tetramethylsilane with the solvent resonance as the internal standard ($CDCl_3$: δ 77.16). ^{19}F-NMR spectra were recorded on a BRUKER AVANCE III HD (376 MHz) spectrometer. Mass spectra were measured with an Agilent Technologies 6120 Quadrupole LC/MS. High resolution mass spectrometry (HRMS) were measured with a GCT Premier^{TM} and BRUKER micrOTF-Q III. Melting points were measured using INESA WRR and values are uncorrected.

2. General procedure for the ring-opening bromination reactions

Cycloalkanol **1** (0.2 mmol, 1.0 equiv.), photocatalyst $[Ir(ppy)_2(dtbbpy)]PF_6$ (5.5 mg, 0.006 mmol, 3 mol %), PIDA (128.8 mg, 0.4 mmol, 2.0 equiv.), and NBS (53.4 mg, 0.3 mmol, 1.5 equiv.) were loaded in a reaction vial which was subjected to evacuation/ flushing with N_2 three times. $PhCF_3$/ H_2O (2 ml/ 0.1 ml) or CCl_4/ H_2O (2 ml/ 0.1 ml) was added to the mixture via syringe and the reaction was irradiated with 14 W blue LEDs. After the reaction completion, the reaction mixture was extracted with ethyl acetate (3 x 10 mL). The combined organic extracts were washed by brine, dried over Na_2SO_4, filtered, concentrated, and purified by flash column chromatography on silica gel (eluent: ethyl acetate/ petroleum ether) to give the desired product **2**.

3. Characterization of new starting materials

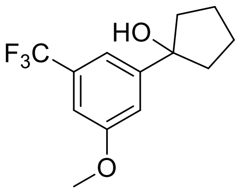

1h: white solid, m.p. 37-39 °C. ^{1}H NMR (400 MHz, $CDCl_3$) δ 7.33-7.31 (m, 1H), 7.24-7.22 (m, 1H), 7.01-6.99 (m, 1H), 3.85 (s, 3H), 2.05-1.82 (m, 8H), 1.75 (s, 1H); ^{13}C NMR (100 MHz, $CDCl_3$) δ 159.2, 149.5, 131.1 (q, J_{C-F} = 31.8 Hz), 123.6 (q, J_{C-F} = 270.9 Hz), 114.4, 113.8 (q, J_{C-F} = 3.9 Hz), 108.2 (q, J_{C-F} = 3.8 Hz), 82.7, 55.0, 41.8, 23.4; ^{19}F NMR (376 MHz, $CDCl_3$) δ -62.5 (s). FT-IR: ν (cm^{-1}) 3354, 2953, 2852, 2361, 1723, 1213. HRMS [ESI] calcd for $C_{13}H_{15}F_3O_2Na$ $[M+Na]^+$ 283.0916, found 283.0914.

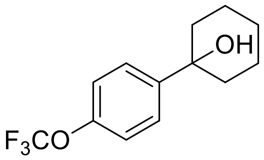

1p: white solid, m.p. 57-59 °C. ^{1}H NMR (400 MHz, $CDCl_3$) δ 7.56-7.49 (m, 2H), 7.21-7.15 (m, 2H), 1.85-1.60 (m, 10H), 1.35-1.22 (m, 1H); ^{13}C NMR (100 MHz, $CDCl_3$) δ 147.6, 147.4 (q, J_{C-F} = 16.4 Hz), 125.7, 120.1, 120.0 (q, J_{C-F} = 255.2 Hz), 72.4, 38.4, 24.9, 21.6; ^{19}F NMR (376 MHz, $CDCl_3$) δ -57.9 (s). FT-IR: ν (cm^{-1}) 3316, 2932, 2859, 1509, 1390,

1251. HRMS [ESI] calcd for $C_{13}H_{15}F_3O_2Na$ $[M+Na]^+$ 283.0916, found 283.0921.

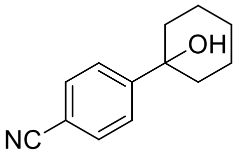

1q: white solid, m.p. 65-66 °C. ^1H NMR (400 MHz, $CDCl_3$) δ 7.62-7.54 (m, 5H), 2.23 (s, 1H), 1.80-1.60 (m, 9H), 1.33-1.21 (m, 1H); ^{13}C NMR (100 MHz, $CDCl_3$) δ 154.6, 131.5, 125.1, 118.5, 109.7, 72.8, 38.0, 24.8, 21.3. FT-IR: ν (cm^{-1}) 3338, 2936, 2864, 2230, 1607, 1264. HRMS [ESI] calcd for $C_{13}H_{15}NONa$ $[M+Na]^+$ 224.1046, found 224.1033.

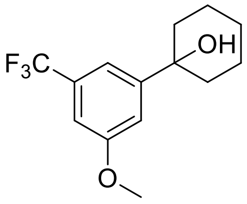

1s: yellow solid, m.p. 64-66 °C. ^1H NMR (400 MHz, $CDCl_3$) δ 7.35 (s, 1H), 7.28-7.25 (m, 1H), 7.01 (s, 1H), 3.86 (s, 3H), 1.87-1.59 (m, 10H), 1.38-1.25 (m, 1H); ^{13}C NMR (100 MHz, $CDCl_3$) δ 159.3, 151.9, 131.1 (q, J_{C-F} = 31.7 Hz), 123.6 (q, J_{C-F} = 270.9 Hz), 114.0 (q, J_{C-F} = 0.9 Hz), 113.4 (d, J_{C-F} = 3.9 Hz), 108.1 (q, J_{C-F} = 3.8 Hz), 72.7, 55.0, 38.3, 24.8, 21.5; ^{19}F NMR (376 MHz, $CDCl_3$) δ -62.5 (s). FT-IR: ν (cm^{-1}) 3349, 3007, 2944, 2856, 1601, 1258. HRMS [ESI] calcd for $C_{14}H_{18}F_3O_2$ $[M+H]^+$ 275.1253, found 275.1257.

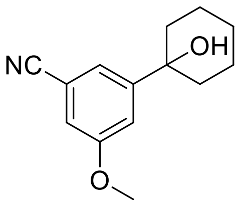

1t: yellow oil. ^1H NMR (400 MHz, $CDCl_3$) δ 7.36 (dd, J = 1.6, 1.6 Hz, 1H), 7.30 (dd, J = 2.4, 1.6 Hz, 1H), 6.98 (dd, J = 2.4, 1.2 Hz, 1H), 3.82 (s, 3H), 1.90 (s, 1H), 1.82-1.60 (m, 9H), 1.34-1.20 (m, 1H); ^{13}C NMR (100 MHz, $CDCl_3$) δ 159.2, 152.4, 120.8, 118.6, 115.8, 114.2, 112.3, 72.5, 55.1, 38.2, 24.8, 21.4. FT-IR: ν (cm^{-1}) 3448, 2929, 2853, 2231, 1590, 1260. HRMS [ESI] calcd for $C_{14}H_{17}NO_2Na$ $[M+Na]^+$ 254.1151, found 254.1156.

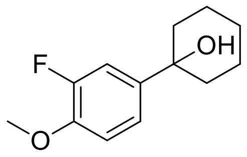

1u: yellow solid, m.p. 54-55 °C. ^1H NMR (400 MHz, $CDCl_3$) δ 7.23 (dd, J = 13.2, 2.4 Hz, 1H), 7.20-7.16 (m, 1H), 6.92 (dd, J = 8.8, 8.8 Hz, 1H), 3.87 (s, 3H), 1.80-1.58 (m, 10H), 1.32-1.20 (m, 1H); ^{13}C NMR (100 MHz, $CDCl_3$) δ 151.6 (d, J_{C-F} = 243.2 Hz), 145.6 (d, J_{C-F} = 10.8 Hz), 142.4 (d, J_{C-F} = 4.9 Hz), 119.7 (d, J_{C-F} = 3.5 Hz), 112.6 (d, J_{C-F} = 6.9 Hz), 112.5 (d, J_{C-F} = 14.2 Hz), 72.1, 55.8, 38.4, 24.9, 21.7; ^{19}F NMR (376 MHz, $CDCl_3$) δ -135.2 (s). FT-IR: ν (cm^{-1}) 3400, 3017, 2926, 2858, 1624, 1270. HRMS [ESI] calcd for $C_{13}H_{17}FO_2Na$ $[M+Na]^+$ 247.1105, found 247.1117.

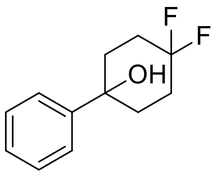

1ab: red solid, m.p. 50-51 °C. ^1H NMR (400 MHz, $CDCl_3$) δ 7.54-7.47 (m, 2H), 7.42-7.35 (m, 2H), 7.33-7.27 (m, 1H), 2.40-2.00 (m, 6H), 1.92-1.84 (m, 2H), 1.64 (s, 1H); ^{13}C NMR (100 MHz, $CDCl_3$) δ 146.9, 128.0, 126.9, 123.9, 123.1 (dd, J_{C-F} = 241.3, 237.1 Hz), 71.4 (d, J_{C-F} = 1.7 Hz), 34.9 (d, J_{C-F} = 9.7 Hz), 29.4 (dd, J_{C-F} = 25.0, 23.6 Hz); ^{19}F NMR (376 MHz, $CDCl_3$) δ -92.4 (d, J = -235.3 Hz), -104.2 (d, J = -235.3 Hz). FT-IR: ν (cm^{-1}) 3577, 3450, 3089, 2855, 1493, 1286. HRMS [ESI] calcd for $C_{12}H_{15}F_2O$ $[M+H]^+$ 213.1085, found 213.1089.

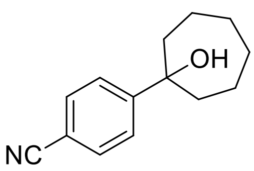

1ai: yellow solid, m.p. 57-59 °C. ^1H NMR (400 MHz, $CDCl_3$) δ 7.63-7.55 (m, 4H), 2.05-1.93 (m, 3H), 1.88-1.54 (m, 10H); ^{13}C NMR (100 MHz, $CDCl_3$) δ 155.8, 131.5, 124.9, 118.5, 109.7, 76.3, 42.6, 28.3, 22.0. FT-IR: ν (cm^{-1}) 3458, 3342, 2920, 2226, 1459, 1245. HRMS [ESI] calcd for $C_{14}H_{17}NONa$ $[M+Na]^+$ 238.1202, found 238.1196.

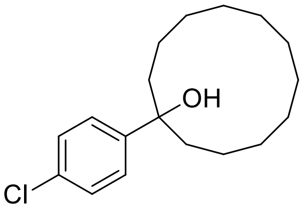

1an: white solid, m.p. 141-143 °C. ^{1}H NMR (400 MHz, $CDCl_3$) δ 7.42-7.36 (m, 2H), 7.30-7.25 (m, 2H), 1.89-1.75 (m, 5H), 1.44-1.12 (m, 18H); ^{13}C NMR (100 MHz, $CDCl_3$) δ 146.4, 132.0, 127.5, 126.3, 75.7, 34.9, 25.8, 25.5, 21.9, 21.6, 19.4. FT-IR: ν (cm^{-1}) 3268, 2926, 2848, 1487, 1397, 1252. HRMS [ESI] calcd for $C_{18}H_{27}ClONa$ $[M+Na]^+$ 317.1643, found 317.1651.

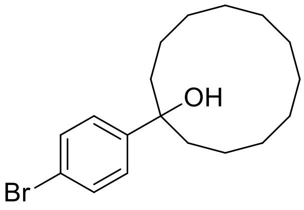

1ao: white solid, m.p. 145-146 °C. ^{1}H NMR (400 MHz, $CDCl_3$) δ 7.47-7.41 (m, 2H), 7.37-7.31 (m, 2H), 1.87-1.77 (m, 4H), 1.71 (s, 1H), 1.44-1.15 (m, 18H); ^{13}C NMR (100 MHz, $CDCl_3$) δ 146.9, 130.5, 126.7, 120.2, 75.8, 34.9, 25.8, 25.5, 22.0, 21.6, 19.4. FT-IR: ν (cm^{-1}) 3270, 2926, 2848, 1485, 1394, 1252. HRMS [ESI] calcd for $C_{18}H_{27}BrONa$ $[M+Na]^+$ 361.1137, found 361.1130.

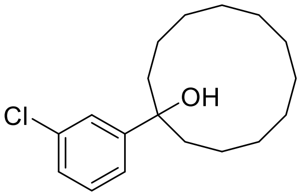

1ap: white solid, m.p. 138-139 °C. ^{1}H NMR (400 MHz, $CDCl_3$) δ 7.48 (dd, *J* = 1.6, 1.6 Hz, 1H), 7.37-7.33 (m, 1H), 7.29-7.20 (m, 2H), 1.95 (s, 1H), 1.88-1.78 (m, 4H), 1.46-1.16 (m, 18H); ^{13}C NMR (100 MHz, $CDCl_3$) δ 150.5, 134.0, 129.2, 126.8, 125.7, 123.6, 76.3, 35.4, 26.2, 26.0, 22.5, 22.1, 19.8. FT-IR: ν (cm^{-1}) 3235, 2942, 2847, 1593, 1417, 1250. HRMS [ESI] calcd for $C_{18}H_{27}ClONa$ $[M+Na]^+$ 317.1643, found 317.1642.

4. Characterization of products

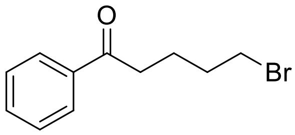

2a: yellow solid, m.p. 53-55 °C. ^{1}H NMR (400 MHz, $CDCl_3$) δ 7.99-7.86 (m, 2H), 7.56-7.49 (m, 1H), 7.46-7.39 (m, 2H), 3.41 (t, *J* = 6.4 Hz, 2H), 2.97 (t, *J* = 6.8 Hz, 2H), 2.05-1.75 (m, 4H); ^{13}C NMR (100 MHz, $CDCl_3$) δ 199.0, 136.3, 132.6, 128.1, 127.5, 36.9, 33.0, 31.7, 22.3. FT-IR: ν (cm^{-1}) 3653, 3335, 3060, 2866, 1674, 1343. HRMS [ESI] calcd for $C_{11}H_{13}BrONa$ $[M+Na]^+$ 263.0042, found 263.0048.

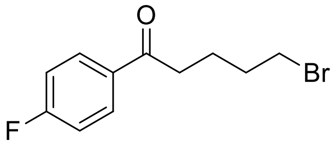

2b: yellow oil. ^{1}H NMR (400 MHz, $CDCl_3$) δ 8.02-7.95 (m, 2H), 7.17-7.09 (m, 2H), 3.45 (t, *J* = 6.4 Hz, 2H), 2.99 (t, *J* = 6.8 Hz, 2H), 2.05-1.83 (m, 4H); ^{13}C NMR (100 MHz, $CDCl_3$) δ 197.4, 165.3 (d, $J_{C\text{-}F}$ = 253.2 Hz), 132.8 (d, $J_{C\text{-}F}$ = 3.0 Hz), 130.2 (d, $J_{C\text{-}F}$ = 9.2 Hz), 115.2 (d, $J_{C\text{-}F}$ = 21.7 Hz), 36.9, 32.8, 31.7, 22.2; ^{19}F NMR (376 MHz, $CDCl_3$) δ -105.3 (s). FT-IR: ν (cm^{-1}) 3073, 2962, 2866, 1677, 1459, 1272. HRMS [ESI] calcd for $C_{11}H_{12}BrFONa$ $[M+Na]^+$ 280.9948, found 280.9955.

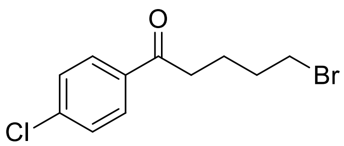

2c: yellow solid, m.p. 41-42 °C. ^{1}H NMR (400 MHz, $CDCl_3$) δ 7.94-7.85 (m, 2H), 7.48-7.39 (m, 2H), 3.45 (t, *J* = 6.4 Hz, 2H), 2.98 (t, *J* = 6.8 Hz, 2H), 2.02-1.84 (m, 4H); ^{13}C NMR (100 MHz, $CDCl_3$) δ 197.8, 139.1, 134.6, 129.0, 128.5, 36.9, 32.8, 31.6, 22.2.

FT-IR: ν (cm^{-1}) 3036, 2933, 2851, 1934, 1676, 1255. HRMS [ESI] calcd for $C_{11}H_{12}BrClONa$ [M+Na]$^+$ 296.9652, found 296.9648.

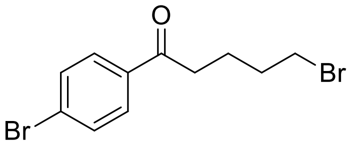

2d: yellow solid, m.p. 50-52 ^oC. ^1H NMR (400 MHz, $CDCl_3$) δ 7.85-7.78 (m, 2H), 7.64-7.56 (m, 2H), 3.45 (t, *J* = 6.4 Hz, 2H), 2.97 (t, *J* = 6.8 Hz, 2H), 2.00-1.82 (m, 4H); ^{13}C NMR (100 MHz, $CDCl_3$) δ 198.0, 135.0, 131.5, 129.1, 127.8, 36.9, 32.8, 31.6, 22.2. FT-IR: ν (cm^{-1}) 2942, 2861, 2168, 1686, 1466, 1221. HRMS [ESI] calcd for $C_{11}H_{12}Br_2ONa$ [M+Na]$^+$ 340.9147, found 340.9157.

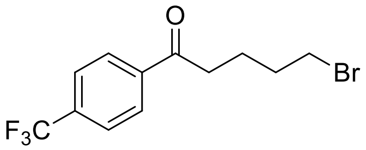

2e: yellow solid, m.p. 33-34 ^oC. ^1H NMR (400 MHz, $CDCl_3$) δ 8.05 (d, *J* = 8.0 Hz, 2H), 7.73 (d, *J* = 8.0 Hz, 2H), 3.46 (t, *J* = 6.4 Hz, 2H), 3.04 (t, *J* = 6.8 Hz, 2H), 2.02-1.86 (m, 4H); ^{13}C NMR (100 MHz, $CDCl_3$) δ 198.5, 139.4 (q, J_{C-F} = 1.0 Hz), 134.4 (q, J_{C-F} = 32.5 Hz), 128.3, 125.7 (q, J_{C-F} = 3.8 Hz), 123.6 (q, J_{C-F} = 270.9 Hz), 37.8, 33.2, 32.0, 22.5; ^{19}F NMR (376 MHz, $CDCl_3$) δ -63.1 (s). FT-IR: ν (cm^{-1}) 2940, 2869, 1690, 1511, 1409, 1209. HRMS [ESI] calcd for $C_{12}H_{12}BrF_3ONa$ [M+Na]$^+$ 330.9916, found 330.9925.

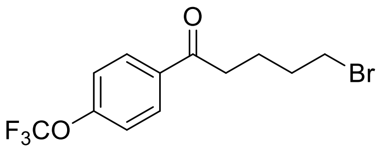

2f: yellow oil. ^1H NMR (400 MHz, $CDCl_3$) δ 8.04-7.98 (m, 2H), 7.29 (d, *J* = 8.0 Hz, 2H), 3.45 (t, *J* = 6.4 Hz, 2H), 3.00 (t, *J* = 6.8 Hz, 2H), 2.02-1.85 (m, 4H); ^{13}C NMR (100 MHz, CDCl3) δ 197.9, 152.6 (q, J_{C-F} = 1.7 Hz), 135.0, 130.0, 120.4 (q, J_{C-F} = 0.7 Hz), 120.3 (q, J_{C-F} = 257.2 Hz), 37.5, 33.2, 32.1, 22.6; ^{19}F NMR (376 MHz, $CDCl_3$) δ -57.6 (s). FT-IR: ν (cm^{-1}) 2940, 2871, 1687, 1505, 1413, 1207. HRMS [ESI] calcd for $C_{12}H_{12}BrF_3O_2Na$ [M+Na]$^+$ 346.9865, found 346.9858.

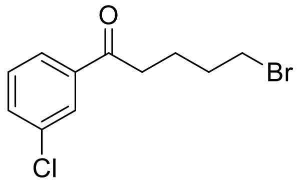

2g: yellow oil. ^1H NMR (400 MHz, $CDCl_3$) δ 7.92 (dd, *J* = 1.6, 1.6 Hz, 1H), 7.85-7.80 (m, 1H), 7.56-7.51 (m, 1H), 7.41 (dd, *J* = 6.8, 6.8 Hz, 1H), 3.45 (t, *J* = 6.4 Hz, 2H), 2.99 (t, *J* = 6.8 Hz, 2H), 2.04-1.78 (m, 4H); ^{13}C NMR (100 MHz, $CDCl_3$) δ 197.7, 137.9, 134.5, 132.6, 129.5, 127.7, 125.6, 37.1, 32.7, 31.6, 22.1. FT-IR: ν (cm^{-1}) 3064, 2919, 2850, 1686, 1469, 1256. HRMS [ESI] calcd for $C_{11}H_{12}BrClONa$ [M+Na]$^+$ 296.9652 found 296.9656.

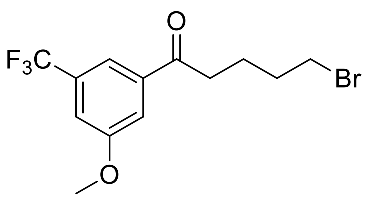

2h: white solid, m.p. 51-53 ^oC. ^1H NMR (400 MHz, $CDCl_3$) δ 7.77-7.74 (m, 1H), 7.65-7.63 (m, 1H), 7.33-7.31 (m, 1H), 3.90 (s, 3H), 3.46 (t, *J* = 6.4 Hz, 2H), 3.02 (t, *J* = 6.8 Hz, 2H), 2.01-1.87 (m, 4H); ^{13}C NMR (100 MHz, $CDCl_3$) δ 198.0, 160.2, 138.8, 132.3 (q, J_{C-F} = 32.7 Hz), 123.5 (q, J_{C-F} = 271.1 Hz), 117.0 (q, J_{C-F} = 3.8 Hz), 116.0 (q, J_{C-F} = 0.5 Hz), 115.7 (q, J_{C-F} = 3.7 Hz), 55.9, 37.6, 33.2, 32.0, 22.5; ^{19}F NMR (376 MHz, $CDCl_3$) δ -62.8 (s). FT-IR: ν (cm^{-1}) 2954, 2873, 2360, 1684, 1408, 1243. HRMS [ESI] calcd for $C_{13}H_{14}BrF_3O_2Na$ [M+Na]$^+$ 361.0021, found 361.0020.

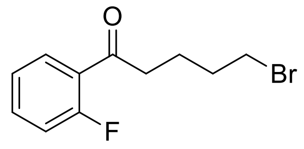

2i: yellow oil. ^{1}H NMR (400 MHz, $CDCl_3$) δ 7.86 (ddd, *J* = 7.6, 7.6, 1.6 Hz, 1H), 7.57-7.46 (m, 1H), 7.23 (ddd, *J* = 7.6, 7.6, 0.8 Hz, 1H), 7.17-7.10 (m, 1H), 3.44 (t, *J* = 6.4 Hz, 2H), 3.02 (td, *J* = 6.8, 3.2 Hz, 2H), 2.00-1.80 (m, 4H); ^{13}C NMR (100 MHz, $CDCl_3$) δ 198.0 (d, J_{C-F} = 4.1 Hz), 161.9 (d, J_{C-F} = 252.6 Hz), 134.5 (d, J_{C-F} = 9.0 Hz), 130.6 (d, J_{C-F} = 2.7 Hz), 125.6 (d, J_{C-F} = 13.0 Hz), 124.5 (d, J_{C-F} = 3.3 Hz), 116.7 (d, J_{C-F} = 23.8 Hz), 42.5 (d, J_{C-F} = 7.3 Hz), 33.3, 32.2, 22.5 (d, J_{C-F} = 2.0 Hz); ^{19}F NMR (376 MHz, $CDCl_3$) δ -109.5 (s). FT-IR: ν (cm^{-1}) 2930, 2869, 1685, 1479, 1365, 1210. HRMS [ESI] calcd for $C_{11}H_{12}BrFONa$ $[M+Na]^+$ 280.9948, found 280.9958.

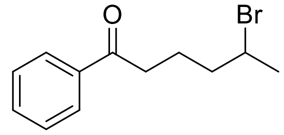

2j: yellow oil. ^{1}H NMR (400 MHz, $CDCl_3$) δ 8.00-7.92 (m, 2H), 7.61-7.53 (m, 1H), 7.51-7.43 (m, 2H), 4.23-4.12 (m, 1H), 3.06-2.94 (m, 2H), 2.04-1.79 (m, 4H), 1.74 (d, *J* = 6.8 Hz, 3H); ^{13}C NMR (100 MHz, $CDCl_3$) δ 199.7, 136.8, 133.1, 128.6, 128.0, 51.2, 40.5, 37.6, 26.4, 22.4. FT-IR: ν (cm^{-1}) 2964, 2869, 2361, 1683, 1448, 1244. HRMS [ESI] calcd for $C_{12}H_{15}BrONa$ $[M+Na]^+$ 277.0198, found 277.0196.

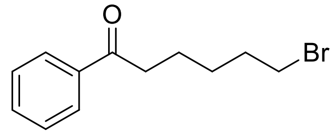

2k: yellow solid, m.p. 33-35 °C. ^{1}H NMR (400 MHz, $CDCl_3$) δ 8.00-7.92 (m, 2H), 7.59 -7.52 (m, 1H), 7.49-7.43 (m, 2H), 3.43 (t, *J* = 6.8 Hz, 2H), 3.00 (t, *J* = 7.2 Hz, 2H), 2.01-1.87 (m, 2H), 1.83-1.72 (m, 2H), 1.60-1.48 (m, 2H); ^{13}C NMR (100 MHz, $CDCl_3$) δ 199.5, 136.5, 132.5, 128.1, 127.5, 37.8, 33.2, 32.2, 27.4, 22.8. FT-IR: ν (cm^{-1}) 3057, 2946, 2861, 1969, 1678, 1263. HRMS [ESI] calcd for $C_{12}H_{15}BrONa$ $[M+Na]^+$ 277.0198, found 277.0204.

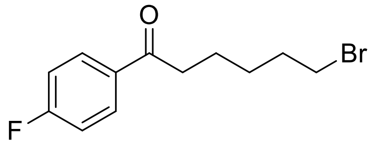

2l: yellow oil. ^{1}H NMR (400 MHz, $CDCl_3$) δ 8.02-7.94 (m, 2H), 7.16-7.09 (m, 2H), 3.43 (t, *J* = 6.8 Hz, 2H), 2.96 (t, *J* = 7.2 Hz, 2H), 1.96-1.87 (m, 2H), 1.81-1.71 (m, 2H), 1.58-1.48 (m, 2H); ^{13}C NMR (100 MHz, $CDCl_3$) δ 197.9, 165.2 (d, J_{C-F} = 253.0 Hz), 132.9 (d, J_{C-F} = 3.0 Hz), 130.1 (d, J_{C-F} = 9.2 Hz), 115.2 (d, J_{C-F} = 21.7 Hz), 37.7, 33.1, 32.1, 27.4, 22.8; ^{19}F NMR (376 MHz, $CDCl_3$) δ -105.4 (s). FT-IR: ν (cm^{-1}) 3053, 2936, 2864, 1917, 1723, 1262. HRMS [ESI] calcd for $C_{12}H_{15}BrFO$ $[M+H]^+$ 273.0285, found 273.0294.

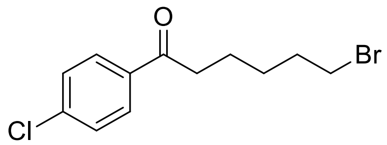

2m: yellow solid, m.p. 38-40 °C. ^{1}H NMR (400 MHz, $CDCl_3$) δ 7.92-7.86 (m, 2H), 7.45-7.40 (m, 2H), 3.42 (t, *J* = 6.8 Hz, 2H), 2.96 (t, *J* = 7.2 Hz, 2H), 1.96-1.86 (m, 2H), 1.81-1.71 (m, 2H), 1.58-1.48 (m, 2H); ^{13}C NMR (100 MHz, $CDCl_3$) δ 198.2, 139.0, 134.8, 128.9, 128.4, 37.8, 33.1, 32.1, 27.3, 22.7. FT-IR: ν (cm^{-1}) 3092, 2962, 2838, 1937, 1681, 1268. HRMS [ESI] calcd for $C_{12}H_{15}BrClO$ $[M+H]^+$ 288.9989, found 288.9993.

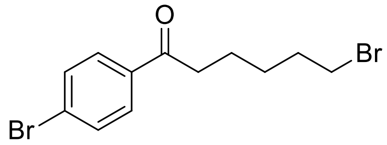

2n: yellow solid, m.p. 41-43 °C. ^{1}H NMR (400 MHz, $CDCl_3$) δ 7.85-7.76 (m, 2H), 7.64-7.56 (m, 2H), 3.42 (t, *J* = 6.8 Hz, 2H), 2.95 (t, *J* = 7.2 Hz, 2H), 1.96-1.86 (m, 2H), 1.81-1.70 (m, 2H), 1.57-1.46 (m, 2H); ^{13}C NMR (100 MHz, $CDCl_3$) δ 198.4,

135.2, 131.4, 129.1, 127.7, 37.8, 33.1, 32.1, 27.3, 22.7. FT-IR: ν (cm^{-1}) 3090, 2959, 2861, 1937, 1681, 1269. HRMS [ESI] calcd for $C_{12}H_{15}Br_2O$ $[M+H]^+$ 332.9484, found 332.9498.

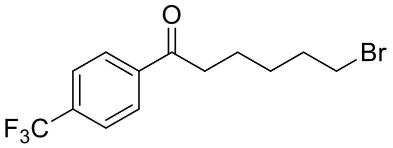

2o: yellow oil. ^1H NMR (400 MHz, $CDCl_3$) δ 8.05 (d, *J* = 8.0 Hz, 2H), 7.73 (d, *J* = 8.0 Hz, 2H), 3.43 (t, *J* = 6.8 Hz, 2H), 3.02 (t, *J* = 7.2 Hz, 2H), 1.97-1.88 (m, 2H), 1.83-1.74 (m, 2H), 1.58-1.49 (m, 2H); ^{13}C NMR (100 MHz, $CDCl_3$) δ 198.9, 139.5, 134.3 (q, $J_{C\text{-}F}$ = 32.3 Hz), 128.3, 125.7 (q, $J_{C\text{-}F}$ = 3.5 Hz), 123.6 (q, $J_{C\text{-}F}$ = 271.4 Hz), 38.6, 33.5, 32.6, 27.8, 23.1; ^{19}F NMR (376 MHz, $CDCl_3$) δ -63.1 (s). FT-IR: ν (cm^{-1}) 2961, 2869, 2361, 1692, 1511, 1258. HRMS [ESI] calcd for $C_{13}H_{15}BrF_3O$ $[M+H]^+$ 323.0253, found 323.0245.

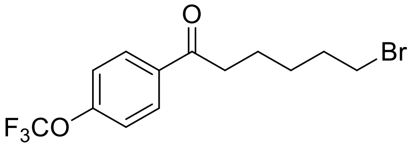

2p: yellow oil. ^1H NMR (400 MHz, $CDCl_3$) δ 8.01 (d, *J* = 8.8 Hz, 2H), 7.29 (d, *J* = 8.4 Hz, 2H), 3.43 (t, *J* = 6.8 Hz, 2H), 2.98 (t, *J* = 7.2 Hz, 2H), 2.00-1.86 (m, 2H), 1.83-1.72 (m, 2H), 1.58-1.48 (m, 2H); ^{13}C NMR (100 MHz, $CDCl_3$) δ 198.3, 152.6 (q, $J_{C\text{-}F}$ = 1.6 Hz), 135.2, 130.0, 120.4, 120.3 (q, $J_{C\text{-}F}$ = 257.2 Hz), 38.3, 33.6, 32.6, 27.8, 23.2; ^{19}F NMR (376 MHz, $CDCl_3$) δ -57.6 (s). FT-IR: ν (cm^{-1}) 2938, 2864, 1687, 1505, 1412, 1251. HRMS [ESI] calcd for $C_{13}H_{15}BrF_3O_2$ $[M+H]^+$ 339.0202, found 339.0197.

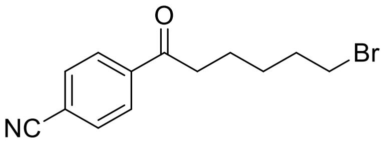

2q: yellow solid, m.p. 49-51 °C. ^1H NMR (400 MHz, $CDCl_3$) δ 8.04 (d, *J* = 8.4 Hz, 2H), 7.77 (d, *J* = 8.4 Hz, 2H), 3.43 (t, *J* = 6.8 Hz, 2H), 3.01 (t, *J* = 7.2 Hz, 2H), 1.96-1.87 (m, 2H), 1.83-1.73 (m, 2H), 1.58-1.48 (m, 2H); ^{13}C NMR (100 MHz, $CDCl_3$) δ 198.0, 139.4, 132.1, 127.9, 117.5, 115.8, 38.1, 33.0, 32.0, 27.2, 22.5. FT-IR: ν (cm^{-1}) 3093, 2952, 2849, 2232, 1687, 1216. HRMS [ESI] calcd for $C_{13}H_{14}BrNONa$ $[M+Na]^+$ 302.0151, found 302.0139.

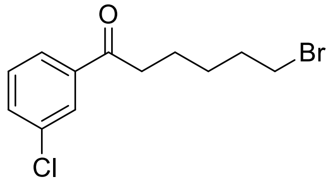

2r: yellow oil. ^1H NMR (400 MHz, $CDCl_3$) δ 7.93-7.90 (m, 1H), 7.84-7.80 (m, 1H), 7.55-7.51 (m, 1H), 7.41 (dd, *J* = 8.0, 8.0 Hz, 1H), 3.43 (t, *J* = 6.8 Hz, 2H), 2.97 (t, *J* = 7.2 Hz, 2H), 1.96-1.86 (m, 2H), 1.81-1.72 (m, 2H), 1.58-1.49 (m, 2H); ^{13}C NMR (100 MHz, $CDCl_3$) δ 198.1, 138.0, 134.5, 132.5, 129.5, 127.7, 125.6, 37.9, 33.1, 32.1, 27.3, 22.6. FT-IR: ν (cm^{-1}) 3066, 2937, 2859, 1687, 1459, 1205. HRMS [ESI] calcd for $C_{12}H_{15}BrClO$ $[M+H]^+$ 288.9989, found 288.9981.

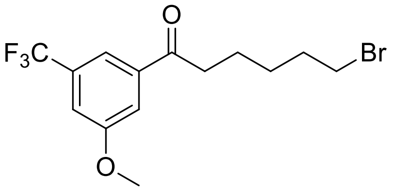

2s: yellow oil. ^1H NMR (400 MHz, $CDCl_3$) δ 7.75 (s, 1H), 7.64 (s, 1H), 7.31 (s, 1H), 3.90 (s, 3H), 3.43 (t, *J* = 6.8 Hz, 2H), 3.00 (t, *J* = 7.2 Hz, 2H), 1.97-1.88 (m, 2H), 1.83-1.73 (m, 2H), 1.58-1.49 (m, 2H); ^{13}C NMR (100 MHz, $CDCl_3$) δ 198.4, 160.1, 138.9, 132.3 (q, $J_{C\text{-}F}$ = 32.6 Hz), 123.5 (q, $J_{C\text{-}F}$ = 271.0 Hz), 117.1 (q, $J_{C\text{-}F}$ = 3.8 Hz), 116.0 (q, $J_{C\text{-}F}$ = 0.4 Hz), 115.6 (q, $J_{C\text{-}F}$ = 3.7 Hz), 55.9, 38.4, 33.5, 32.6, 27.8, 23.1; ^{19}F NMR (376 MHz, $CDCl_3$) δ

-62.8 (s). FT-IR: ν (cm^{-1}) 3069, 2963, 2852, 1684, 1407, 1266. HRMS [ESI] calcd for $C_{14}H_{16}BrF_3O_2Na$ [M+Na]$^+$ 375.0178, found 375.0171.

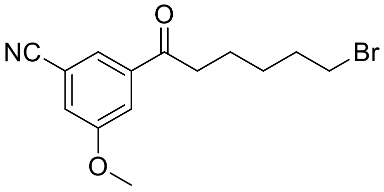

2t: yellow solid, m.p. 54-55 ^{o}C. ^{1}H NMR (400 MHz, $CDCl_3$) δ 7.78 (dd, *J* = 1.2, 1.2 Hz, 1H), 7.68 (dd, *J* = 2.4, 1.2 Hz, 1H), 7.32 (dd, *J* = 2.4, 1.2 Hz, 1H), 3.89 (s, 3H), 3.43 (t, *J* = 6.4 Hz, 2H), 2.97 (t, *J* = 7.2 Hz, 2H), 1.96-1.87 (m, 2H), 1.80-1.72 (m, 2H), 1.58-1.48 (m, 2H); ^{13}C NMR (100 MHz, $CDCl_3$) δ 197.2, 159.6, 138.6, 123.5, 121.0, 117.4, 117.1, 113.3, 55.5, 38.0, 33.0, 32.0, 27.2, 22.5. FT-IR: ν (cm^{-1}) 3079, 2967, 2839, 2232, 1682, 1262. HRMS [ESI] calcd for $C_{14}H_{16}BrNO_2Na$ [M+Na]$^+$ 332.0257, found 332.0270.

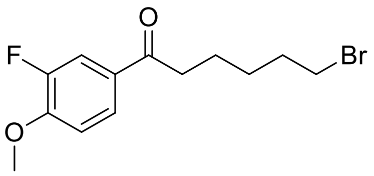

2u: yellow solid, m.p. 52-54 ^{o}C. ^{1}H NMR (400 MHz, $CDCl_3$) δ 7.76-7.71 (m, 1H), 7.69 (dd, *J* = 12.0, 2.0 Hz, 1H), 6.99 (dd, *J* = 8.4, 8.4 Hz, 1H), 3.95 (s, 3H), 3.42 (t, *J* = 6.8 Hz, 2H), 2.92 (t, *J* = 7.2 Hz, 2H), 1.95-1.85 (m, 2H), 1.80-1.70 (m, 2H), 1.57-1.46 (m, 2H); ^{13}C NMR (100MHz, $CDCl_3$) δ 197.7 (d, $J_{C\text{-}F}$ = 1.7 Hz), 152.0 (d, $J_{C\text{-}F}$ = 246.3 Hz), 151.8 (d, $J_{C\text{-}F}$ = 10.8 Hz), 130.3 (d, $J_{C\text{-}F}$ = 4.8 Hz), 125.3 (d, $J_{C\text{-}F}$ = 3.4 Hz), 115.7 (d, $J_{C\text{-}F}$ = 18.8 Hz), 112.3 (d, $J_{C\text{-}F}$ = 1.8 Hz), 56.3, 37.9, 33.6, 32.6, 27.9, 23.4; ^{19}F NMR (376 MHz, $CDCl_3$) δ -134.3 (s). FT-IR: ν (cm^{-1}) 3077, 2963, 2854, 1676, 1465, 1218. HRMS [ESI] calcd for $C_{13}H_{16}BrFO_2Na$ [M+Na]$^+$ 325.0210, found 325.0204.

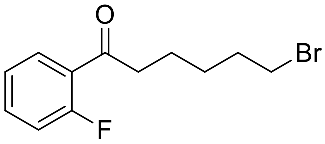

2v: yellow oil. ^{1}H NMR (400 MHz, $CDCl_3$) δ 7.85 (ddd, *J* = 7.6, 7.6, 2.0 Hz, 1H), 7.54-7.47 (m, 1H), 7.22 (ddd, *J* = 7.6, 7.6, 0.8 Hz, 1H), 7.13 (ddd, *J* = 11.2, 8.4, 0.8 Hz, 1H), 3.42 (t, *J* = 6.8 Hz, 2H), 3.00 (td, *J* = 7.2, 3.2 Hz, 2H), 1.96-1.86 (m, 2H), 1.80-1.70 (m, 2H), 1.57-1.47 (m, 2H); ^{13}C NMR (100 MHz, $CDCl_3$) δ 198.0 (d, $J_{C\text{-}F}$ = 4.2 Hz), 161.4 (d, $J_{C\text{-}F}$ = 252.7 Hz), 133.9 (d, $J_{C\text{-}F}$ = 9.0 Hz), 130.1 (d, $J_{C\text{-}F}$ = 2.7 Hz), 125.3 (d, $J_{C\text{-}F}$ = 13.1 Hz), 124.0 (d, $J_{C\text{-}F}$ = 3.4 Hz), 116.2 (d, $J_{C\text{-}F}$ = 23.8 Hz), 42.8 (d, $J_{C\text{-}F}$ = 7.0 Hz), 33.1, 32.1, 27.3, 22.6 (d, $J_{C\text{-}F}$ = 1.9 Hz); ^{19}F NMR (376 MHz, $CDCl_3$) δ -109.6 (s). FT-IR: ν (cm^{-1}) 3080, 2933, 2858, 1685, 1404, 1210. HRMS [ESI] calcd for $C_{12}H_{15}BrFO$ [M+H]$^+$ 273.0285, found 273.0298.

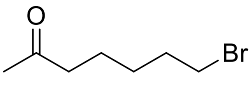

2w: yellow oil. ^{1}H NMR (400 MHz, $CDCl_3$) δ 3.40 (t, *J* = 6.8 Hz, 2H), 2.45 (t, *J* = 7.2 Hz, 2H), 2.14 (s, 3H), 1.92-1.81 (m, 2H), 1.65-1.55 (m, 2H), 1.48-1.38 (m, 2H); ^{13}C NMR (100 MHz, $CDCl_3$) δ 208.7, 43.4, 33.6, 32.5, 30.0, 27.7, 22.8. FT-IR: ν (cm^{-1}) 2935, 2860, 1713, 1457, 1358, 1228. HRMS [ESI] calcd for $C_7H_{13}BrONa$ [M+Na]$^+$ 215.0042, found 215.0034.

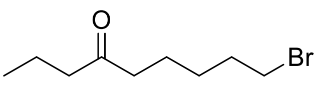

2x: yellow oil. ^{1}H NMR (400 MHz, $CDCl_3$) δ 3.40 (t, *J* = 6.8 Hz, 2H), 2.45-2.34 (m, 4H), 1.90-1.81 (m, 2H), 1.65-1.54 (m, 4H), 1.47-1.38 (m, 2H), 0.91 (t, *J* = 7.2 Hz, 3H); ^{13}C NMR (100 MHz, $CDCl_3$) δ 211.0, 44.8, 42.4, 33.6, 32.6, 27.8, 22.8, 17.3, 13.8. FT-IR: ν (cm^{-1}) 2961, 2873, 1710, 1458, 1373, 1251. HRMS [ESI] calcd for $C_9H_{18}BrO$ [M+H]$^+$ 221.0536, found 221.0523.

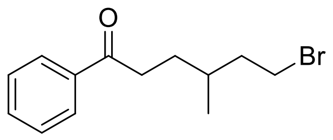

2y: yellow oil. ^{1}H NMR (400 MHz, $CDCl_3$) δ 7.98-7.94 (m, 2H), 7.59-7.54 (m, 1H), 7.50-7.44 (m, 2H), 3.54-3.39 (m, 2H), 3.07-2.92 (m, 2H), 1.98-1.88 (m, 1H), 1.87-1.70 (m, 3H), 1.62-1.56 (m, 1H), 0.97 (d, *J* = 6.4 Hz, 3H); ^{13}C NMR (100 MHz, $CDCl_3$) δ 200.2, 136.9, 133.0, 128.6, 128.0, 39.8, 36.1, 31.8, 31.4, 30.6, 18.8. FT-IR: ν (cm^{-1}) 2960, 2928, 2316, 1683, 1411, 1207. HRMS [ESI] calcd for $C_{13}H_{17}BrONa$ $[M+Na]^+$ 291.0355, found 291.0350.

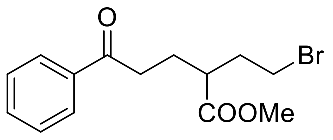

2z: yellow oil. ^{1}H NMR (400 MHz, $CDCl_3$) δ 7.97-7.92 (m, 2H), 7.59-7.54 (m, 1H), 7.49-7.43 (m, 2H), 3.70 (s, 3H), 3.52-3.35 (m, 2H), 3.09-2.94 (m, 2H), 2.79-2.70 (m, 1H), 2.35-2.25 (m, 1H), 2.12-1.96 (m, 3H); ^{13}C NMR (100 MHz, $CDCl_3$) δ 198.9, 175.1, 136.7, 133.2, 128.6, 128.0, 51.9, 43.2, 35.8, 35.1, 30.7, 25.9. FT-IR: ν (cm^{-1}) 3504, 3059, 2851, 1731, 1684, 1245. HRMS [ESI] calcd for $C_{14}H_{17}BrO_3Na$ $[M+Na]^+$ 335.0253, found 335.0265.

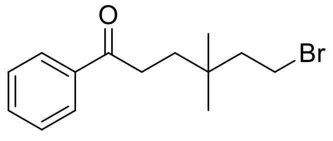

2aa: yellow solid, m.p. 47-48 ^{o}C. ^{1}H NMR (400 MHz, $CDCl_3$) δ 7.95 (d, *J* = 7.6 Hz, 2H), 7.55 (dd, *J* = 7.2, 7.2 Hz, 1H), 7.47 (dd, *J* = 7.6, 7.6 Hz, 2H), 3.44-3.35 (m, 2H), 2.97-2.89 (m, 2H), 1.94-1.86 (m, 2H), 1.70-1.64 (m, 2H), 0.96 (s, 6H); ^{13}C NMR (100 MHz, $CDCl_3$) δ 199.8, 136.5, 132.6, 128.2, 127.6, 45.0, 35.1, 33.6, 32.9, 28.6, 26.2. FT-IR: ν (cm^{-1}) 3065, 2869, 2361, 1975, 1681, 1246. HRMS [ESI] calcd for $C_{14}H_{20}BrO$ $[M+H]^+$ 283.0692, found 283.0691.

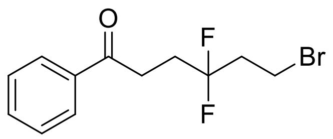

2ab: yellow solid, m.p. 54-56 ^{o}C. ^{1}H NMR (400 MHz, $CDCl_3$) δ 7.98 (d, *J* = 7.2 Hz, 2H), 7.59 (dd, *J* = 7.2, 7.2 Hz, 1H), 7.48 (dd, *J* = 7.6, 7.6 Hz, 2H), 3.50 (t, *J* = 8.0 Hz, 2H), 3.23 (t, *J* = 7.2 Hz, 2H), 2.59-2.43 (m, 2H), 2.43-2.27 (m, 2H); ^{13}C NMR (100 MHz, $CDCl_3$) δ 197.3, 135.9, 132.9, 128.2, 127.6, 122.9 (t, J_{C-F} = 240.6 Hz), 40.2 (t, J_{C-F} = 25.4 Hz), 30.5 (t, J_{C-F} = 3.8 Hz), 30.3 (t, J_{C-F} = 24.6 Hz), 22.8 (t, J_{C-F} = 6.0 Hz); ^{19}F NMR (376 MHz, $CDCl_3$) δ -100.1 (s). FT-IR: ν (cm^{-1}) 2982, 2851, 2361, 1679, 1423, 1215. HRMS [ESI] calcd for $C_{12}H_{13}BrF_2ONa$ $[M+Na]^+$ 313.0010, found 313.0006.

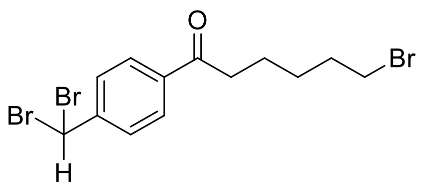

2ac: yellow oil. ^{1}H NMR (400 MHz, $CDCl_3$) δ 7.98-7.93 (m, 2H), 7.78-7.64 (m, 2H), 6.66 (s, 1H), 3.43 (t, *J* = 6.8 Hz, 2H), 2.99 (t, *J* = 7.2 Hz, 2H), 1.96-1.88 (m, 2H), 1.82-1.72 (m, 2H), 1.58-1.49 (m, 2H); ^{13}C NMR (100 MHz, $CDCl_3$) δ 198.4, 145.6, 137.3, 128.0, 126.4, 39.2, 38.0, 33.1, 32.1, 27.3, 22.7. FT-IR: ν (cm^{-1}) 3029, 2869, 1951, 1678, 1465, 1262. HRMS [ESI] calcd for $C_{13}H_{15}Br_3ONa$ $[M+Na]^+$ 446.8565, found 446.8554.

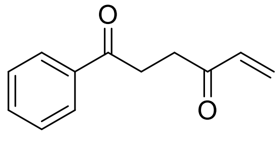

2ad: yellow oil. ^{1}H NMR (400 MHz, $CDCl_3$) δ 8.02-7.96 (m, 2H), 7.59-7.53 (m, 1H), 7.49-7.43 (m, 2H), 6.44 (dd, *J* = 17.6, 10.4 Hz, 1H), 6.32 (dd, *J* = 17.6, 1.2 Hz, 1H), 5.89 (dd, *J* = 10.4, 1.2 Hz, 1H), 3.34 (t, *J* = 6.4 Hz, 2H), 3.07 (t, *J* = 6.4 Hz, 2H); ^{13}C NMR (100 MHz, $CDCl_3$)

δ 199.2, 198.5, 136.7, 136.4, 133.2, 128.6, 128.5, 128.1, 33.3, 32.3. FT-IR: ν (cm^{-1}) 3060, 3026, 2855, 2911, 1678, 1328. HRMS [ESI] calcd for $C_{12}H_{13}O_2$ $[M+H]^+$ 189.0910, found 189.0920.

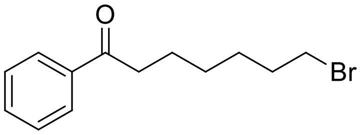

2ae: yellow solid, m.p. 36-38 °C. ^{1}H NMR (400 MHz, $CDCl_3$) δ 7.99-7.93 (m, 2H), 7.59-7.53 (m, 1H), 7.50-7.43 (m, 2H), 3.41 (t, J = 6.8 Hz, 2H), 2.98 (t, J = 7.2 Hz, 2H), 1.93-1.83 (m, 2H), 1.81-1.71 (m, 2H), 1.55-1.37 (m, 4H); ^{13}C NMR (100 MHz, $CDCl_3$) δ 199.8, 136.5, 132.5, 128.1, 127.6, 37.9, 33.4, 32.1, 28.0, 27.5, 23.6. FT-IR: ν (cm-1) 3058, 2959, 2853, 1677, 1426, 1221. HRMS [ESI] calcd for $C_{13}H_{17}BrONa$ $[M+Na]^+$ 291.0355, found 291.0353.

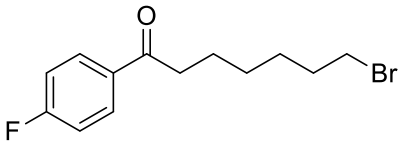

2af: white solid, m.p. 40-42 °C. ^{1}H NMR (400 MHz, $CDCl_3$) δ 8.02-7.94 (m, 2H), 7.17-7.09 (m, 2H), 3.41 (t, J = 6.8 Hz, 2H), 2.95 (t, J = 7.2 Hz, 2H), 1.92-1.83 (m, 2H), 1.80-1.69 (m, 2H), 1.55-1.36 (m, 4H); ^{13}C NMR (100 MHz, $CDCl_3$) δ 198.6, 165.7 (d, J_{C-F} = 252.8 Hz), 133.4 (d, J_{C-F} = 3.1 Hz), 130.6 (d, J_{C-F} = 9.2 Hz), 115.7 (d, J_{C-F} = 21.9 Hz), 38.3, 33.8, 32.6, 28.4, 28.0, 24.0; ^{19}F NMR (376 MHz, $CDCl_3$) δ -105.6 (s). FT-IR: ν (cm^{-1}) 3052, 2936, 2836, 1930, 1676, 1256. HRMS [ESI] calcd for $C_{13}H_{16}BrFONa$ $[M+Na]^+$ 309.0261, found 309.0266.

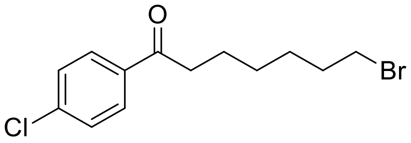

2ag: yellow solid, m.p. 47-48 °C. ^{1}H NMR (400 MHz, $CDCl_3$) δ 7.92-7.86 (m, 2H), 7.46-7.41 (m, 2H), 3.44-3.38 (t, J = 6.8 Hz, 2H), 2.94 (t, J = 7.2 Hz, 2H), 1.92-1.83 (m, 2H), 1.80-1.70 (m, 2H), 1.54-1.36 (m, 4H); ^{13}C NMR (100 MHz, $CDCl_3$) δ 199.0, 139.4, 135.3, 129.5, 128.9, 38.3, 33.8, 32.5, 28.4, 28.0, 23.9. FT-IR: ν (cm^{-1}) 3091, 2932, 2852, 1952, 1675, 1285. HRMS [ESI] calcd for $C_{13}H_{16}BrClONa$ $[M+Na]^+$ 324.9965, found 324.9965.

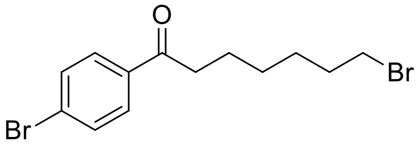

2ah: yellow solid, m.p. 49-50 °C. ^{1}H NMR (400 MHz, $CDCl_3$) δ 7.84-7.79 (m 2H), 7.62-7.57 (m, 2H), 3.41 (t, J = 6.8 Hz, 2H), 2.94 (t, J = 7.2 Hz, 2H), 1.92-1.82 (m, 2H), 1.79-1.69 (m, 2H), 1.53-1.35 (m, 4H); ^{13}C NMR (100 MHz, $CDCl_3$) δ 199.2, 135.7, 131.9, 129.6, 128.1, 38.3, 33.8, 32.5, 28.4, 28.0, 23.9. FT-IR: ν (cm^{-1}) 3087, 2915, 2852, 1935, 1676, 1254. HRMS [ESI] calcd for $C_{13}H_{17}Br_2O$ $[M+H]^+$ 346.9641, found 346.9657.

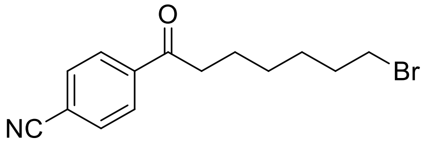

2ai: white solid, m.p. 55-57 °C. ^{1}H NMR (400 MHz, $CDCl_3$) δ 8.06-8.00 (m, 2H), 7.79-7.74 (m, 2H), 3.41 (t, J = 6.8 Hz, 2H), 2.99 (t, J = 7.2 Hz, 2H), 1.92-1.82 (m, 2H), 1.81-1.71 (m, 2H), 1.55-1.36 (m, 4H); ^{13}C NMR (100 MHz, $CDCl_3$) δ 198.2, 139.4, 132.0, 128.0, 117.5, 115.8, 38.2, 33.3, 32.0, 27.8, 27.4, 23.2. FT-IR: ν (cm^{-1}) 2941, 2852, 2361, 2230, 1684, 1249. HRMS [ESI] calcd for $C_{14}H_{16}BrNONa$ $[M+Na]^+$ 316.0307, found 316.0306.

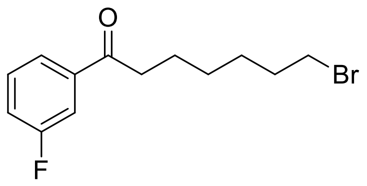

2aj: yellow solid, m.p. 28-29 ºC. ^{1}H NMR (400 MHz, $CDCl_3$) δ 7.77-7.70 (m, 1H), 7.66-7.60 (m, 1H), 7.48-7.40 (m, 1H), 7.29-7.22 (m, 1H), 3.41 (t, *J* = 6.8 Hz, 2H), 2.95 (t, *J* = 7.2 Hz, 2H), 1.93-1.82 (m, 2H), 1.81-1.71 (m, 2H), 1.55-1.36 (m, 4H); ^{13}C NMR (100 MHz, $CDCl_3$) δ 198.9, 162.9 (d, J_{C-F} = 246.3 Hz), 139.1 (d, J_{C-F} = 5.9 Hz), 130.2 (d, J_{C-F} = 7.6 Hz), 123.8 (d, J_{C-F} = 3.0 Hz), 120.0 (d, J_{C-F} = 21.4 Hz), 114.8 (d, J_{C-F} = 22.1 Hz), 38.5, 33.8, 32.5, 28.4, 28.0, 23.9; ^{19}F NMR (376 MHz, $CDCl_3$) δ -111.9 (s). FT-IR: ν (cm^{-1}) 2935, 2857, 2361, 1682, 1442, 1251. HRMS [ESI] calcd for $C_{13}H_{16}BrFONa$ $[M+Na]^+$ 309.0261, found 309.0261.

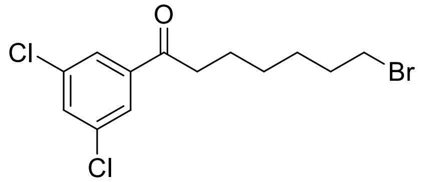

2ak: white solid, m.p. 50-52 ºC. ^{1}H NMR (400 MHz, $CDCl_3$) δ 7.79 (d, *J* = 2.0 Hz, 2H), 7.54 (dd, *J* = 2.0, 2.0 Hz, 1H), 3.42 (t, *J* = 6.8 Hz, 2H), 2.93 (t, *J* = 7.2 Hz, 2H), 1.92-1.84 (m, 2H), 1.79-1.70 (m, 2H), 1.54-1.36 (m, 4H); ^{13}C NMR (100 MHz, $CDCl_3$) δ 197.5, 139.3, 135.6, 132.6, 126.5, 38.5, 33.8, 32.5, 28.3, 27.9, 23.7. FT-IR: ν (cm^{-1}) 3078, 2960, 2853, 2360, 1787, 1337. HRMS [EI] calcd for $C_{13}H_{15}BrCl_2O$ $[M]^+$ 335.9683, found 335.9675.

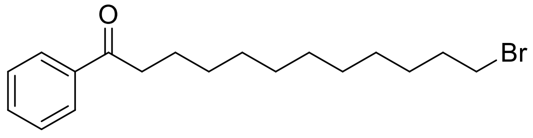

2al: yellow solid, m.p. 32-33 ºC. ^{1}H NMR (400 MHz, $CDCl_3$) δ 8.00-7.91 (m, 2H), 7.58-7.52 (m, 1H), 7.49-7.42 (m, 2H), 3.40 (t, *J* = 6.8 Hz, 2H), 2.96 (t, *J* = 7.2 Hz, 2H), 1.89-1.79 (m, 2H), 1.78-1.68 (m, 2H), 1.48-1.18 (m, 14H); ^{13}C NMR (100 MHz, $CDCl_3$) δ 200.1, 136.6, 132.4, 128.1, 127.6, 38.1, 33.6, 32.4, 29.0, 29.0, 29.0, 28.9, 28.9, 28.3, 27.7, 23.9. FT-IR: ν (cm^{-1}) 2921, 2850, 1686, 1578, 1406, 1264. HRMS [ESI] calcd for $C_{18}H_{27}BrONa$ $[M+Na]^+$ 361.1137, found 361.1146.

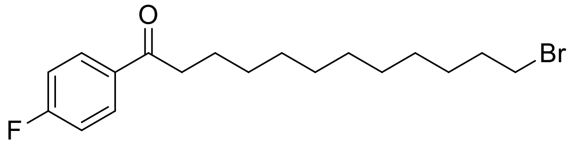

2am: yellow oil. ^{1}H NMR (400 MHz, $CDCl_3$) δ 8.01-7.95 (m, 2H), 7.16-7.08 (m, 2H), 3.40 (t, *J* = 6.8 Hz, 2H), 2.93 (t, *J* = 7.2 Hz, 2H), 1.89-1.79 (m, 2H), 1.76-1.67 (m, 2H), 1.46-1.23 (m, 14H); ^{13}C NMR (100 MHz, $CDCl_3$) δ 198.5, 165.1 (d, J_{C-F} = 252.8 Hz), 133.0 (d, J_{C-F} = 3.0 Hz), 130.2 (d, J_{C-F} = 9.2 Hz), 115.1 (d, J_{C-F} = 21.7 Hz), 38.1, 33.6, 32.3, 29.0, 29.0, 28.9, 28.9, 28.8, 28.3, 27.7, 23.8; ^{19}F NMR (376 MHz, $CDCl_3$) δ -105.8 (s). FT-IR: ν (cm^{-1}) 2925, 2853, 1686, 1506, 1409, 1263. HRMS [ESI] calcd for $C_{18}H_{26}BrFONa$ $[M+Na]^+$ 379.1043, found 379.1051.

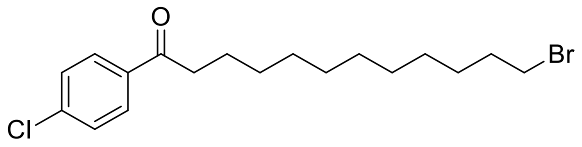

2an: white solid, m.p. 45-46 ºC. ^{1}H NMR (400 MHz, $CDCl_3$) δ 7.92-7.86 (m, 2H), 7.45-7.40 (m, 2H), 3.40 (t, *J* = 6.8 Hz, 2H), 2.92 (t, *J* = 7.2 Hz, 2H), 1.89-1.79 (m, 2H), 1.77-1.67 (m, 2H), 1.46-1.22 (m, 14H); ^{13}C NMR (100 MHz, $CDCl_3$) δ 199.3, 139.3, 135.4, 129.5, 128.9, 38.6, 34.1, 32.8, 29.5, 29.4, 29.4, 29.4, 29.3, 28.7, 28.2, 24.3. FT-IR: ν (cm^{-1}) 3002, 2849, 2362, 1804, 1687, 1275. HRMS [ESI]

calcd for $C_{18}H_{27}BrClO$ [M+H]$^+$ 373.0928, found 373.0929.

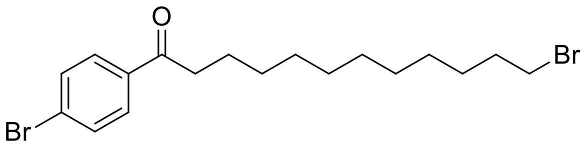

2ao: yellow solid, m.p. 57-58 °C. ^{1}H NMR (400 MHz, $CDCl_3$) δ 7.84-7.79 (m, 2H), 7.62-7.57 (m, 2H), 3.40 (t, *J* = 6.8 Hz, 2H), 2.92 (t, *J* = 7.2 Hz, 2H), 1.89-1.80 (m, 2H), 1.76-1.67 (m, 2H), 1.47-1.23 (m, 14H); ^{13}C NMR (100 MHz, $CDCl_3$) δ 199.5, 135.8, 131.9, 129.6, 128.0, 38.6, 34.1, 32.8, 29.5, 29.4, 29.4, 29.4, 29.3, 28.7, 28.2, 24.3. FT-IR: ν (cm^{-1}) 2961, 2850, 2360, 1678, 1466, 1217. HRMS [ESI] calcd for $C_{18}H_{27}Br_2O$ [M+H]$^+$ 417.0423, found 417.0414.

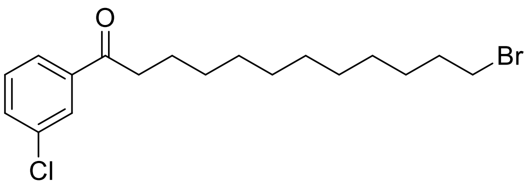

2ap: white solid, m.p. 51-53 °C. ^{1}H NMR (400 MHz, $CDCl_3$) δ 7.92 (dd, *J* = 1.6, 1.6 Hz, 1H), 7.85-7.80 (m, 1H), 7.52 (ddd, *J* = 8.0, 2.0, 0.8 Hz, 1H), 7.40 (dd, *J* = 8.0, 8.0 Hz, 1H), 3.40 (t, *J* = 6.8 Hz, 2H), 2.93 (t, *J* = 7.2 Hz, 2H), 1.90-1.80 (m, 2H), 1.78-1.67 (m, 2H), 1.47-1.21 (m, 14H); ^{13}C NMR (100 MHz, $CDCl_3$) δ 199.2, 138.6, 134.9, 132.8, 129.9, 128.2, 126.1, 38.7, 34.1, 32.8, 29.5, 29.4 (three carbons overlap), 29.3, 28.8, 28.2, 24.2. FT-IR: ν (cm^{-1}) 2919, 2850, 2361, 1679, 1570, 1243. HRMS [ESI] calcd for $C_{18}H_{26}BrClONa$ [M+Na]$^+$ 395.0748, found 395.0742.

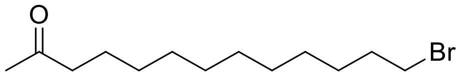

2aq: yellow oil. ^{1}H NMR (400 MHz, $CDCl_3$) δ 3.40 (t, *J* = 6.8 Hz, 2H), 2.41 (t, *J* = 7.2 Hz, 2H), 2.13 (s, 3H), 1.90-1.80 (m, 2H), 1.62-1.52 (m, 3H), 1.46-1.36 (m, 3H), 1.31-1.23 (m, 10H); ^{13}C NMR (100 MHz, $CDCl_3$) δ 208.9, 43.3, 33.6, 32.3, 29.4, 29.2, 29.0, 29.0, 28.9, 28.7, 28.3, 27.7, 23.4. FT-IR: ν (cm^{-1}) 2924, 2853, 1716, 1494, 1359, 1226. HRMS [ESI] calcd for $C_{13}H_{25}BrONa$ [M+Na]$^+$ 299.0981, found 299.0973.

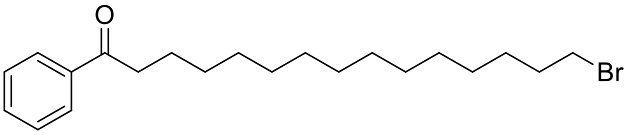

2ar: white solid, m.p. 67-68 °C. ^{1}H NMR (400 MHz, $CDCl_3$) δ 7.98-7.94 (m, 2H), 7.58-7.52 (m, 1H), 7.49-7.43 (m, 2H), 3.41 (t, *J* = 6.8 Hz, 2H), 2.96 (t, *J* = 7.2 Hz, 2H), 1.90-1.81 (m, 2H), 1.78-1.69 (m, 2H), 1.46-1.22 (m, 20H); ^{13}C NMR (100 MHz, $CDCl_3$) δ 200.1, 136.6, 132.4, 128.1, 127.6, 38.2, 33.6, 32.4, 29.1, 29.1, 29.0, 29.0, 29.0, 29.0, 28.9, 28.3, 27.7, 23.9. FT-IR: ν (cm^{-1}) 2915, 2848, 1677, 1445, 1337, 1211. HRMS [ESI] calcd for $C_{21}H_{34}BrO$ [M+H]$^+$ 381.1788, found 381.1779.

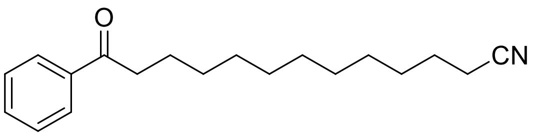

9: white solid, m.p. 40-42 °C. ^{1}H NMR (400 MHz, $CDCl_3$) δ 7.98-7.93 (m, 2H), 7.58-7.52 (m, 1H), 7.49-7.42 (m, 2H), 2.96 (t, *J* = 7.2 Hz, 2H), 2.33 (t, *J* = 7.2 Hz, 2H), 1.78-1.68 (m, 2H), 1.68-1.58 (m, 2H), 1.48-1.24 (m, 14H); ^{13}C NMR (100 MHz, $CDCl_3$) δ 200.6, 137.1, 132.9, 128.5, 128.0, 119.9, 38.6, 29.4, 29.4, 29.4, 29.3, 29.3, 28.7, 28.7, 25.4, 24.3, 17.1. FT-IR: ν (cm^{-1}) 2935, 2852, 2342, 1686, 1449, 1248. HRMS [ESI] calcd for $C_{19}H_{27}NONa$ [M+Na]$^+$ 308.1985, found 308.1977.

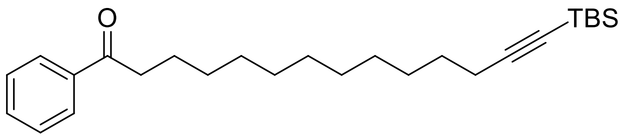

10: yellow oil. ^{1}H NMR (400 MHz, $CDCl_3$) δ 7.98-7.94 (m, 2H), 7.58-7.52 (m, 1H), 7.49-7.43 (m, 2H), 2.96 (t, *J* = 7.2 Hz, 2H), 2.22 (t, *J* = 7.2 Hz, 2H), 1.77-1.69 (m, 2H), 1.55-1.46 (m, 2H), 1.43-1.24 (m, 14H), 0.93 (s, 9H), 0.08 (s, 6H); ^{13}C NMR (100 MHz, $CDCl_3$) δ 200.6, 137.1, 132.8, 128.5, 128.0, 108.3, 82.3, 38.6, 29.5, 29.5, 29.4, 29.1, 28.7, 28.7, 26.1, 24.4, 19.8, 16.5, -4.4. FT-IR: ν (cm^{-1}) 2926, 2854, 2361, 1687, 1361, 1248. HRMS [ESI] calcd for $C_{26}H_{42}OSiNa$ $[M+Na]^+$ 421.2897, found 421.2889.

5. Transformation of products

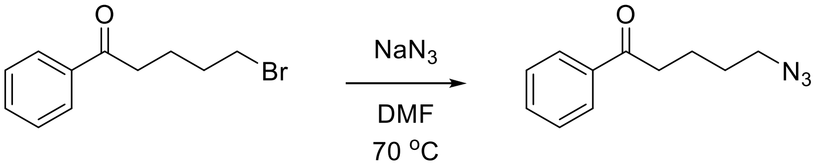

2a **3**

Compound **2a** (48.2 mg, 0.2 mmol, 1.0 equiv.) and NaN_3 (64.0 mg, 0.4 mmol, 2.0 equiv.) were suspended in DMF (2.0 mL) and the reaction mixture was stirred at 70 °C for 2 h. After completion of the reaction, the reaction mixture was diluted with Et_2O and washed with water. The organic layer was separated and dried over anhydrous Na_2SO_4. The solvent was removed under reduced pressure and the residue was purified by flash column chromatography on silica gel (eluent: ethyl acetate/ petroleum ether) to afford the desired product **3** (35.4 mg, 87%).

3: yellow oil. ^{1}H NMR (400 MHz, $CDCl_3$) δ 7.99-7.93 (m, 2H), 7.59-7.54 (m, 1H), 7.49-7.43 (m, 2H), 3.33 (t, *J* = 6.8 Hz, 2H), 3.02 (t, *J* = 7.2 Hz, 2H), 1.88-1.79 (m, 2H), 1.74-1.64 (m, 2H); ^{13}C NMR (100 MHz, $CDCl_3$) δ 199.6, 136.8, 133.1, 128.6, 128.0, 51.3, 37.8, 28.5, 21.3. FT-IR: ν (cm^{-1}) 3087, 2939, 2091, 1683, 1580, 1254. HRMS [ESI] calcd for $C_{11}H_{13}N_3ONa$ $[M+Na]^+$ 226.0951, found 226.0944.

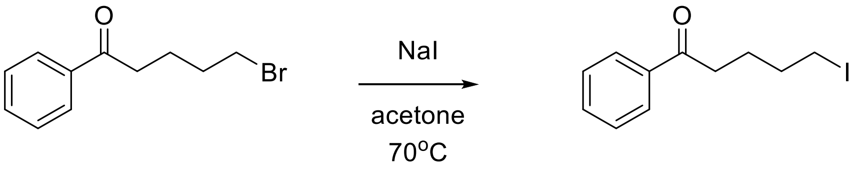

2a **4**

Compound **2a** (48.2 mg, 0.2 mmol, 1.0 equiv.) and NaI (149.9 mg, 1 mmol, 5.0 equiv.) were suspended in acetone (2.0 mL) and the reaction mixture was stirred at 70 °C for 13 h. After completion of the reaction, the solvent was removed under reduced pressure and the residue was purified by flash column chromatography on silica gel (eluent: ethyl acetate/ petroleum ether) to afford the desired product **4** (49.3 mg, 86%).

4: white solid, m.p. 69-70 °C. ^{1}H NMR (400 MHz, $CDCl_3$) δ 7.99-7.92 (m, 2H), 7.60-7.53 (m, 1H),

7.50-7.43 (m, 2H), 3.23 (t, *J* = 6.8 Hz, 2H), 3.00 (t, *J* = 7.2 Hz, 2H), 1.97-1.81 (m, 4H); ^{13}C NMR (100 MHz, $CDCl_3$) δ 199.1, 136.3, 132.6, 128.2, 127.5, 36.8, 32.5, 24.6, 5.8. FT-IR: ν (cm^{-1}) 3056, 2927, 2851, 1673, 1403, 1209. HRMS [ESI] calcd for $C_{11}H_{13}IONa$ $[M+Na]^+$ 310.9903, found 310.9911.

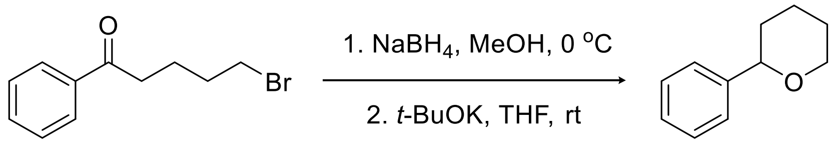

2a **5**

Compound **2a** (48.2 mg, 0.2 mmol, 1.0 equiv.) was dissolved in MeOH (2.0 mL), then sodium borohydride (15.1 mg, 0.4 mmol, 2.0 equiv.) was added in one portion at 0 °C. The reaction mixture was stirred for 20 min. After completion, the reaction mixture was quenched with saturated aqueous ammonium chloride (5.0 mL) and the aqueous layer was extracted by DCM (3 x 5.0 mL). The combined organic layer was dried over anhydrous Na_2SO_4. The solvent was removed under reduced pressure and the residue was purified by flash column chromatography on silica gel (eluent: ethyl acetate/ petroleum ether) to afford the corresponding alcohol product (48.6 mg, quantitative yield). The alcohol (48.6 mg, 0.2 mmol, 1.0 equiv.) and potassium *tert*-butoxide (45.0 mg, 0.4 mmol, 2.0 equiv.) were suspended in THF (2.0 mL), and the reaction mixture was stirred at rt under N_2 for 11 h. The solvent was removed under reduced pressure and the residue was purified by flash column chromatography on silica gel (eluent: ethyl acetate/ petroleum ether) to afford the desired product **5** (26.4 mg, 82%).

5: yellow oil. ^{1}H NMR (400 MHz, $CDCl_3$) δ 7.37-7.22 (m, 5H), 4.35-4.29 (m, 1H), 4.17-4.11 (m, 1H), 3.66-3.58 (m, 1H), 2.01-1.90 (m, 1H), 1.88-1.79 (m, 1H), 1.76-1.58 (m, 4H); ^{13}C NMR (100 MHz, $CDCl_3$) δ 142.8, 127.8, 126.8, 125.4, 79.7, 68.5, 33.5, 25.4, 23.5. FT-IR: ν (cm^{-1}) 3062, 2935, 2731, 1605, 1439, 1204. HRMS [ESI] calcd for $C_{11}H_{15}O$ $[M+H]^+$ 163.1117, found 163.1122.

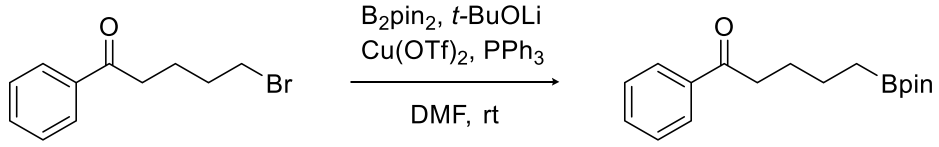

2a **6**

Compound **2a** (48.2 mg, 0.2 mmol, 1.0 equiv.), $Cu(OTf)_2$ (7.2 mg, 0.01 mmol, 0.1 equiv.), PPh_3 (6.8 mg, 0.026 mmol, 0.13 equiv.), *t*-BuOLi (32.0mg, 0.4 mmol, 2.0 equiv), and B_2pin_2 (75.0 mg, 0.3 mmol, 1.5 equiv.) were suspended in DMF (1.0 mL) and the reaction mixture was stirred at rt under N_2 for 10 h. After completion of the reaction, the reaction mixture was diluted with Et_2O and washed with water. The organic layer was separated and dried over anhydrous Na_2SO_4. The solvent was removed under reduced pressure and the residue was purified by flash column chromatography on silica gel (eluent: ethyl acetate/ petroleum ether) to afford the desired product **6** (20.0 mg, 35%).

6: yellow oil. ^{1}H NMR (400 MHz, $CDCl_3$) δ 7.97-7.92 (m, 2H), 7.57-7.51 (m, 1H), 7.47-7.42 (m,

2H), 2.96 (t, J = 7.2 Hz, 2H), 1.79-1.70 (m, 2H), 1.56-1.47 (m, 2H), 1.23 (s, 12H), 0.84 (t, J = 8.0 Hz, 2H); ^{13}C NMR (100 MHz, $CDCl_3$) δ 200.1, 136.6, 132.3, 128.0, 127.6, 82.5, 38.0, 26.5, 24.3, 23.4; ^{11}B NMR (128 MHz, $CDCl_3$) δ 34.1 (s). FT-IR: ν (cm^{-1}) 3061, 2977, 2866, 1685, 1408, 1214. HRMS [ESI] calcd for $C_{17}H_{25}BO_3Na$ $[M+Na]^+$ 311.1789, found 311.1799.

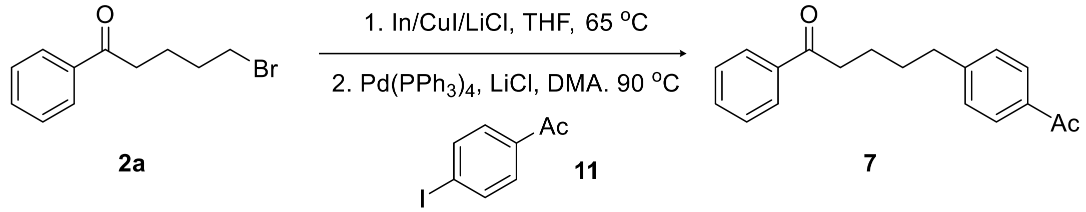

Compound **2a** (120.5 mg, 0.5 mmol, 1.7 equiv.), indium (57.1 mg, 0.5 mmol, 1.7 equiv.), CuI (95.2 mg, 0.5 mmol, 1.7 equiv), LiCl (21.2 mg, 0.5 mmol, 1.7 equiv.), and THF (1.5 mL) were loaded in a 5 mL reaction vial. The reaction was stirred vigorously at 65 °C for 15 h. After completion of the reaction, it was kept still for 10 min. Then the upper clear solution was separated from the bottom black precipitate by syringe. The residual black precipitate was washed with 3 mL THF and the THF layer was separated by syringe. The combined organic layers were concentrated to give the residue which was then dissolved in 1.5 mL DMA and transferred to another 5 mL reaction vial. Aryl halide **11** (73.8 mg, 0.3 mmol, 1.0 equiv.), LiCl (21.2 mg, 0.5 mmol, 1.7 equiv.), and $Pd(PPh_3)_4$ (28.9 mg, 0.025 mmol, 0.083 equiv.) was added to the vial sequentially. The reaction mixture was stirred at 90 °C for 27 h. After completion of the reaction, the reaction mixture was diluted with Et_2O and washed with water. The organic layer was separated and dried over anhydrous Na_2SO_4. The solvent was removed under reduced pressure and the residue was purified by flash column chromatography on silica gel (eluent: ethyl acetate/ petroleum ether) to afford the desired product **7** (53.8 mg, 64%).

7: yellow solid, m.p. 61-63 °C. ^{1}H NMR (400 MHz, $CDCl_3$) δ 7.97-7.91 (m, 2H), 7.90-7.85 (m, 2H), 7.58-7.52 (m, 1H), 7.48-7.42 (m, 2H), 7.30-7.25 (m, 2H), 2.99 (t, J = 6.8 Hz, 2H), 2.73 (t, J = 7.6 Hz, 2H), 2.57 (s, 3H), 1.84-1.68 (m, 4H); ^{13}C NMR (100 MHz, $CDCl_3$) δ 200.0, 197.8, 148.1, 137.0, 135.0, 133.0, 128.6, 128.6, 128.5, 128.0, 38.3, 35.8, 30.7, 26.6, 23.8. FT-IR: ν (cm^{-1}) 2930, 2860, 2360, 1735, 1522, 1268. HRMS [ESI] calcd for $C_{19}H_{20}O_2Na$ $[M+Na]^+$ 303.1356, found 303.1346.

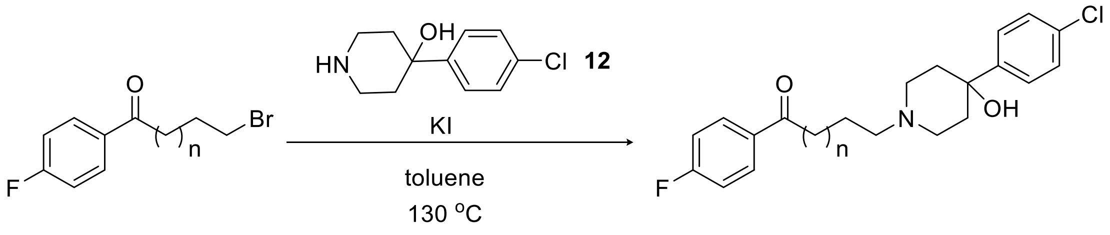

The alkyl bromide (0.2 mmol, 1.0 equiv.), compound **12** (84.7 mg, 0.4 mmol, 2.0 equiv.), and KI (1.0 mg, 0.006 mmol, 0.03 equiv.) were dissolved in anhydrous toluene (2.0 mL). The reaction mixture was stirred at 130 °C for 60 h. After completion of the reaction, the solvent was removed under reduced pressure and the residue was purified by flash column chromatography on silica gel

(eluent: ethyl acetate/ petroleum ether) to afford the desired product **8a** (n = 2, 63.8 mg, 82%), **8b** (n = 3, 64.8 mg, 81%), **8c** (n = 4, 70.9 mg, 85%), and **8d** (n = 9, 74.0 mg, 76%) respectively.

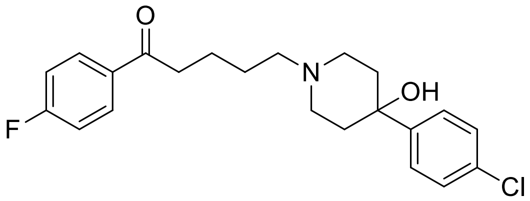

8a: yellow solid, m.p. 70-72 °C. ^{1}H NMR (400 MHz, $CDCl_3$) δ 8.01-7.95 (m, 2H), 7.44-7.39 (m, 2H), 7.32-7.27 (m, 2H), 7.16-7.09 (m, 2H), 2.97 (t, *J* = 7.2 Hz, 2H), 2.85-2.77 (m, 2H), 2.48-2.37 (m, 4H), 2.14-2.04 (m, 2H), 1.81-1.68 (m, 4H), 1.66-1.56 (m, 2H); ^{13}C NMR (100 MHz, $CDCl_3$) δ 198.5, 165.7 (d, J_{C-F} = 252.9 Hz), 146.9, 133.4 (d, J_{C-F} = 3.0 Hz), 132.7, 130.7 (d, J_{C-F} = 9.2 Hz), 128.4, 126.1, 115.7 (d, J_{C-F} = 21.7 Hz), 71.1, 58.4, 49.4, 38.4, 38.3, 26.6, 22.3; ^{19}F NMR (376 MHz, $CDCl_3$) δ -105.5 (s). FT-IR: ν (cm^{-1}) 3358, 3192, 2953, 2772, 1690, 1225. HRMS [ESI] calcd for $C_{22}H_{26}ClFNO_2$ $[M+H]^+$ 390.1631, found 390.1645.

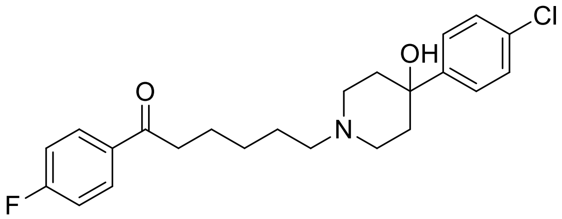

8b: yellow solid, m.p. 111-113 °C. ^{1}H NMR (400 MHz, $CDCl_3$) δ 8.01-7.95 (m, 2H), 7.47-7.41 (m, 2H), 7.33-7.28 (m, 2H), 7.16-7.09 (m, 2H), 2.95 (t, *J* = 7.2 Hz, 2H), 2.86-2.79 (m, 2H), 2.46-2.37 (m, 4H), 2.17-2.08 (m, 2H), 1.81-1.69 (m, 4H), 1.64-1.54 (m, 2H), 1.46-1.36 (m, 2H); ^{13}C NMR (100 MHz, $CDCl_3$) δ 198.3, 165.2 (d, J_{C-F} = 252.9 Hz), 146.5, 132.9 (d, J_{C-F} = 3.0 Hz), 132.2, 130.2 (d, J_{C-F} = 9.2 Hz), 127.9, 125.7, 115.2 (d, J_{C-F} = 21.6 Hz), 70.5, 58.2, 49.0, 37.9, 37.9, 26.9, 26.4, 23.7; ^{19}F NMR (376 MHz, $CDCl_3$) δ -105.5 (s). FT-IR: ν (cm^{-1}) 3177, 2951, 2772, 1684, 1487, 1262. HRMS [ESI] calcd for $C_{23}H_{28}ClFNO_2$ $[M+H]^+$ 404.1787, found 404.1799.

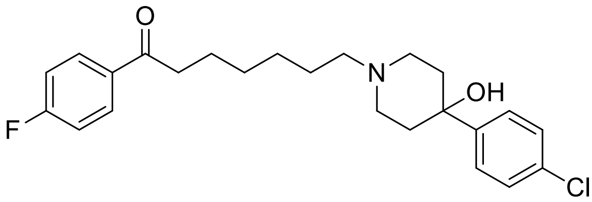

8c: yellow solid, m.p. 98-100 °C. ^{1}H NMR (400 MHz, $CDCl_3$) δ 8.01-7.94 (m, 2H), 7.46-7.41 (m, 2H), 7.33-7.28 (m, 2H), 7.16-7.08 (m, 2H), 2.93 (t, *J* = 7.2 Hz, 2H), 2.85-2.78 (m, 2H), 2.46-2.36 (m, 4H), 2.17-2.07 (m, 2H), 1.79-1.68 (m, 4H), 1.59-1.50 (m, 2H), 1.45-1.33 (m, 4H); ^{13}C NMR (100 MHz, $CDCl_3$) δ 198.8, 165.6 (d, J_{C-F} = 252.8 Hz), 147.0, 133.4 (d, J_{C-F} = 3.0 Hz), 132.7, 130.6 (d, J_{C-F} = 9.2 Hz), 128.4, 126.1, 115.6 (d, J_{C-F} = 21.7 Hz), 71.1, 58.8, 49.5, 38.5, 38.4, 29.3, 27.6, 26.9, 24.2; ^{19}F NMR (376 MHz, $CDCl_3$) δ -105.6 (s). FT-IR: ν (cm^{-1}) 2957, 2830, 2361, 1679, 1471, 1274. HRMS [ESI] calcd for $C_{24}H_{30}ClFNO_2$ $[M+H]^+$ 418.1944, found 418.1954.

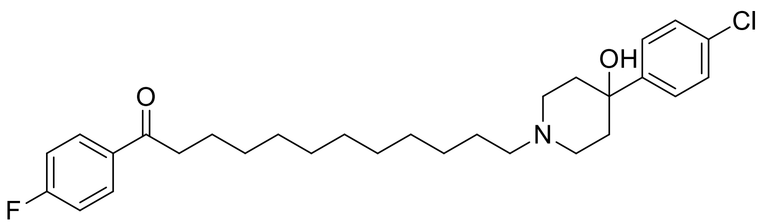

8d: yellow solid, m.p. 88-90 °C. ^{1}H NMR (400 MHz, $CDCl_3$) δ 8.01-7.94 (m 2H), 7.46-7.41 (m, 2H), 7.34-7.27 (m, 2H), 7.15-7.08 (m, 2H), 2.92 (t,

J = 7.2 Hz, 2H), 2.85-2.77 (m, 2H), 2.44-2.34 (m, 4H), 2.16-2.07 (m, 2H), 1.76-1.66 (m, 4H), 1.56-1.46 (m, 2H), 1.40-1.22 (m, 14H); ^{13}C NMR (100 MHz, $CDCl_3$) δ 199.0, 165.6 (d, J_{C-F} = 252.9 Hz), 147.1, 133.4 (d, J_{C-F} = 3.0 Hz), 132.6, 130.7 (d, J_{C-F} = 9.2 Hz), 128.3, 126.1, 115.6 (d, J_{C-F} = 21.7 Hz), 71.0, 59.0, 49.5, 38.5, 38.4, 29.6, 29.6, 29.5, 29.3, 27.7, 27.0, 24.3; ^{19}F NMR (376 MHz, $CDCl_3$) δ -105.7 (s). FT-IR: ν (cm^{-1}) 3132, 2959, 2774, 1685, 1405, 1209. HRMS [ESI] calcd for $C_{29}H_{40}ClFNO_2$ $[M+H]^+$ 488.2726, found 488.2734.

6. ^{1}H, ^{13}C, and ^{19}F NMR spectra

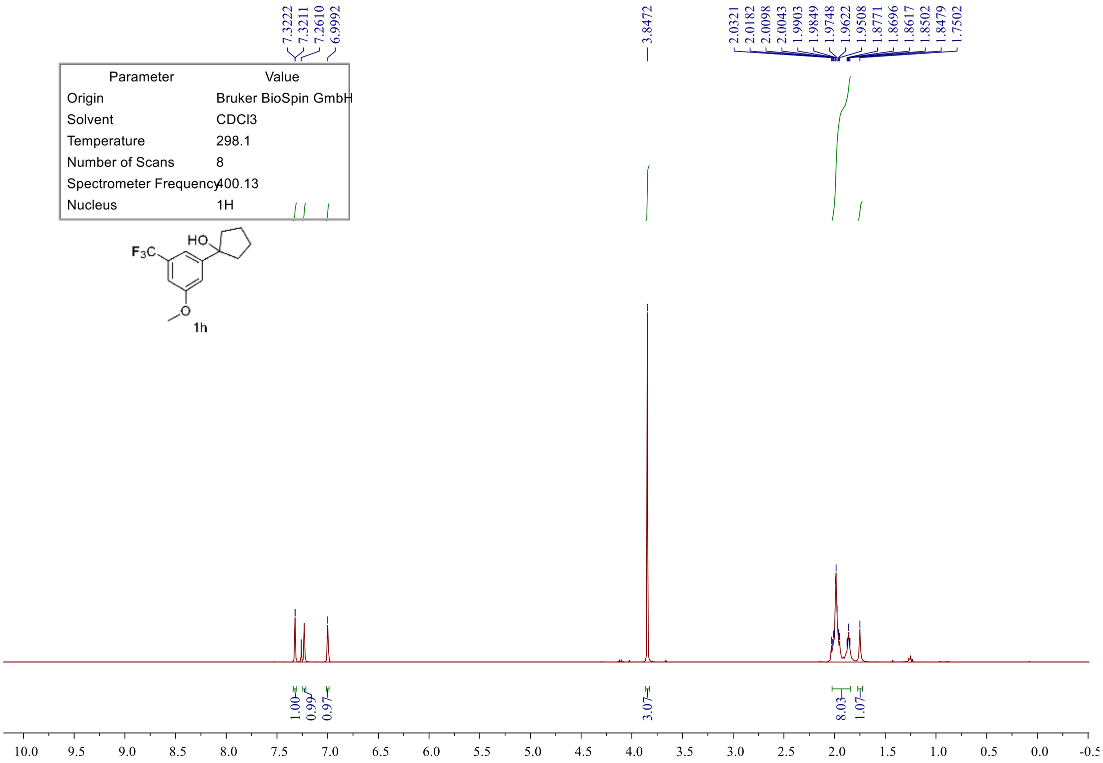

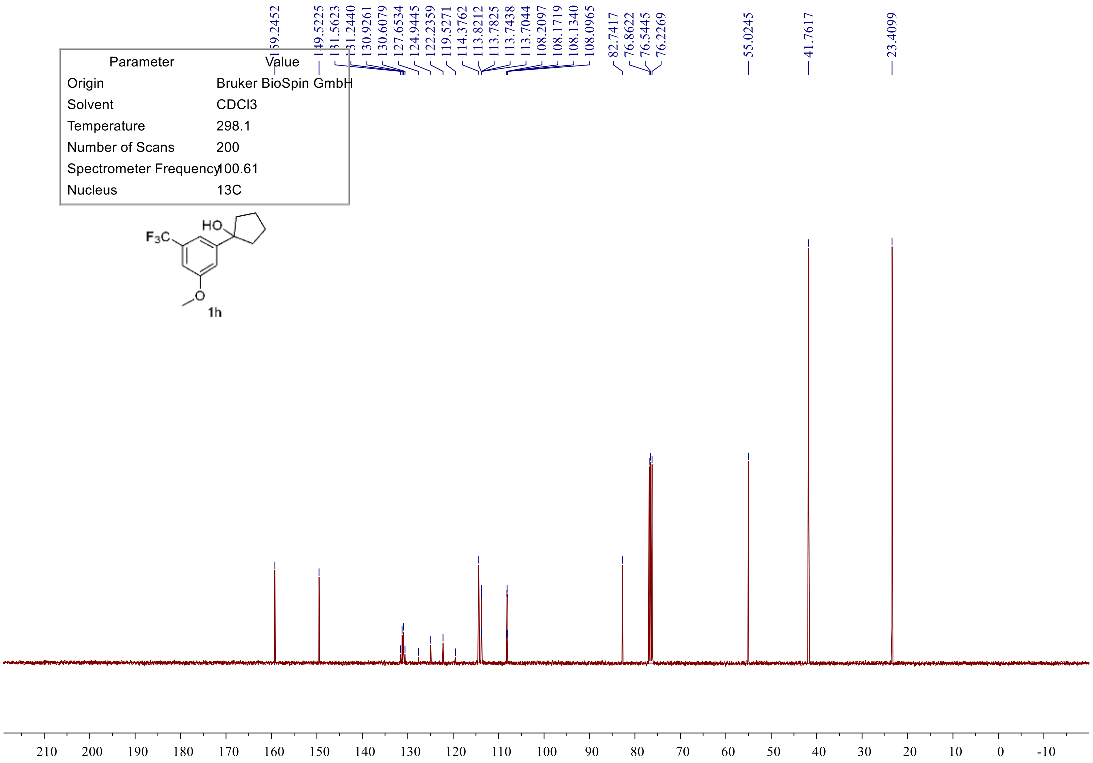
149.5225
131.5623
130.9261
130.6079
127.6534
124.9445
122.2359
119.5271
114.3762
113.8212
113.7825
113.7438
113.7044
108.2097
108.1719
108.1340
108.0965
82.7417
76.8622
76.5445
76.2269
55.0245
41.7617
23.4099
Parameter
Value
Origin
Bruker BioSpin GmbH
Solvent
CDCl3
Temperature
298.1
Number of Scans
200
Spectrometer Frequency
100.61
Nucleus
13C
HO
F_3C
O
1h
210 200 190 180 170 160 150 140 130 120 110 100 90 80 70 60 50 40 30 20 10 0 -10

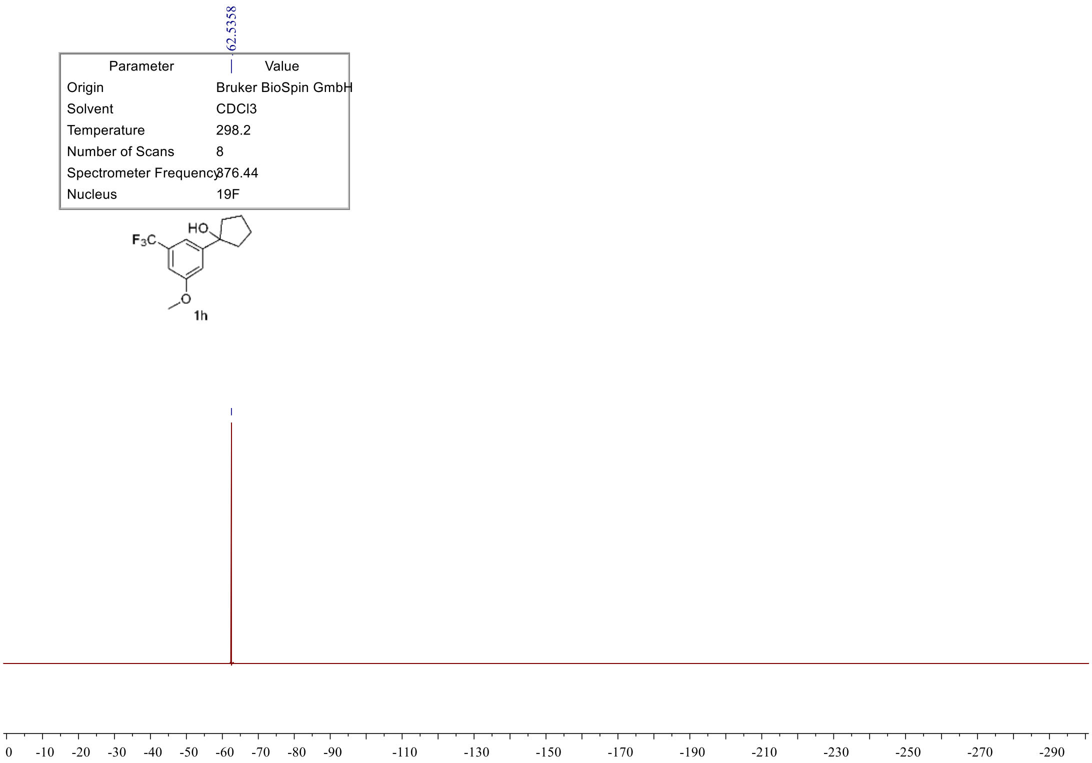
62.5358
Parameter
Value
Origin
Bruker BioSpin GmbH
Solvent
CDCl3
Temperature
298.2
Number of Scans
8
Spectrometer Frequency
376.44
Nucleus
19F
HO
F_3C
O
1h
0 -10 -20 -30 -40 -50 -60 -70 -80 -90 -110 -130 -150 -170 -190 -210 -230 -250 -270 -290

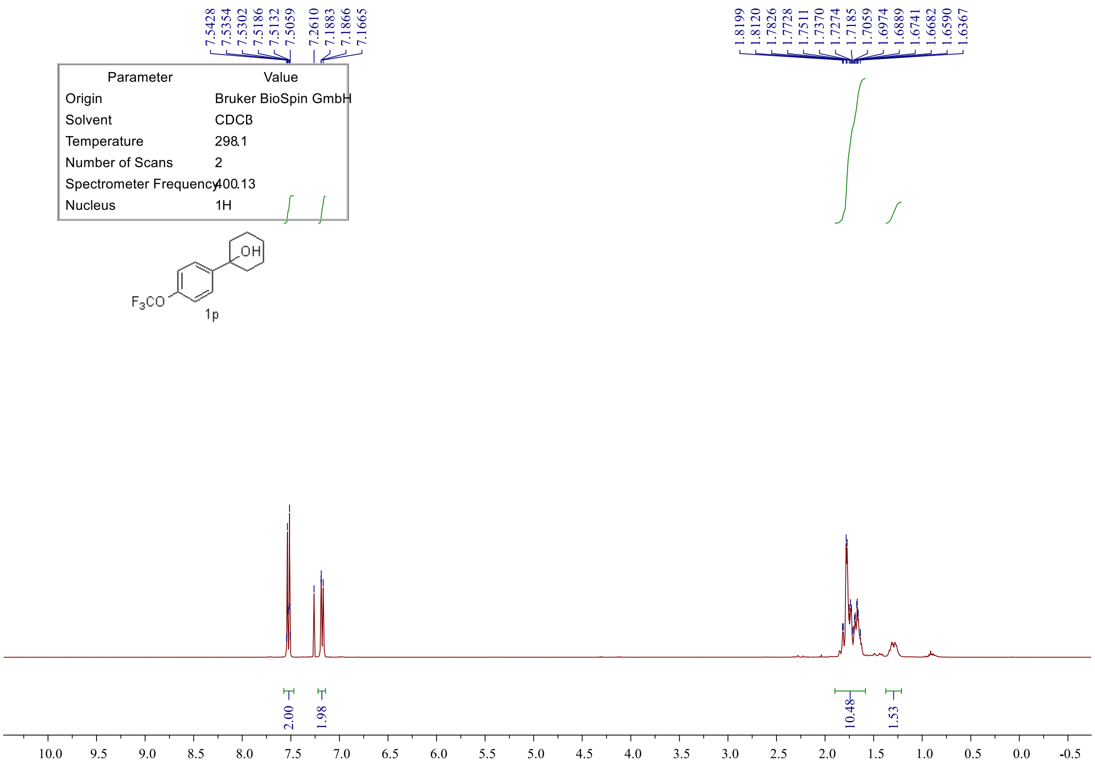
7.5428
7.5354
7.5302
7.5186
7.5132
7.5059
7.2610
7.1883
7.1866
7.1665
1.8199
1.8120
1.7826
1.7728
1.7511
1.7370
1.7274
1.7185
1.7059
1.6974
1.6889
1.6741
1.6682
1.6590
1.6367
Parameter Value
Origin Bruker BioSpin GmbH
Solvent CDCl3
Temperature 298.1
Number of Scans 2
Spectrometer Frequency 400.13
Nucleus 1H
OH
F3CO
1p
2.00
1.98
10.48
1.53
10.0 9.5 9.0 8.5 8.0 7.5 7.0 6.5 6.0 5.5 5.0 4.5 4.0 3.5 3.0 2.5 2.0 1.5 1.0 0.5 0.0 -0.5

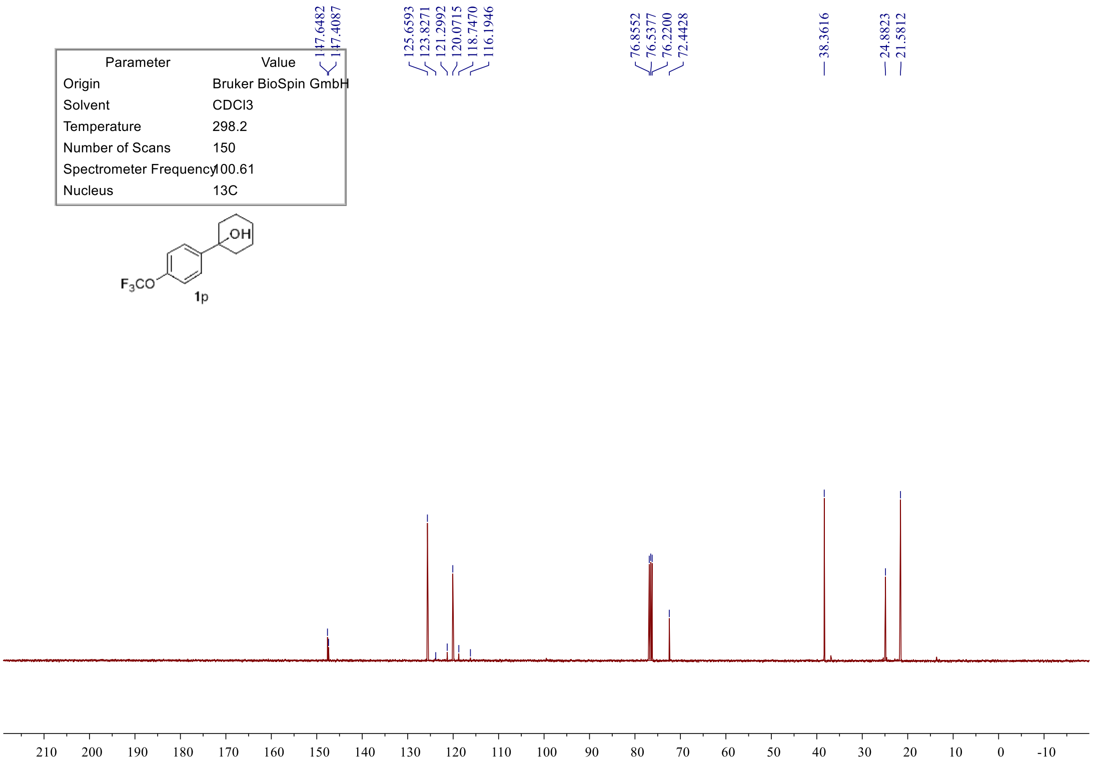
147.6482
147.4087
125.6593
123.8271
121.2992
120.0715
118.7470
116.1946
76.8552
76.5377
76.2200
72.4428
38.3616
24.8823
21.5812
Parameter Value
Origin Bruker BioSpin GmbH
Solvent CDCl3
Temperature 298.2
Number of Scans 150
Spectrometer Frequency 100.61
Nucleus 13C
OH
F3CO
1p
210 200 190 180 170 160 150 140 130 120 110 100 90 80 70 60 50 40 30 20 10 0 -10

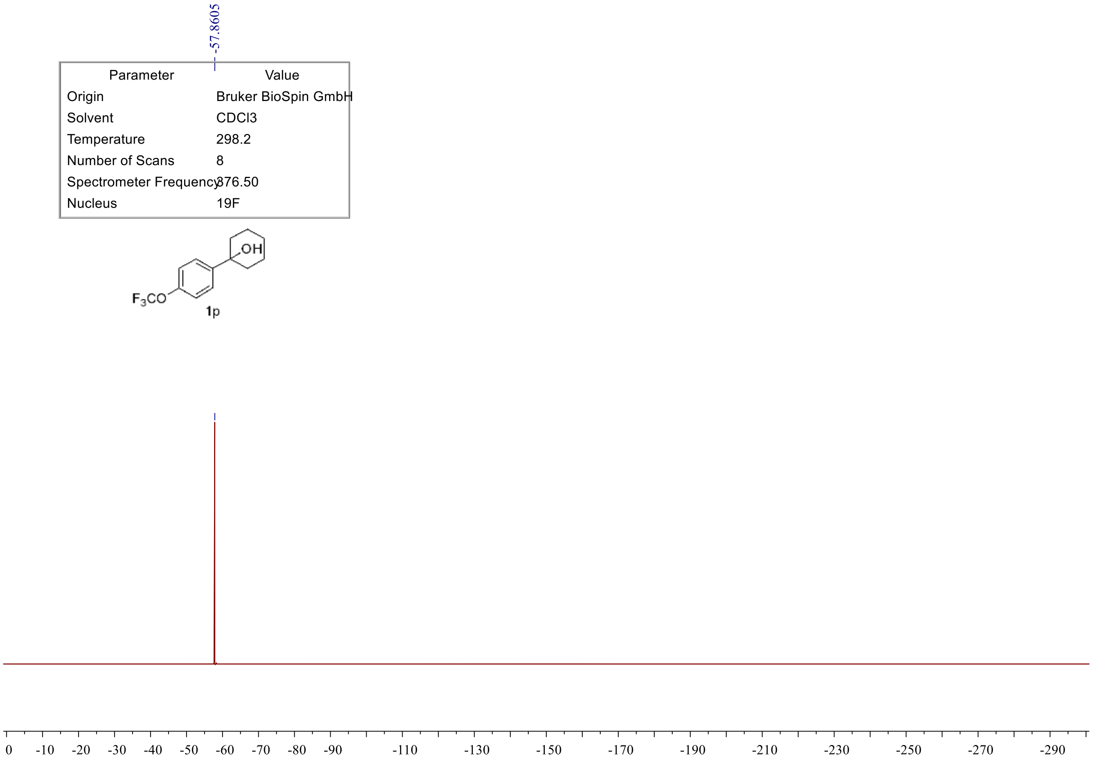
-57.8605
Parameter Value
Origin Bruker BioSpin GmbH
Solvent CDCl3
Temperature 298.2
Number of Scans 8
Spectrometer Frequency 876.50
Nucleus 19F
OH
F3CO
1p
0 -10 -20 -30 -40 -50 -60 -70 -80 -90 -110 -130 -150 -170 -190 -210 -230 -250 -270 -290

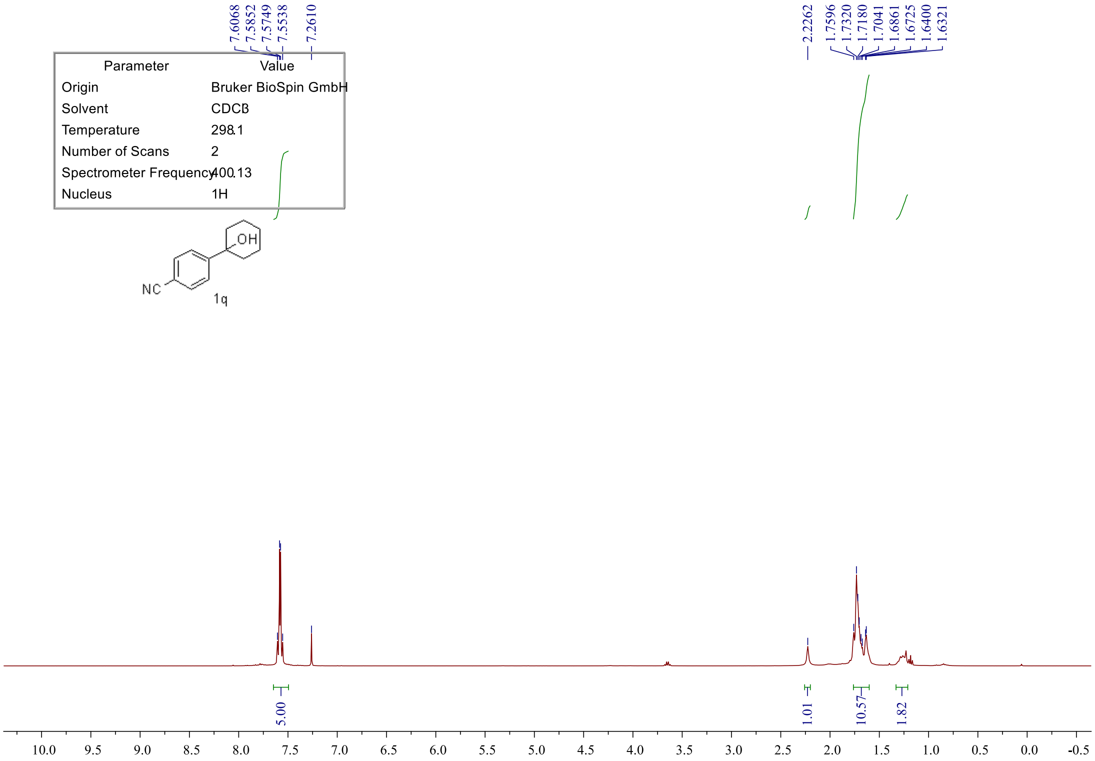
7.6068
7.5852
7.5749
7.5538
7.2610
2.2262
1.7596
1.7320
1.7180
1.7041
1.6861
1.6725
1.6400
1.6321
Parameter Value
Origin Bruker BioSpin GmbH
Solvent CDCl3
Temperature 298.1
Number of Scans 2
Spectrometer Frequency 400.13
Nucleus 1H
OH
NC
1q
5.00
1.01
10.57
1.82
10.0 9.5 9.0 8.5 8.0 7.5 7.0 6.5 6.0 5.5 5.0 4.5 4.0 3.5 3.0 2.5 2.0 1.5 1.0 0.5 0.0 -0.5

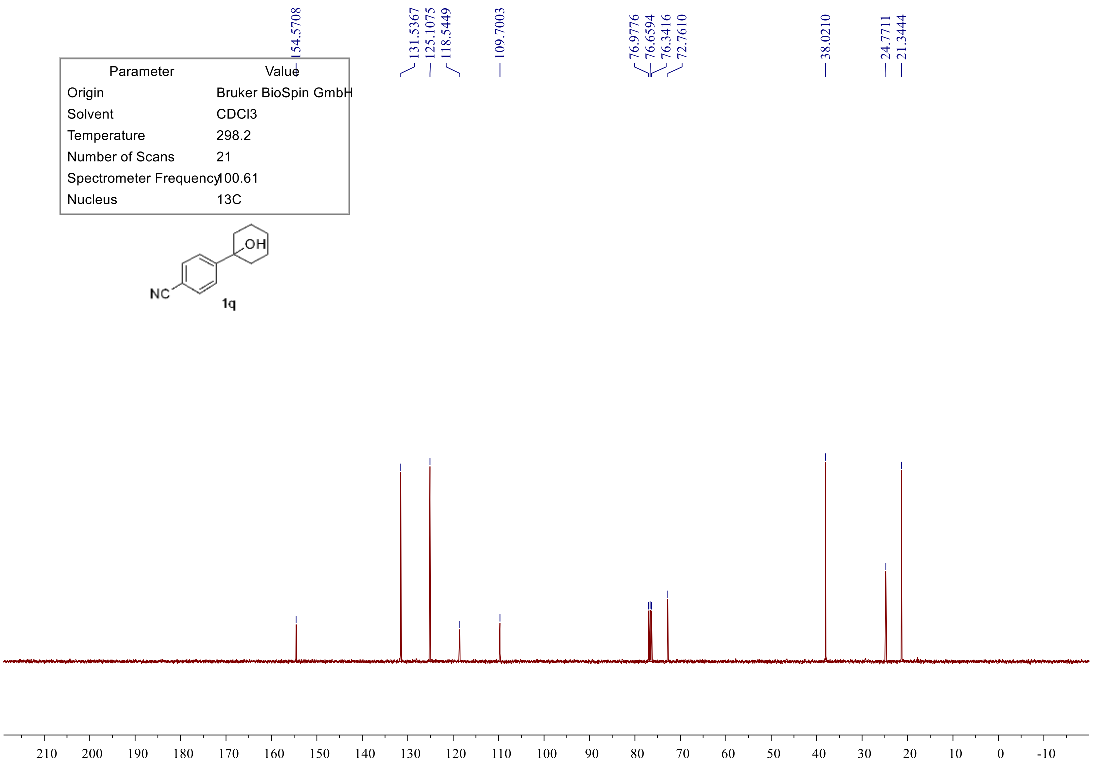
Parameter Value
Origin Bruker BioSpin GmbH
Solvent CDCl3
Temperature 298.2
Number of Scans 21
Spectrometer Frequency 100.61
Nucleus 13C
154.5708
131.5367
125.1075
118.5449
109.7003
76.9776
76.6594
76.3416
72.7610
38.0210
24.7711
21.3444
OH
NC
1q

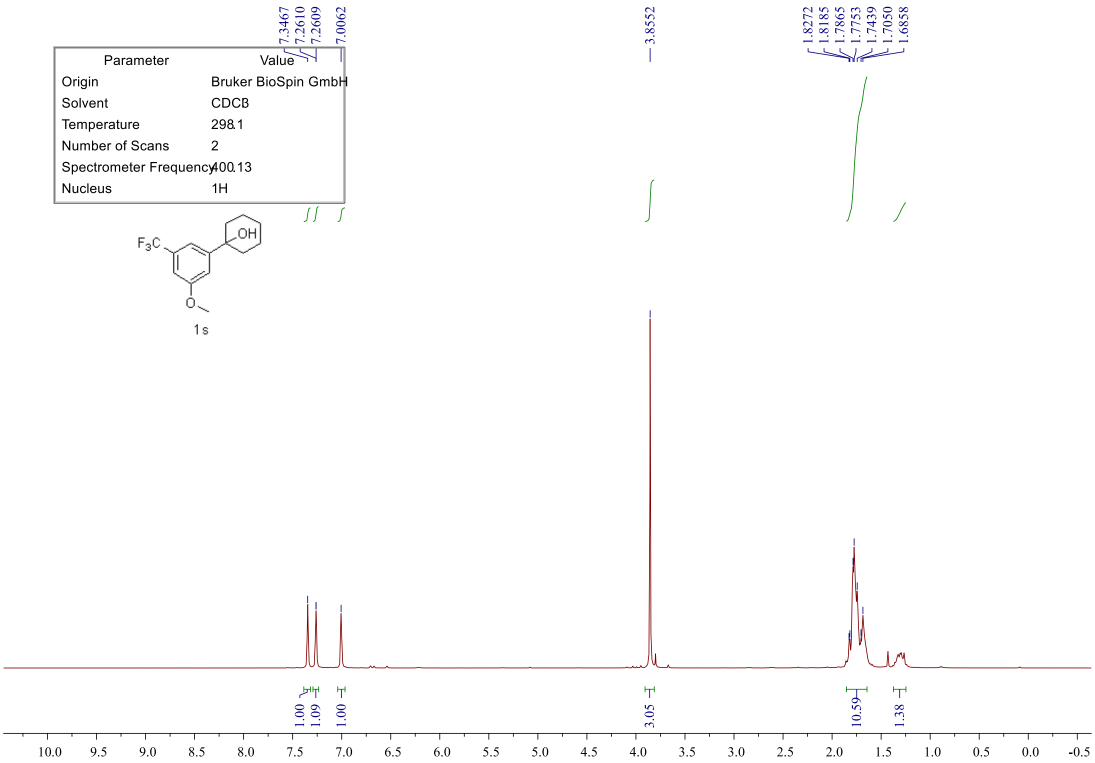
Parameter Value
Origin Bruker BioSpin GmbH
Solvent CDCl3
Temperature 298.1
Number of Scans 2
Spectrometer Frequency 400.13
Nucleus 1H
7.3467
7.2610
7.2609
7.0062
3.8552
1.8272
1.8185
1.7865
1.7753
1.7439
1.7050
1.6858
F3C
OH
O
1s
1.00
1.09
1.00
3.05
10.59
1.38

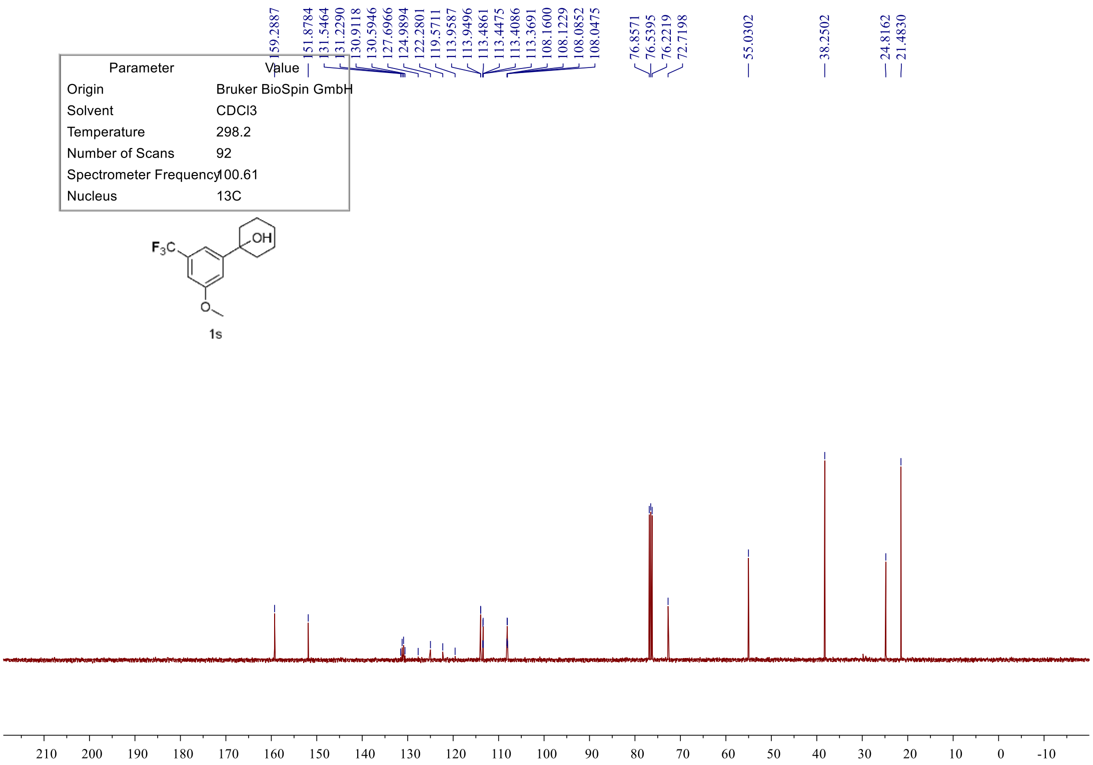
159.2887
151.8784
131.5464
131.2290
130.9118
130.5946
127.6966
124.9894
122.2801
119.5711
113.9587
113.9496
113.4861
113.4475
113.4086
113.3691
108.1600
108.1229
108.0852
108.0475
76.8571
76.5395
76.2219
72.7198
55.0302
38.2502
24.8162
21.4830
Parameter Value
Origin Bruker BioSpin GmbH
Solvent CDCl3
Temperature 298.2
Number of Scans 92
Spectrometer Frequency 100.61
Nucleus 13C
F3C
OH
O
1s
210 200 190 180 170 160 150 140 130 120 110 100 90 80 70 60 50 40 30 20 10 0 -10

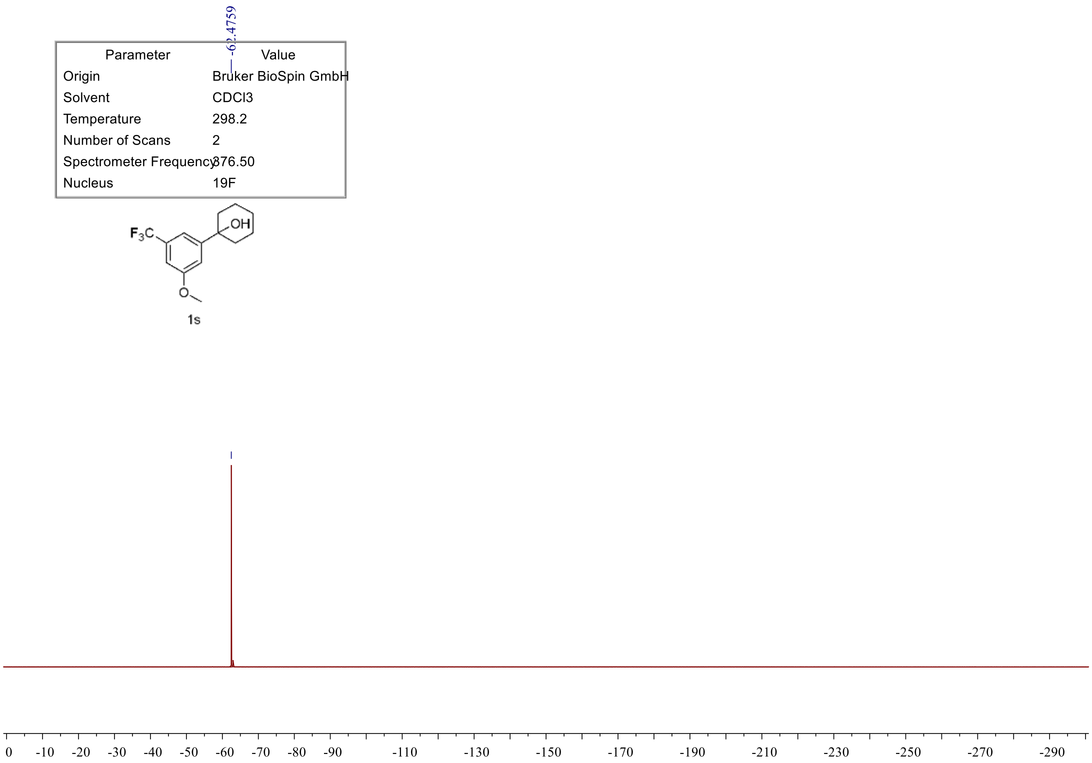
-62.4759
Parameter Value
Origin Bruker BioSpin GmbH
Solvent CDCl3
Temperature 298.2
Number of Scans 2
Spectrometer Frequency 376.50
Nucleus 19F
F3C
OH
O
1s
0 -10 -20 -30 -40 -50 -60 -70 -80 -90 -110 -130 -150 -170 -190 -210 -230 -250 -270 -290

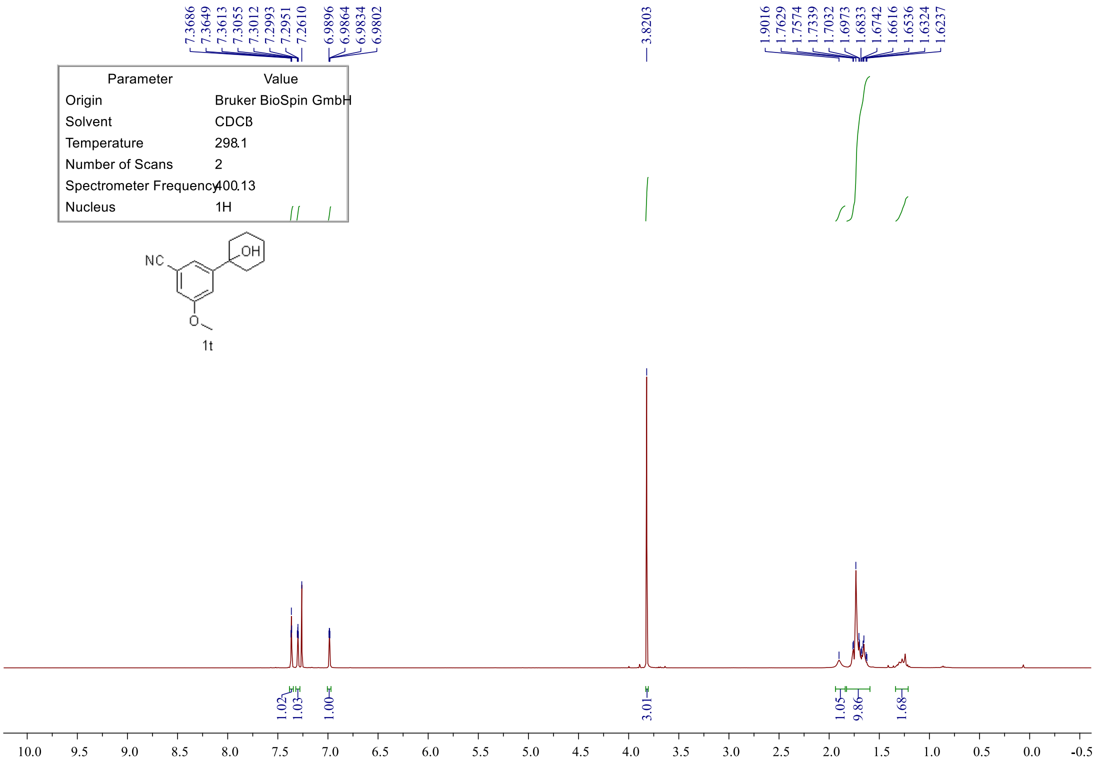
7.3686 7.3649 7.3613 7.3055 7.3012 7.2993 7.2951 7.2610 6.9896 6.9864 6.9834 6.9802
3.8203
1.9016 1.7629 1.7574 1.7339 1.7032 1.6973 1.6833 1.6742 1.6616 1.6536 1.6324 1.6237
Parameter Value
Origin Bruker BioSpin GmbH
Solvent CDCl3
Temperature 298.1
Number of Scans 2
Spectrometer Frequency 400.13
Nucleus 1H
NC
OH
O
1t
1.02 1.03 1.00 3.01 1.05 9.86 1.68
10.0 9.5 9.0 8.5 8.0 7.5 7.0 6.5 6.0 5.5 5.0 4.5 4.0 3.5 3.0 2.5 2.0 1.5 1.0 0.5 0.0 -0.5

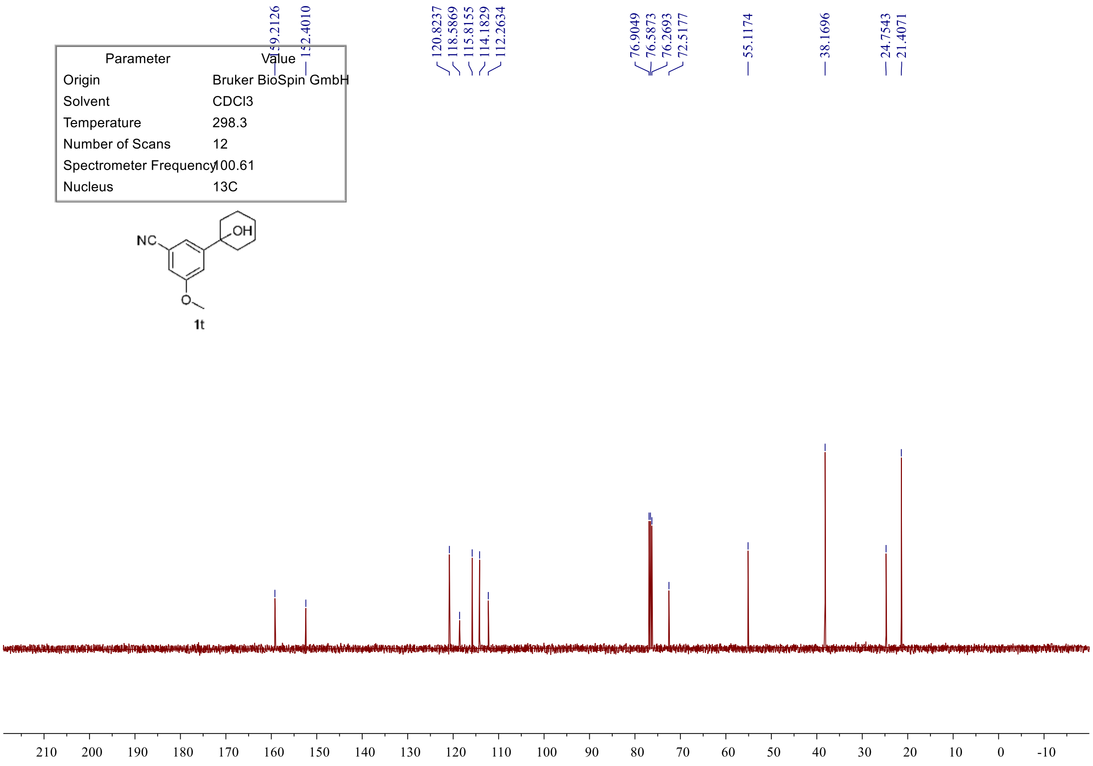
159.2126 152.4010
120.8237 118.5869 115.8155 114.1829 112.2634
76.9049 76.5873 76.2693 72.5177
55.1174
38.1696
24.7543 21.4071
Parameter Value
Origin Bruker BioSpin GmbH
Solvent CDCl3
Temperature 298.3
Number of Scans 12
Spectrometer Frequency 100.61
Nucleus 13C
NC
OH
O
1t
210 200 190 180 170 160 150 140 130 120 110 100 90 80 70 60 50 40 30 20 10 0 -10

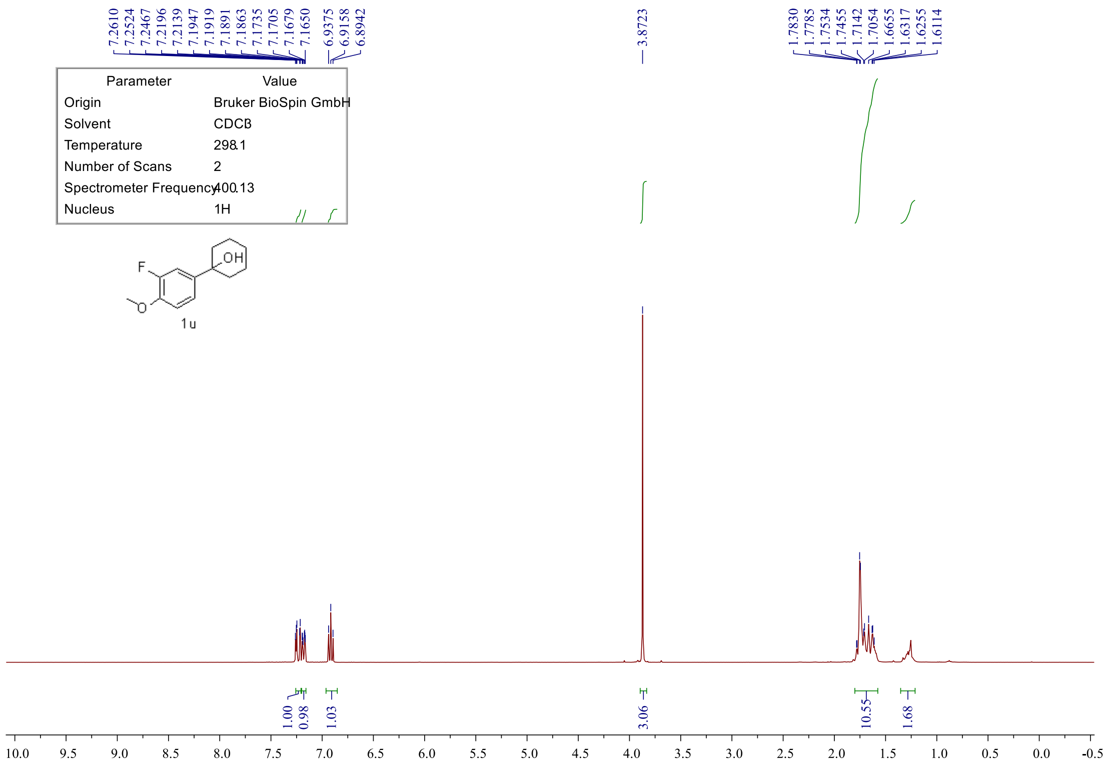
7.2610 7.2524 7.2467 7.2196 7.2139 7.1947 7.1919 7.1891 7.1863 7.1735 7.1705 7.1679 7.1650 6.9375 6.9158 6.8942
3.8723
1.7830 1.7785 1.7534 1.7455 1.7142 1.7054 1.6655 1.6317 1.6255 1.6114
Parameter Value
Origin Bruker BioSpin GmbH
Solvent CDCl3
Temperature 298.1
Number of Scans 2
Spectrometer Frequency 400.13
Nucleus 1H
F
O
OH
1u
1.00 0.98 1.03 3.06 10.55 1.68
10.0 9.5 9.0 8.5 8.0 7.5 7.0 6.5 6.0 5.5 5.0 4.5 4.0 3.5 3.0 2.5 2.0 1.5 1.0 0.5 0.0 -0.5

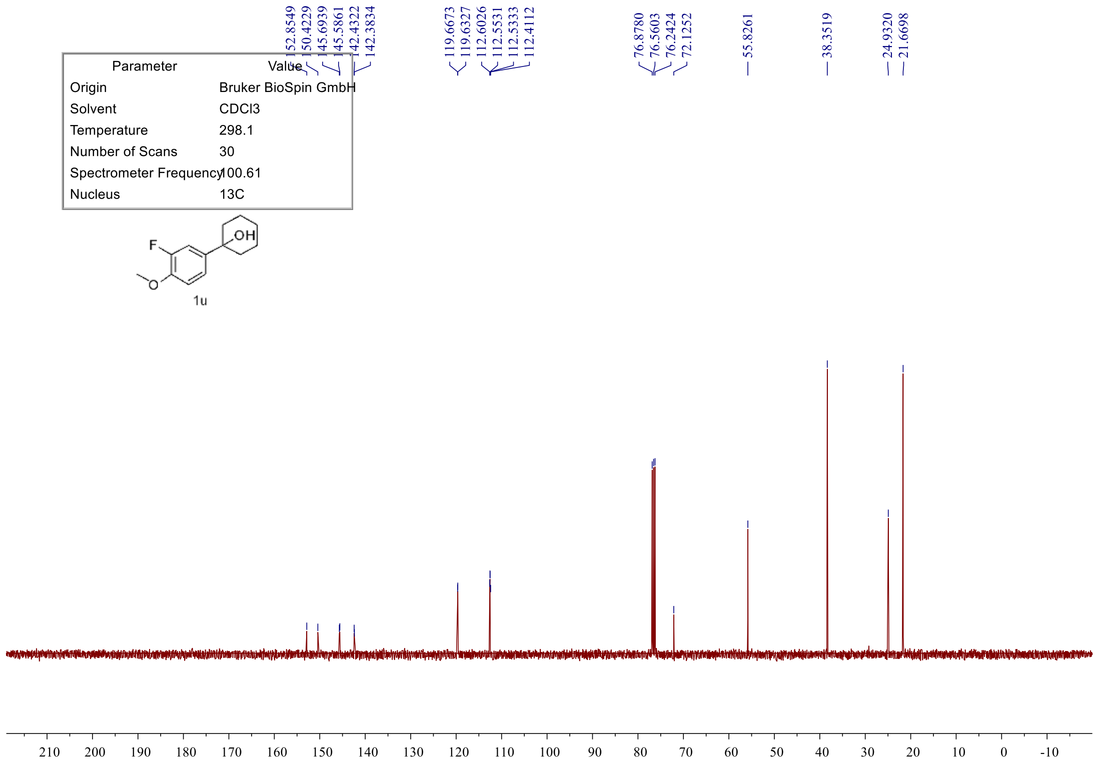
152.8549 150.4229 145.6939 145.5861 142.4322 142.3834
119.6673 119.6327 112.6026 112.5531 112.5333 112.4112
76.8780 76.5603 76.2424 72.1252
55.8261
38.3519
24.9320 21.6698
Parameter Value
Origin Bruker BioSpin GmbH
Solvent CDCl3
Temperature 298.1
Number of Scans 30
Spectrometer Frequency 100.61
Nucleus 13C
F
O
OH
1u
210 200 190 180 170 160 150 140 130 120 110 100 90 80 70 60 50 40 30 20 10 0 -10

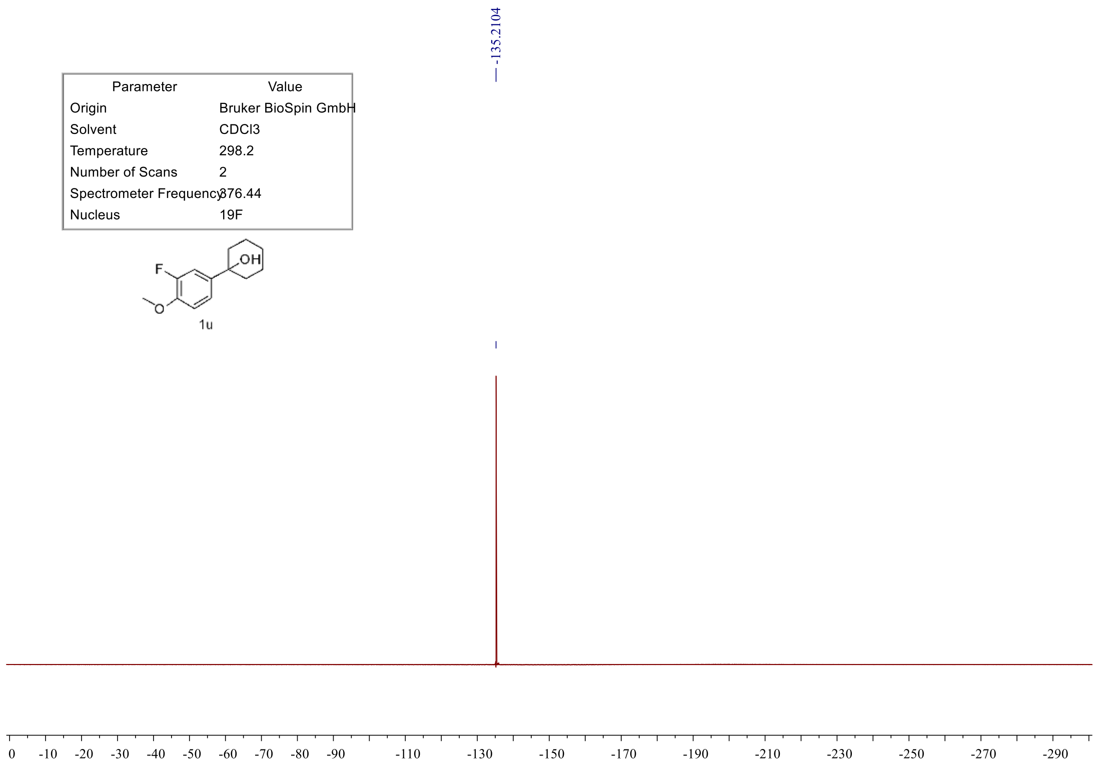
-135.2104
Parameter Value
Origin Bruker BioSpin GmbH
Solvent CDCl3
Temperature 298.2
Number of Scans 2
Spectrometer Frequency 376.44
Nucleus 19F
F
O
OH
1u
0 -10 -20 -30 -40 -50 -60 -70 -80 -90 -110 -130 -150 -170 -190 -210 -230 -250 -270 -290

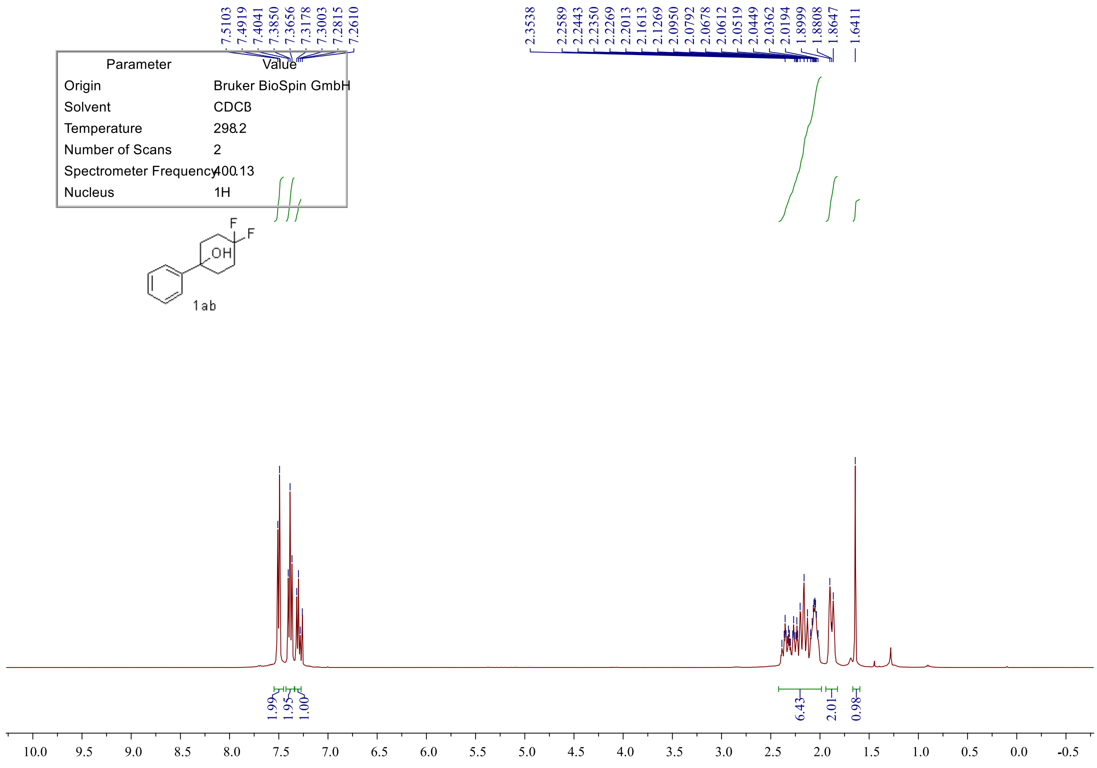
7.5103 7.4919 7.4041 7.3850 7.3656 7.3178 7.3003 7.2815 7.2610
2.3538 2.2589 2.2443 2.2350 2.2269 2.2013 2.1613 2.1269 2.0950 2.0792 2.0678 2.0612 2.0519 2.0449 2.0362 2.0194 1.8999 1.8808 1.8647
1.6411
Parameter Value
Origin Bruker BioSpin GmbH
Solvent CDCl3
Temperature 298.2
Number of Scans 2
Spectrometer Frequency 400.13
Nucleus 1H
F
F
OH
1ab
1.99 1.95 1.00
6.43 2.01 0.98
10.0 9.5 9.0 8.5 8.0 7.5 7.0 6.5 6.0 5.5 5.0 4.5 4.0 3.5 3.0 2.5 2.0 1.5 1.0 0.5 0.0 -0.5

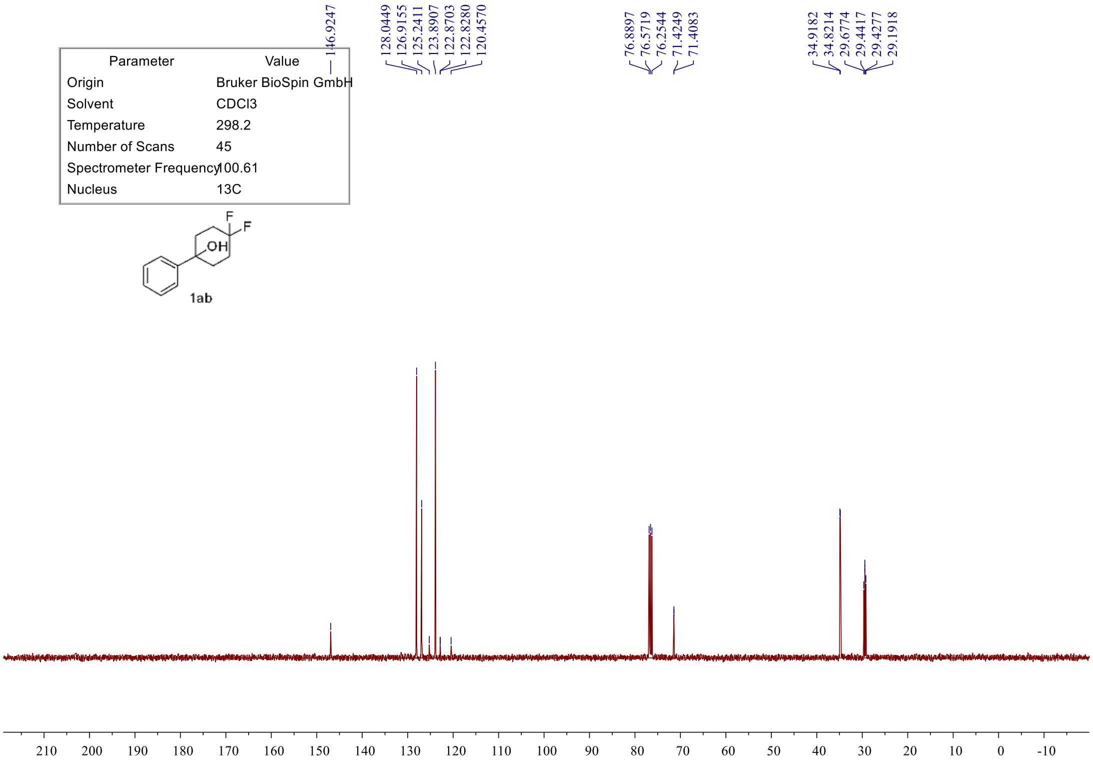
Parameter Value
Origin Bruker BioSpin GmbH
Solvent CDCl3
Temperature 298.2
Number of Scans 45
Spectrometer Frequency 100.61
Nucleus 13C
146.9247
128.0449
126.9155
125.2411
123.8907
122.8703
122.8280
120.4570
76.8897
76.5719
76.2544
71.4249
71.4083
34.9182
34.8214
29.6774
29.4417
29.4277
29.1918
1ab
210 200 190 180 170 160 150 140 130 120 110 100 90 80 70 60 50 40 30 20 10 0 -10

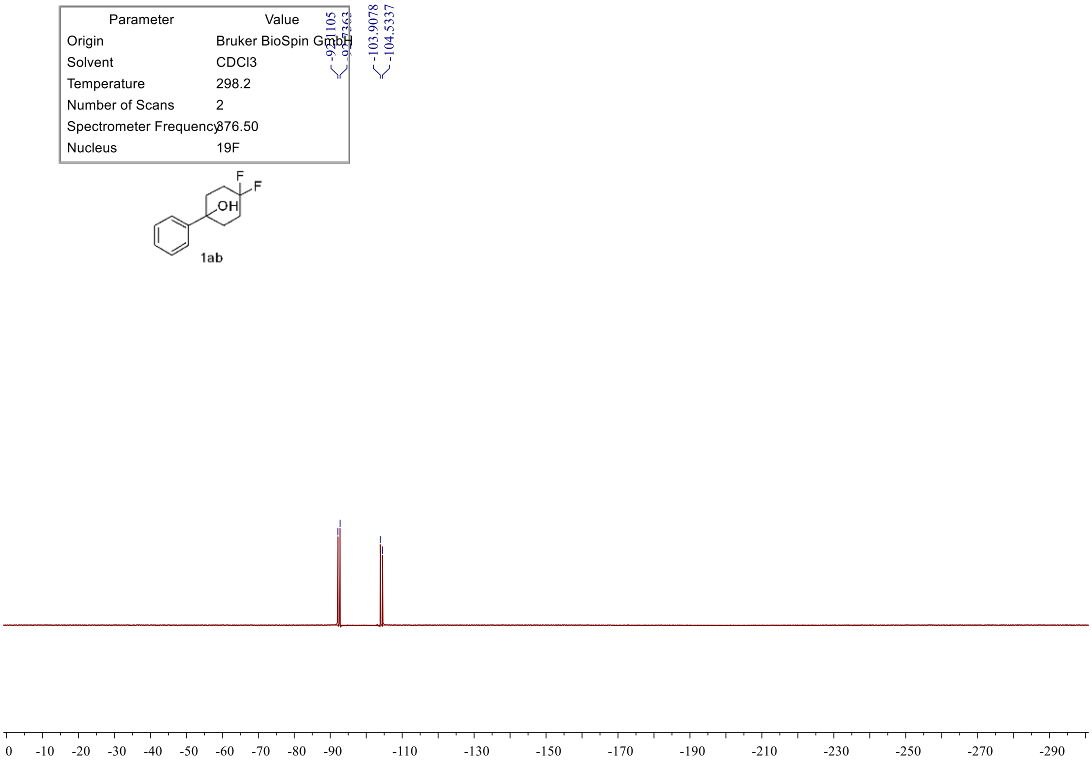
Parameter Value
Origin Bruker BioSpin GmbH
Solvent CDCl3
Temperature 298.2
Number of Scans 2
Spectrometer Frequency 376.50
Nucleus 19F
-103.9078
-104.5337
1ab
0 -10 -20 -30 -40 -50 -60 -70 -80 -90 -110 -130 -150 -170 -190 -210 -230 -250 -270 -290

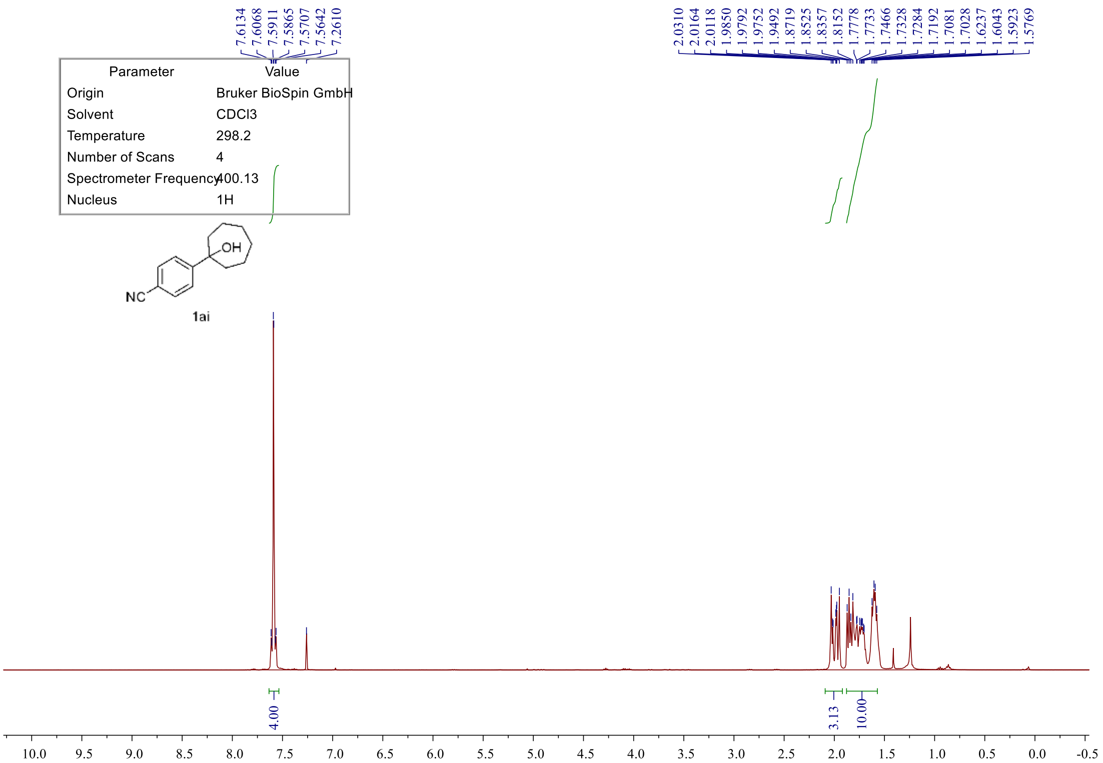
Parameter Value
Origin Bruker BioSpin GmbH
Solvent CDCl3
Temperature 298.2
Number of Scans 4
Spectrometer Frequency 400.13
Nucleus 1H
OH
NC
1ai
7.6134 7.6068 7.5911 7.5865 7.5707 7.5642 7.2610
2.0310 2.0164 2.0118 1.9850 1.9792 1.9752 1.9492 1.8719 1.8525 1.8357 1.8152 1.7778 1.7733 1.7466 1.7328 1.7284 1.7192 1.7081 1.7028 1.6237 1.6043 1.5923 1.5769
4.00
3.13
10.00
10.0 9.5 9.0 8.5 8.0 7.5 7.0 6.5 6.0 5.5 5.0 4.5 4.0 3.5 3.0 2.5 2.0 1.5 1.0 0.5 0.0 -0.5

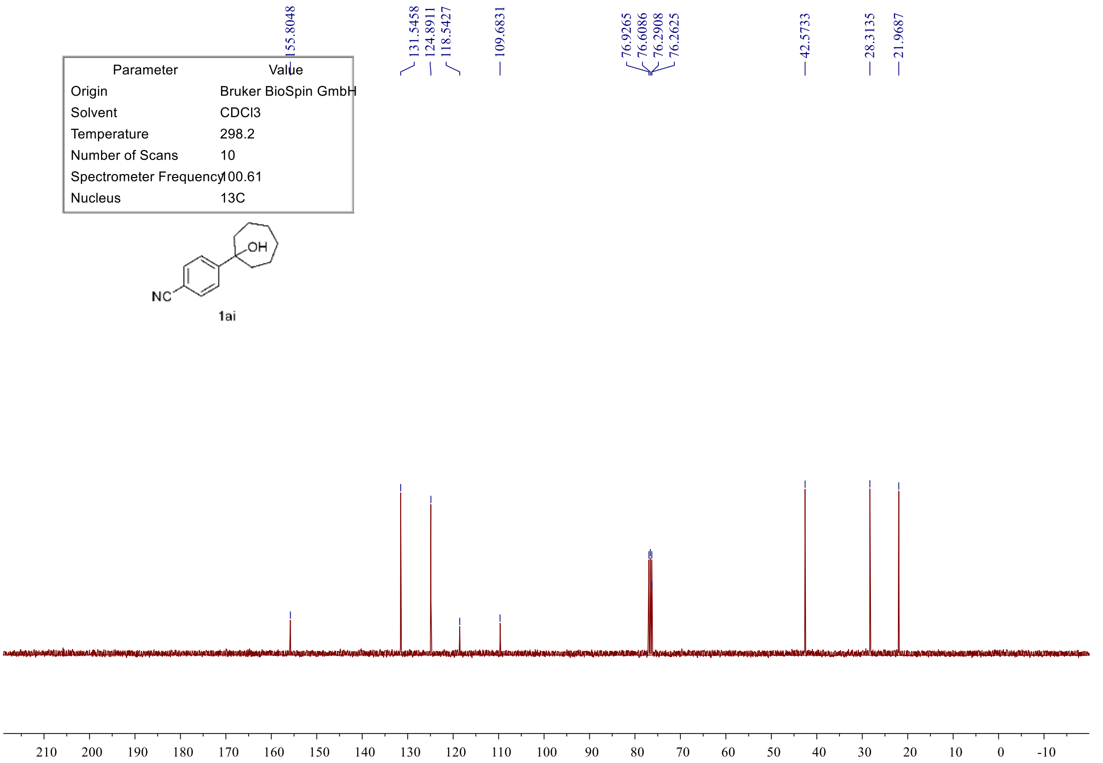
Parameter Value
Origin Bruker BioSpin GmbH
Solvent CDCl3
Temperature 298.2
Number of Scans 10
Spectrometer Frequency 100.61
Nucleus 13C
OH
NC
1ai
155.8048
131.5458 124.8911 118.5427
109.6831
76.9265 76.6086 76.2908 76.2625
42.5733
28.3135
21.9687
210 200 190 180 170 160 150 140 130 120 110 100 90 80 70 60 50 40 30 20 10 0 -10

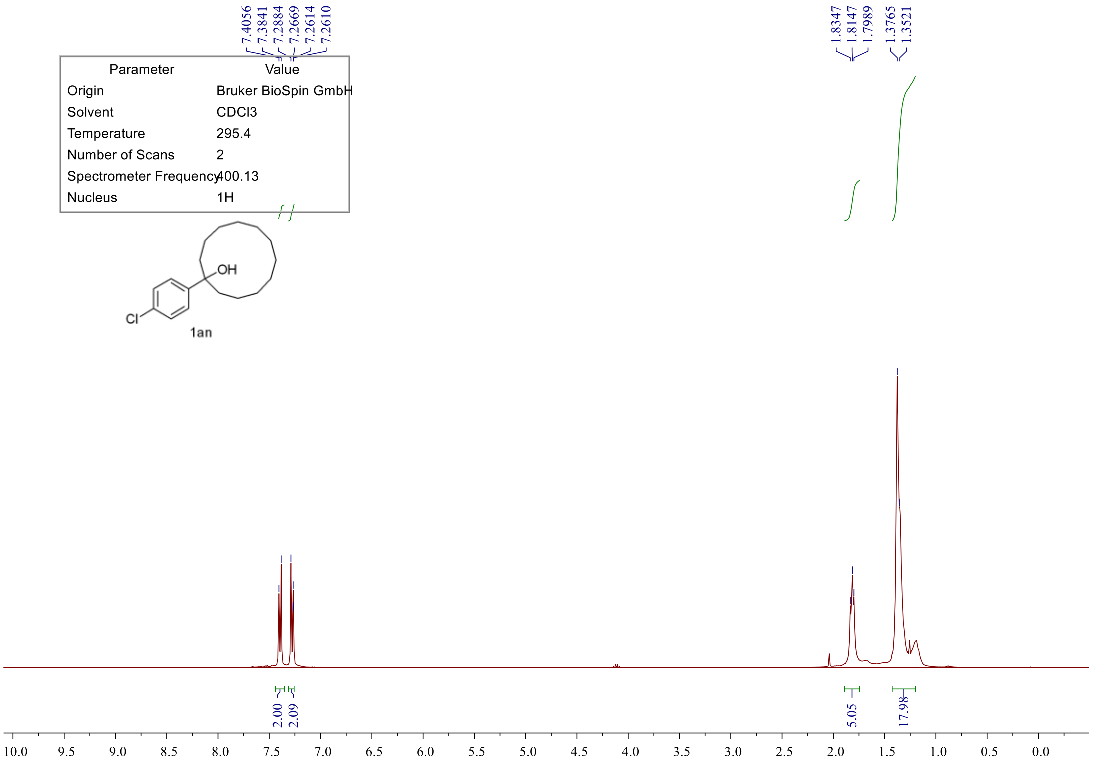

Parameter Value
Origin Bruker BioSpin GmbH
Solvent CDCl3
Temperature 295.4
Number of Scans 2
Spectrometer Frequency 400.13
Nucleus 1H
OH
Cl
1an
7.4056 7.3841 7.2884 7.2669 7.2614 7.2610
1.8347 1.8147 1.7989 1.3765 1.3521
2.00 2.09 5.05 17.98
10.0 9.5 9.0 8.5 8.0 7.5 7.0 6.5 6.0 5.5 5.0 4.5 4.0 3.5 3.0 2.5 2.0 1.5 1.0 0.5 0.0

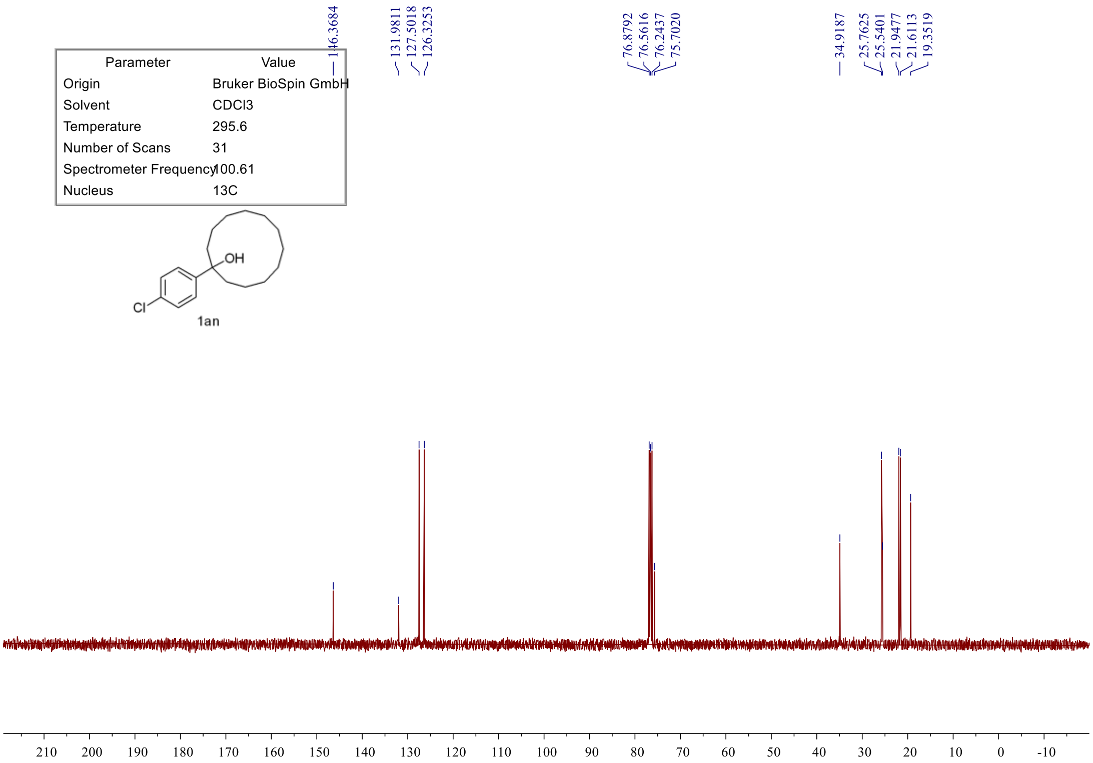

Parameter Value
Origin Bruker BioSpin GmbH
Solvent CDCl3
Temperature 295.6
Number of Scans 31
Spectrometer Frequency 100.61
Nucleus 13C
OH
Cl
1an
146.3684
131.9811 127.5018 126.3253
76.8792 76.5616 76.2437 75.7020
34.9187 25.7625 25.5401 21.9477 21.6113 19.3519
210 200 190 180 170 160 150 140 130 120 110 100 90 80 70 60 50 40 30 20 10 0 -10

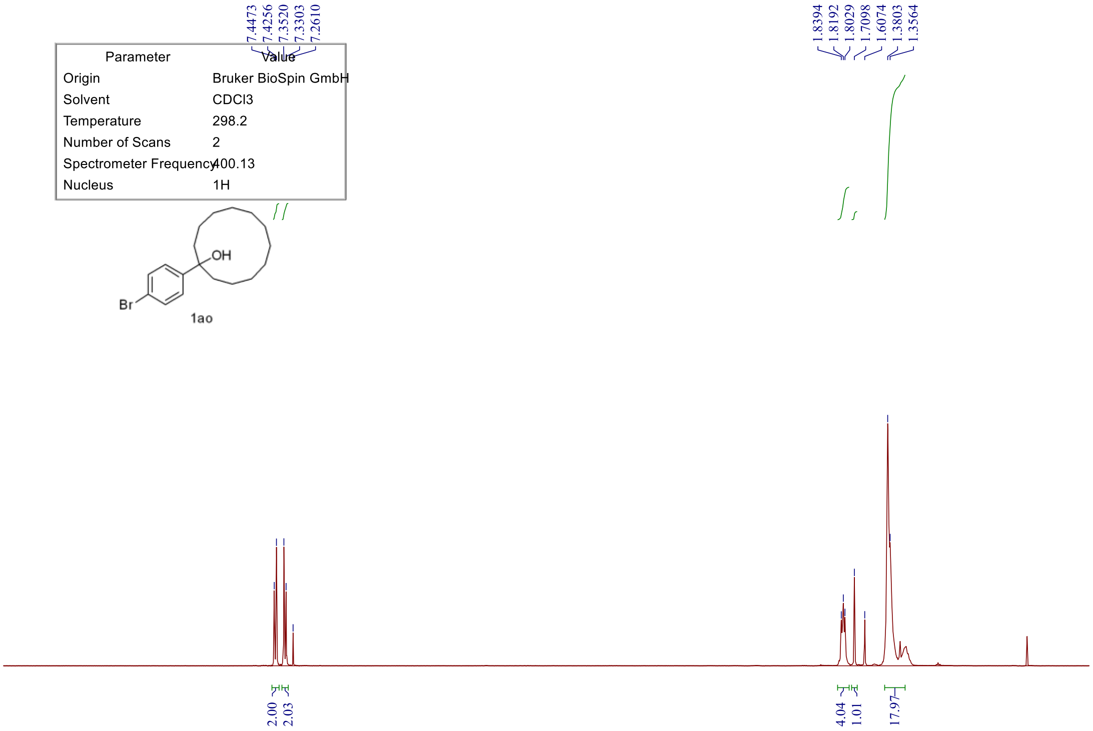

Parameter	Value
Origin	Bruker BioSpin GmbH
Solvent	CDCl3
Temperature	298.2
Number of Scans	2
Spectrometer Frequency	400.13
Nucleus	1H

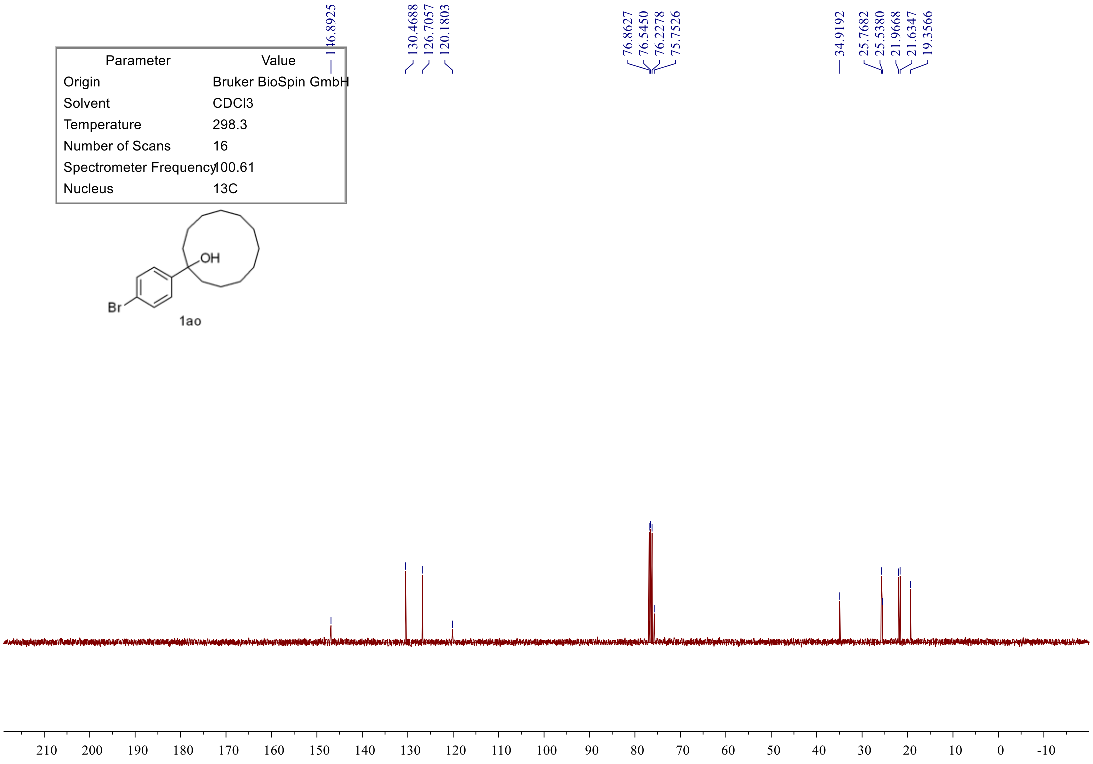

Parameter	Value
Origin	Bruker BioSpin GmbH
Solvent	CDCl3
Temperature	298.3
Number of Scans	16
Spectrometer Frequency	100.61
Nucleus	13C

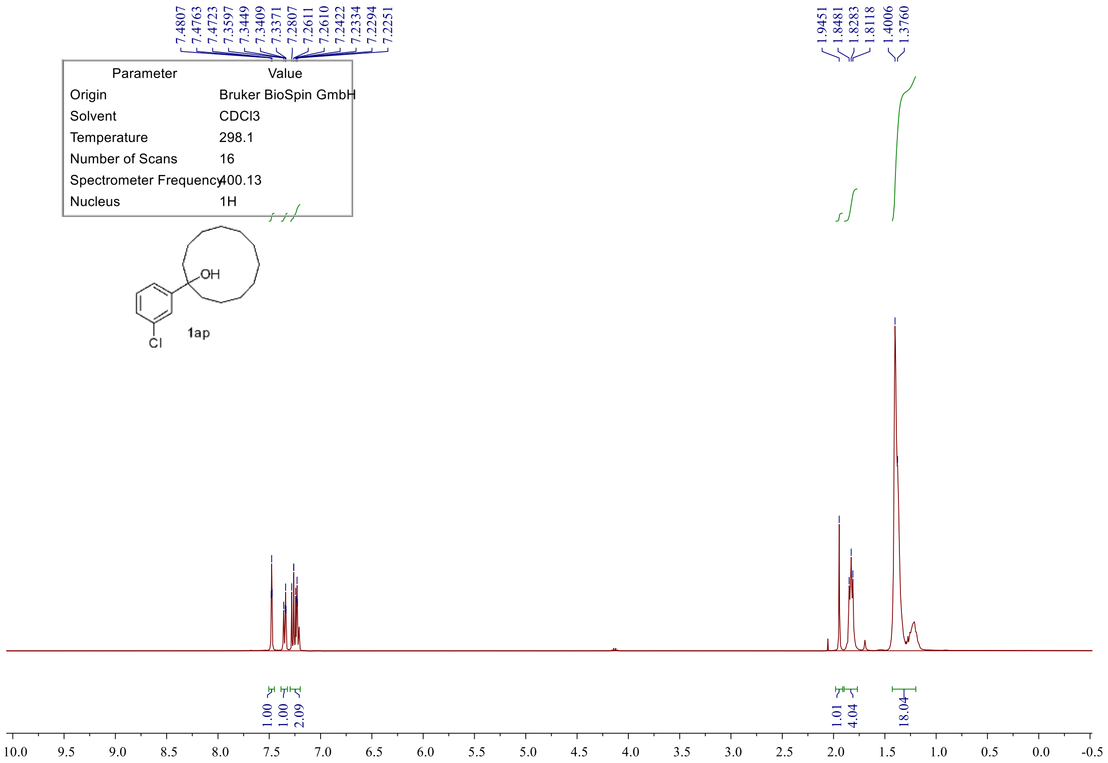
7.4807
7.4763
7.4723
7.3597
7.3449
7.3409
7.3371
7.2807
7.2611
7.2610
7.2422
7.2334
7.2294
7.2251
1.9451
1.8481
1.8283
1.8118
1.4006
1.3760
Parameter Value
Origin Bruker BioSpin GmbH
Solvent CDCl3
Temperature 298.1
Number of Scans 16
Spectrometer Frequency 400.13
Nucleus 1H
OH
Cl
1ap
1.00
1.00
2.09
1.01
4.04
18.04
10.0 9.5 9.0 8.5 8.0 7.5 7.0 6.5 6.0 5.5 5.0 4.5 4.0 3.5 3.0 2.5 2.0 1.5 1.0 0.5 0.0 -0.5

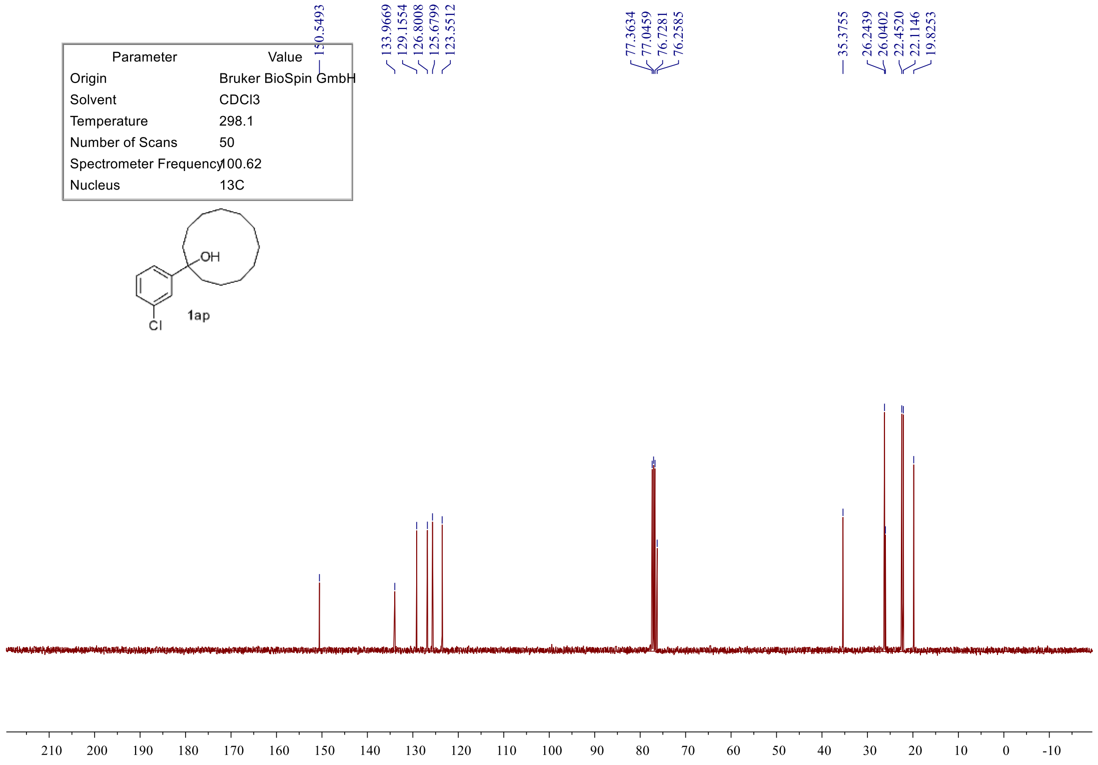
150.5493
133.9669
129.1554
126.8008
125.6799
123.5512
77.3634
77.0459
76.7281
76.2585
35.3755
26.2439
26.0402
22.4520
22.1146
19.8253
Parameter Value
Origin Bruker BioSpin GmbH
Solvent CDCl3
Temperature 298.1
Number of Scans 50
Spectrometer Frequency 100.62
Nucleus 13C
OH
Cl
1ap
210 200 190 180 170 160 150 140 130 120 110 100 90 80 70 60 50 40 30 20 10 0 -10

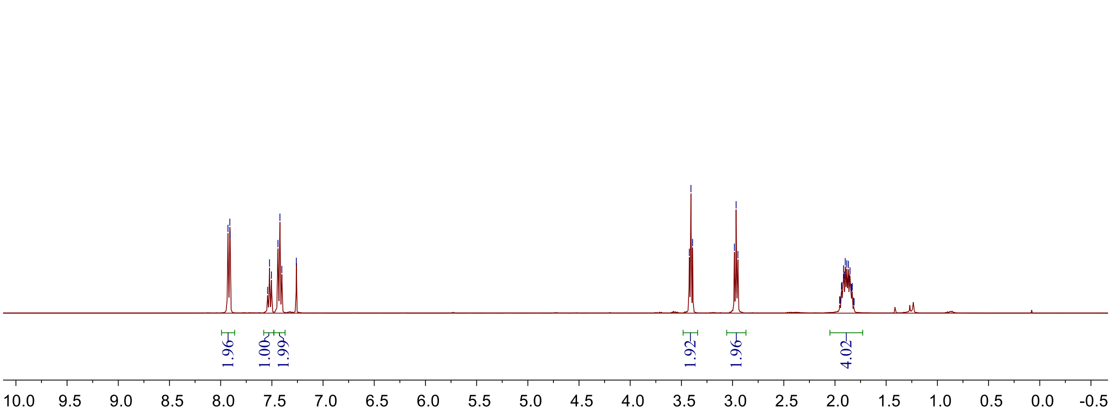

Parameter	Value
1 Origin	Bruker BioSpin GmbH
2 Solvent	CDCl3
3 Temperature	298.2
4 Number of Scans	2
5 Spectrometer Frequency	400.13
6 Nucleus	1H

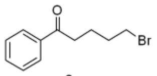

2a

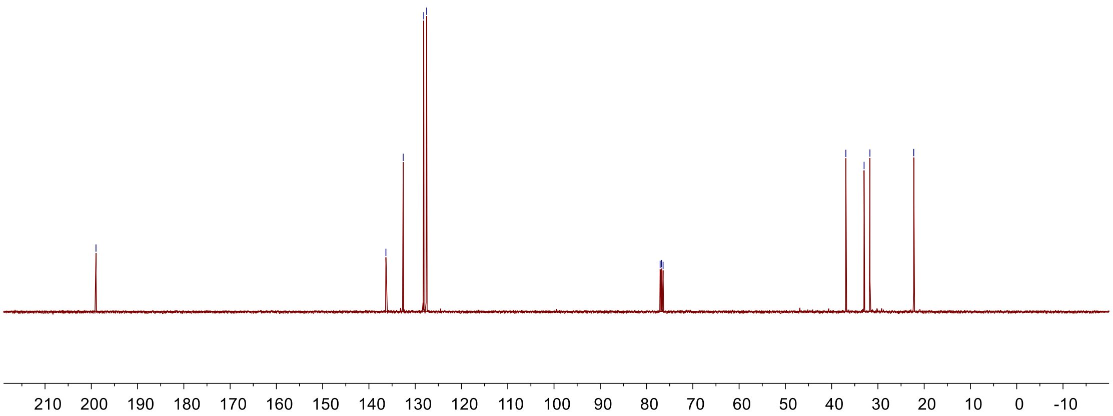

Parameter	Value
1 Origin	Bruker BioSpin GmbH
2 Solvent	CDCl3
3 Temperature	298.2
4 Number of Scans	19
5 Spectrometer Frequency	100.61
6 Nucleus	13C

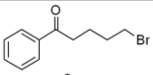

2a

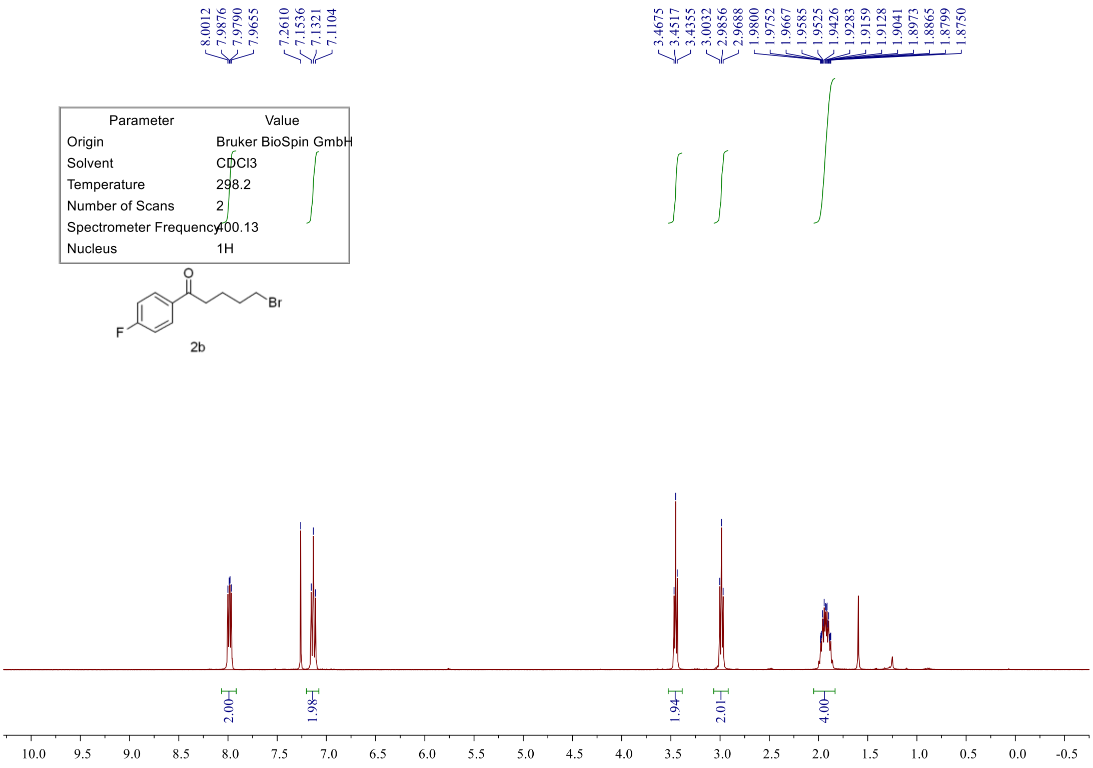
8.0012
7.9876
7.9790
7.9655
7.2610
7.1536
7.1321
7.1104
3.4675
3.4517
3.4355
3.0032
2.9856
2.9688
1.9800
1.9752
1.9667
1.9585
1.9525
1.9426
1.9283
1.9159
1.9128
1.9041
1.8973
1.8865
1.8799
1.8750
Parameter Value
Origin Bruker BioSpin GmbH
Solvent CDCl3
Temperature 298.2
Number of Scans 2
Spectrometer Frequency 400.13
Nucleus 1H
2b
2.00
1.98
1.94
2.01
4.00

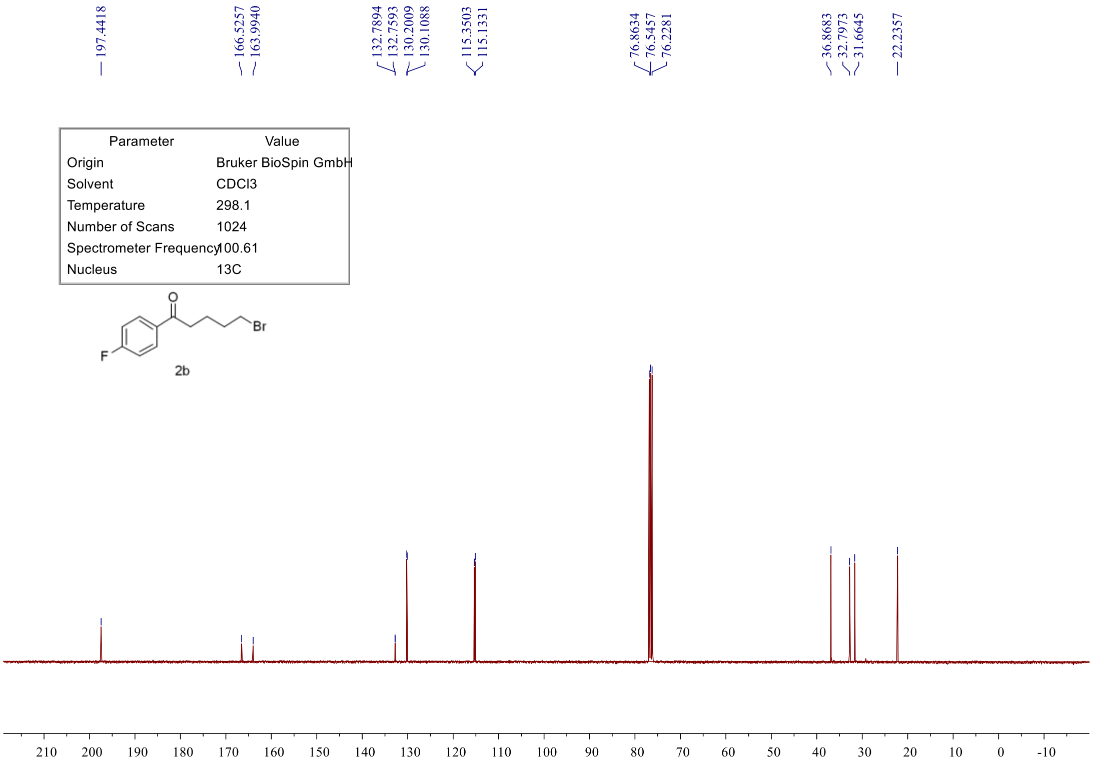
197.4418
166.5257
163.9940
132.7894
132.7593
130.2009
130.1088
115.3503
115.1331
76.8634
76.5457
76.2281
36.8683
32.7973
31.6645
22.2357
Parameter Value
Origin Bruker BioSpin GmbH
Solvent CDCl3
Temperature 298.1
Number of Scans 1024
Spectrometer Frequency 100.61
Nucleus 13C
2b

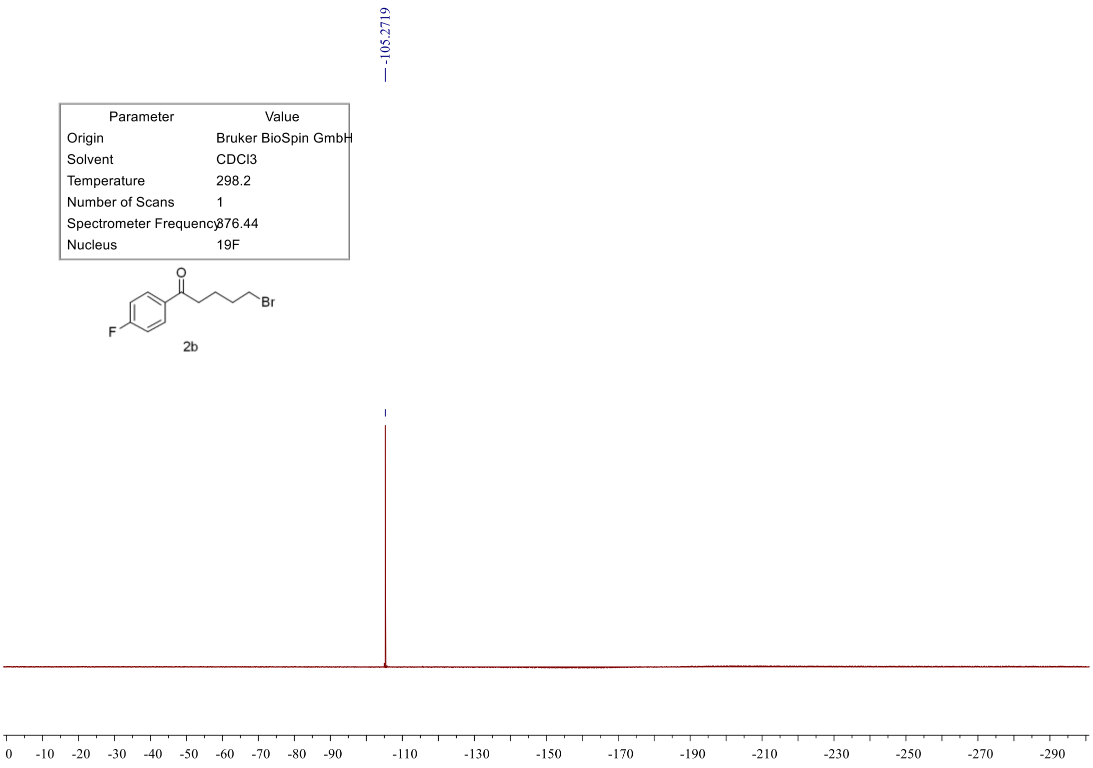
-105.2719
Parameter Value
Origin Bruker BioSpin GmbH
Solvent CDCl3
Temperature 298.2
Number of Scans 1
Spectrometer Frequency 376.44
Nucleus 19F
O
F
Br
2b
0 -10 -20 -30 -40 -50 -60 -70 -80 -90 -110 -130 -150 -170 -190 -210 -230 -250 -270 -290

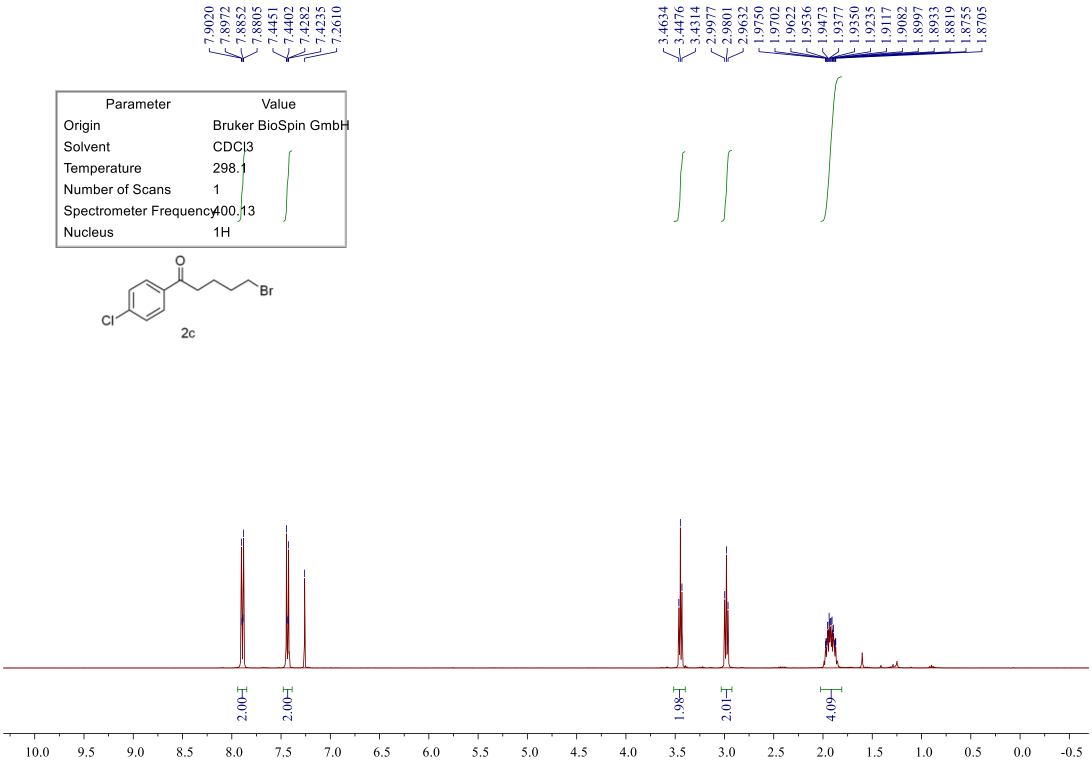
7.9020 7.8972 7.8852 7.8805 7.4451 7.4402 7.4282 7.4235 7.2610
3.4634 3.4476 3.4314 2.9977 2.9801 2.9632 1.9750 1.9702 1.9622 1.9536 1.9473 1.9377 1.9350 1.9235 1.9117 1.9082 1.8997 1.8933 1.8819 1.8755 1.8705
Parameter Value
Origin Bruker BioSpin GmbH
Solvent CDCl3
Temperature 298.1
Number of Scans 1
Spectrometer Frequency 400.13
Nucleus 1H
O
Cl
Br
2c
2.00 2.00 1.98 2.01 4.09
10.0 9.5 9.0 8.5 8.0 7.5 7.0 6.5 6.0 5.5 5.0 4.5 4.0 3.5 3.0 2.5 2.0 1.5 1.0 0.5 0.0 -0.5

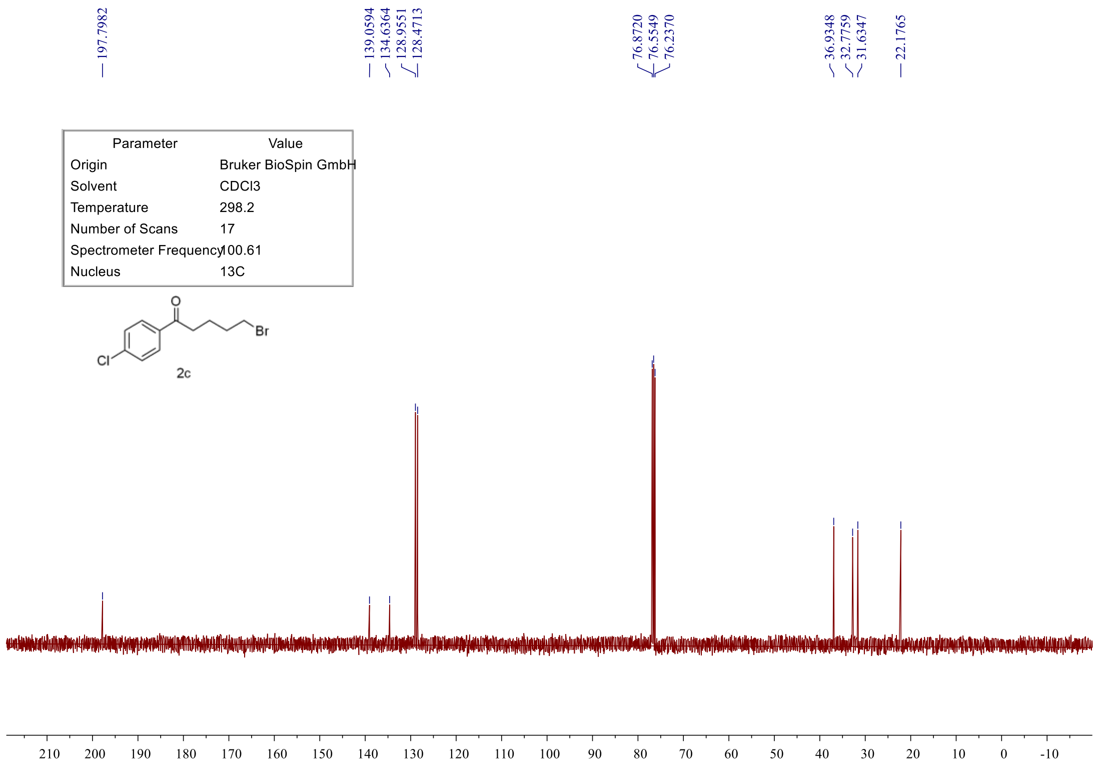
197.7982
139.0594
134.6364
128.9551
128.4713
76.8720
76.5549
76.2370
36.9348
32.7759
31.6347
22.1765
Parameter Value
Origin Bruker BioSpin GmbH
Solvent CDCl3
Temperature 298.2
Number of Scans 17
Spectrometer Frequency 100.61
Nucleus 13C
O
Br
Cl
2c
210 200 190 180 170 160 150 140 130 120 110 100 90 80 70 60 50 40 30 20 10 0 -10

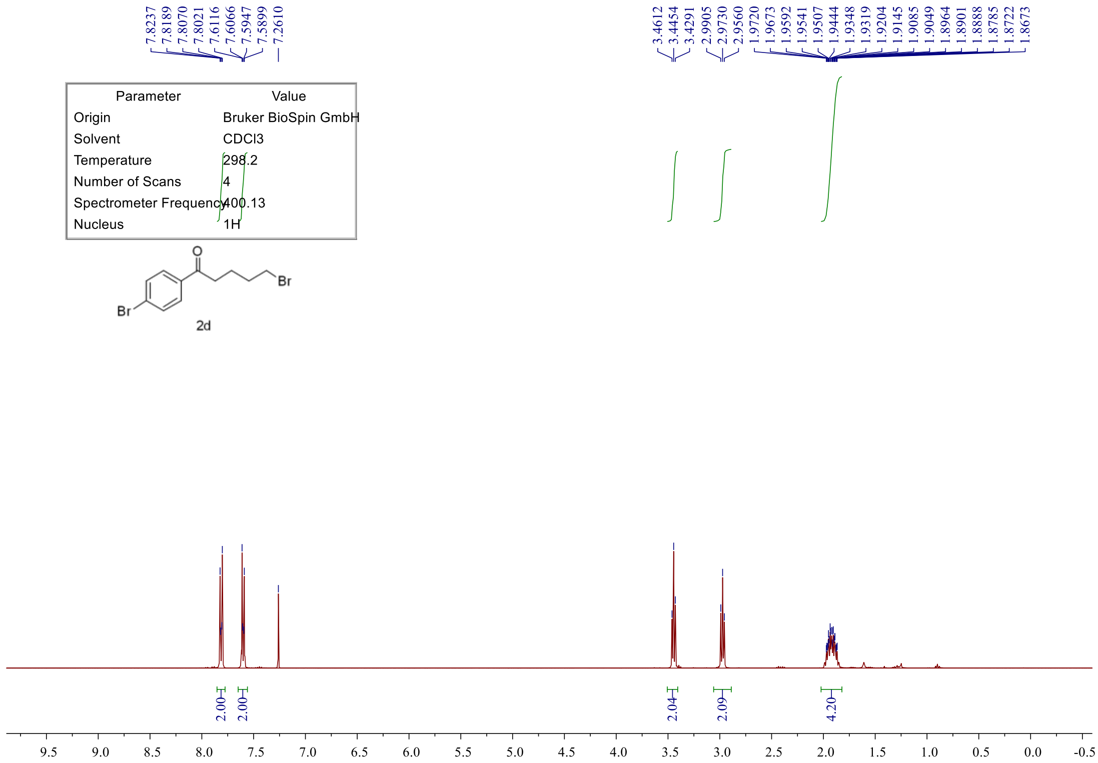
7.8237
7.8189
7.8070
7.8021
7.6116
7.6066
7.5947
7.5899
7.2610
3.4612
3.4454
3.4291
2.9905
2.9730
2.9560
1.9720
1.9673
1.9592
1.9541
1.9507
1.9444
1.9348
1.9319
1.9204
1.9145
1.9085
1.9049
1.8964
1.8901
1.8888
1.8785
1.8722
1.8673
Parameter Value
Origin Bruker BioSpin GmbH
Solvent CDCl3
Temperature 298.2
Number of Scans 4
Spectrometer Frequency 400.13
Nucleus 1H
O
Br
Br
2d
2.00
2.00
2.04
2.09
4.20
9.5 9.0 8.5 8.0 7.5 7.0 6.5 6.0 5.5 5.0 4.5 4.0 3.5 3.0 2.5 2.0 1.5 1.0 0.5 0.0 -0.5

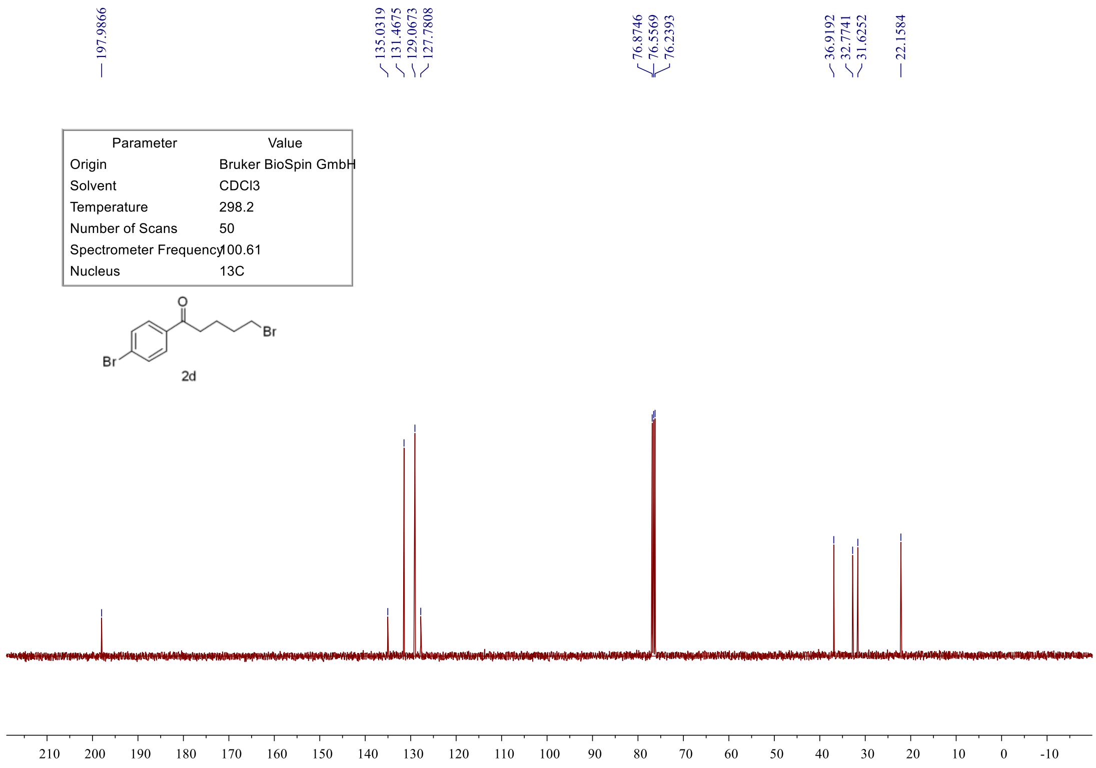
197.9866
135.0319
131.4675
129.0673
127.7808
76.8746
76.5569
76.2393
36.9192
32.7741
31.6252
22.1584
Parameter Value
Origin Bruker BioSpin GmbH
Solvent CDCl3
Temperature 298.2
Number of Scans 50
Spectrometer Frequency 100.61
Nucleus 13C
O
Br
Br
2d
210 200 190 180 170 160 150 140 130 120 110 100 90 80 70 60 50 40 30 20 10 0 -10

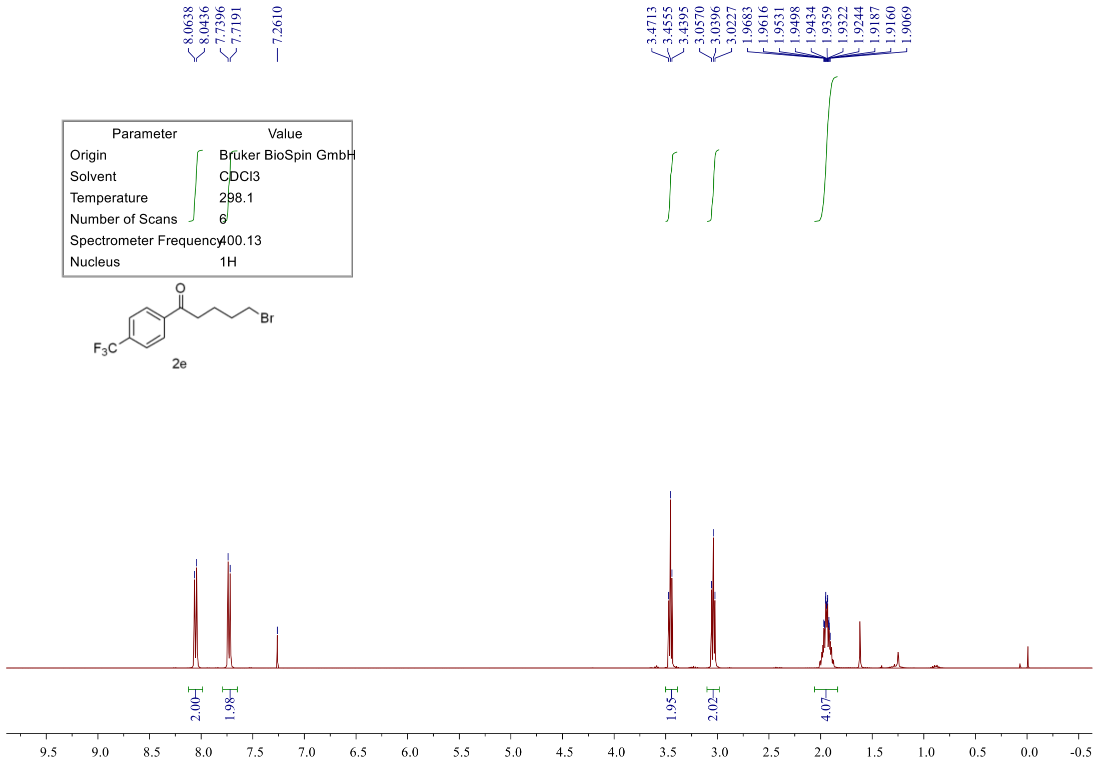
8.0638
8.0436
7.7396
7.7191
7.2610
3.4713
3.4555
3.4395
3.0570
3.0396
3.0227
1.9683
1.9616
1.9531
1.9498
1.9434
1.9359
1.9322
1.9244
1.9187
1.9160
1.9069
Parameter Value
Origin Bruker BioSpin GmbH
Solvent CDCl3
Temperature 298.1
Number of Scans 6
Spectrometer Frequency 400.13
Nucleus 1H
O
Br
F_3C
2e
2.00
1.98
1.95
2.02
4.07
9.5 9.0 8.5 8.0 7.5 7.0 6.5 6.0 5.5 5.0 4.5 4.0 3.5 3.0 2.5 2.0 1.5 1.0 0.5 0.0 -0.5

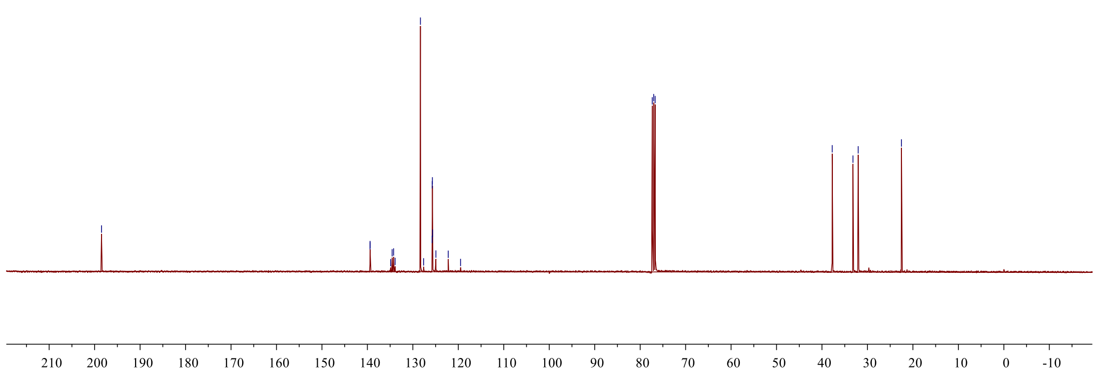

Parameter	Value
Origin	Bruker BioSpin GmbH
Solvent	CDCl3
Temperature	298.2
Number of Scans	1024
Spectrometer Frequency	100.62
Nucleus	13C

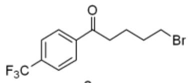

2e

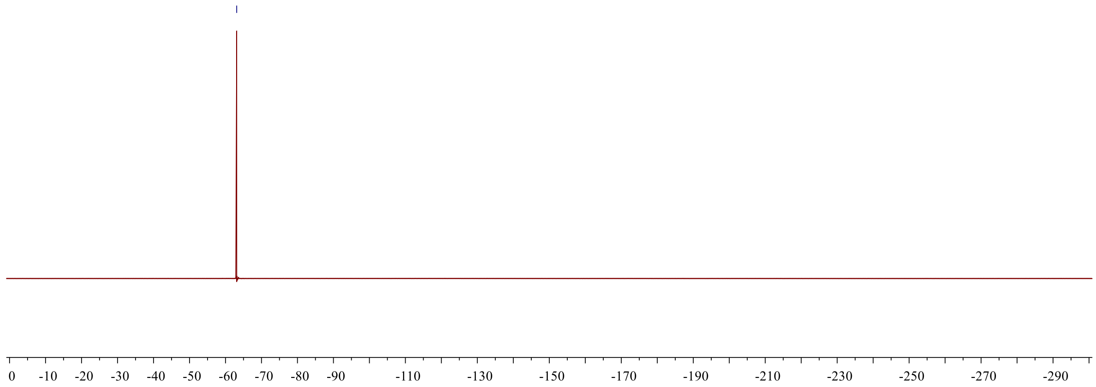

Parameter	Value
Origin	Bruker BioSpin GmbH
Solvent	CDCl3
Temperature	298.2
Number of Scans	4
Spectrometer Frequency	376.50
Nucleus	19F

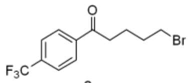

2e

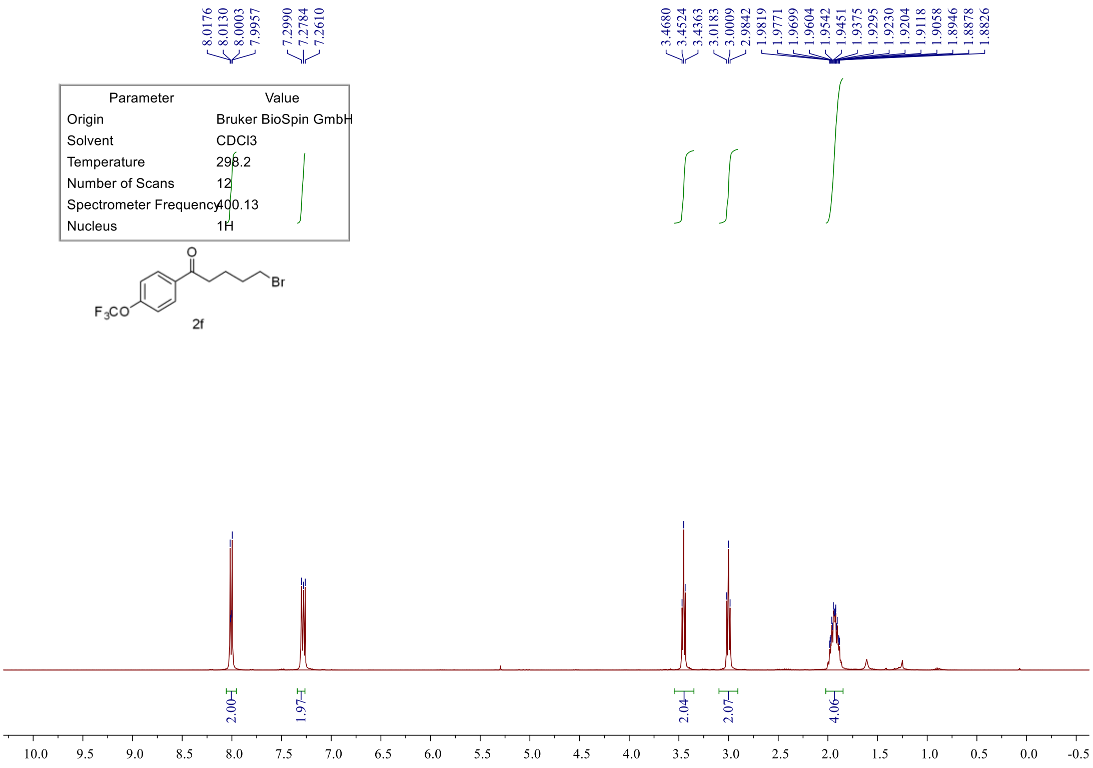

Parameter Value
Origin Bruker BioSpin GmbH
Solvent CDCl3
Temperature 298.2
Number of Scans 12
Spectrometer Frequency 400.13
Nucleus 1H
O
Br
F3CO
2f
8.0176
8.0130
8.0003
7.9957
7.2990
7.2784
7.2610
3.4680
3.4524
3.4363
3.0183
3.0009
2.9842
1.9819
1.9771
1.9699
1.9604
1.9542
1.9451
1.9375
1.9295
1.9230
1.9204
1.9118
1.9058
1.8946
1.8878
1.8826
2.00
1.97
2.04
2.07
4.06
10.0 9.5 9.0 8.5 8.0 7.5 7.0 6.5 6.0 5.5 5.0 4.5 4.0 3.5 3.0 2.5 2.0 1.5 1.0 0.5 0.0 -0.5

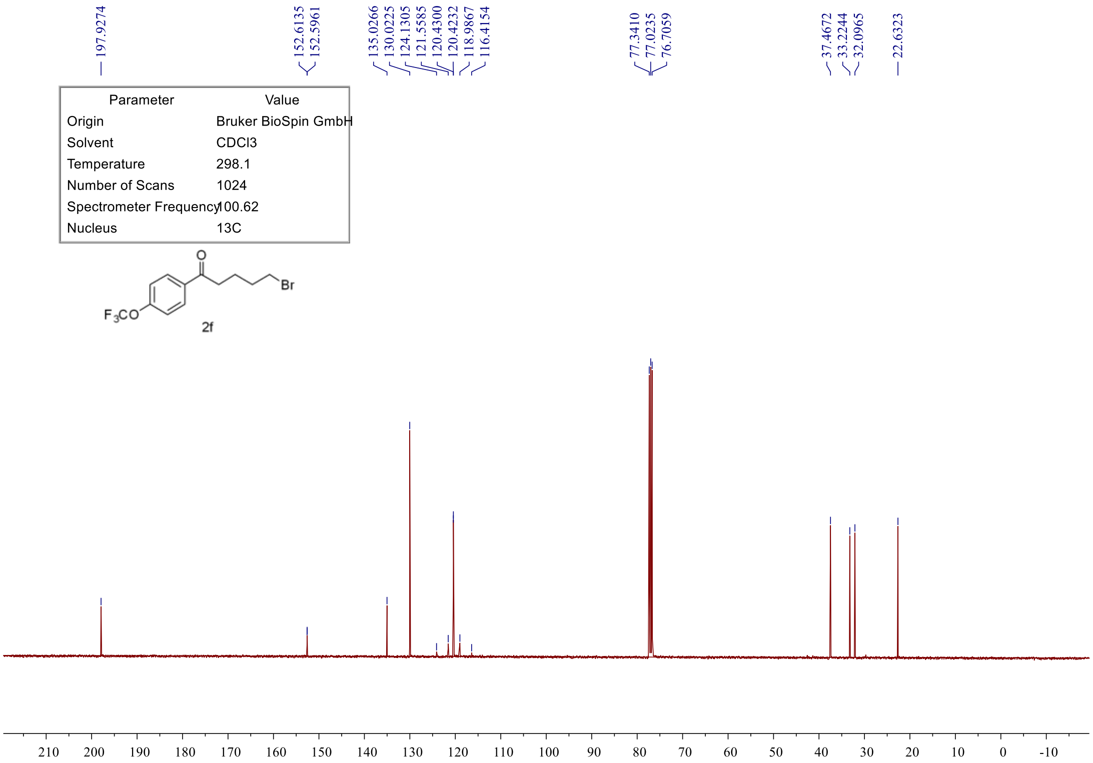

197.9274
152.6135
152.5961
135.0266
130.0225
124.1305
121.5585
120.4300
120.4232
118.9867
116.4154
77.3410
77.0235
76.7059
37.4672
33.2244
32.0965
22.6323
Parameter Value
Origin Bruker BioSpin GmbH
Solvent CDCl3
Temperature 298.1
Number of Scans 1024
Spectrometer Frequency 100.62
Nucleus 13C
O
Br
F3CO
2f
210 200 190 180 170 160 150 140 130 120 110 100 90 80 70 60 50 40 30 20 10 0 -10

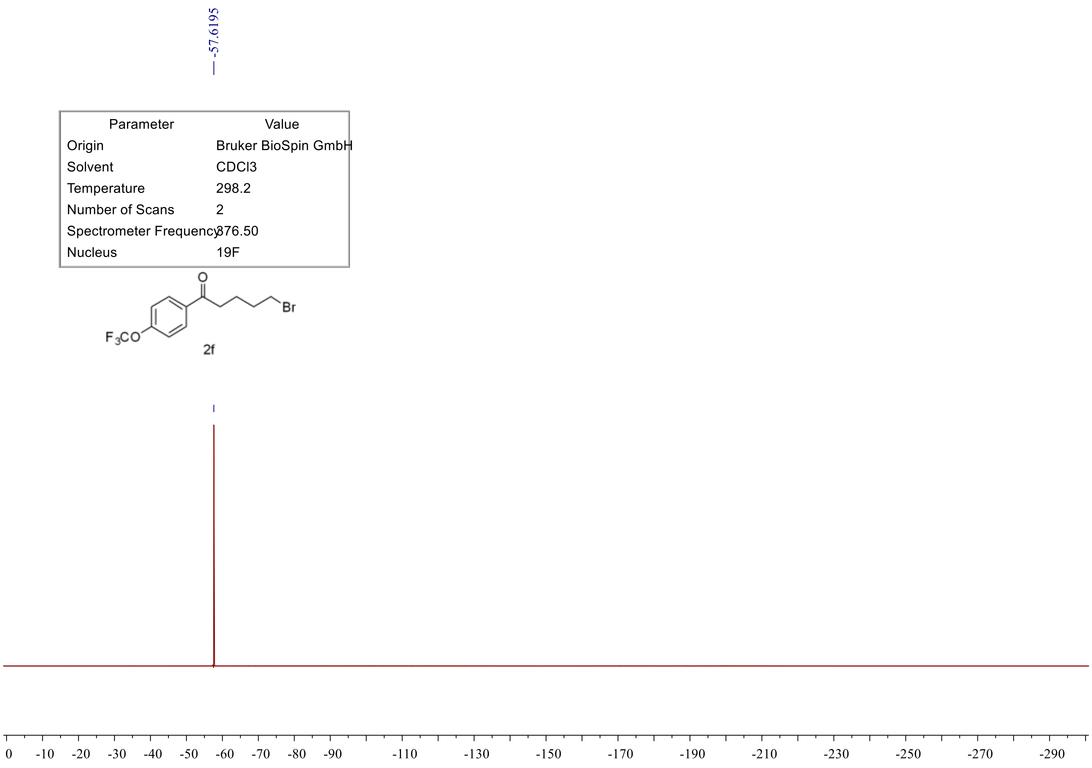
-57.6195
Parameter Value
Origin Bruker BioSpin GmbH
Solvent CDCl3
Temperature 298.2
Number of Scans 2
Spectrometer Frequency 376.50
Nucleus 19F
O
Br
F3CO
2f
0 -10 -20 -30 -40 -50 -60 -70 -80 -90 -110 -130 -150 -170 -190 -210 -230 -250 -270 -290

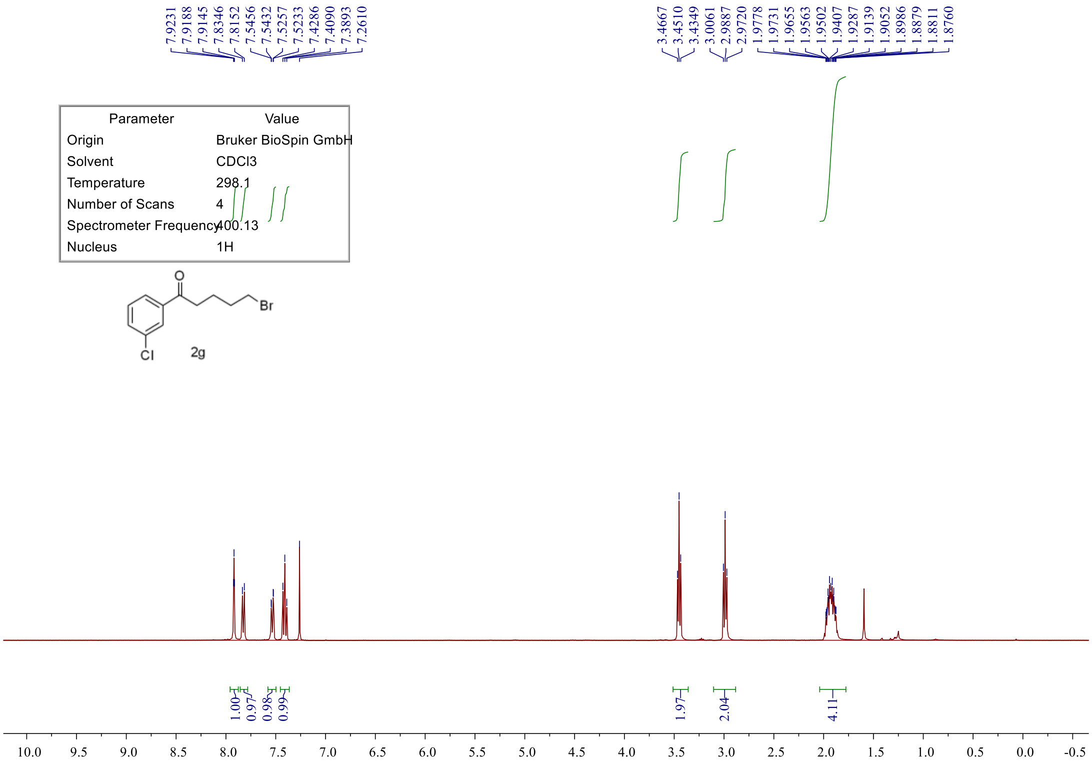
7.9231 7.9188 7.9145 7.8346 7.8152 7.5456 7.5432 7.5257 7.5233 7.4286 7.4090 7.3893 7.2610
3.4667 3.4510 3.4349 3.0061 2.9887 2.9720 1.9778 1.9731 1.9655 1.9563 1.9502 1.9407 1.9287 1.9139 1.9052 1.8986 1.8879 1.8811 1.8760
Parameter Value
Origin Bruker BioSpin GmbH
Solvent CDCl3
Temperature 298.1
Number of Scans 4
Spectrometer Frequency 400.13
Nucleus 1H
O
Br
Cl
2g
1.00 0.97 0.98 0.99 1.97 2.04 4.11
10.0 9.5 9.0 8.5 8.0 7.5 7.0 6.5 6.0 5.5 5.0 4.5 4.0 3.5 3.0 2.5 2.0 1.5 1.0 0.5 0.0 -0.5

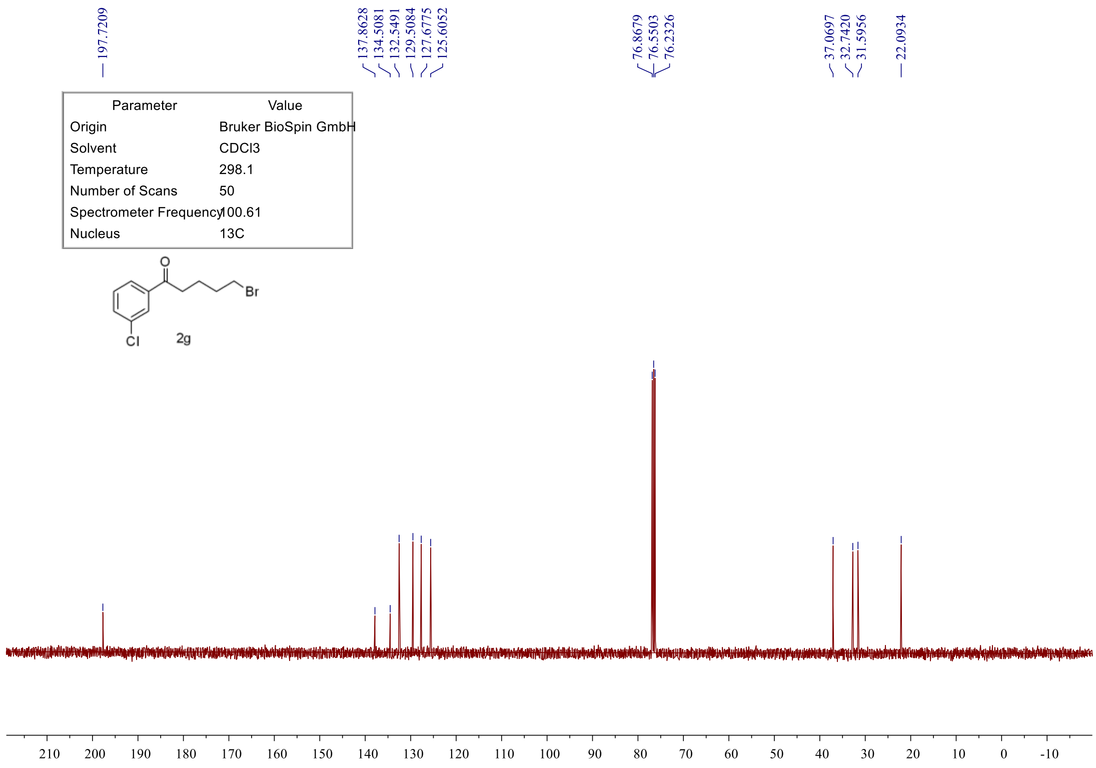
197.7209
137.8628
134.5081
132.5491
129.5084
127.6775
125.6052
76.8679
76.5503
76.2326
37.0697
32.7420
31.5956
22.0934
Parameter Value
Origin Bruker BioSpin GmbH
Solvent CDCl3
Temperature 298.1
Number of Scans 50
Spectrometer Frequency 100.61
Nucleus 13C
O
Br
Cl
2g
210 200 190 180 170 160 150 140 130 120 110 100 90 80 70 60 50 40 30 20 10 0 -10

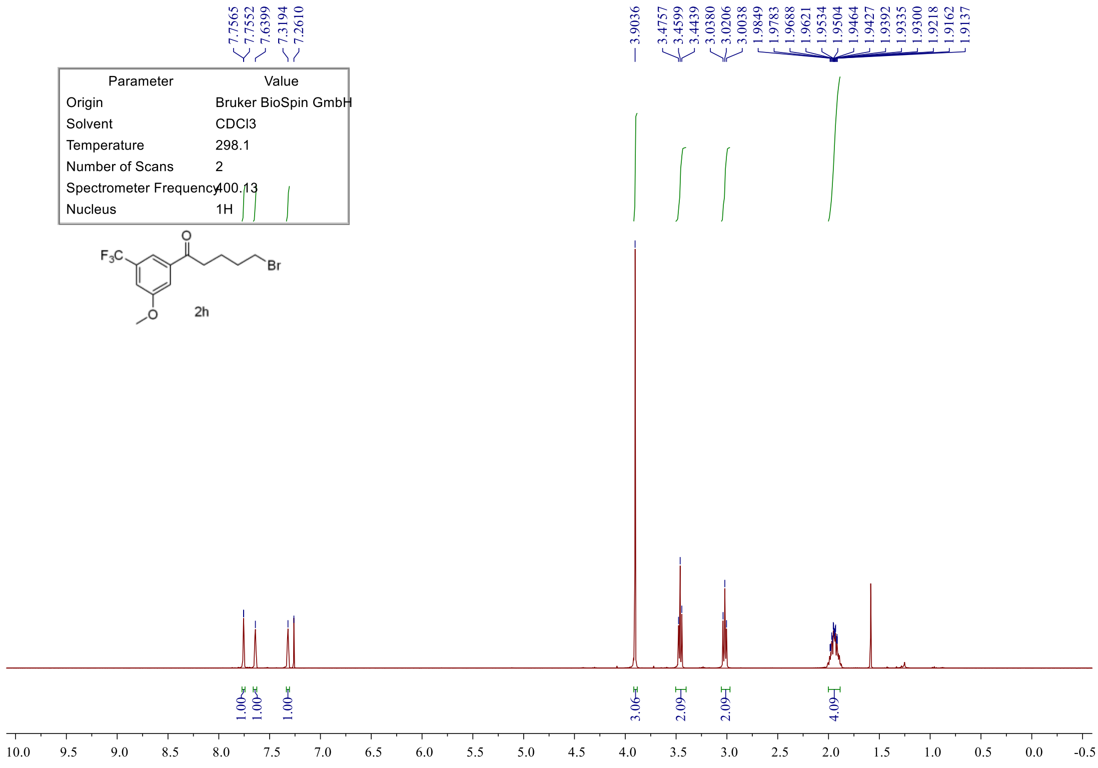
7.7565
7.7552
7.6399
7.3194
7.2610
3.9036
3.4757
3.4599
3.4439
3.0380
3.0206
3.0038
1.9849
1.9783
1.9688
1.9621
1.9534
1.9504
1.9464
1.9427
1.9392
1.9335
1.9300
1.9218
1.9162
1.9137
Parameter Value
Origin Bruker BioSpin GmbH
Solvent CDCl3
Temperature 298.1
Number of Scans 2
Spectrometer Frequency 400.13
Nucleus 1H
F3C
O
Br
O
2h
1.00
1.00
1.00
3.06
2.09
2.09
4.09
10.0 9.5 9.0 8.5 8.0 7.5 7.0 6.5 6.0 5.5 5.0 4.5 4.0 3.5 3.0 2.5 2.0 1.5 1.0 0.5 0.0 -0.5

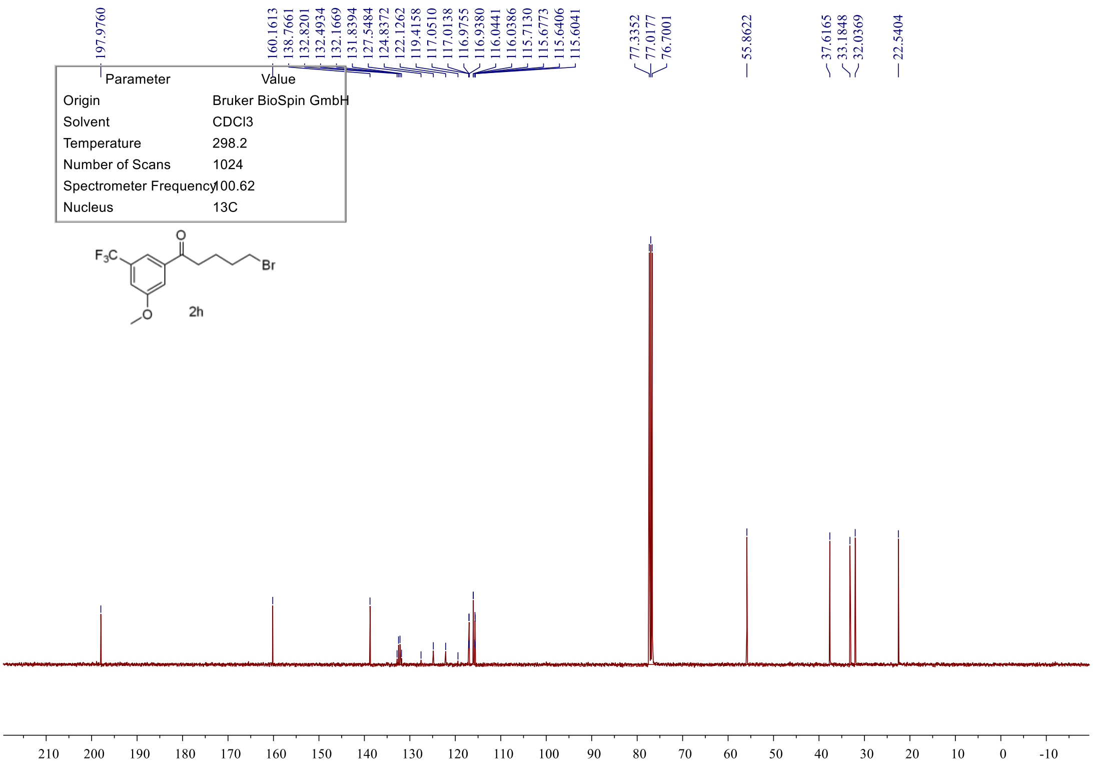
197.9760
160.1613 138.7661 132.8201 132.4934 132.1669 131.8394 127.5484 124.8372 122.1262 119.4158 117.0510 117.0138 116.9755 116.9380 116.0441 116.0386 115.7130 115.6773 115.6406 115.6041
77.3352 77.0177 76.7001
55.8622
37.6165 33.1848 32.0369
22.5404
Parameter | Value
Origin | Bruker BioSpin GmbH
Solvent | CDCl3
Temperature | 298.2
Number of Scans | 1024
Spectrometer Frequency | 100.62
Nucleus | 13C
F_3C
O
Br
2h
210 200 190 180 170 160 150 140 130 120 110 100 90 80 70 60 50 40 30 20 10 0 -10

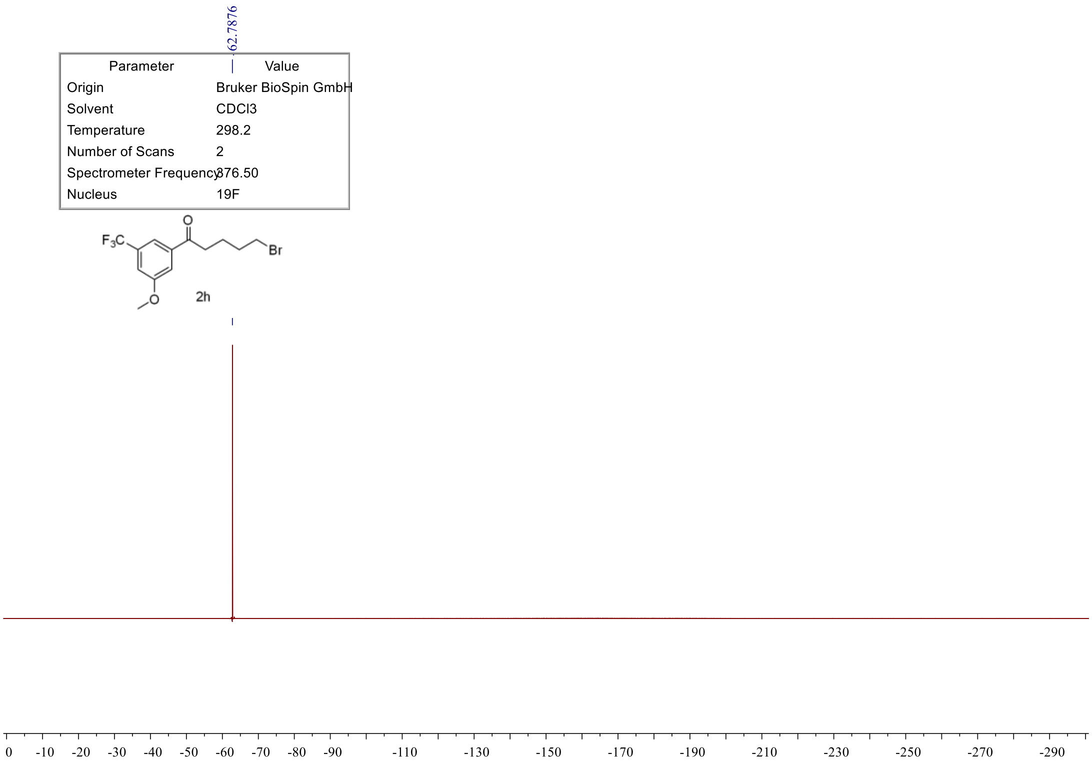
-62.7876
Parameter | Value
Origin | Bruker BioSpin GmbH
Solvent | CDCl3
Temperature | 298.2
Number of Scans | 2
Spectrometer Frequency | 376.50
Nucleus | 19F
F_3C
O
Br
2h
0 -10 -20 -30 -40 -50 -60 -70 -80 -90 -110 -130 -150 -170 -190 -210 -230 -250 -270 -290

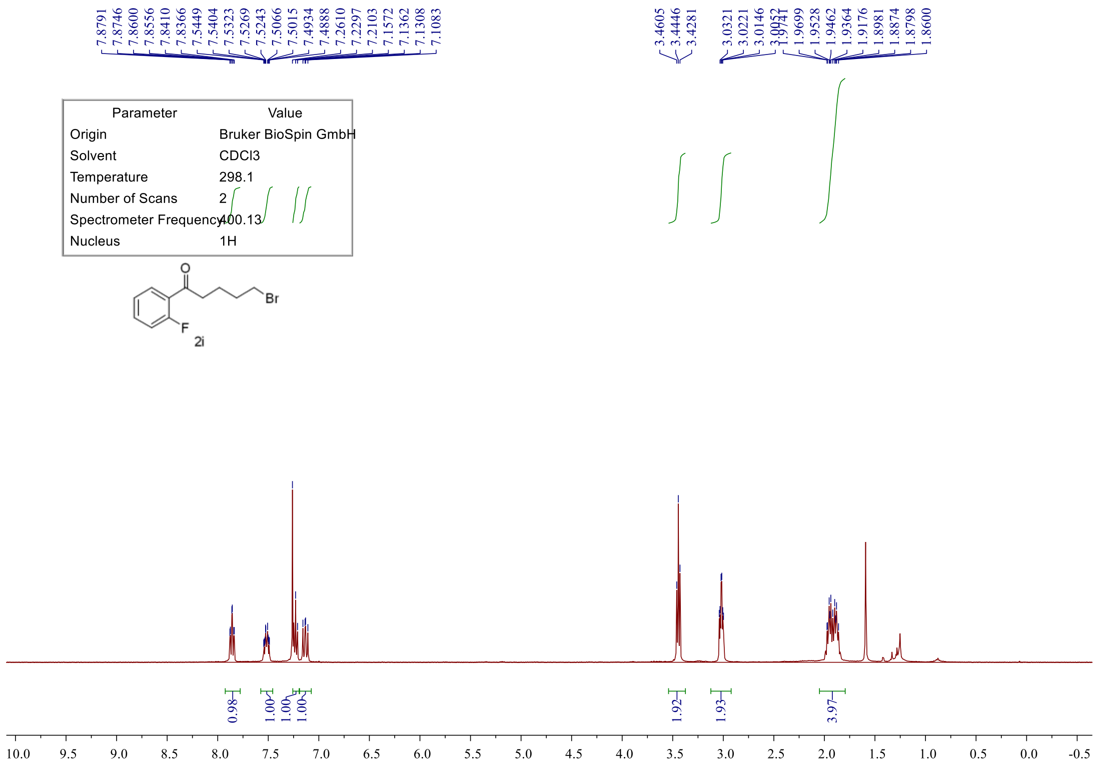
Parameter Value
Origin Bruker BioSpin GmbH
Solvent CDCl3
Temperature 298.1
Number of Scans 2
Spectrometer Frequency 400.13
Nucleus 1H
2i

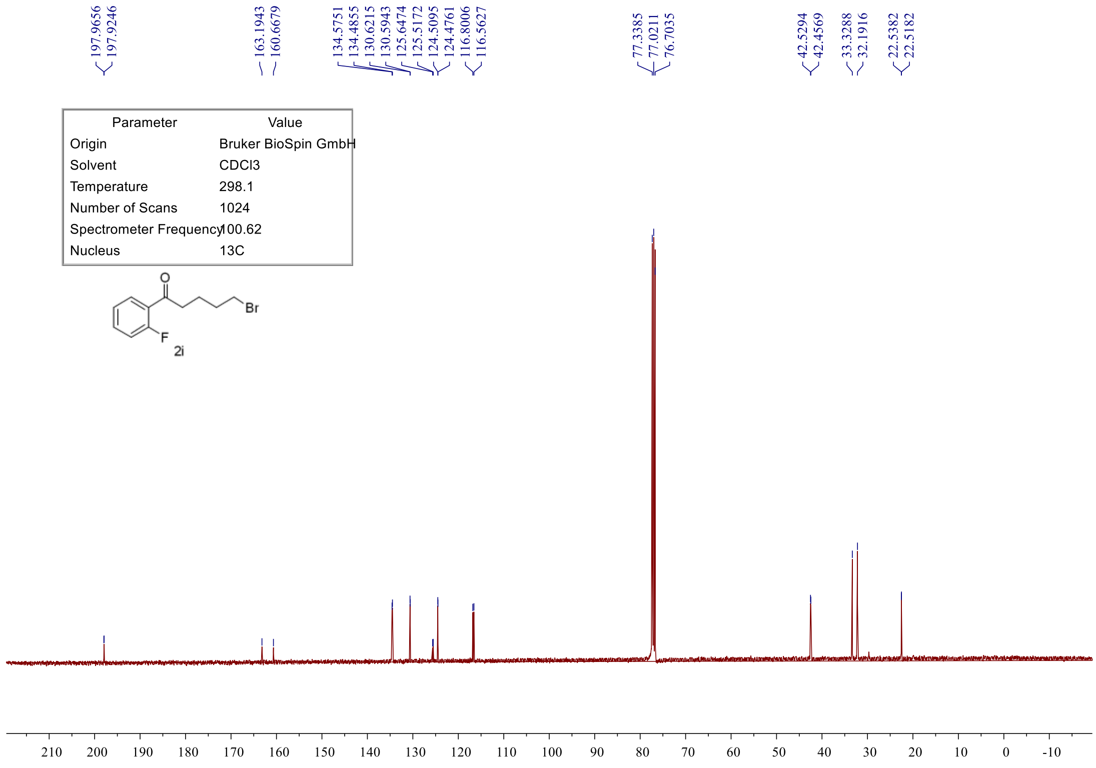
Parameter Value
Origin Bruker BioSpin GmbH
Solvent CDCl3
Temperature 298.1
Number of Scans 1024
Spectrometer Frequency 100.62
Nucleus 13C
2i

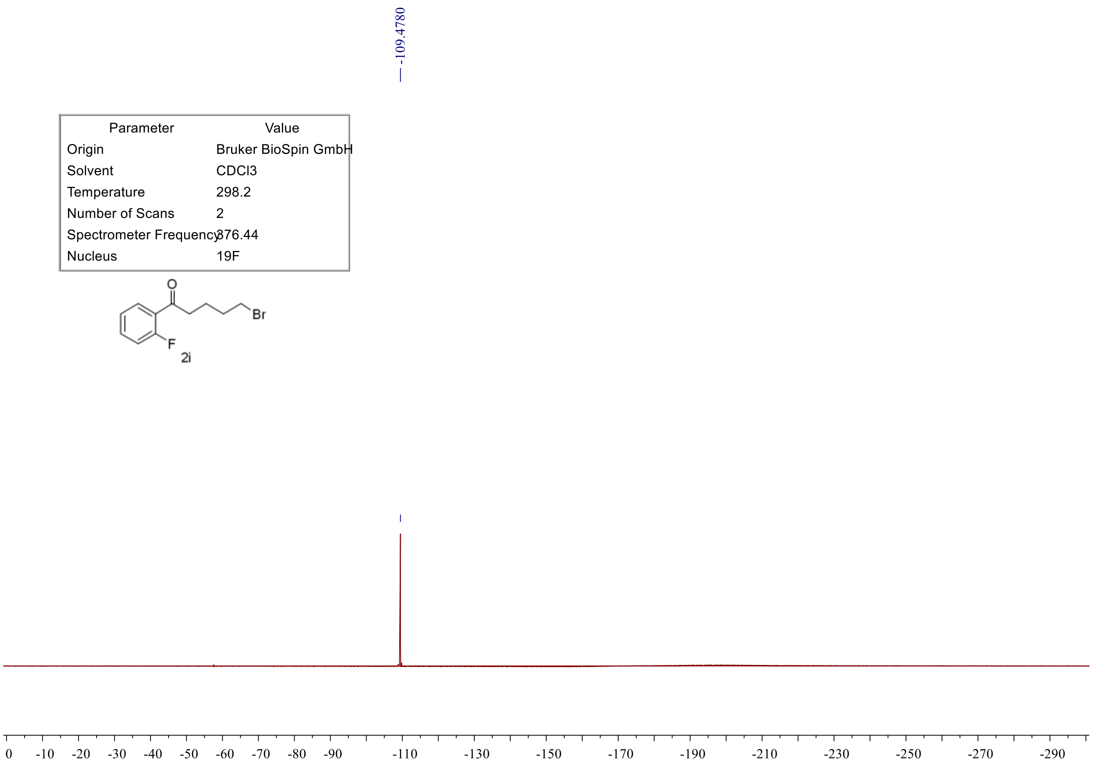
-109.4780
Parameter Value
Origin Bruker BioSpin GmbH
Solvent CDCl3
Temperature 298.2
Number of Scans 2
Spectrometer Frequency 376.44
Nucleus 19F
O
Br
F
2i
0 -10 -20 -30 -40 -50 -60 -70 -80 -90 -110 -130 -150 -170 -190 -210 -230 -250 -270 -290

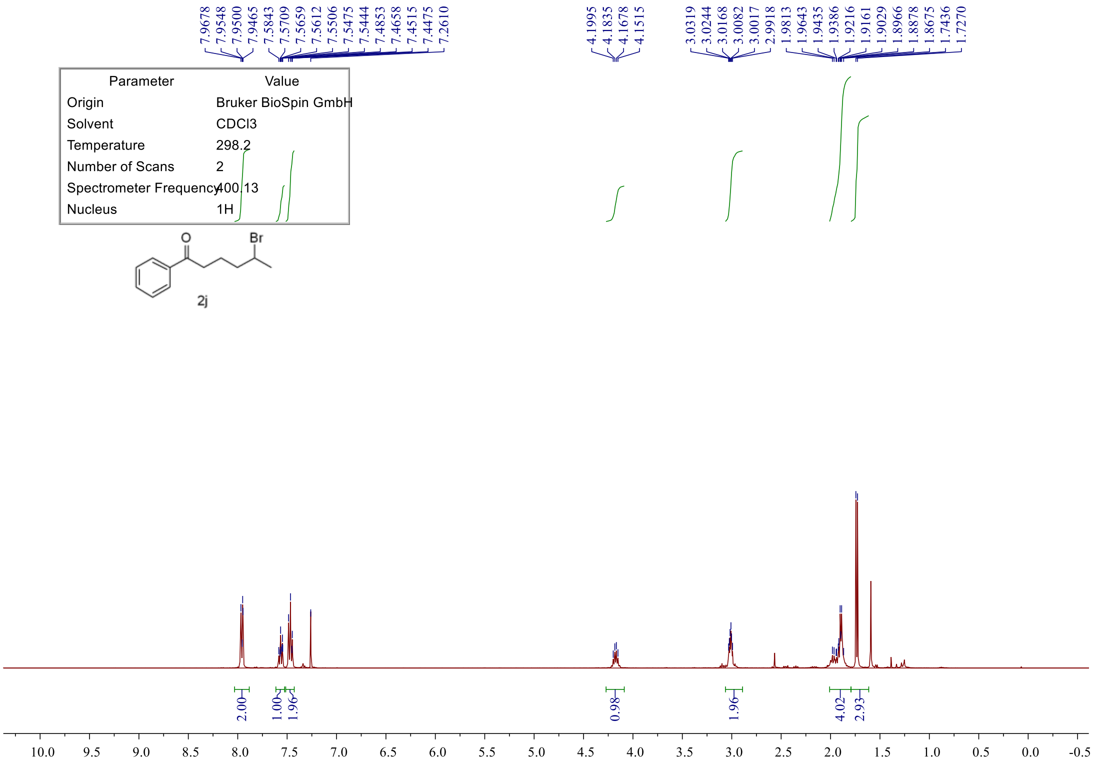
7.9678 7.9548 7.9500 7.9465 7.5843 7.5709 7.5659 7.5612 7.5506 7.5475 7.5444 7.4853 7.4658 7.4515 7.4475 7.2610
4.1995 4.1835 4.1678 4.1515
3.0319 3.0244 3.0168 3.0082 3.0017 2.9918 1.9813 1.9643 1.9435 1.9386 1.9216 1.9161 1.9029 1.8966 1.8878 1.8675 1.7436 1.7270
Parameter Value
Origin Bruker BioSpin GmbH
Solvent CDCl3
Temperature 298.2
Number of Scans 2
Spectrometer Frequency 400.13
Nucleus 1H
O
Br
2j
2.00 1.00 1.96 0.98 1.96 4.02 2.93
10.0 9.5 9.0 8.5 8.0 7.5 7.0 6.5 6.0 5.5 5.0 4.5 4.0 3.5 3.0 2.5 2.0 1.5 1.0 0.5 0.0 -0.5

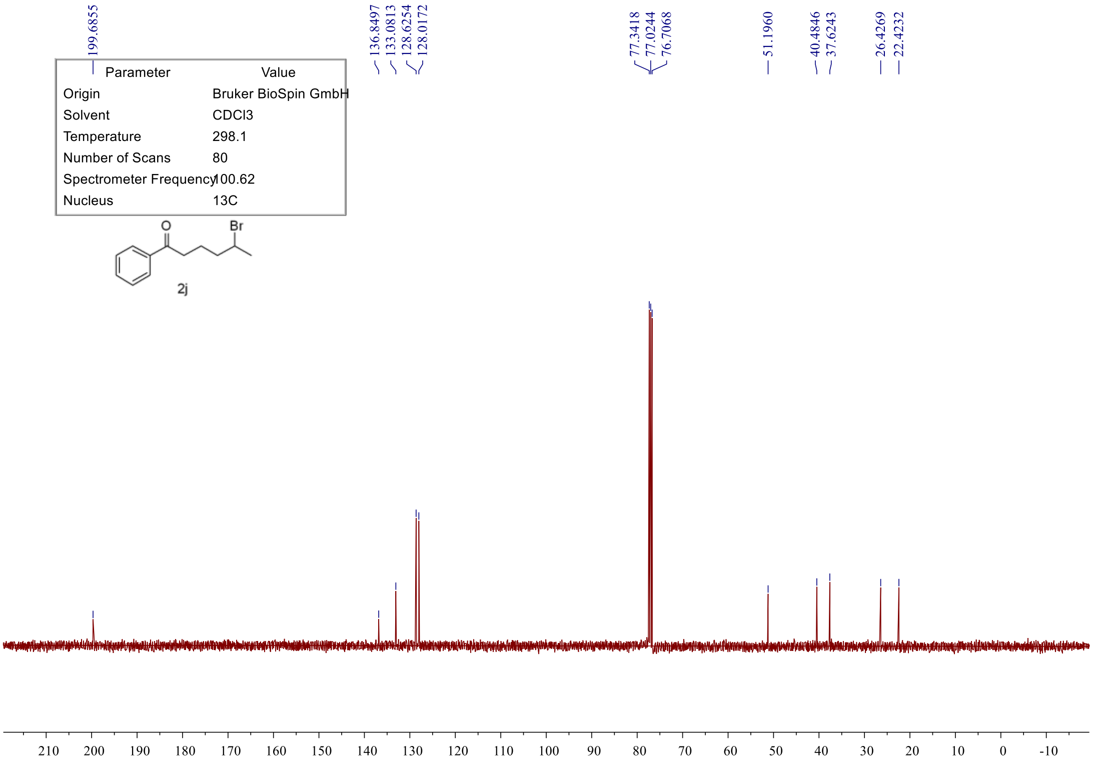

Parameter	Value
Origin	Bruker BioSpin GmbH
Solvent	CDCl3
Temperature	298.1
Number of Scans	80
Spectrometer Frequency	100.62
Nucleus	13C

2j

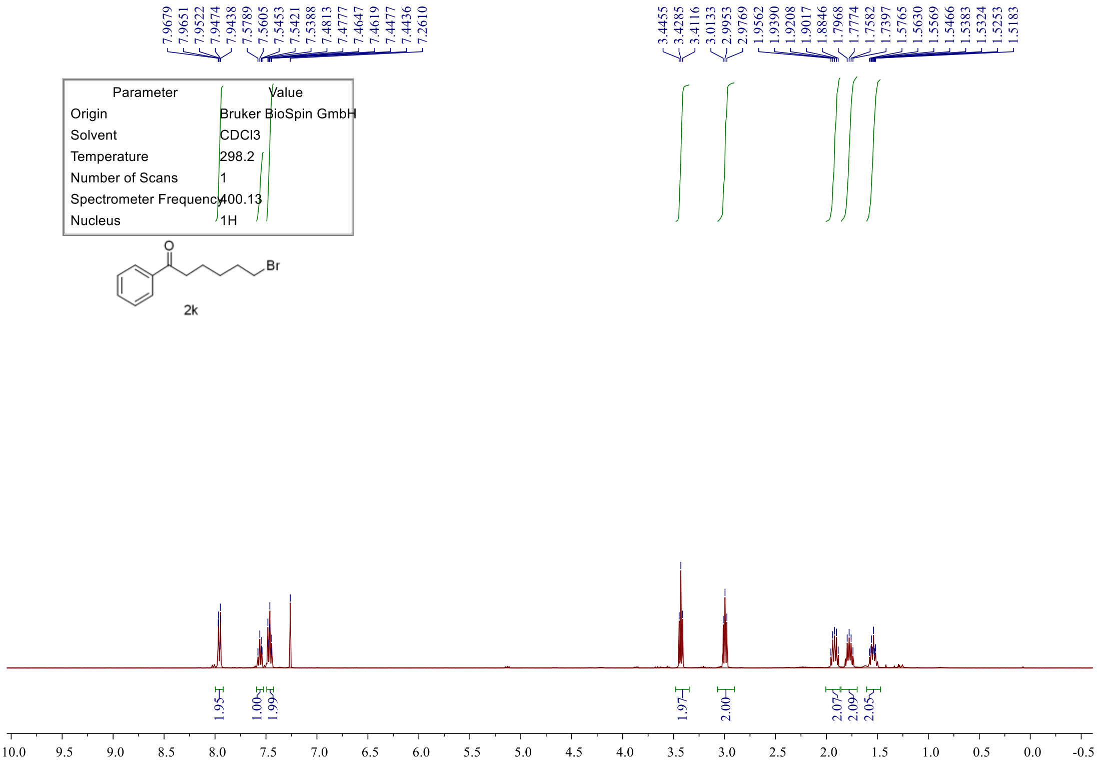

Parameter	Value
Origin	Bruker BioSpin GmbH
Solvent	CDCl3
Temperature	298.2
Number of Scans	1
Spectrometer Frequency	400.13
Nucleus	1H

2k

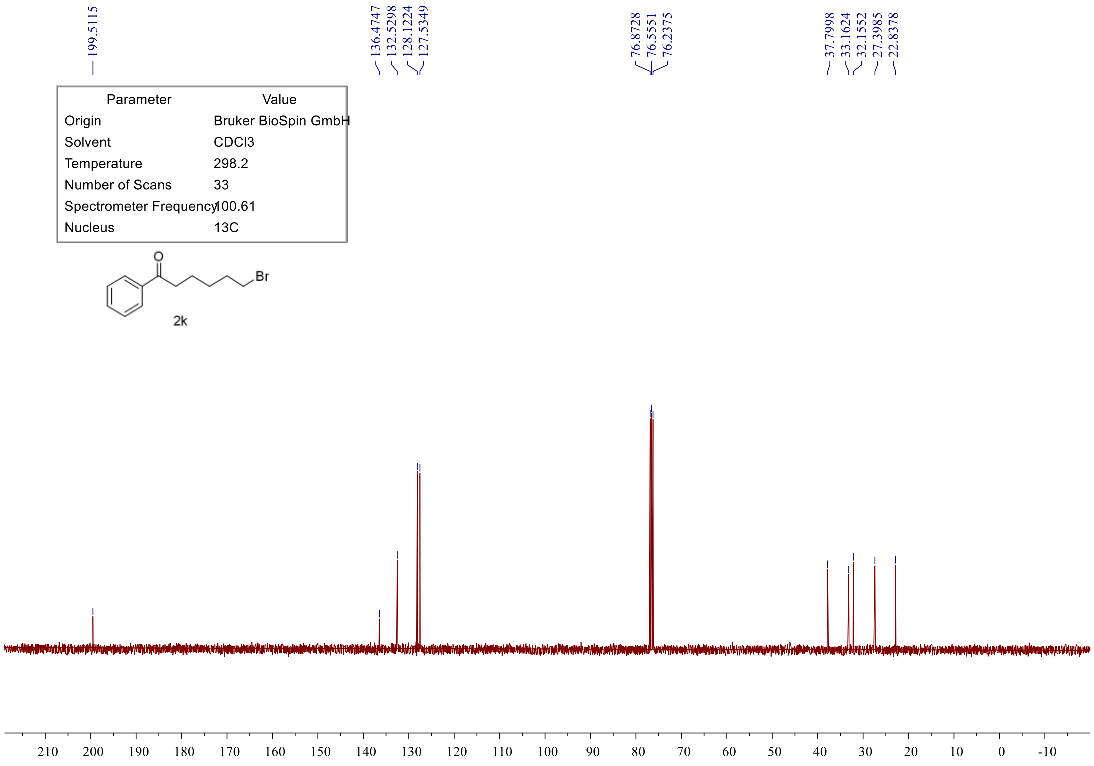

Parameter	Value
Origin	Bruker BioSpin GmbH
Solvent	CDCl3
Temperature	298.2
Number of Scans	33
Spectrometer Frequency	100.61
Nucleus	13C

2k

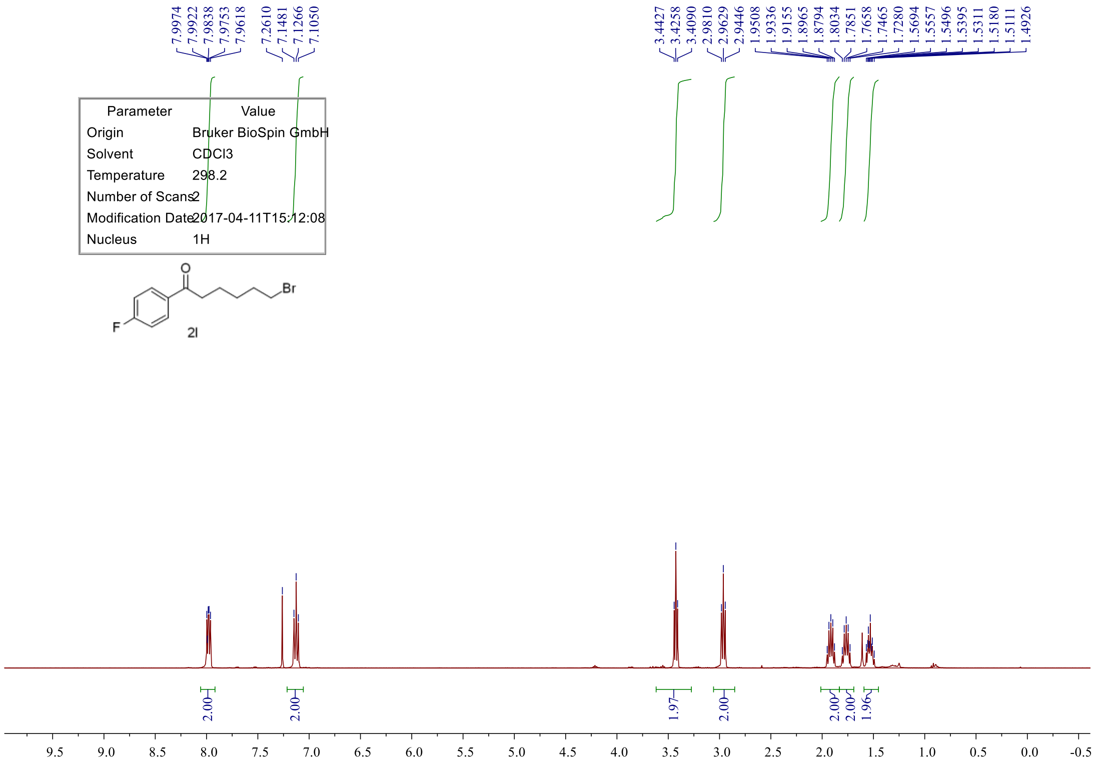

Parameter	Value
Origin	Bruker BioSpin GmbH
Solvent	CDCl3
Temperature	298.2
Number of Scans	2
Modification Date	2017-04-11T15:12:08
Nucleus	1H

2l

Parameter	Value
Origin	Bruker BioSpin GmbH
Solvent	CDCl3
Temperature	298.2
Number of Scans	228
Spectrometer Frequency	100.61
Nucleus	13C

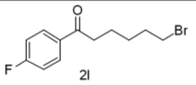

2l

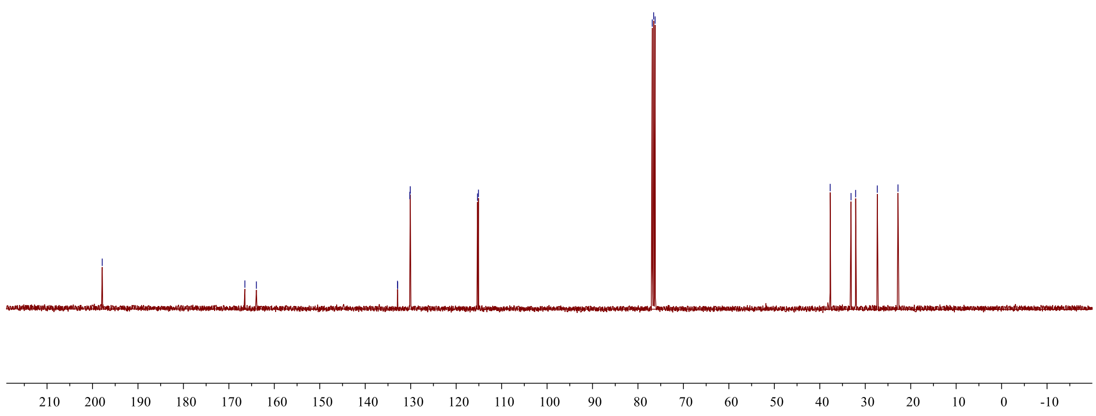

Parameter	Value
Origin	Bruker BioSpin GmbH
Solvent	CDCl3
Temperature	298.2
Number of Scans	1
Spectrometer Frequency	376.50
Nucleus	19F

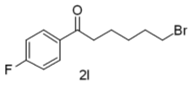

2l

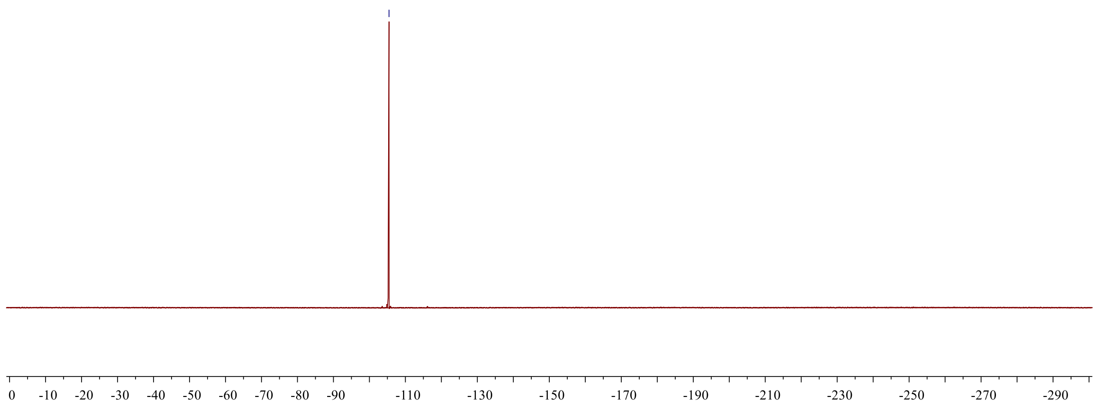

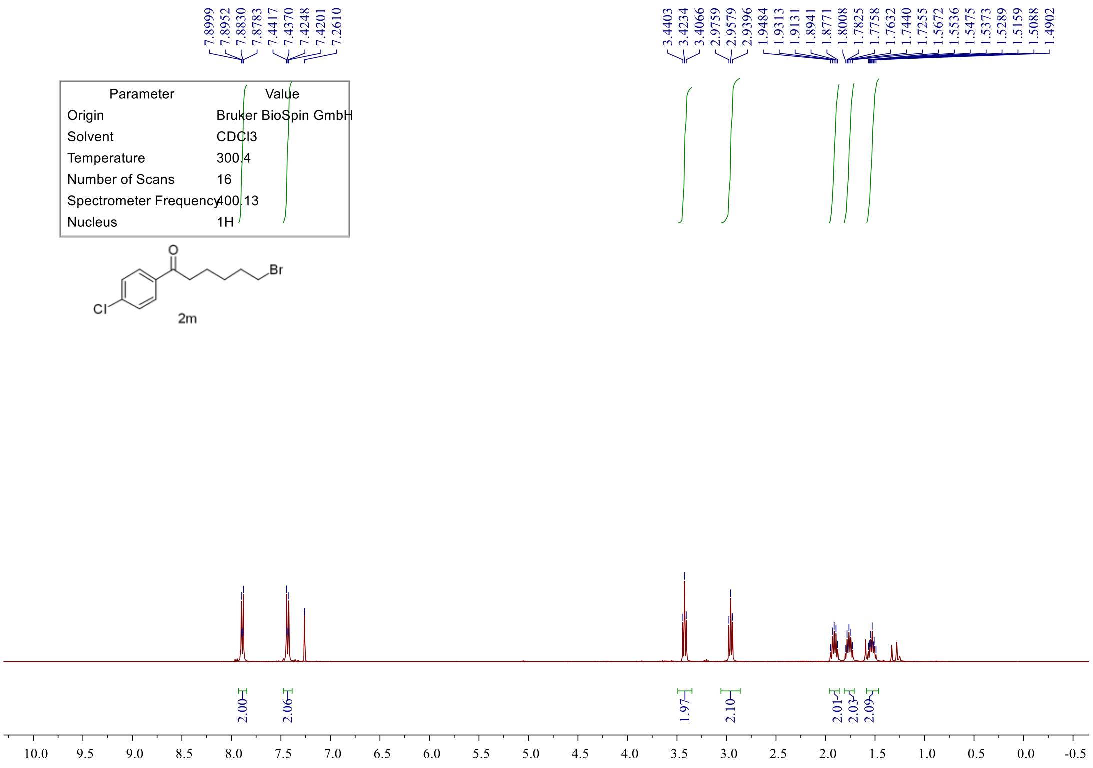
7.8999
7.8952
7.8830
7.8783
7.4417
7.4370
7.4248
7.4201
7.2610
3.4403
3.4234
3.4066
2.9759
2.9579
2.9396
1.9484
1.9313
1.9131
1.8941
1.8771
1.8008
1.7825
1.7758
1.7632
1.7440
1.7255
1.5672
1.5536
1.5475
1.5373
1.5289
1.5159
1.5088
1.4902
Parameter Value
Origin Bruker BioSpin GmbH
Solvent CDCl3
Temperature 300.4
Number of Scans 16
Spectrometer Frequency 400.13
Nucleus 1H
O
Br
Cl
2m
2.00
2.06
1.97
2.10
2.01
2.03
2.09
10.0 9.5 9.0 8.5 8.0 7.5 7.0 6.5 6.0 5.5 5.0 4.5 4.0 3.5 3.0 2.5 2.0 1.5 1.0 0.5 0.0 -0.5

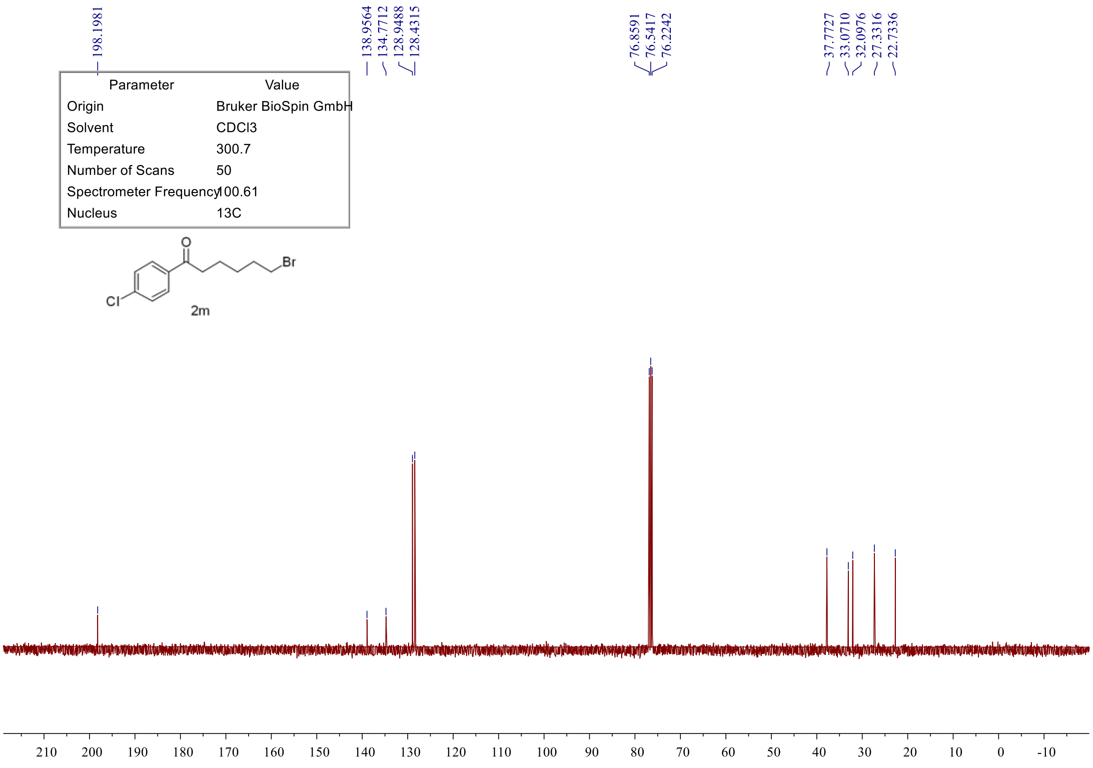
198.1981
138.9564
134.7712
128.9488
128.4315
76.8591
76.5417
76.2242
37.7727
33.0710
32.0976
27.3316
22.7336
Parameter Value
Origin Bruker BioSpin GmbH
Solvent CDCl3
Temperature 300.7
Number of Scans 50
Spectrometer Frequency 100.61
Nucleus 13C
O
Br
Cl
2m
210 200 190 180 170 160 150 140 130 120 110 100 90 80 70 60 50 40 30 20 10 0 -10

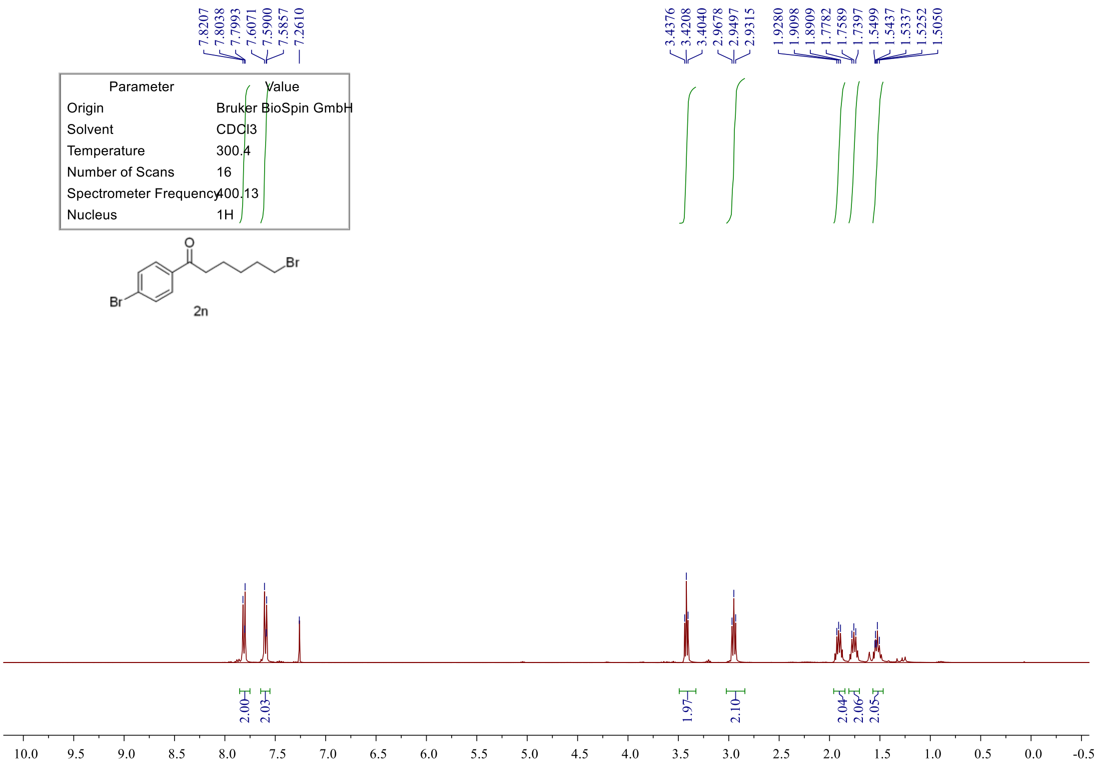

Parameter	Value
Origin	Bruker BioSpin GmbH
Solvent	CDCl3
Temperature	300.4
Number of Scans	16
Spectrometer Frequency	400.13
Nucleus	1H

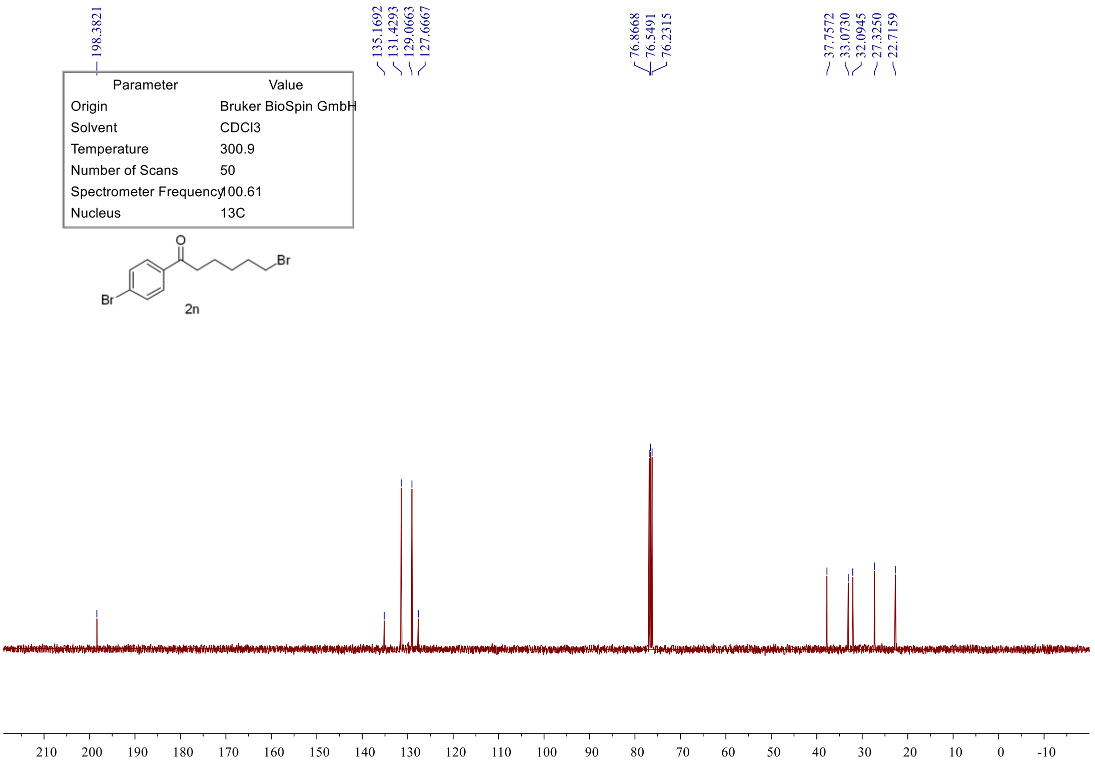

Parameter	Value
Origin	Bruker BioSpin GmbH
Solvent	CDCl3
Temperature	300.9
Number of Scans	50
Spectrometer Frequency	100.61
Nucleus	13C

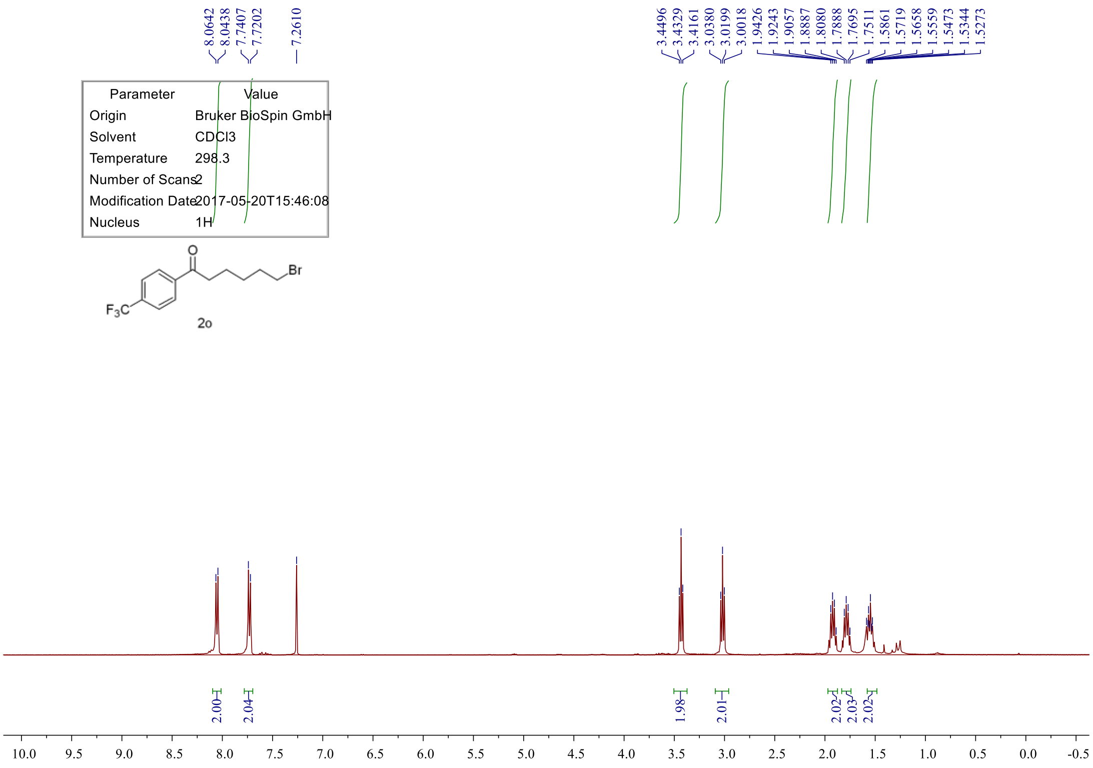
8.0642
8.0438
7.7407
7.7202
7.2610
3.4496
3.4329
3.4161
3.0380
3.0199
3.0018
1.9426
1.9243
1.9057
1.8887
1.8080
1.7888
1.7695
1.7511
1.5861
1.5719
1.5658
1.5559
1.5473
1.5344
1.5273
Parameter
Value
Origin
Bruker BioSpin GmbH
Solvent
CDCl3
Temperature
298.3
Number of Scans
2
Modification Date
2017-05-20T15:46:08
Nucleus
1H
O
Br
F_3C
2o
2.00
2.04
1.98
2.01
2.02
2.03
2.02
10.0 9.5 9.0 8.5 8.0 7.5 7.0 6.5 6.0 5.5 5.0 4.5 4.0 3.5 3.0 2.5 2.0 1.5 1.0 0.5 0.0 -0.5

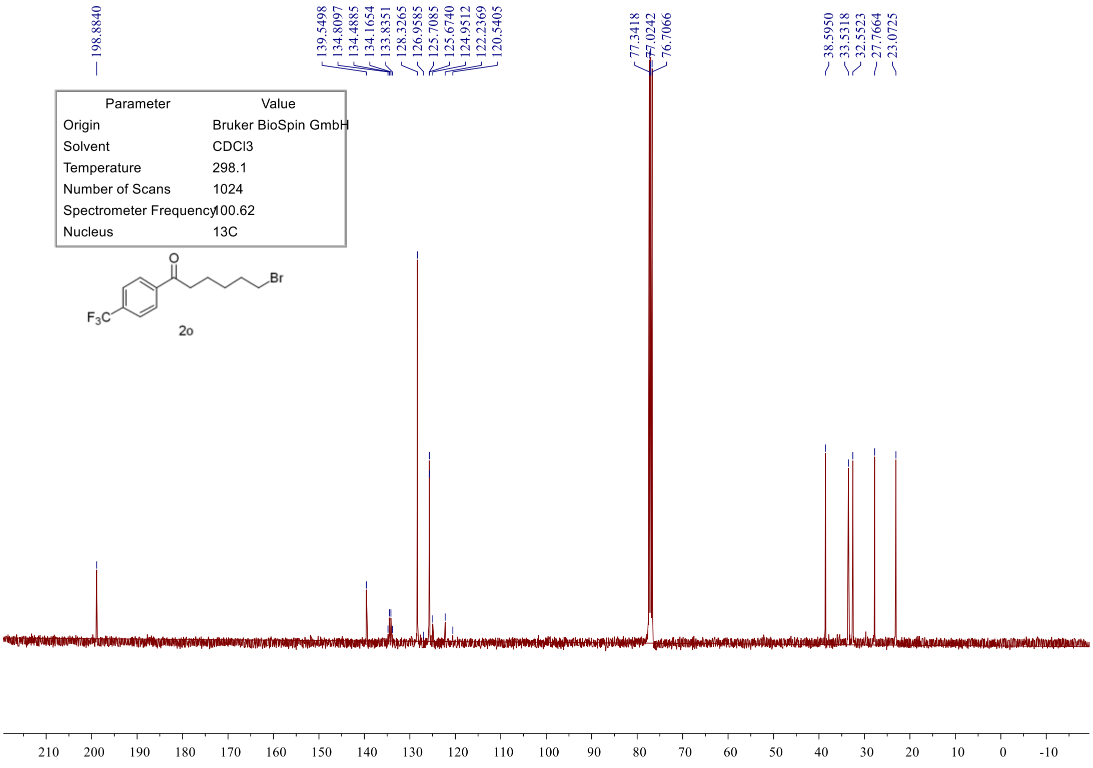
198.8840
139.5498
134.8097
134.4885
134.1654
133.8351
128.3265
126.9585
125.7085
125.6740
124.9512
122.2369
120.5405
77.3418
77.0242
76.7066
38.5950
33.5318
32.5523
27.7664
23.0725
Parameter
Value
Origin
Bruker BioSpin GmbH
Solvent
CDCl3
Temperature
298.1
Number of Scans
1024
Spectrometer Frequency
100.62
Nucleus
13C
O
Br
F_3C
2o
210 200 190 180 170 160 150 140 130 120 110 100 90 80 70 60 50 40 30 20 10 0 -10

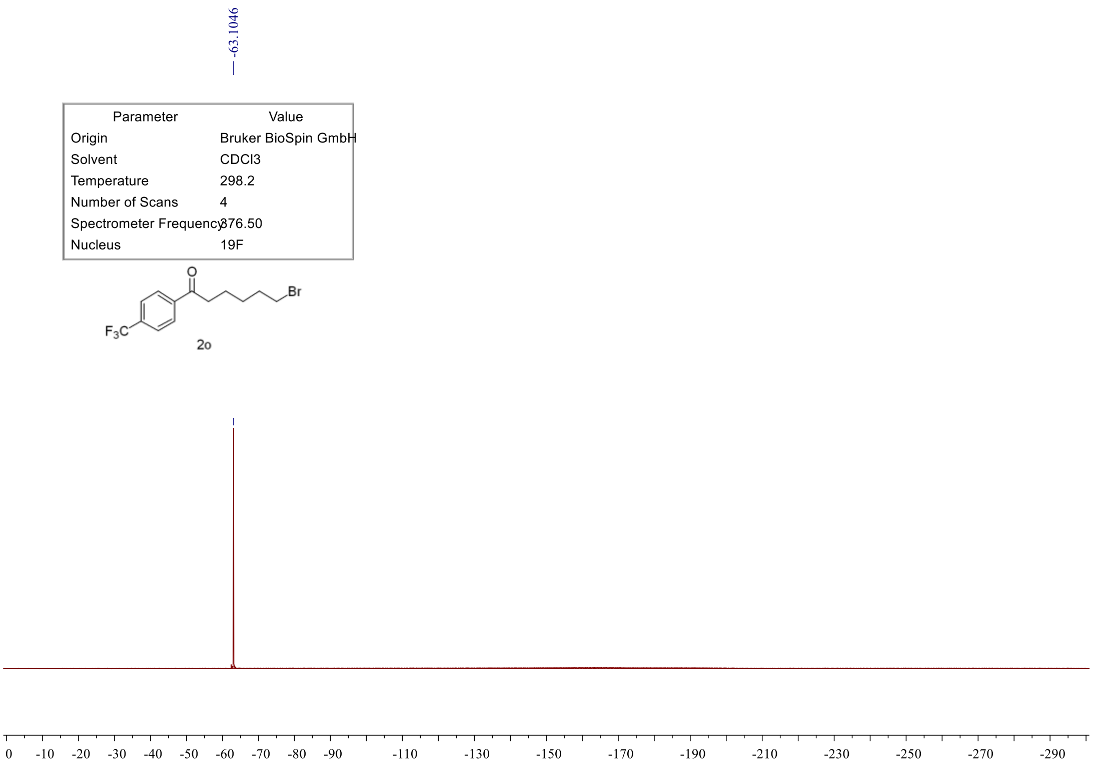
-63.1046
Parameter Value
Origin Bruker BioSpin GmbH
Solvent CDCl3
Temperature 298.2
Number of Scans 4
Spectrometer Frequency 376.50
Nucleus 19F
O
Br
F_3C
2o
0 -10 -20 -30 -40 -50 -60 -70 -80 -90 -110 -130 -150 -170 -190 -210 -230 -250 -270 -290

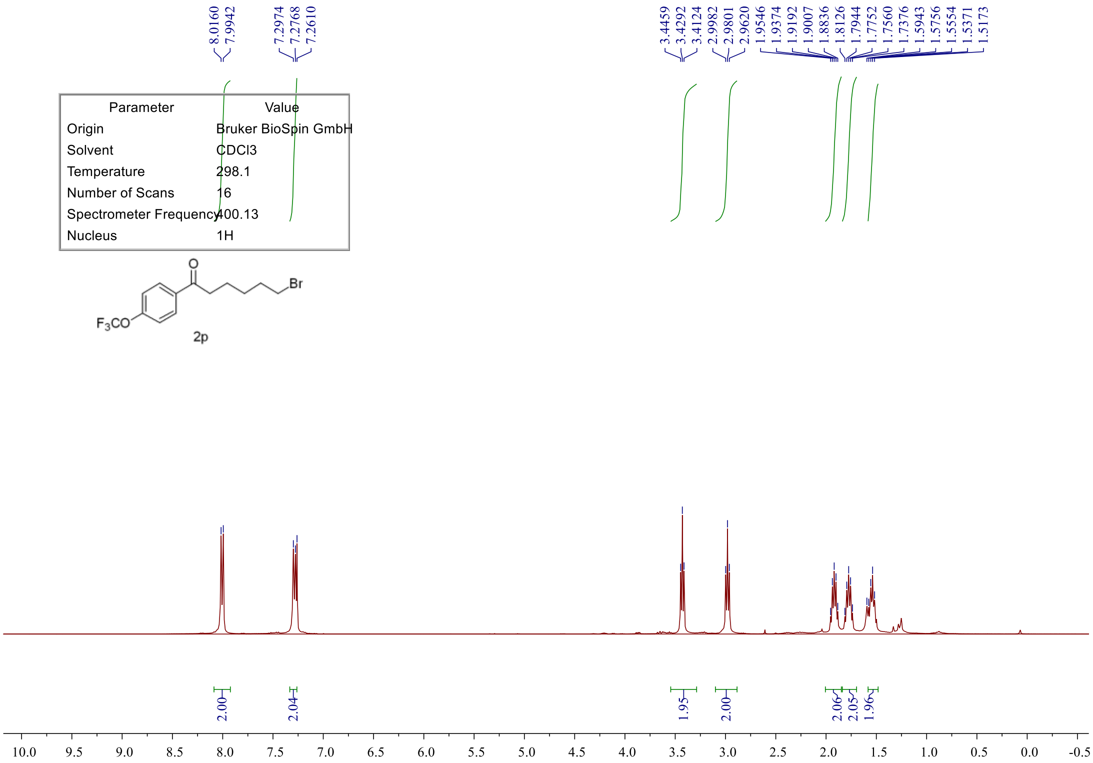
8.0160
7.9942
7.2974
7.2768
7.2610
3.4459
3.4292
3.4124
2.9982
2.9801
2.9620
1.9546
1.9374
1.9192
1.9007
1.8836
1.8126
1.7944
1.7752
1.7560
1.7376
1.5943
1.5756
1.5554
1.5371
1.5173
Parameter Value
Origin Bruker BioSpin GmbH
Solvent CDCl3
Temperature 298.1
Number of Scans 16
Spectrometer Frequency 400.13
Nucleus 1H
O
Br
F_3CO
2p
2.00
2.04
1.95
2.00
2.06
2.05
1.96
10.0 9.5 9.0 8.5 8.0 7.5 7.0 6.5 6.0 5.5 5.0 4.5 4.0 3.5 3.0 2.5 2.0 1.5 1.0 0.5 0.0 -0.5

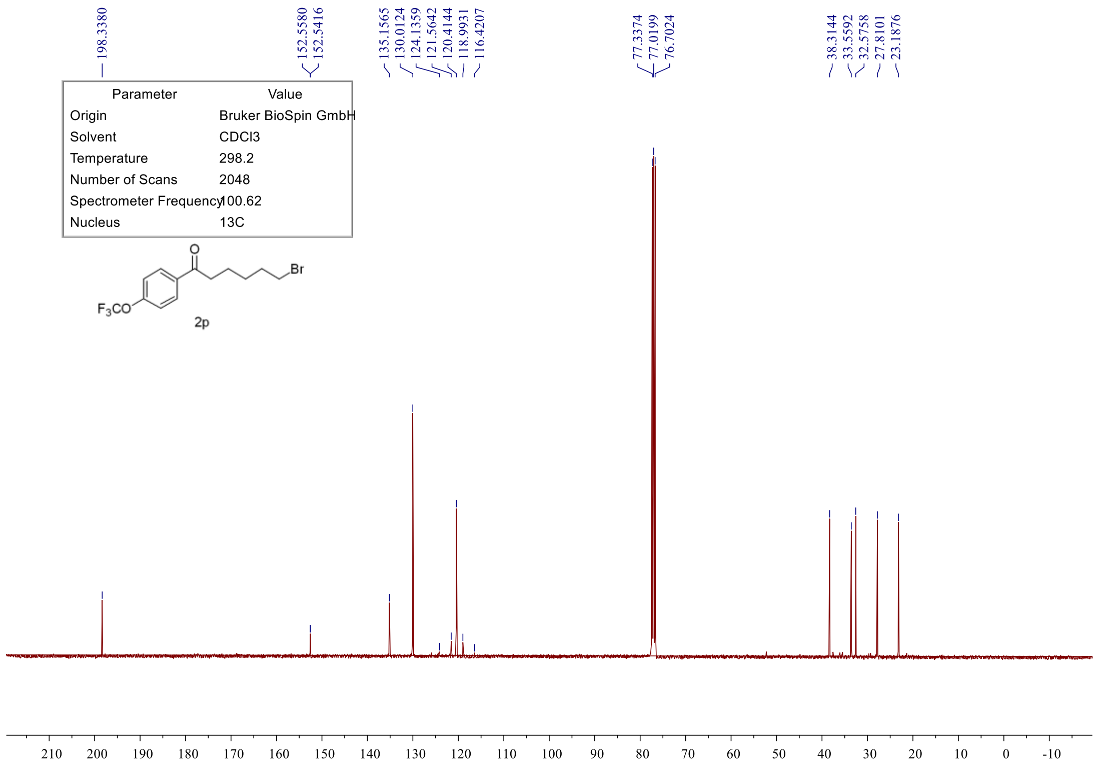
198.3380
152.5580
152.5416
135.1565
130.0124
124.1359
121.5642
120.4144
118.9931
116.4207
77.3374
77.0199
76.7024
38.3144
33.5592
32.5758
27.8101
23.1876
Parameter Value
Origin Bruker BioSpin GmbH
Solvent CDCl3
Temperature 298.2
Number of Scans 2048
Spectrometer Frequency 100.62
Nucleus 13C
O
Br
F3CO
2p
210 200 190 180 170 160 150 140 130 120 110 100 90 80 70 60 50 40 30 20 10 0 -10

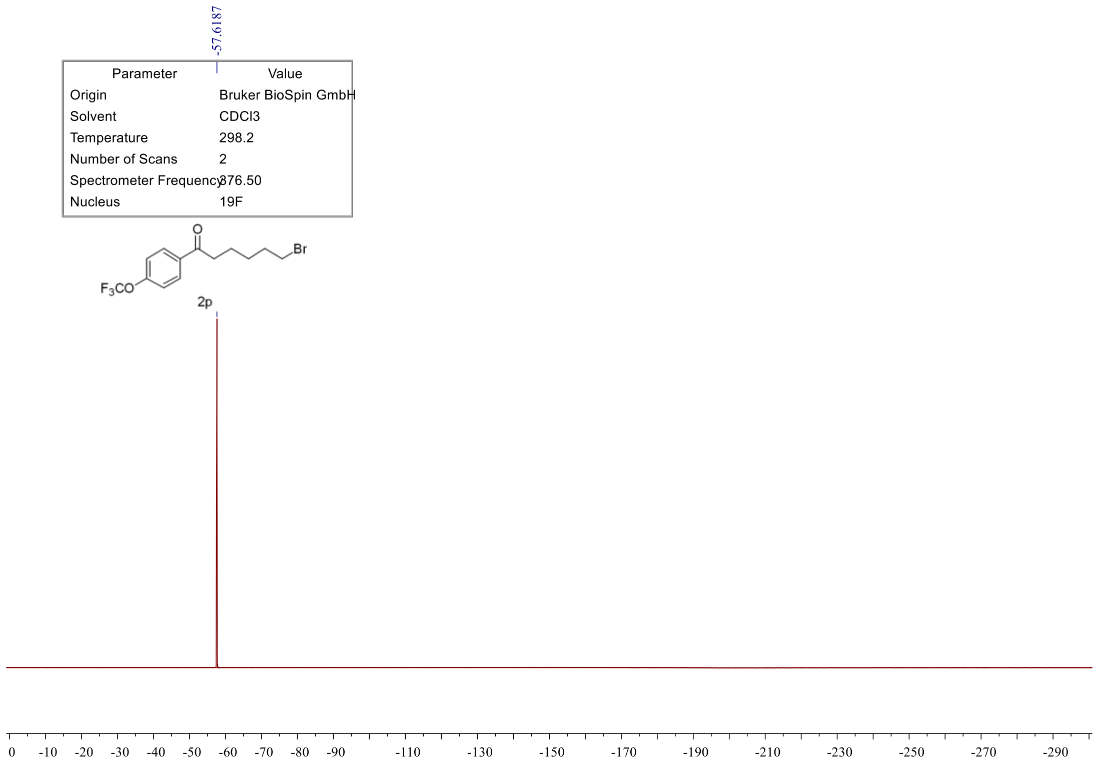
-57.6187
Parameter Value
Origin Bruker BioSpin GmbH
Solvent CDCl3
Temperature 298.2
Number of Scans 2
Spectrometer Frequency 376.50
Nucleus 19F
O
Br
F3CO
2p
0 -10 -20 -30 -40 -50 -60 -70 -80 -90 -110 -130 -150 -170 -190 -210 -230 -250 -270 -290

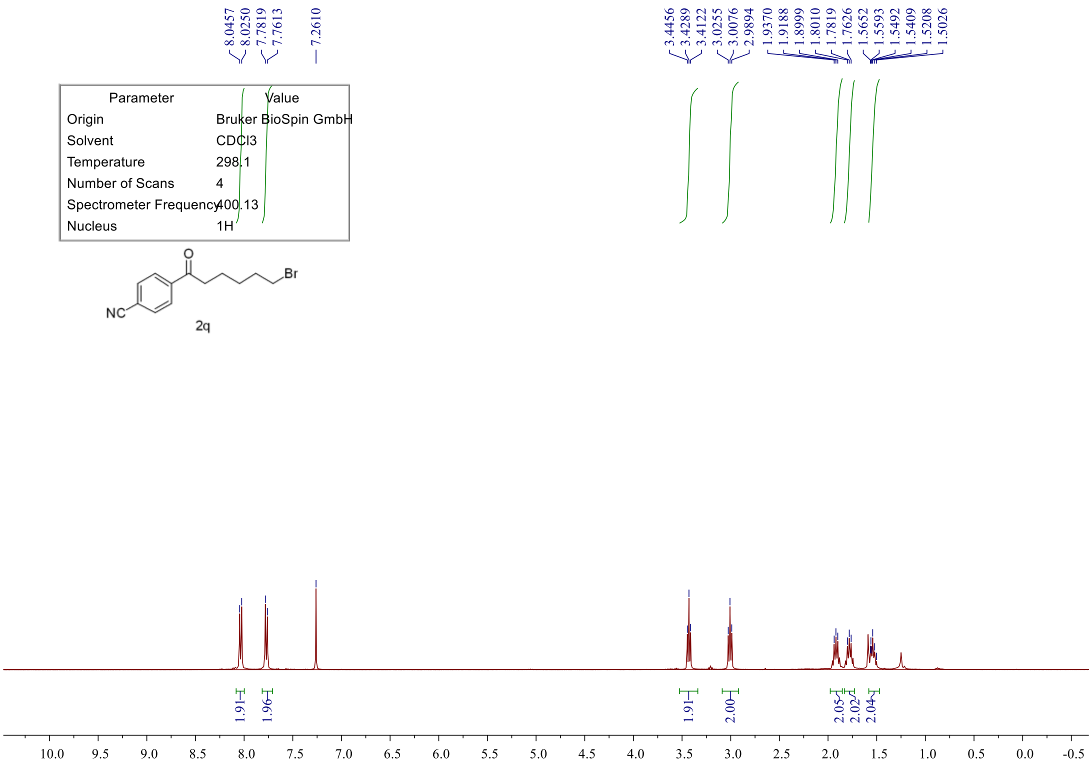

Parameter	Value
Origin	Bruker BioSpin GmbH
Solvent	CDCl3
Temperature	298.1
Number of Scans	4
Spectrometer Frequency	400.13
Nucleus	1H

2q

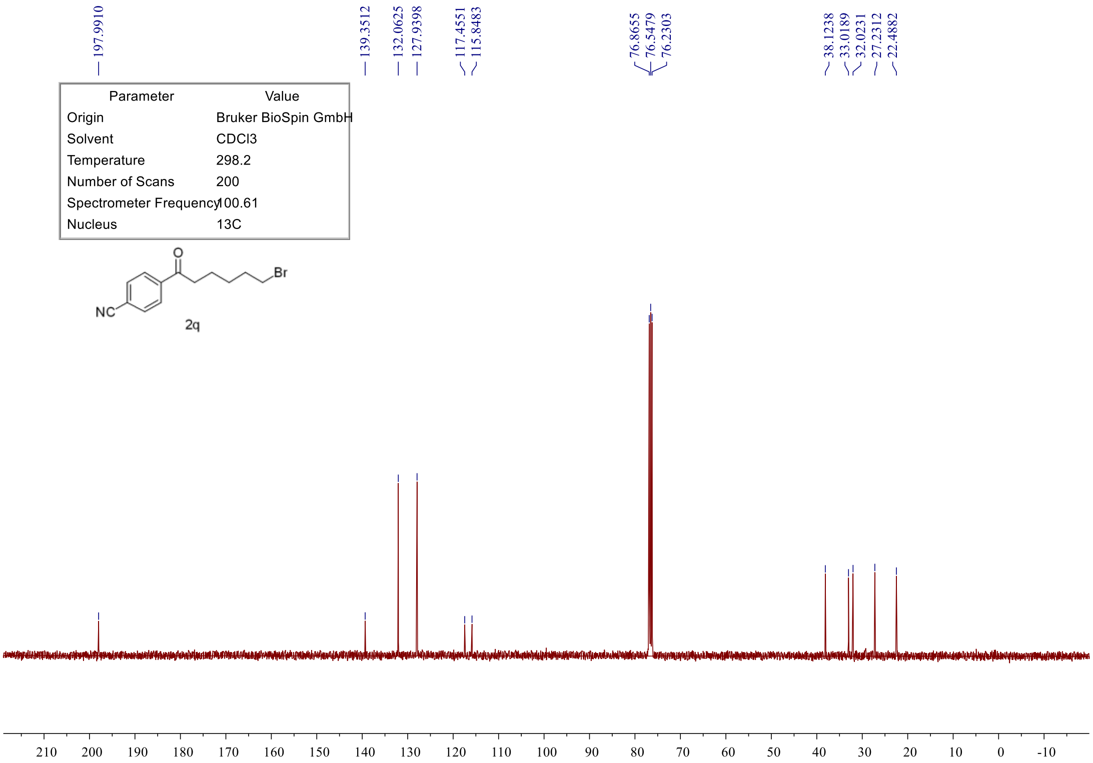

Parameter	Value
Origin	Bruker BioSpin GmbH
Solvent	CDCl3
Temperature	298.2
Number of Scans	200
Spectrometer Frequency	100.61
Nucleus	13C

2q

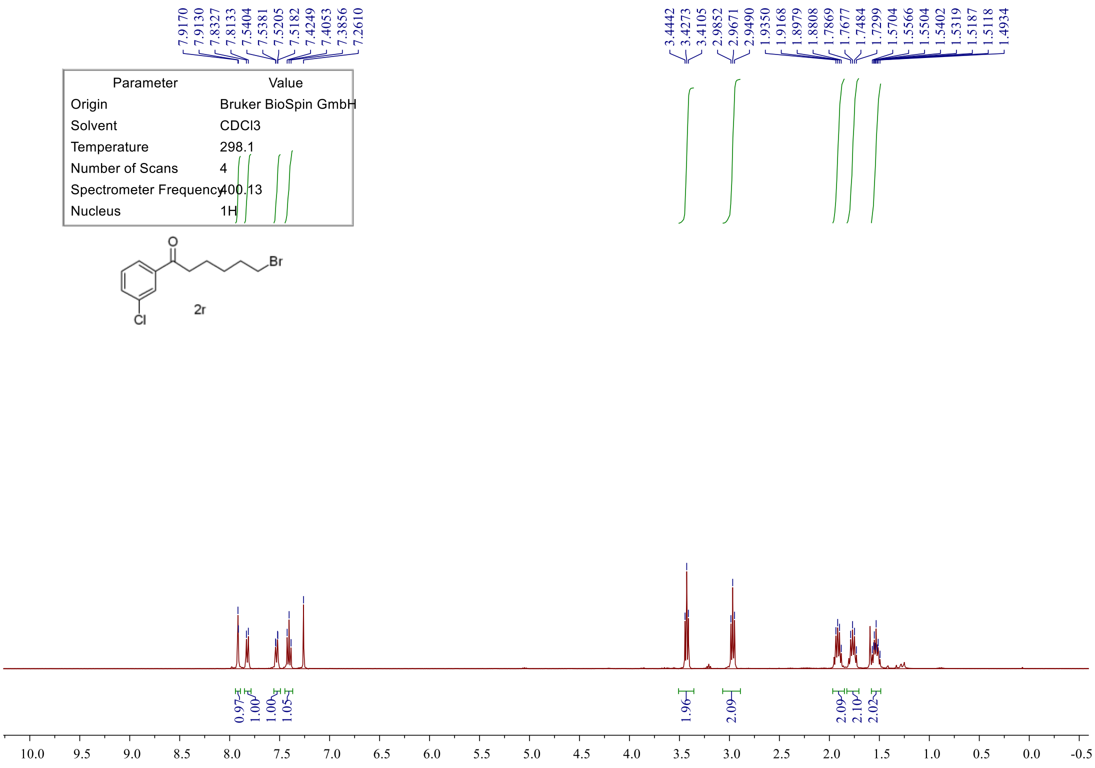
7.9170
7.9130
7.8327
7.8133
7.5404
7.5381
7.5205
7.5182
7.4249
7.4053
7.3856
7.2610
3.4442
3.4273
3.4105
2.9852
2.9671
2.9490
1.9350
1.9168
1.8979
1.8808
1.7869
1.7677
1.7484
1.7299
1.5704
1.5566
1.5504
1.5402
1.5319
1.5187
1.5118
1.4934
Parameter
Value
Origin
Bruker BioSpin GmbH
Solvent
CDCl3
Temperature
298.1
Number of Scans
4
Spectrometer Frequency
400.13
Nucleus
1H
O
Br
Cl
2r
0.97
1.00
1.00
1.05
1.96
2.09
2.09
2.10
2.02
10.0 9.5 9.0 8.5 8.0 7.5 7.0 6.5 6.0 5.5 5.0 4.5 4.0 3.5 3.0 2.5 2.0 1.5 1.0 0.5 0.0 -0.5

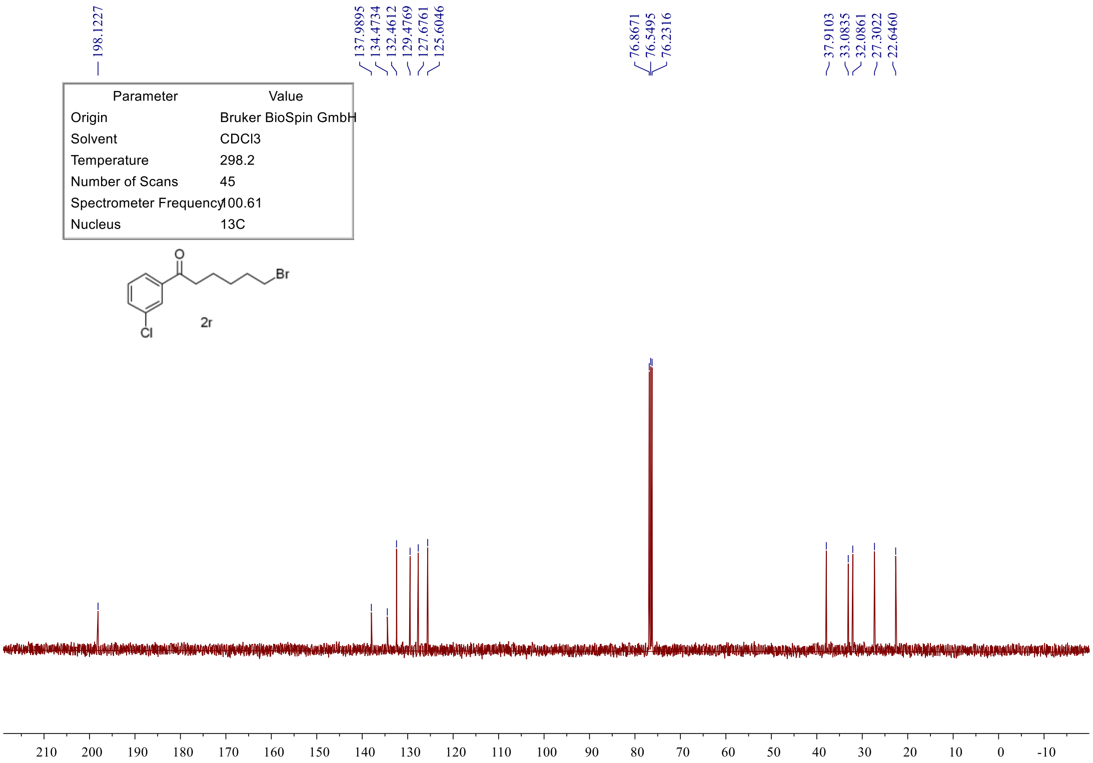
198.1227
137.9895
134.4734
132.4612
129.4769
127.6761
125.6046
76.8671
76.5495
76.2316
37.9103
33.0835
32.0861
27.3022
22.6460
Parameter
Value
Origin
Bruker BioSpin GmbH
Solvent
CDCl3
Temperature
298.2
Number of Scans
45
Spectrometer Frequency
100.61
Nucleus
13C
O
Br
Cl
2r
210 200 190 180 170 160 150 140 130 120 110 100 90 80 70 60 50 40 30 20 10 0 -10

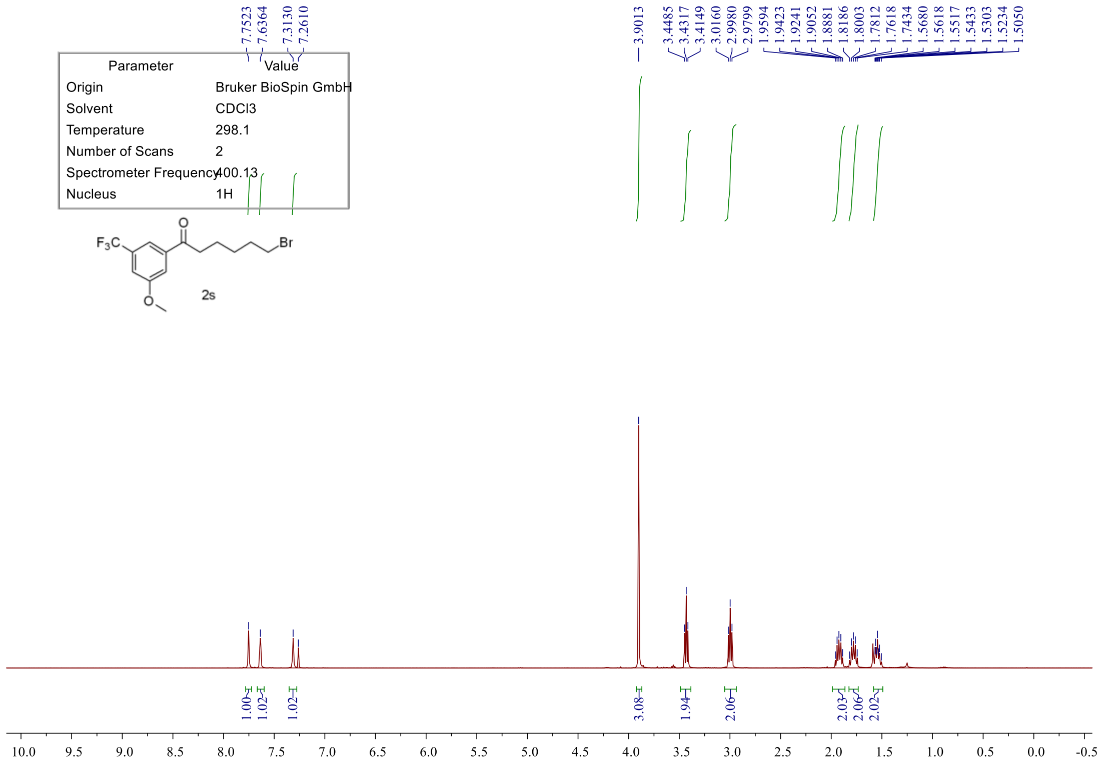
7.7523
7.6364
7.3130
7.2610
3.9013
3.4485
3.4317
3.4149
3.0160
2.9980
2.9799
1.9594
1.9423
1.9241
1.9052
1.8881
1.8186
1.8003
1.7812
1.7618
1.7434
1.5680
1.5618
1.5517
1.5433
1.5303
1.5234
1.5050
Parameter Value
Origin Bruker BioSpin GmbH
Solvent CDCl3
Temperature 298.1
Number of Scans 2
Spectrometer Frequency 400.13
Nucleus 1H
O
F3C
Br
O
2s
1.00
1.02
1.02
3.08
1.94
2.06
2.03
2.06
2.02
10.0 9.5 9.0 8.5 8.0 7.5 7.0 6.5 6.0 5.5 5.0 4.5 4.0 3.5 3.0 2.5 2.0 1.5 1.0 0.5 0.0 -0.5

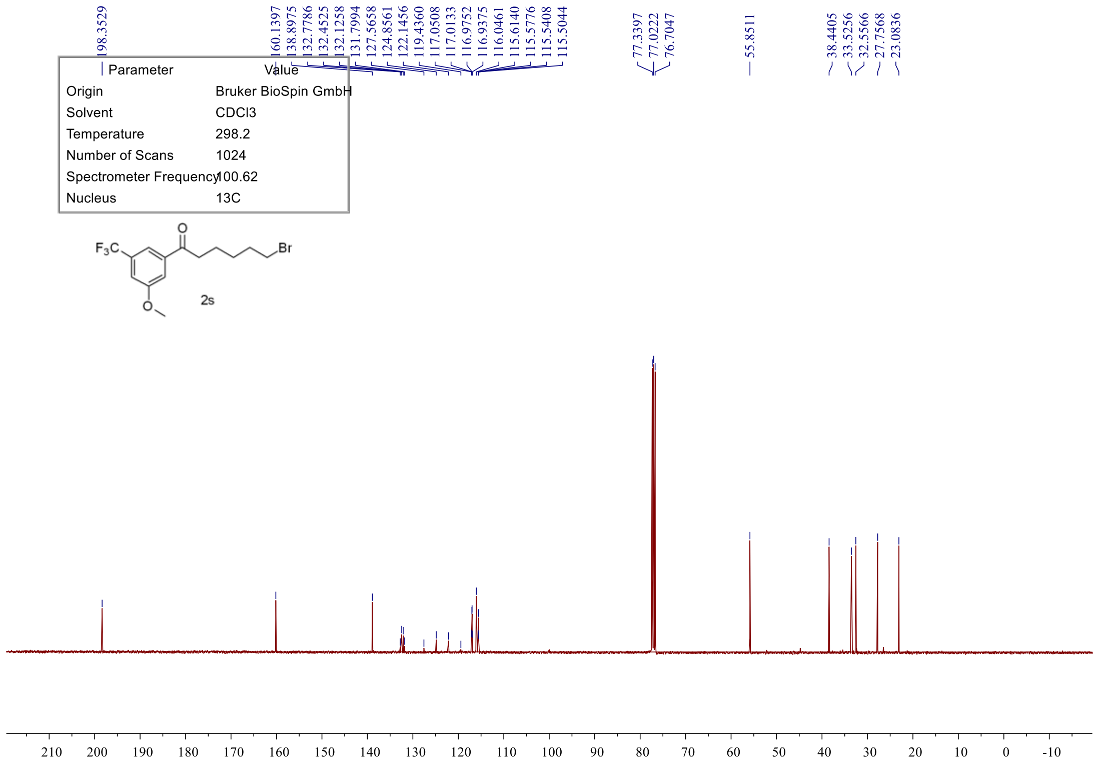
198.3529
160.1397
138.8975
132.7786
132.4525
132.1258
131.7994
127.5658
124.8561
122.1456
119.4360
117.0508
117.0133
116.9752
116.9375
116.0461
115.6140
115.5776
115.5408
115.5044
77.3397
77.0222
76.7047
55.8511
38.4405
33.5256
32.5566
27.7568
23.0836
Parameter Value
Origin Bruker BioSpin GmbH
Solvent CDCl3
Temperature 298.2
Number of Scans 1024
Spectrometer Frequency 100.62
Nucleus 13C
O
F3C
Br
O
2s
210 200 190 180 170 160 150 140 130 120 110 100 90 80 70 60 50 40 30 20 10 0 -10

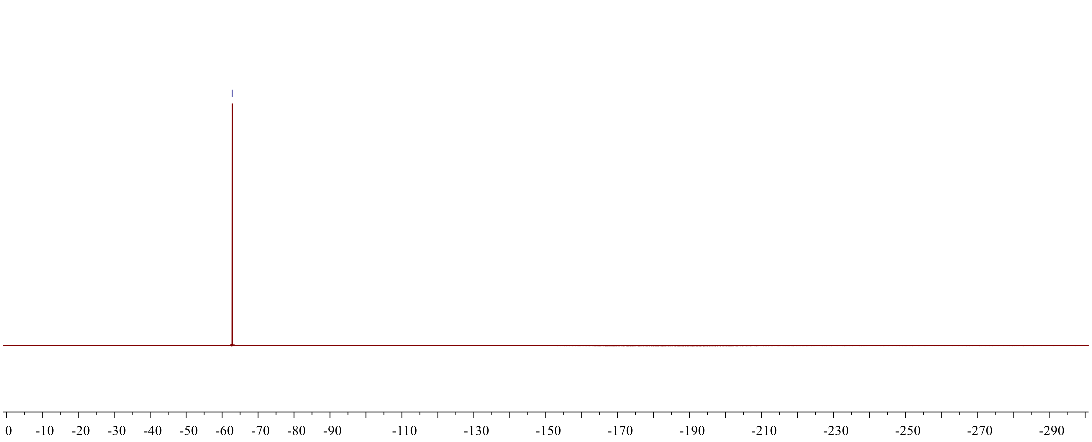

Parameter	Value
Origin	Bruker BioSpin GmbH
Solvent	CDCl3
Temperature	298.2
Number of Scans	2
Spectrometer Frequency	376.44
Nucleus	19F

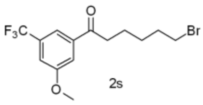

2s

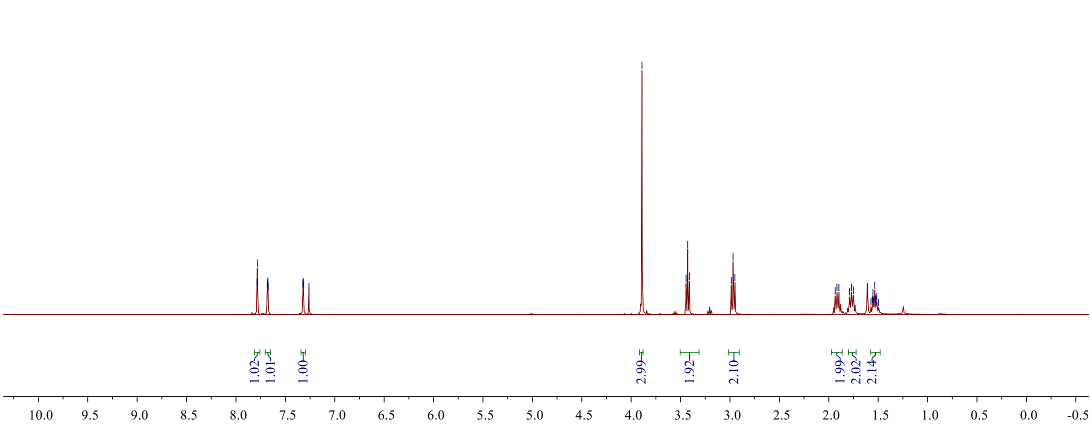

Parameter	Value
Origin	Bruker BioSpin GmbH
Solvent	CDCl3
Temperature	298.1
Number of Scans	2
Spectrometer Frequency	400.13
Nucleus	1H

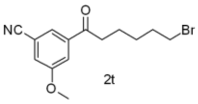

2t

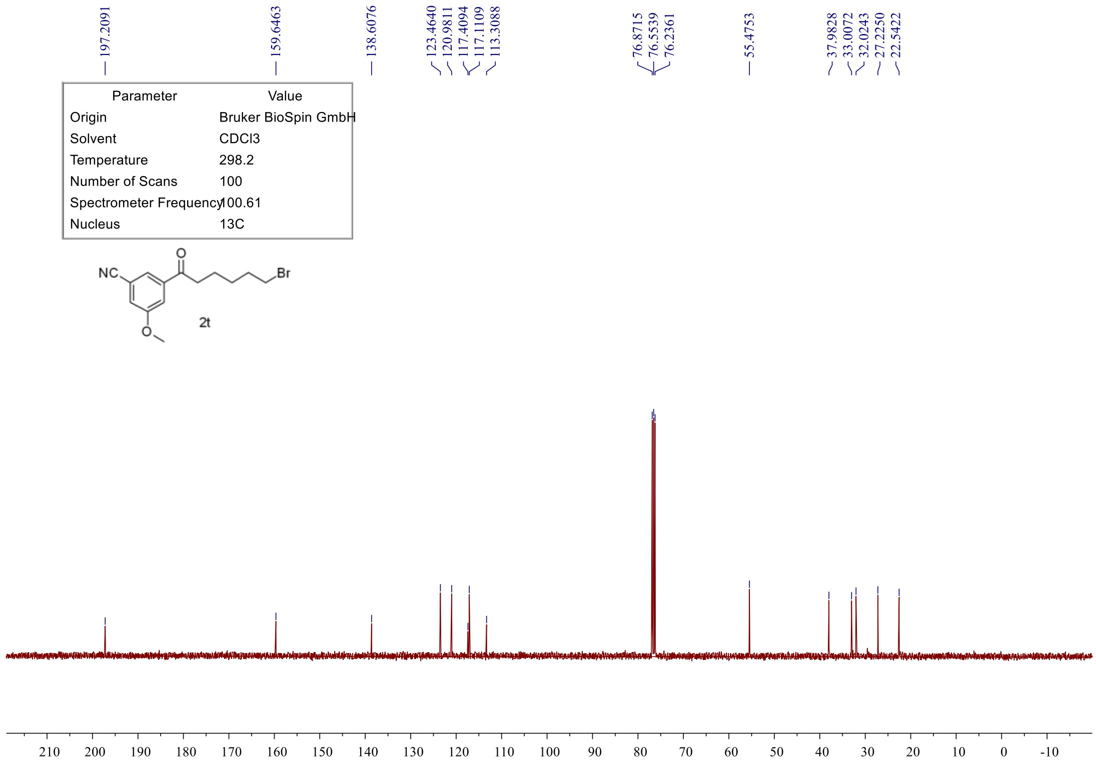
197.2091
159.6463
138.6076
123.4640
120.9811
117.4094
117.1109
113.3088
76.8715
76.5539
76.2361
55.4753
37.9828
33.0072
32.0243
27.2250
22.5422
Parameter Value
Origin Bruker BioSpin GmbH
Solvent CDCl3
Temperature 298.2
Number of Scans 100
Spectrometer Frequency 100.61
Nucleus 13C
NC
O
Br
O
2t

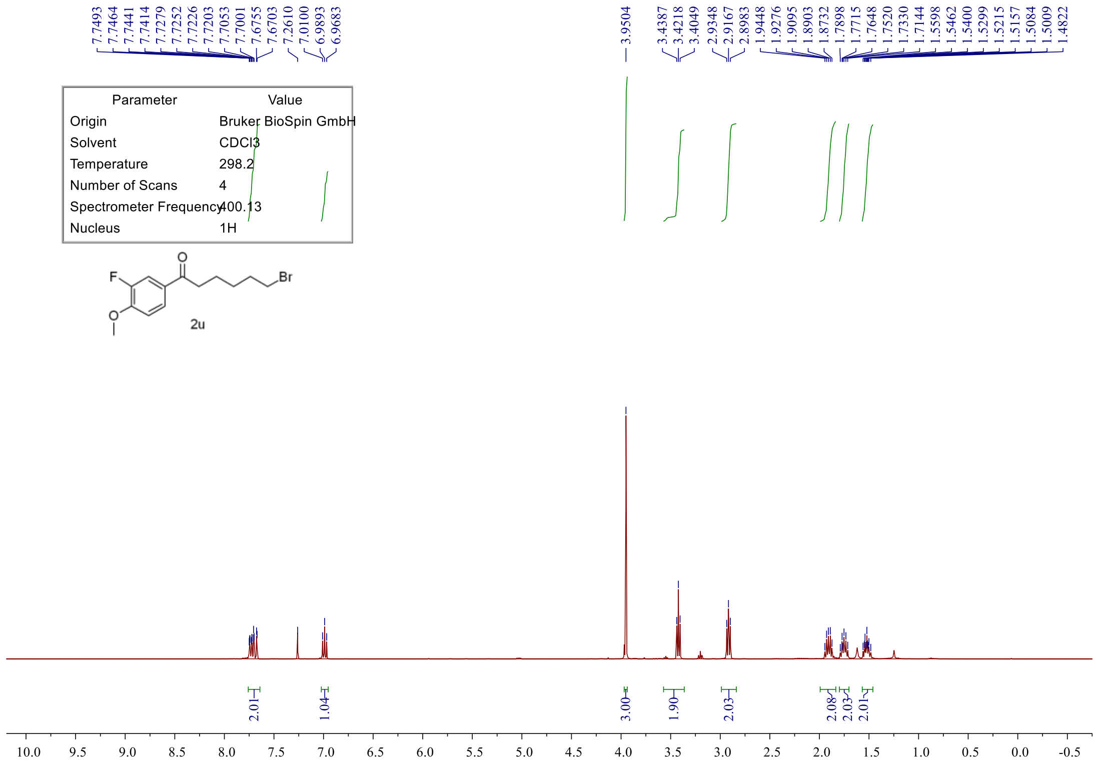
7.7493
7.7464
7.7441
7.7414
7.7279
7.7252
7.7226
7.7203
7.7053
7.7001
7.6755
7.6703
7.2610
7.0100
6.9893
6.9683
3.9504
3.4387
3.4218
3.4049
2.9348
2.9167
2.8983
1.9448
1.9276
1.9095
1.8903
1.8732
1.7898
1.7715
1.7648
1.7520
1.7330
1.7144
1.5598
1.5462
1.5400
1.5299
1.5215
1.5157
1.5084
1.5009
1.4822
Parameter Value
Origin Bruker BioSpin GmbH
Solvent CDCl3
Temperature 298.2
Number of Scans 4
Spectrometer Frequency 400.13
Nucleus 1H
F
O
Br
O
2u
2.01
1.04
3.00
1.90
2.03
2.08
2.03
2.01

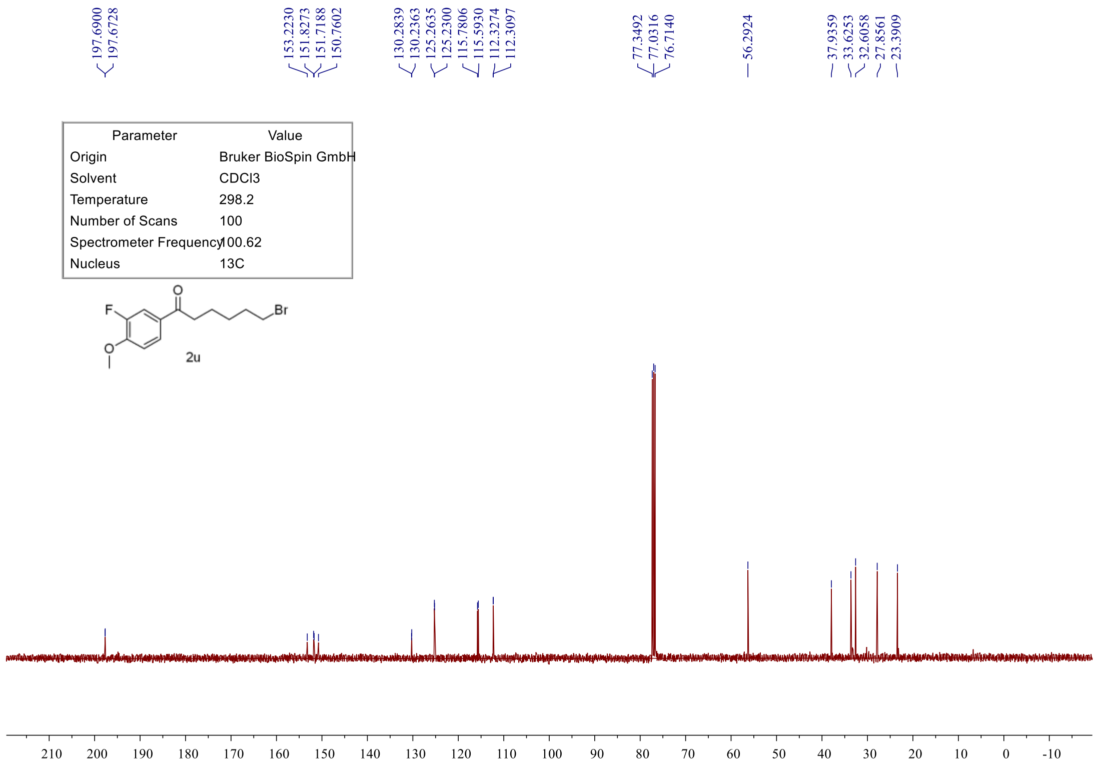
197.6900
197.6728
153.2230
151.8273
151.7188
150.7602
130.2839
130.2363
125.2635
125.2300
115.7806
115.5930
112.3274
112.3097
77.3492
77.0316
76.7140
56.2924
37.9359
33.6253
32.6058
27.8561
23.3909
Parameter Value
Origin Bruker BioSpin GmbH
Solvent CDCl3
Temperature 298.2
Number of Scans 100
Spectrometer Frequency 100.62
Nucleus 13C
F
O
Br
2u
210 200 190 180 170 160 150 140 130 120 110 100 90 80 70 60 50 40 30 20 10 0 -10

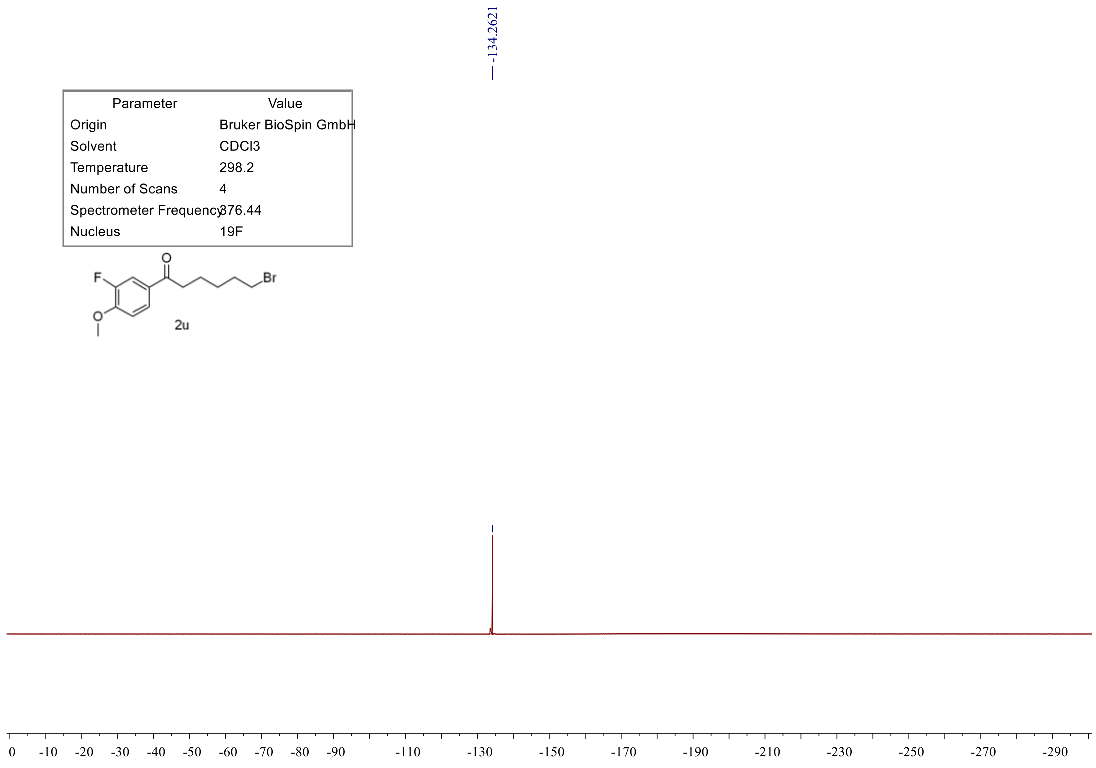
-134.2621
Parameter Value
Origin Bruker BioSpin GmbH
Solvent CDCl3
Temperature 298.2
Number of Scans 4
Spectrometer Frequency 376.44
Nucleus 19F
F
O
Br
2u
0 -10 -20 -30 -40 -50 -60 -70 -80 -90 -110 -130 -150 -170 -190 -210 -230 -250 -270 -290

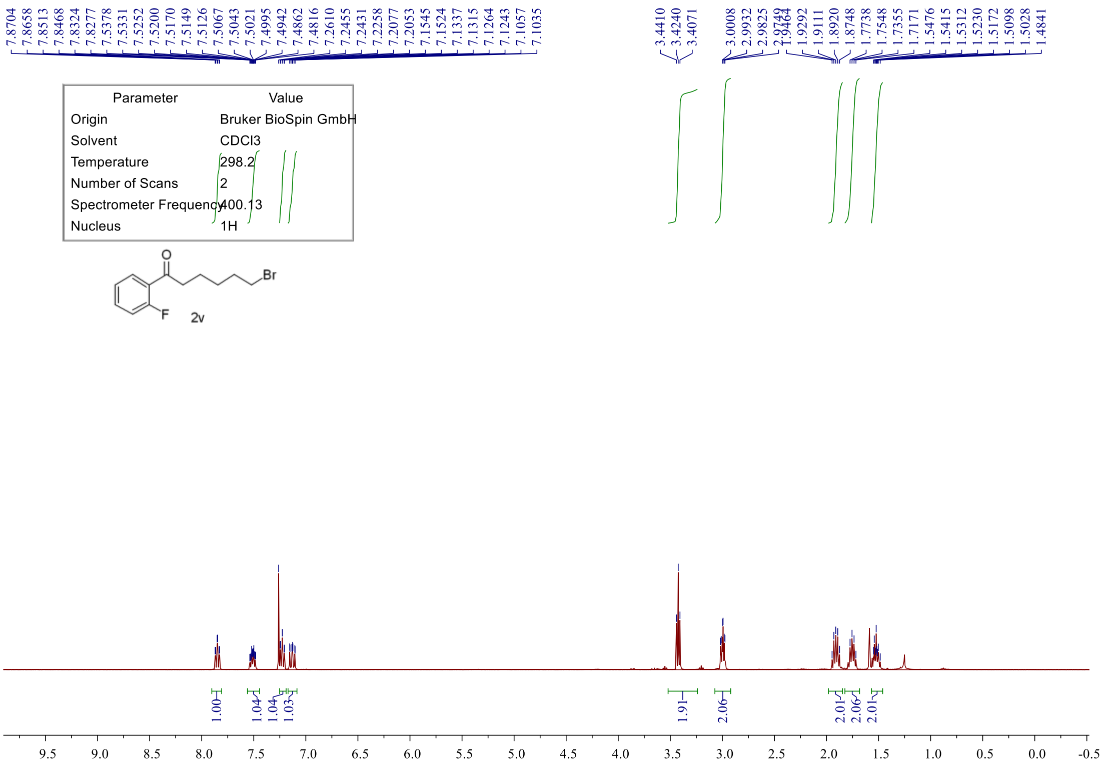
7.8704 7.8658 7.8513 7.8468 7.8324 7.8277 7.5378 7.5331 7.5252 7.5200 7.5170 7.5149 7.5126 7.5067 7.5043 7.5021 7.4995 7.4942 7.4862 7.4816 7.2610 7.2455 7.2431 7.2258 7.2077 7.2053 7.1545 7.1524 7.1337 7.1315 7.1264 7.1243 7.1057 7.1035
3.4410 3.4240 3.4071
3.0008 2.9932 2.9825 2.9749 1.9464 1.9292 1.9111 1.8920 1.8748 1.7738 1.7548 1.7355 1.7171 1.5476 1.5415 1.5312 1.5230 1.5172 1.5098 1.5028 1.4841
Parameter Value
Origin Bruker BioSpin GmbH
Solvent CDCl3
Temperature 298.2
Number of Scans 2
Spectrometer Frequency 400.13
Nucleus 1H
O
Br
F
2v
1.00 1.04 1.04 1.03 1.91 2.06 2.01 2.06 2.01
9.5 9.0 8.5 8.0 7.5 7.0 6.5 6.0 5.5 5.0 4.5 4.0 3.5 3.0 2.5 2.0 1.5 1.0 0.5 0.0 -0.5

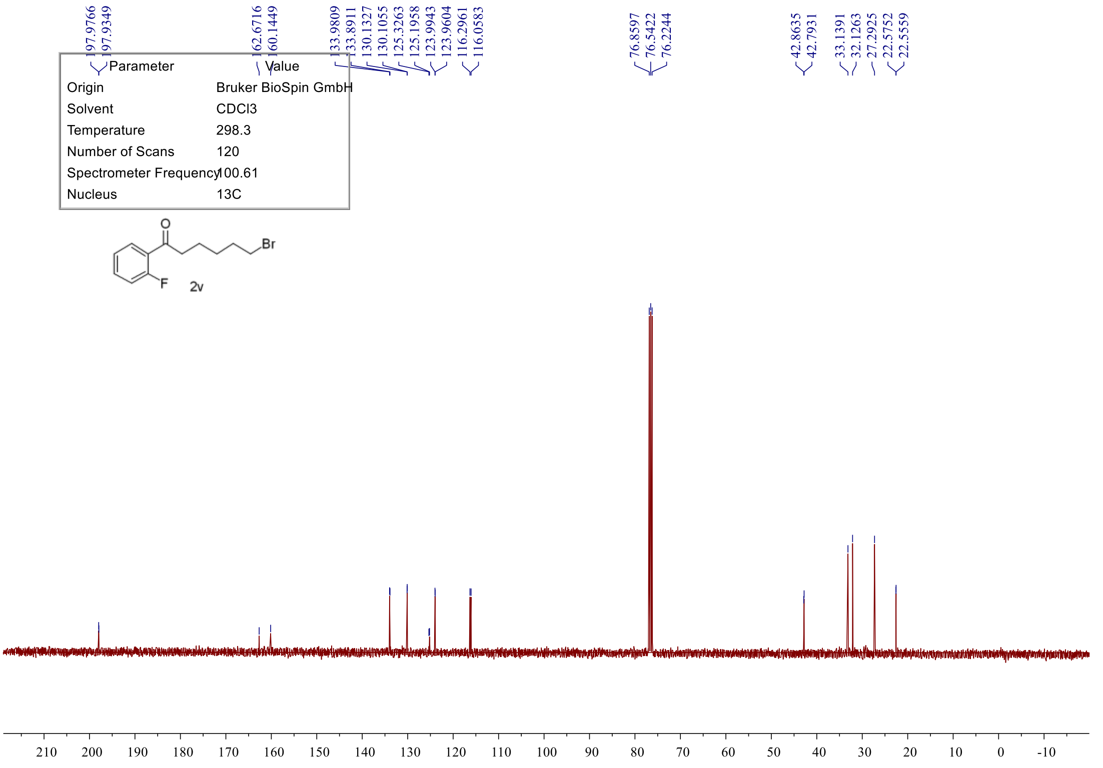
197.9766 197.9349
162.6716 160.1449
133.9809 133.8911 130.1327 130.1055 125.3263 125.1958 123.9943 123.9604 116.2961 116.0583
76.8597 76.5422 76.2244
42.8635 42.7931 33.1391 32.1263 27.2925 22.5752 22.5559
Parameter Value
Origin Bruker BioSpin GmbH
Solvent CDCl3
Temperature 298.3
Number of Scans 120
Spectrometer Frequency 100.61
Nucleus 13C
O
Br
F
2v
210 200 190 180 170 160 150 140 130 120 110 100 90 80 70 60 50 40 30 20 10 0 -10

Parameter	Value
Origin	Bruker BioSpin GmbH
Solvent	CDCl3
Temperature	298.2
Number of Scans	4
Spectrometer Frequency	376.50
Nucleus	19F

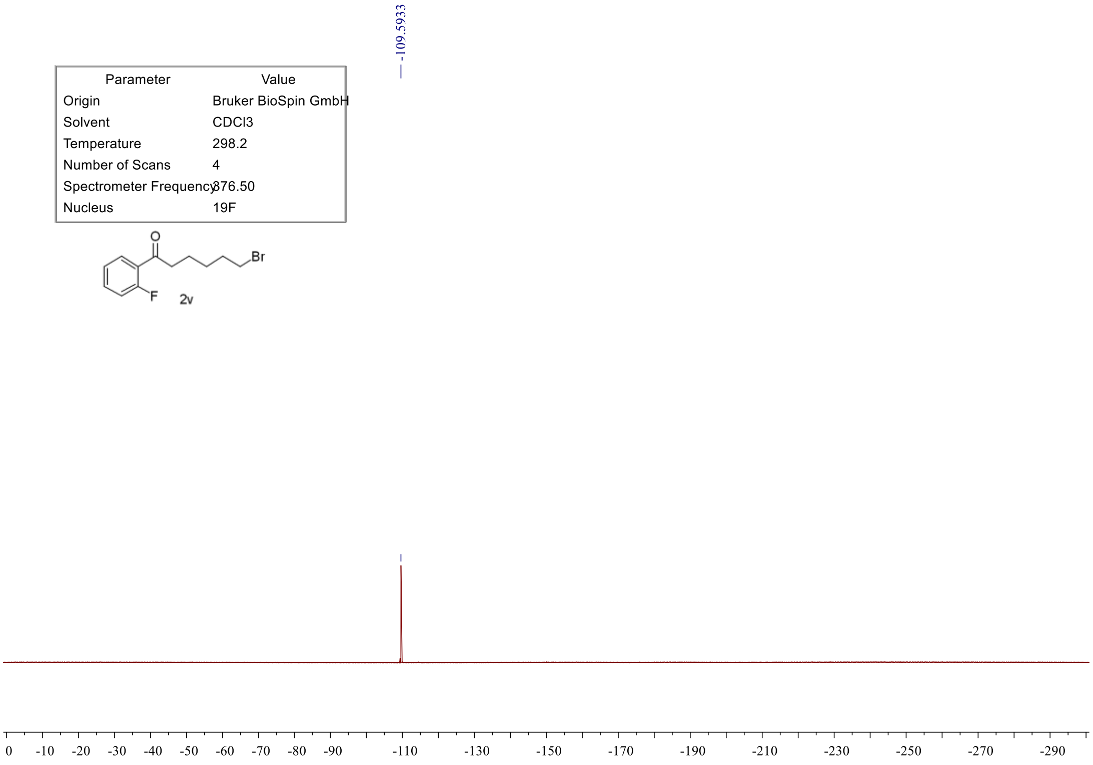

Parameter	Value
Origin	Bruker BioSpin GmbH
Solvent	CDCl3
Temperature	298.1
Number of Scans	4
Spectrometer Frequency	400.13
Nucleus	1H

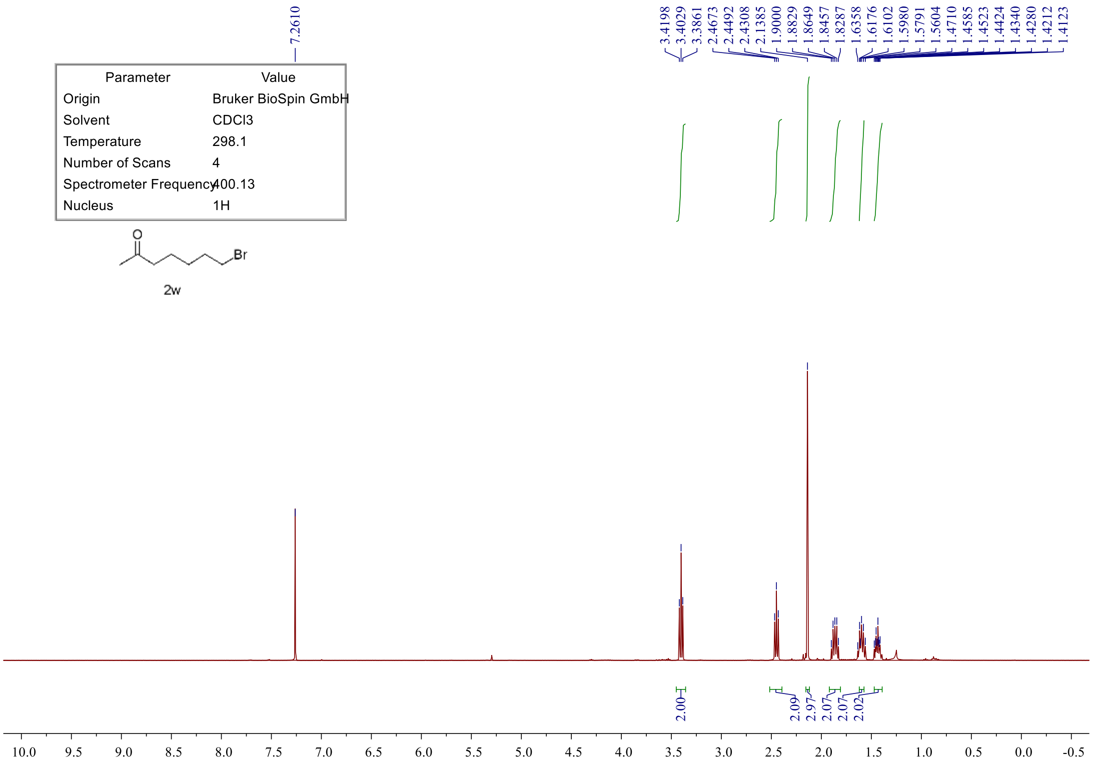

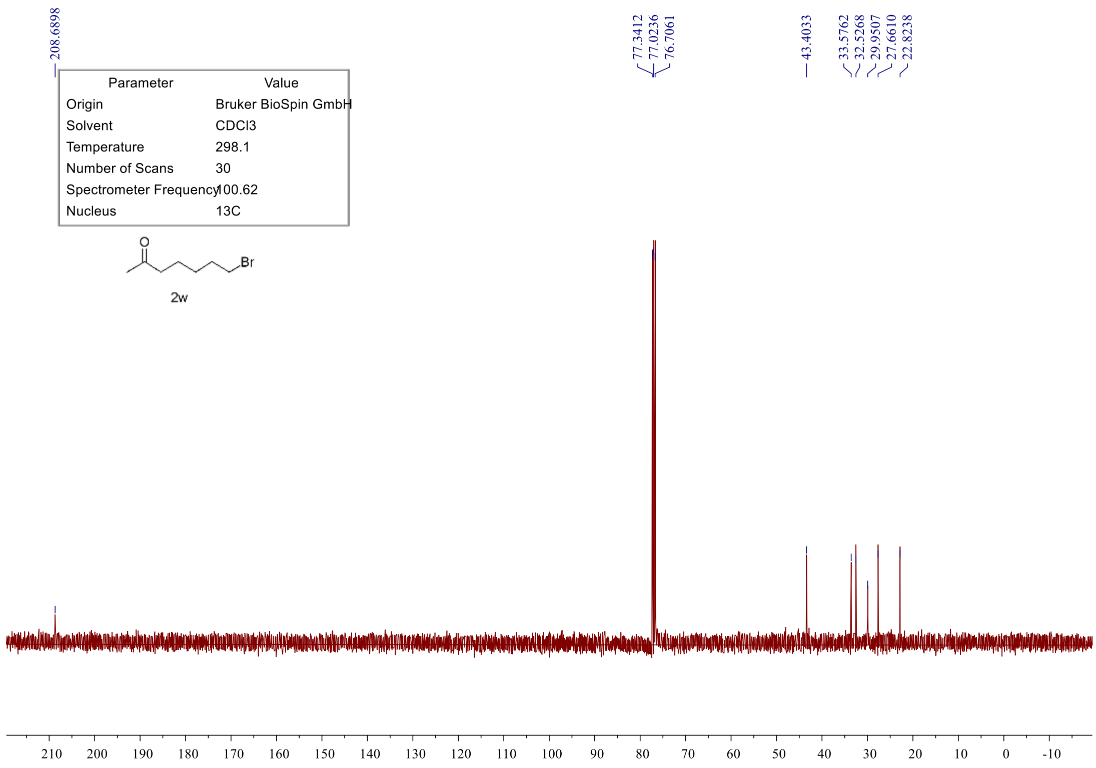
208.6898
77.3412
77.0236
76.7061
43.4033
33.5762
32.5268
29.9507
27.6610
22.8238
Parameter Value
Origin Bruker BioSpin GmbH
Solvent CDCl3
Temperature 298.1
Number of Scans 30
Spectrometer Frequency 100.62
Nucleus 13C
O
Br
2w
210 200 190 180 170 160 150 140 130 120 110 100 90 80 70 60 50 40 30 20 10 0 -10

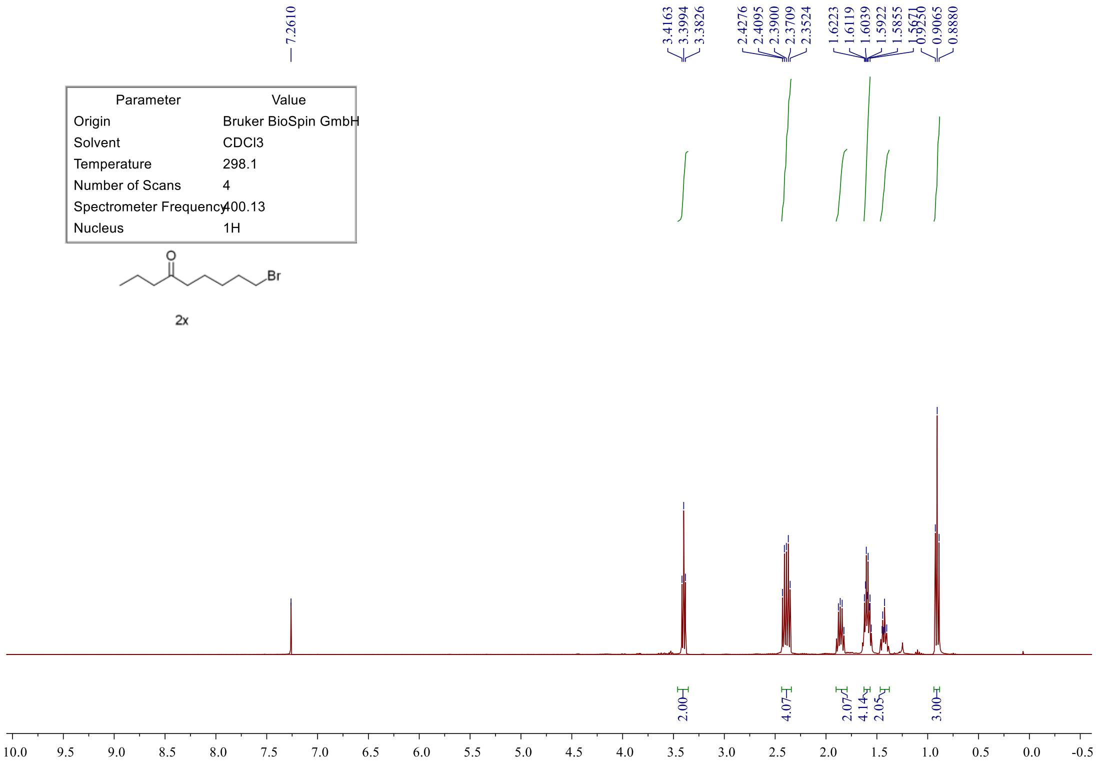
7.2610
3.4163
3.3994
3.3826
2.4276
2.4095
2.3900
2.3709
2.3524
1.6223
1.6119
1.6039
1.5922
1.5855
1.5671
0.9250
0.9065
0.8880
Parameter Value
Origin Bruker BioSpin GmbH
Solvent CDCl3
Temperature 298.1
Number of Scans 4
Spectrometer Frequency 400.13
Nucleus 1H
O
Br
2x
2.00
4.07
2.07
4.14
2.05
3.00
10.0 9.5 9.0 8.5 8.0 7.5 7.0 6.5 6.0 5.5 5.0 4.5 4.0 3.5 3.0 2.5 2.0 1.5 1.0 0.5 0.0 -0.5

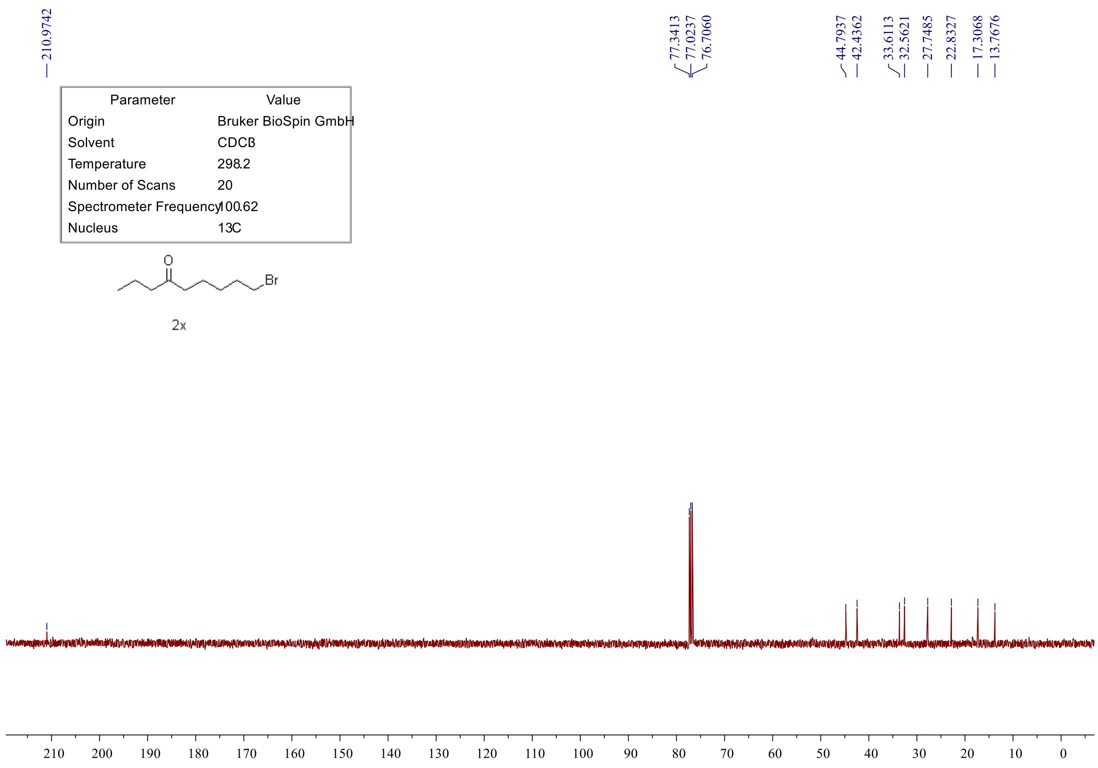

210.9742
77.3413
77.0237
76.7060
44.7937
42.4362
33.6113
32.5621
27.7485
22.8327
17.3068
13.7676
Parameter | Value
Origin | Bruker BioSpin GmbH
Solvent | CDCl3
Temperature | 298.2
Number of Scans | 20
Spectrometer Frequency | 100.62
Nucleus | 13C
Br
2x
210 200 190 180 170 160 150 140 130 120 110 100 90 80 70 60 50 40 30 20 10 0

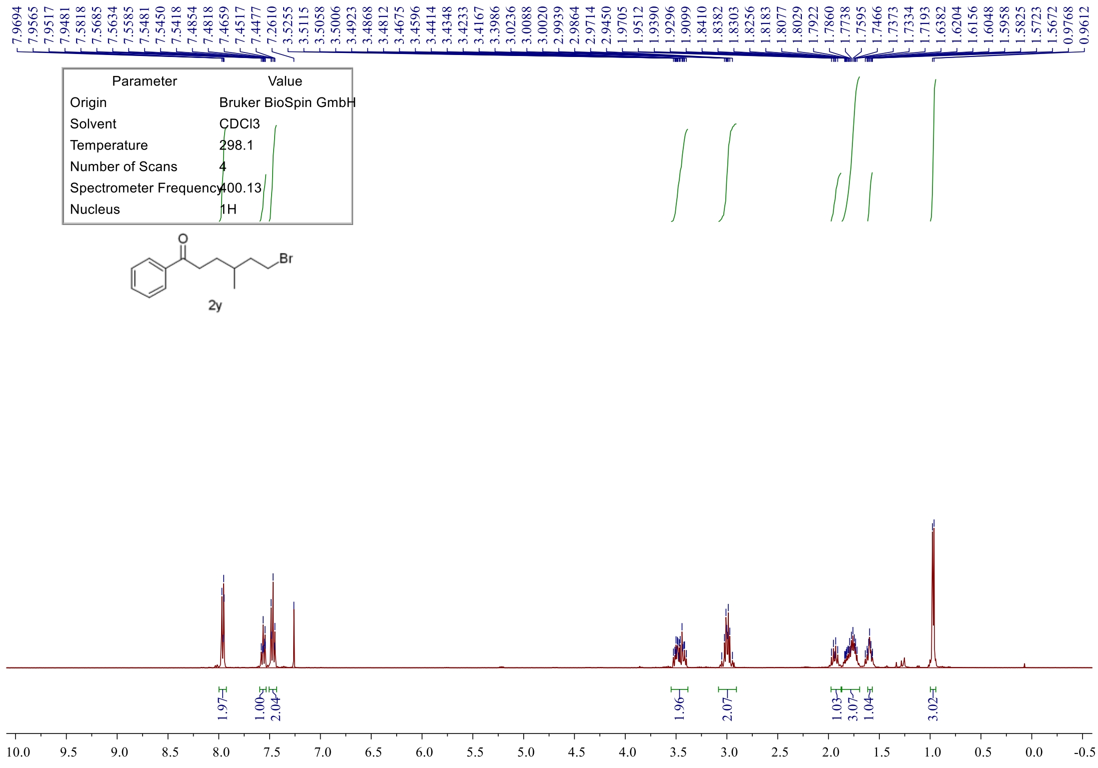

7.9694 7.9565 7.9517 7.9481 7.5818 7.5685 7.5634 7.5585 7.5481 7.5450 7.5418 7.4854 7.4818 7.4659 7.4517 7.4477 7.2610 3.5255 3.5115 3.5058 3.5006 3.4923 3.4868 3.4812 3.4675 3.4596 3.4414 3.4348 3.4233 3.4167 3.3986 3.0236 3.0088 3.0020 2.9939 2.9864 2.9714 2.9450 1.9705 1.9512 1.9390 1.9296 1.9099 1.8410 1.8382 1.8303 1.8256 1.8183 1.8077 1.8029 1.7922 1.7860 1.7738 1.7595 1.7466 1.7373 1.7334 1.7193 1.6382 1.6204 1.6156 1.6048 1.5958 1.5825 1.5723 1.5672 0.9768 0.9612
Parameter | Value
Origin | Bruker BioSpin GmbH
Solvent | CDCl3
Temperature | 298.1
Number of Scans | 4
Spectrometer Frequency | 400.13
Nucleus | 1H
Br
2y
1.97 1.00 2.04 1.96 2.07 1.03 3.07 1.04 3.02
10.0 9.5 9.0 8.5 8.0 7.5 7.0 6.5 6.0 5.5 5.0 4.5 4.0 3.5 3.0 2.5 2.0 1.5 1.0 0.5 0.0 -0.5

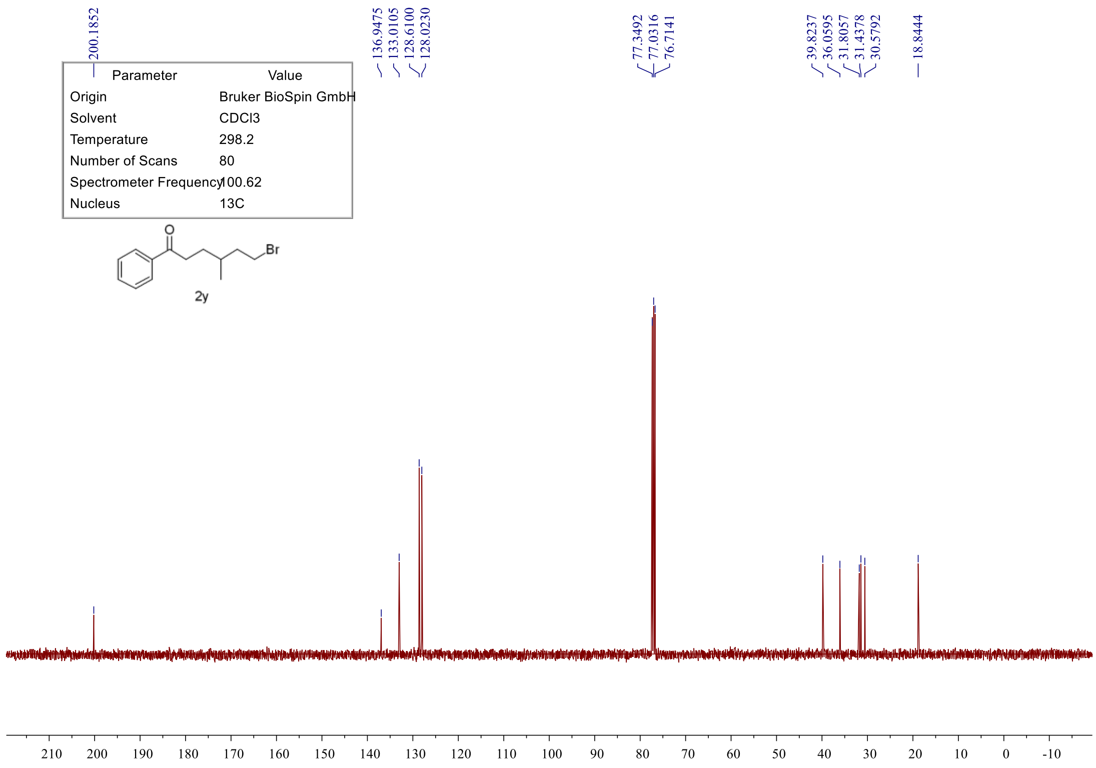
200.1852
136.9475
133.0105
128.6100
128.0230
77.3492
77.0316
76.7141
39.8237
36.0595
31.8057
31.4378
30.5792
18.8444
Parameter Value
Origin Bruker BioSpin GmbH
Solvent CDCl3
Temperature 298.2
Number of Scans 80
Spectrometer Frequency 100.62
Nucleus 13C
O
Br
2y
210 200 190 180 170 160 150 140 130 120 110 100 90 80 70 60 50 40 30 20 10 0 -10

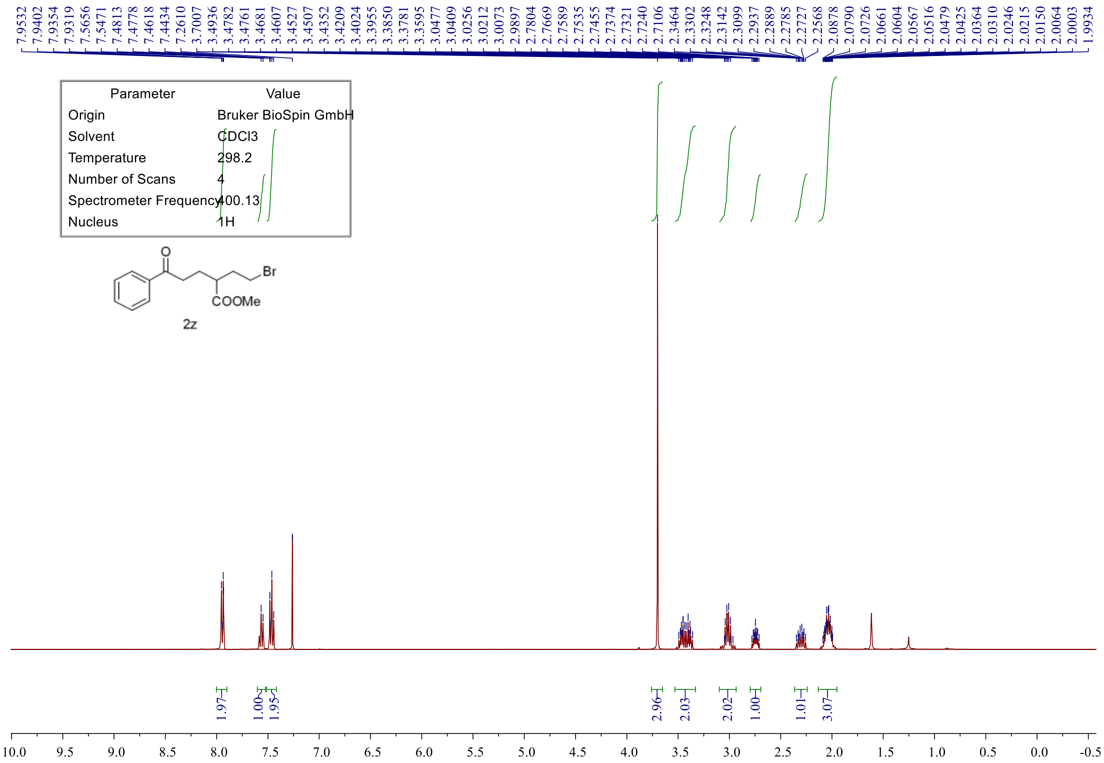
7.9532
7.9402
7.9354
7.9319
7.5656
7.5471
7.4813
7.4778
7.4618
7.4434
7.2610
3.7007
3.4936
3.4782
3.4761
3.4681
3.4607
3.4527
3.4507
3.4352
3.4209
3.4024
3.3955
3.3850
3.3781
3.3595
3.0477
3.0409
3.0256
3.0212
3.0073
2.9897
2.7804
2.7669
2.7589
2.7535
2.7455
2.7374
2.7321
2.7240
2.7106
2.3464
2.3302
2.3248
2.3142
2.3099
2.2937
2.2889
2.2785
2.2727
2.2568
2.0878
2.0790
2.0726
2.0661
2.0604
2.0567
2.0516
2.0479
2.0425
2.0364
2.0310
2.0246
2.0215
2.0150
2.0064
2.0003
1.9934
Parameter Value
Origin Bruker BioSpin GmbH
Solvent CDCl3
Temperature 298.2
Number of Scans 4
Spectrometer Frequency 400.13
Nucleus 1H
O
Br
COOMe
2z
1.97
1.00
1.95
2.96
2.03
2.02
1.00
1.01
3.07
10.0 9.5 9.0 8.5 8.0 7.5 7.0 6.5 6.0 5.5 5.0 4.5 4.0 3.5 3.0 2.5 2.0 1.5 1.0 0.5 0.0 -0.5

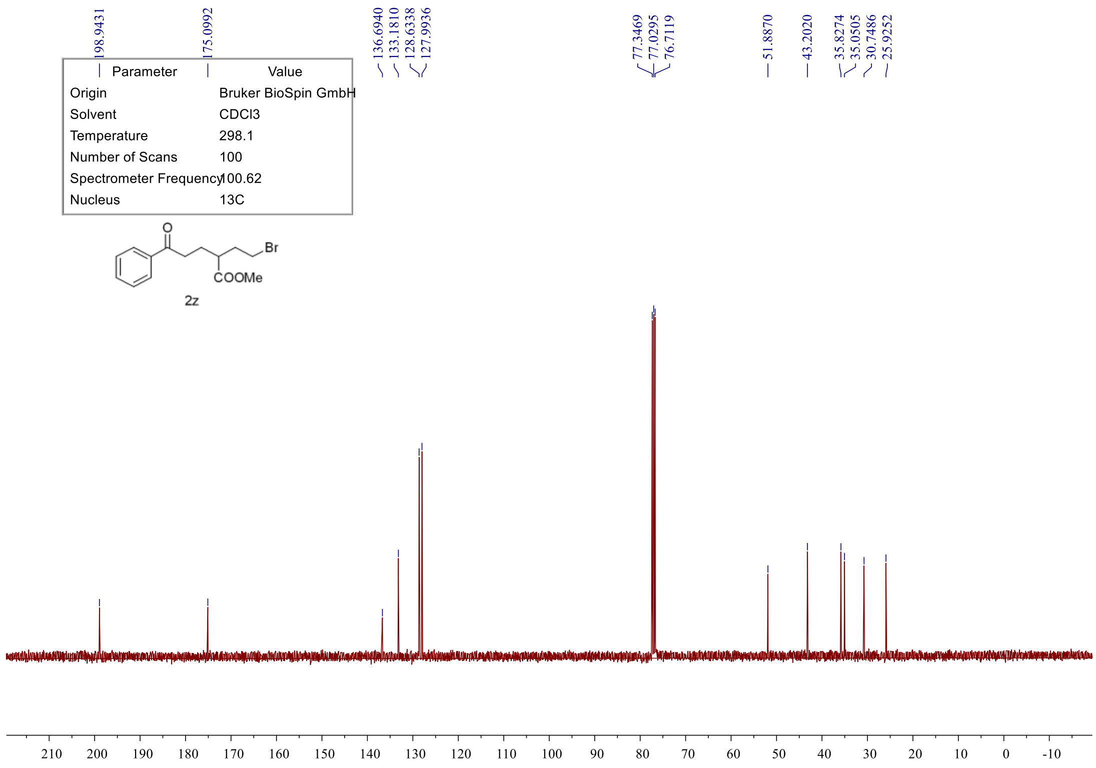
198.9431
175.0992
136.6940
133.1810
128.6338
127.9936
77.3469
77.0295
76.7119
51.8870
43.2020
35.8274
35.0505
30.7486
25.9252
Parameter
Value
Origin
Bruker BioSpin GmbH
Solvent
CDCl3
Temperature
298.1
Number of Scans
100
Spectrometer Frequency
100.62
Nucleus
13C
O
Br
COOMe
2z
210 200 190 180 170 160 150 140 130 120 110 100 90 80 70 60 50 40 30 20 10 0 -10

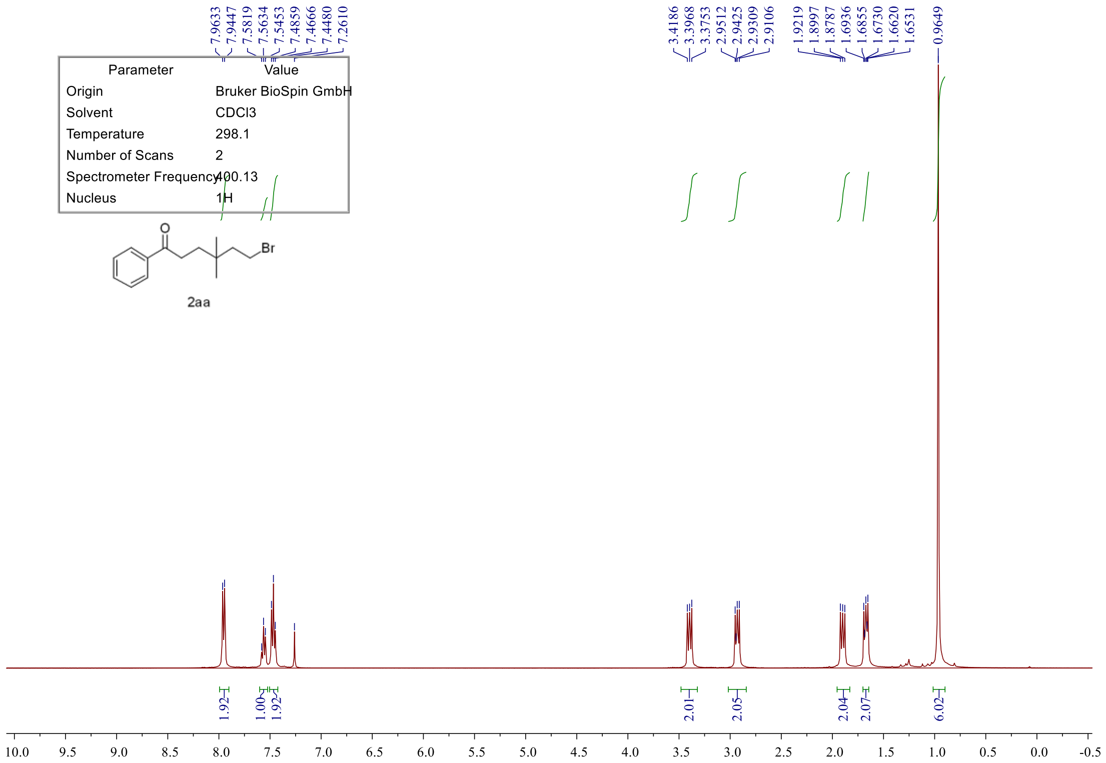
7.9633
7.9447
7.5819
7.5634
7.5453
7.4859
7.4666
7.4480
7.2610
3.4186
3.3968
3.3753
2.9512
2.9425
2.9309
2.9106
1.9219
1.8997
1.8787
1.6936
1.6855
1.6730
1.6620
1.6531
0.9649
Parameter
Value
Origin
Bruker BioSpin GmbH
Solvent
CDCl3
Temperature
298.1
Number of Scans
2
Spectrometer Frequency
400.13
Nucleus
1H
O
Br
2aa
1.92
1.00
1.92
2.01
2.05
2.04
2.07
6.02
10.0 9.5 9.0 8.5 8.0 7.5 7.0 6.5 6.0 5.5 5.0 4.5 4.0 3.5 3.0 2.5 2.0 1.5 1.0 0.5 0.0 -0.5

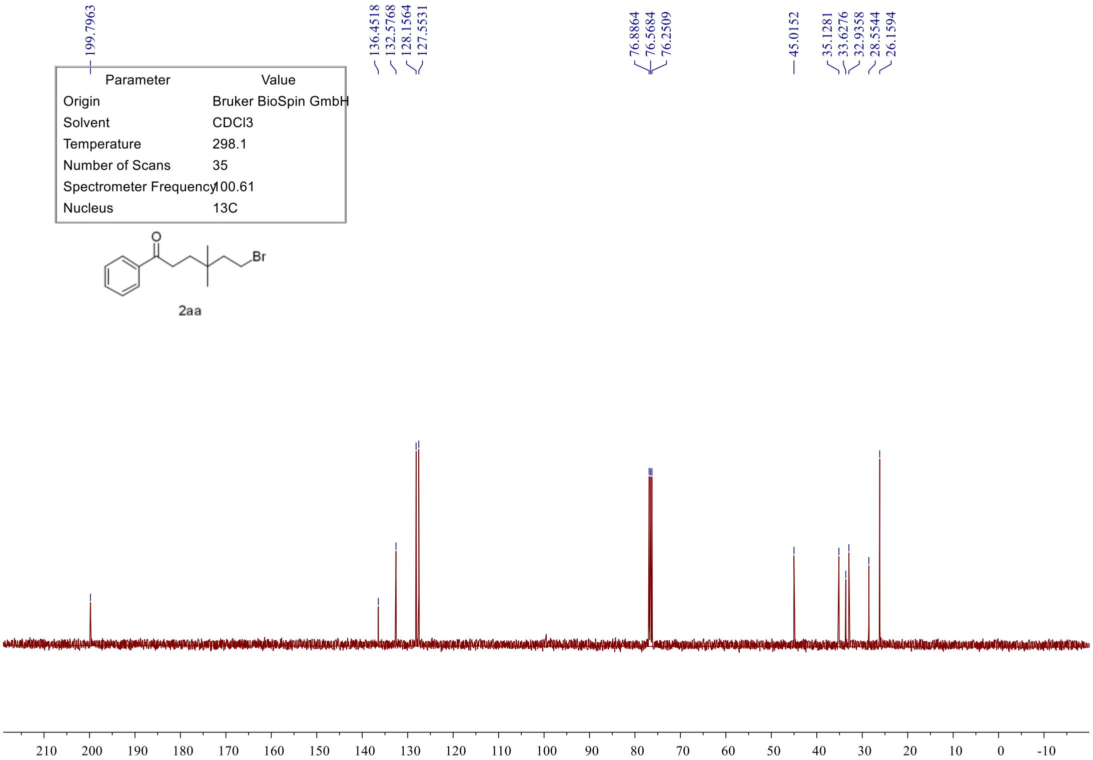
199.7963
136.4518
132.5768
128.1564
127.5531
76.8864
76.5684
76.2509
45.0152
35.1281
33.6276
32.9358
28.5544
26.1594
Parameter Value
Origin Bruker BioSpin GmbH
Solvent CDCl3
Temperature 298.1
Number of Scans 35
Spectrometer Frequency 100.61
Nucleus 13C
O
Br
2aa
210 200 190 180 170 160 150 140 130 120 110 100 90 80 70 60 50 40 30 20 10 0 -10

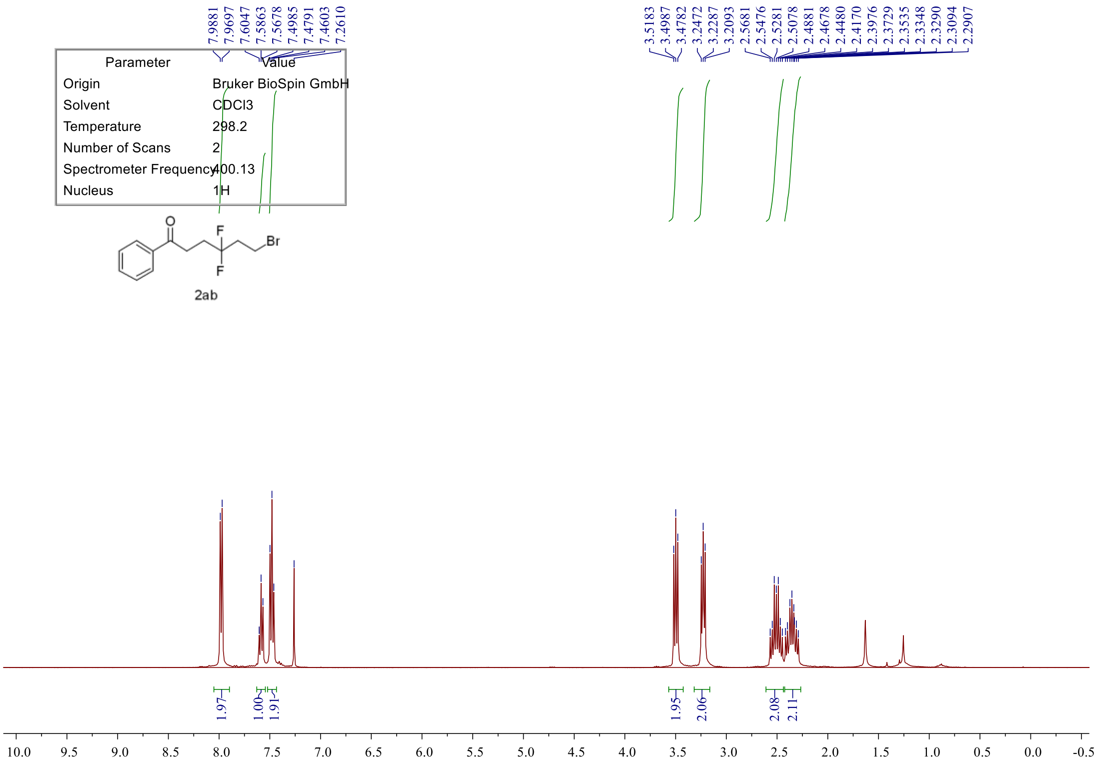
7.9881
7.9697
7.6047
7.5863
7.5678
7.4985
7.4791
7.4603
7.2610
3.5183
3.4987
3.4782
3.2472
3.2287
3.2093
2.5681
2.5476
2.5281
2.5078
2.4881
2.4678
2.4480
2.4170
2.3976
2.3729
2.3535
2.3348
2.3290
2.3094
2.2907
Parameter Value
Origin Bruker BioSpin GmbH
Solvent CDCl3
Temperature 298.2
Number of Scans 2
Spectrometer Frequency 400.13
Nucleus 1H
O
F
F
Br
2ab
1.97
1.00
1.91
1.95
2.06
2.08
2.11
10.0 9.5 9.0 8.5 8.0 7.5 7.0 6.5 6.0 5.5 5.0 4.5 4.0 3.5 3.0 2.5 2.0 1.5 1.0 0.5 0.0 -0.5

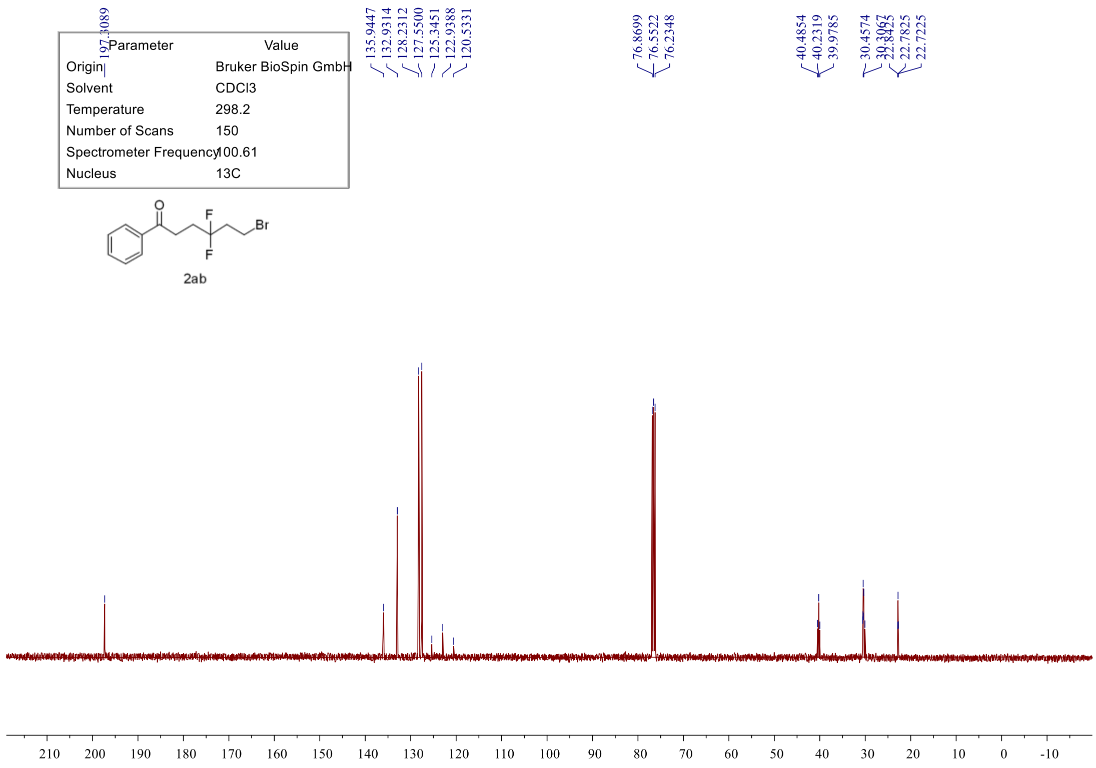

197.5089
135.9447
132.9314
128.2312
127.5500
125.3451
122.9388
120.5331
76.8699
76.5522
76.2348
40.4854
40.2319
39.9785
30.4574
30.3067
22.8425
22.7825
22.7225
Parameter Value
Origin Bruker BioSpin GmbH
Solvent CDCl3
Temperature 298.2
Number of Scans 150
Spectrometer Frequency 100.61
Nucleus 13C
O
F
F
Br
2ab
210 200 190 180 170 160 150 140 130 120 110 100 90 80 70 60 50 40 30 20 10 0 -10

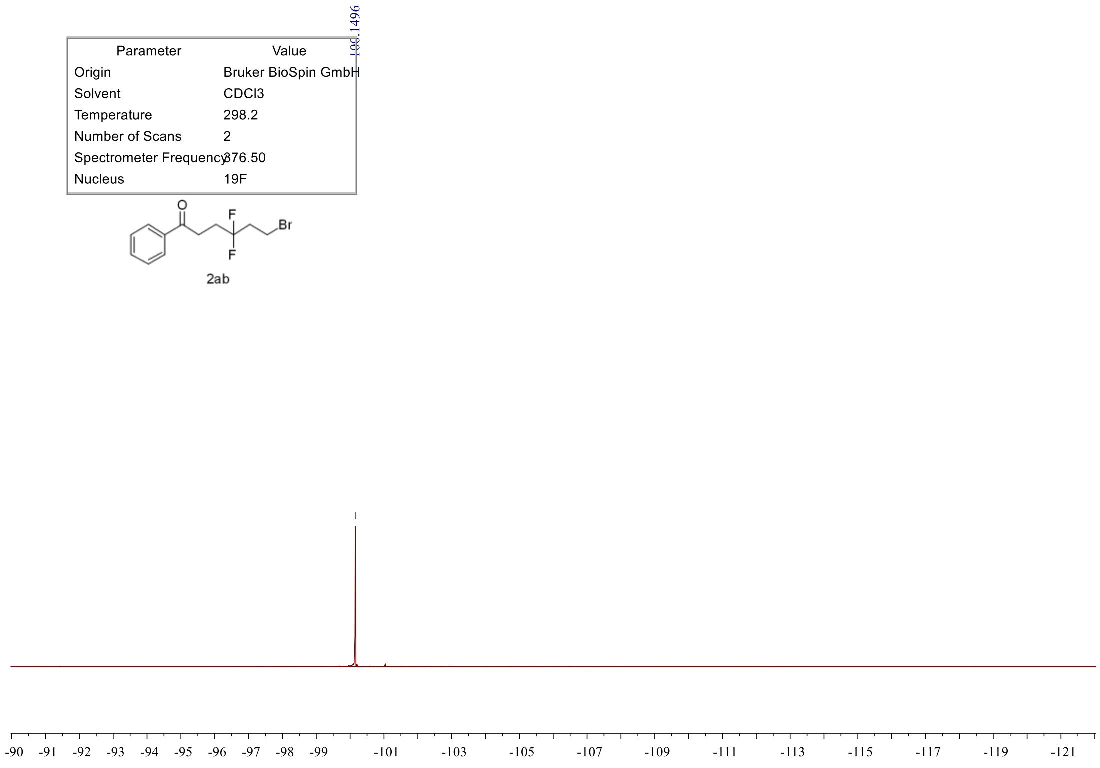

100.1496
Parameter Value
Origin Bruker BioSpin GmbH
Solvent CDCl3
Temperature 298.2
Number of Scans 2
Spectrometer Frequency 376.50
Nucleus 19F
O
F
F
Br
2ab
-90 -91 -92 -93 -94 -95 -96 -97 -98 -99 -101 -103 -105 -107 -109 -111 -113 -115 -117 -119 -121

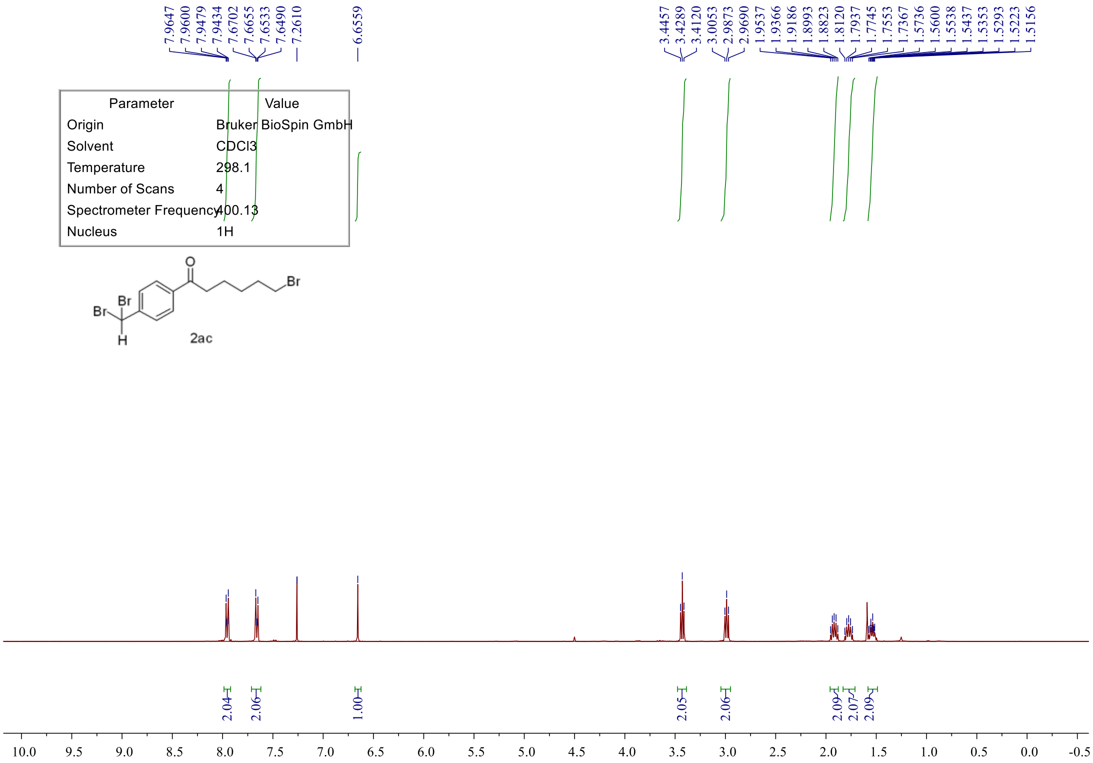
7.9647
7.9600
7.9479
7.9434
7.6702
7.6655
7.6533
7.6490
7.2610
6.6559
3.4457
3.4289
3.4120
3.0053
2.9873
2.9690
1.9537
1.9366
1.9186
1.8993
1.8823
1.8120
1.7937
1.7745
1.7553
1.7367
1.5736
1.5600
1.5538
1.5437
1.5353
1.5293
1.5223
1.5156
Parameter | Value
Origin | Bruker BioSpin GmbH
Solvent | CDCl3
Temperature | 298.1
Number of Scans | 4
Spectrometer Frequency | 400.13
Nucleus | 1H
O
Br
Br
Br
H
2ac
2.04
2.06
1.00
2.05
2.06
2.09
2.07
2.09
10.0 9.5 9.0 8.5 8.0 7.5 7.0 6.5 6.0 5.5 5.0 4.5 4.0 3.5 3.0 2.5 2.0 1.5 1.0 0.5 0.0 -0.5

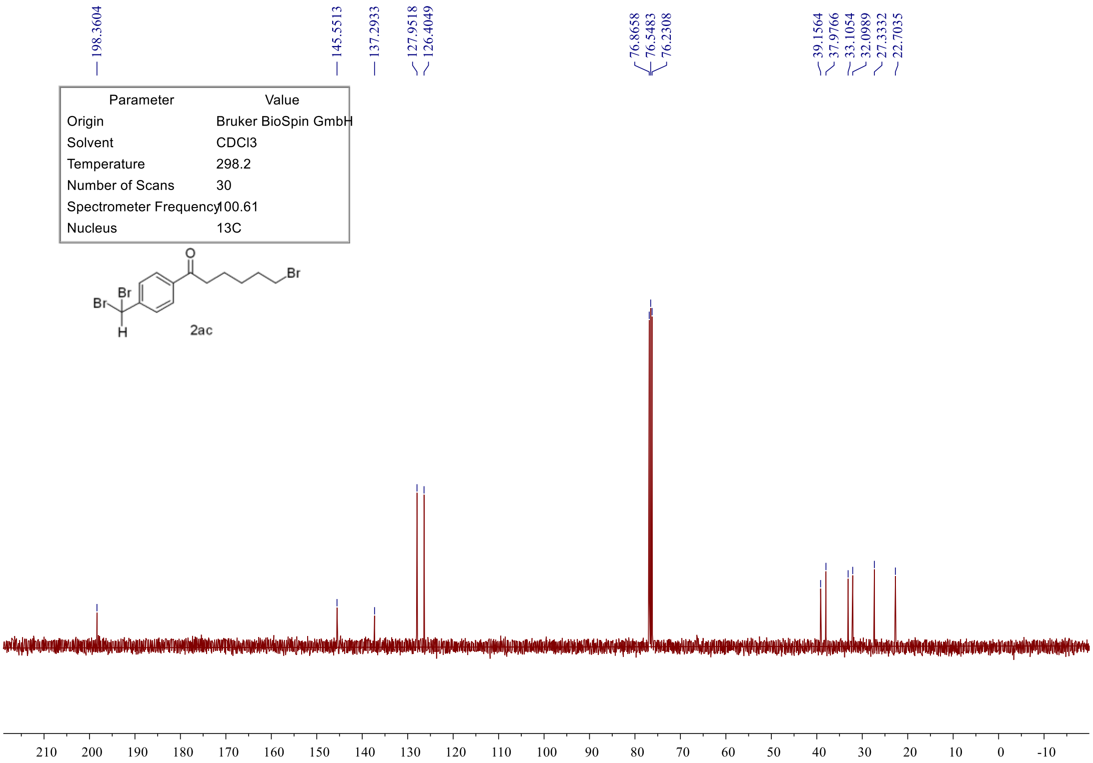
198.3604
145.5513
137.2933
127.9518
126.4049
76.8658
76.5483
76.2308
39.1564
37.9766
33.1054
32.0989
27.3332
22.7035
Parameter | Value
Origin | Bruker BioSpin GmbH
Solvent | CDCl3
Temperature | 298.2
Number of Scans | 30
Spectrometer Frequency | 100.61
Nucleus | 13C
O
Br
Br
Br
H
2ac
210 200 190 180 170 160 150 140 130 120 110 100 90 80 70 60 50 40 30 20 10 0 -10

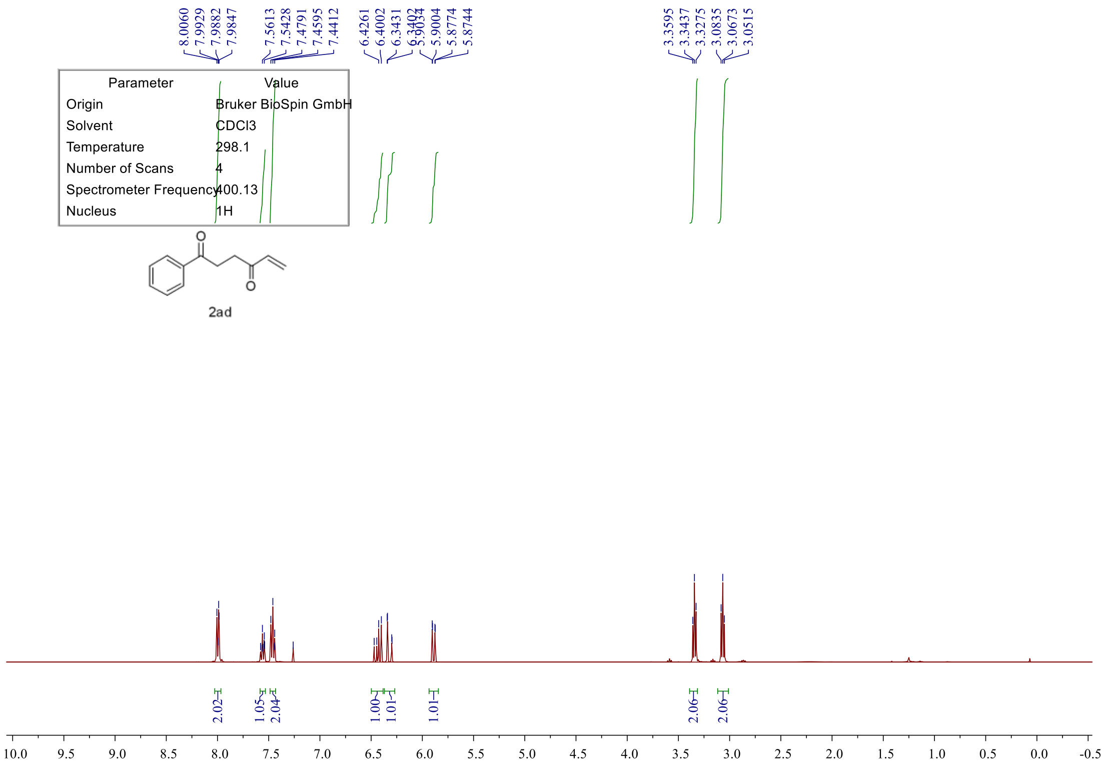
8.0060
7.9929
7.9882
7.9847
7.5613
7.5428
7.4791
7.4595
7.4412
6.4261
6.4002
6.3431
6.3402
5.9034
5.9004
5.8774
5.8744
3.3595
3.3437
3.3275
3.0835
3.0673
3.0515
Parameter Value
Origin Bruker BioSpin GmbH
Solvent CDCl3
Temperature 298.1
Number of Scans 4
Spectrometer Frequency 400.13
Nucleus 1H
O
O
2ad
2.02
1.05
2.04
1.00
1.01
1.01
2.06
2.06
10.0 9.5 9.0 8.5 8.0 7.5 7.0 6.5 6.0 5.5 5.0 4.5 4.0 3.5 3.0 2.5 2.0 1.5 1.0 0.5 0.0 -0.5

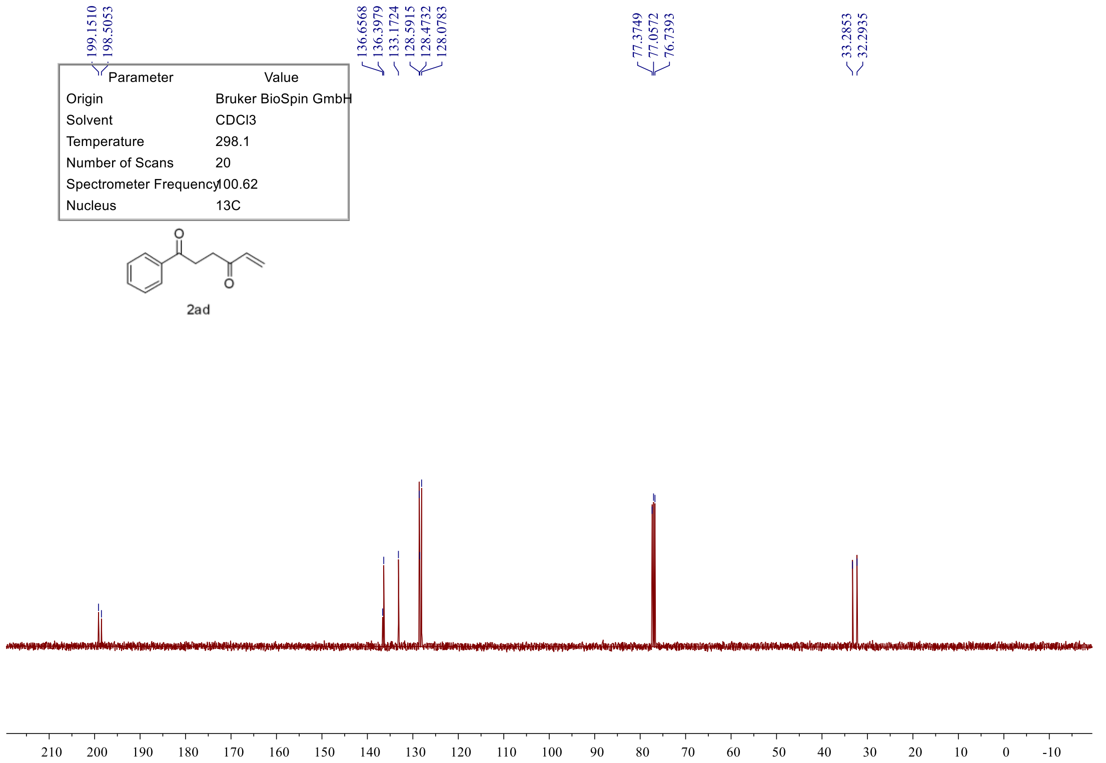
199.1510
198.5053
136.6568
136.3979
133.1724
128.5915
128.4732
128.0783
77.3749
77.0572
76.7393
33.2853
32.2935
Parameter Value
Origin Bruker BioSpin GmbH
Solvent CDCl3
Temperature 298.1
Number of Scans 20
Spectrometer Frequency 100.62
Nucleus 13C
O
O
2ad
210 200 190 180 170 160 150 140 130 120 110 100 90 80 70 60 50 40 30 20 10 0 -10

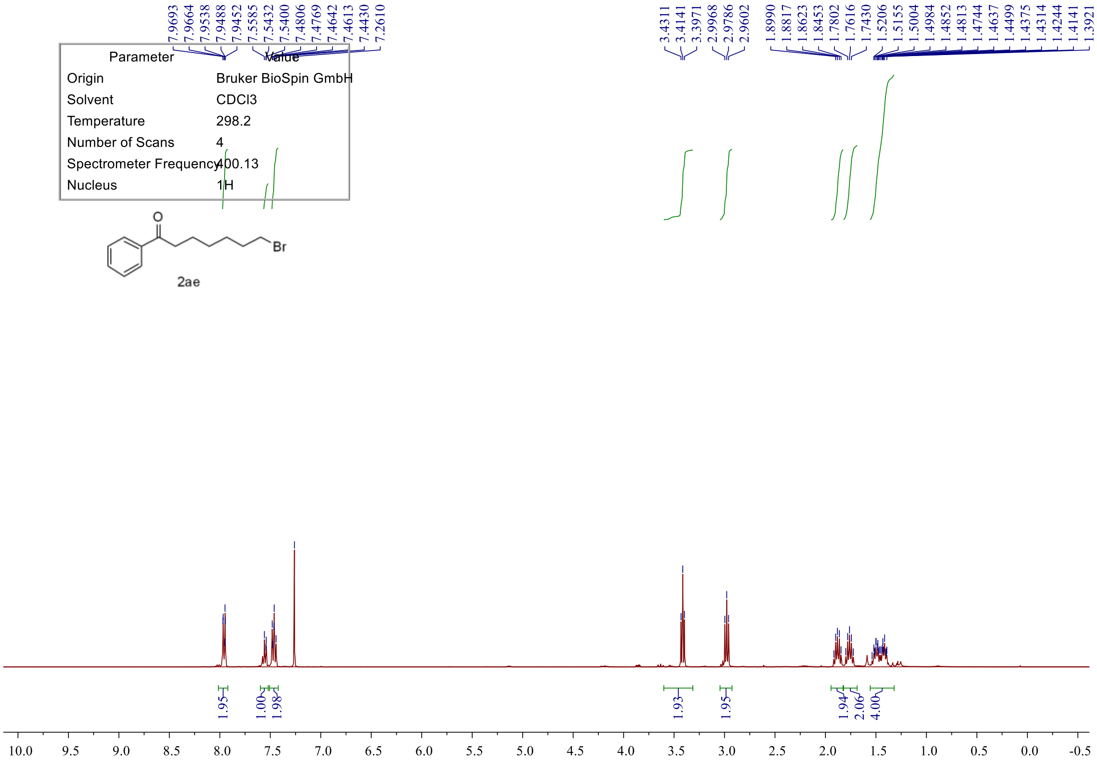
7.9693
7.9664
7.9538
7.9488
7.9452
7.5585
7.5432
7.5400
7.4806
7.4769
7.4642
7.4613
7.4430
7.2610
3.4311
3.4141
3.3971
2.9968
2.9786
2.9602
1.8990
1.8817
1.8623
1.8453
1.7802
1.7616
1.7430
1.5206
1.5155
1.5004
1.4984
1.4852
1.4813
1.4744
1.4637
1.4499
1.4375
1.4314
1.4244
1.4141
1.3921
Parameter Value
Origin Bruker BioSpin GmbH
Solvent CDCl3
Temperature 298.2
Number of Scans 4
Spectrometer Frequency 400.13
Nucleus 1H
O
Br
2ae
1.95
1.00
1.98
1.93
1.95
1.94
2.06
4.00
10.0 9.5 9.0 8.5 8.0 7.5 7.0 6.5 6.0 5.5 5.0 4.5 4.0 3.5 3.0 2.5 2.0 1.5 1.0 0.5 0.0 -0.5

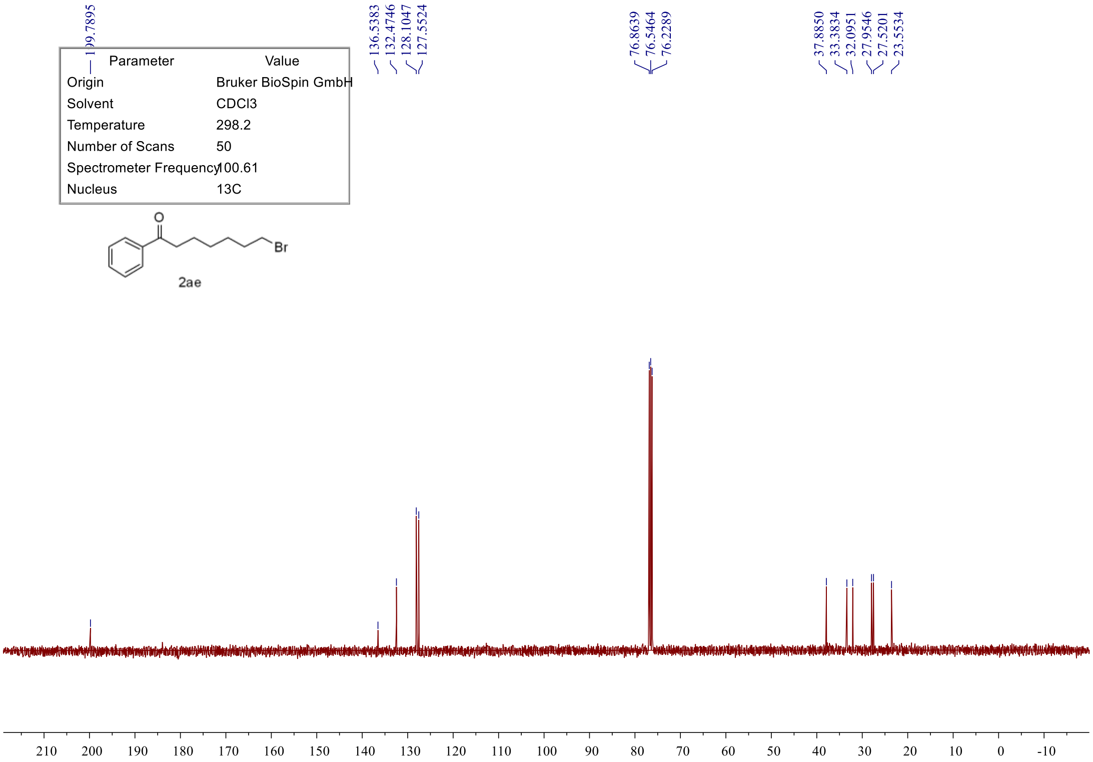
199.7895
136.5383
132.4746
128.1047
127.5524
76.8639
76.5464
76.2289
37.8850
33.3834
32.0951
27.9546
27.5201
23.5534
Parameter Value
Origin Bruker BioSpin GmbH
Solvent CDCl3
Temperature 298.2
Number of Scans 50
Spectrometer Frequency 100.61
Nucleus 13C
O
Br
2ae
210 200 190 180 170 160 150 140 130 120 110 100 90 80 70 60 50 40 30 20 10 0 -10

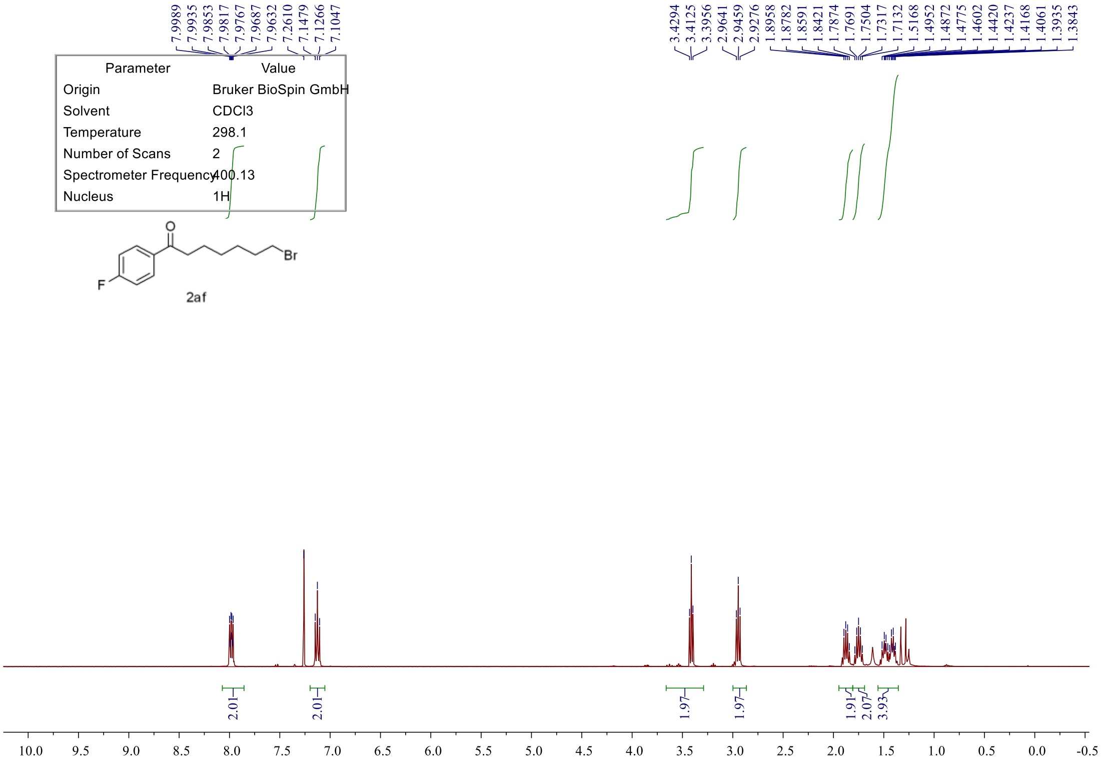
7.9989
7.9935
7.9853
7.9817
7.9767
7.9687
7.9632
7.2610
7.1479
7.1266
7.1047
3.4294
3.4125
3.3956
2.9641
2.9459
2.9276
1.8958
1.8782
1.8591
1.8421
1.7874
1.7691
1.7504
1.7317
1.7132
1.5168
1.4952
1.4872
1.4775
1.4602
1.4420
1.4237
1.4168
1.4061
1.3935
1.3843
Parameter Value
Origin Bruker BioSpin GmbH
Solvent CDCl3
Temperature 298.1
Number of Scans 2
Spectrometer Frequency 400.13
Nucleus 1H
O
F
Br
2af
2.01
2.01
1.97
1.97
1.91
2.07
3.93
10.0 9.5 9.0 8.5 8.0 7.5 7.0 6.5 6.0 5.5 5.0 4.5 4.0 3.5 3.0 2.5 2.0 1.5 1.0 0.5 0.0 -0.5

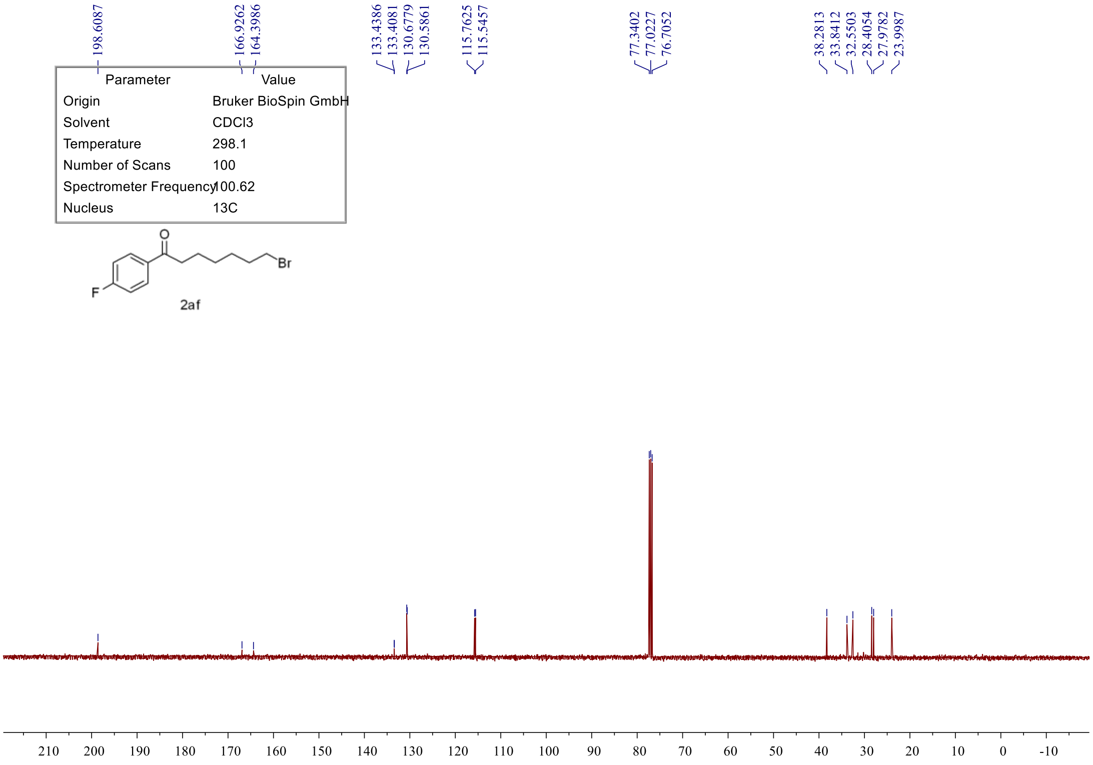
198.6087
166.9262
164.3986
133.4386
133.4081
130.6779
130.5861
115.7625
115.5457
77.3402
77.0227
76.7052
38.2813
33.8412
32.5503
28.4054
27.9782
23.9987
Parameter Value
Origin Bruker BioSpin GmbH
Solvent CDCl3
Temperature 298.1
Number of Scans 100
Spectrometer Frequency 100.62
Nucleus 13C
O
F
Br
2af
210 200 190 180 170 160 150 140 130 120 110 100 90 80 70 60 50 40 30 20 10 0 -10

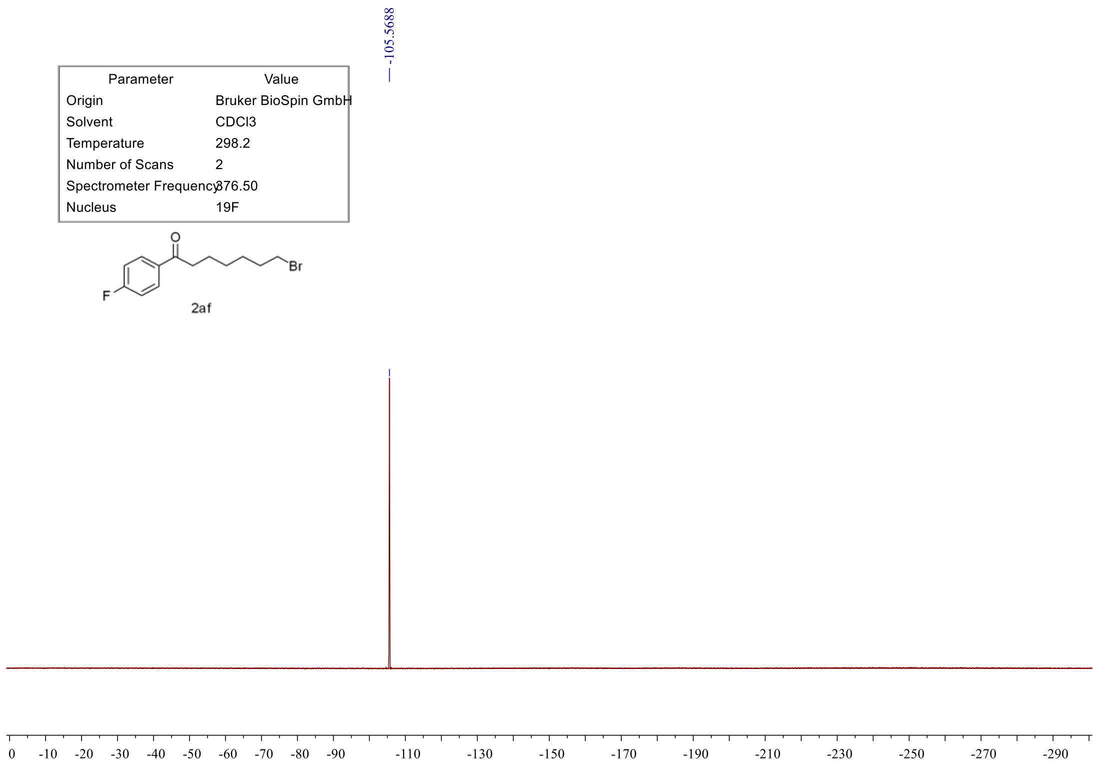

-105.5688
Parameter Value
Origin Bruker BioSpin GmbH
Solvent CDCl3
Temperature 298.2
Number of Scans 2
Spectrometer Frequency 376.50
Nucleus 19F
O
Br
F
2af
0 -10 -20 -30 -40 -50 -60 -70 -80 -90 -110 -130 -150 -170 -190 -210 -230 -250 -270 -290

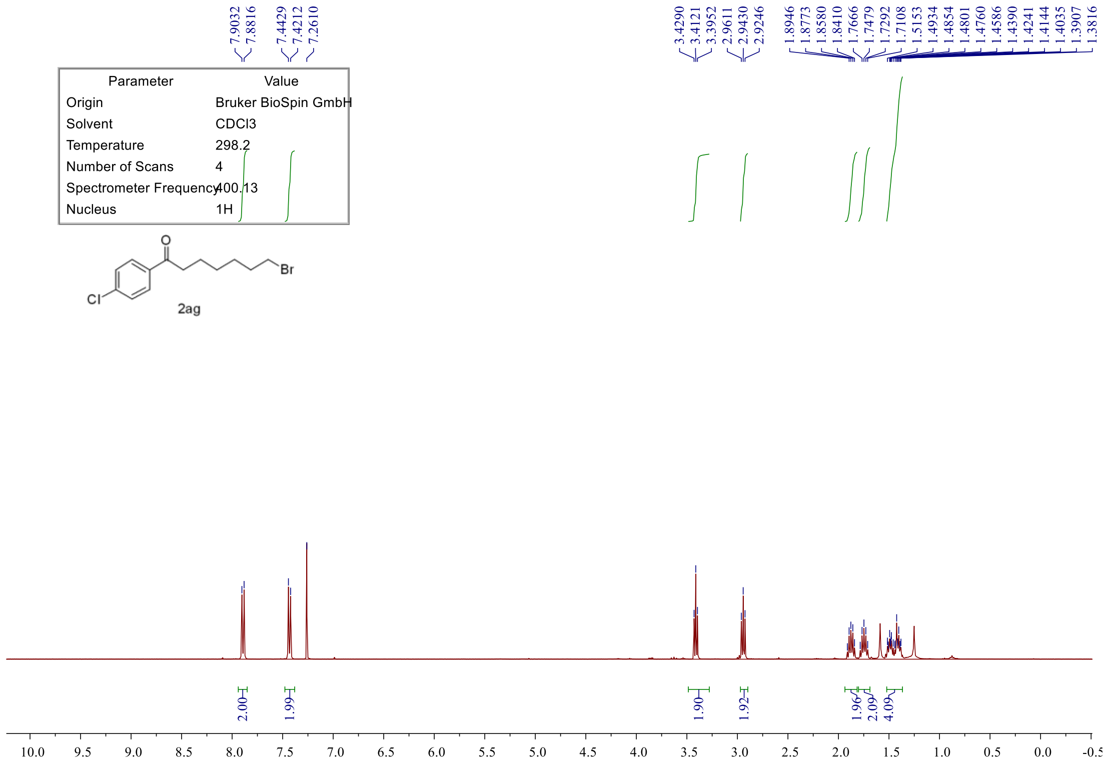

7.9032
7.8816
7.4429
7.4212
7.2610
3.4290
3.4121
3.3952
2.9611
2.9430
2.9246
1.8946
1.8773
1.8580
1.8410
1.7666
1.7479
1.7292
1.7108
1.5153
1.4934
1.4854
1.4801
1.4760
1.4586
1.4390
1.4241
1.4144
1.4035
1.3907
1.3816
Parameter Value
Origin Bruker BioSpin GmbH
Solvent CDCl3
Temperature 298.2
Number of Scans 4
Spectrometer Frequency 400.13
Nucleus 1H
O
Br
Cl
2ag
2.00
1.99
1.90
1.92
1.96
2.09
4.09
10.0 9.5 9.0 8.5 8.0 7.5 7.0 6.5 6.0 5.5 5.0 4.5 4.0 3.5 3.0 2.5 2.0 1.5 1.0 0.5 0.0 -0.5

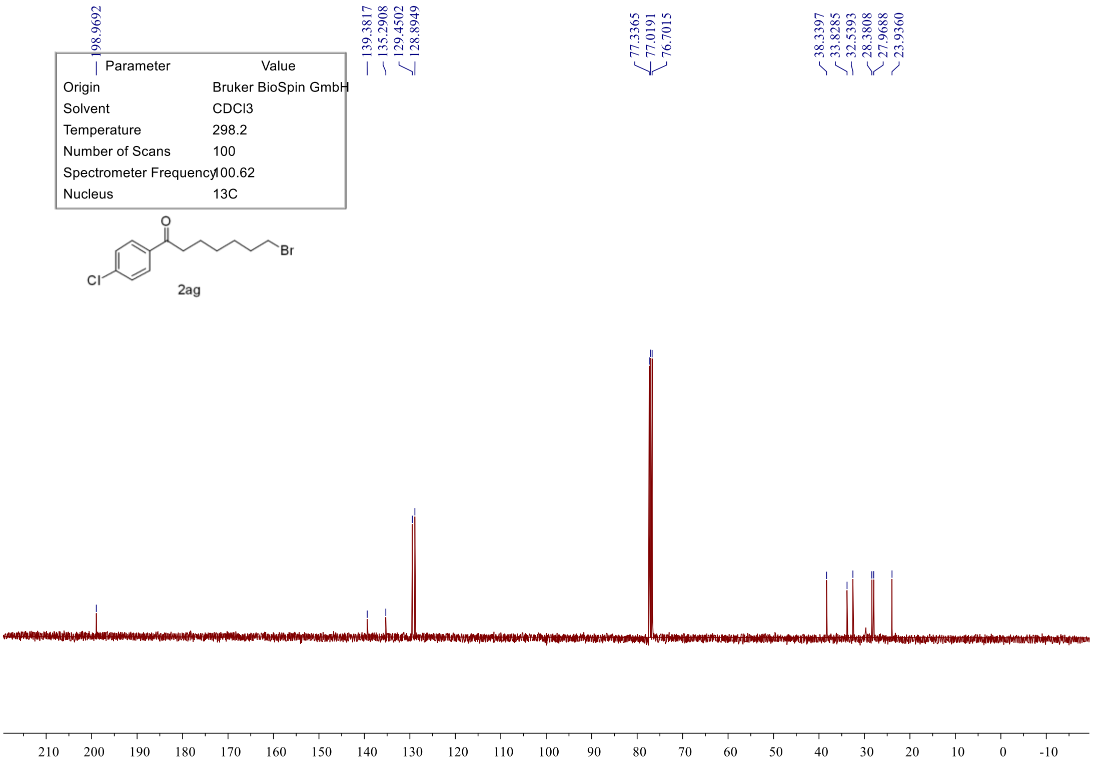

198.9692
139.3817
135.2908
129.4502
128.8949
77.3365
77.0191
76.7015
38.3397
33.8285
32.5393
28.3808
27.9688
23.9360
Parameter | Value
Origin | Bruker BioSpin GmbH
Solvent | CDCl3
Temperature | 298.2
Number of Scans | 100
Spectrometer Frequency | 100.62
Nucleus | 13C
O
Br
Cl
2ag
210 200 190 180 170 160 150 140 130 120 110 100 90 80 70 60 50 40 30 20 10 0 -10

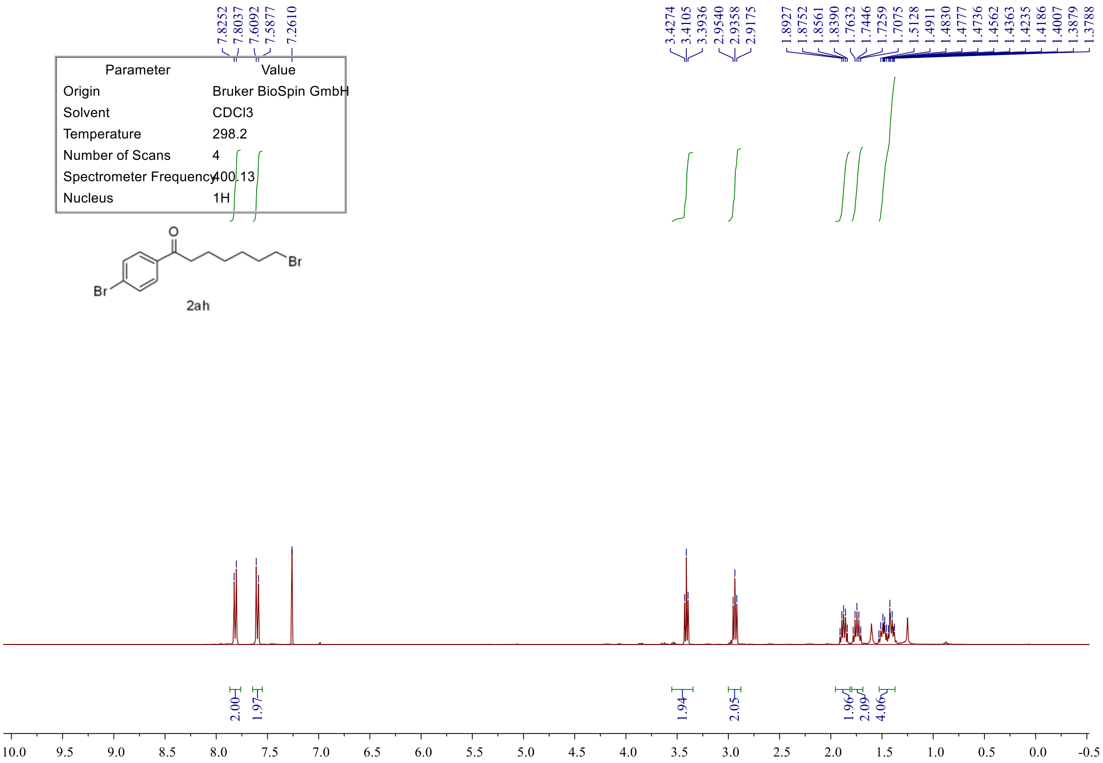

7.8252
7.8037
7.6092
7.5877
7.2610
3.4274
3.4105
3.3936
2.9540
2.9358
2.9175
1.8927
1.8752
1.8561
1.8390
1.7632
1.7446
1.7259
1.7075
1.5128
1.4911
1.4830
1.4777
1.4736
1.4562
1.4363
1.4235
1.4186
1.4007
1.3879
1.3788
Parameter | Value
Origin | Bruker BioSpin GmbH
Solvent | CDCl3
Temperature | 298.2
Number of Scans | 4
Spectrometer Frequency | 400.13
Nucleus | 1H
O
Br
Br
2ah
2.00
1.97
1.94
2.05
1.96
2.09
4.06
10.0 9.5 9.0 8.5 8.0 7.5 7.0 6.5 6.0 5.5 5.0 4.5 4.0 3.5 3.0 2.5 2.0 1.5 1.0 0.5 0.0 -0.5

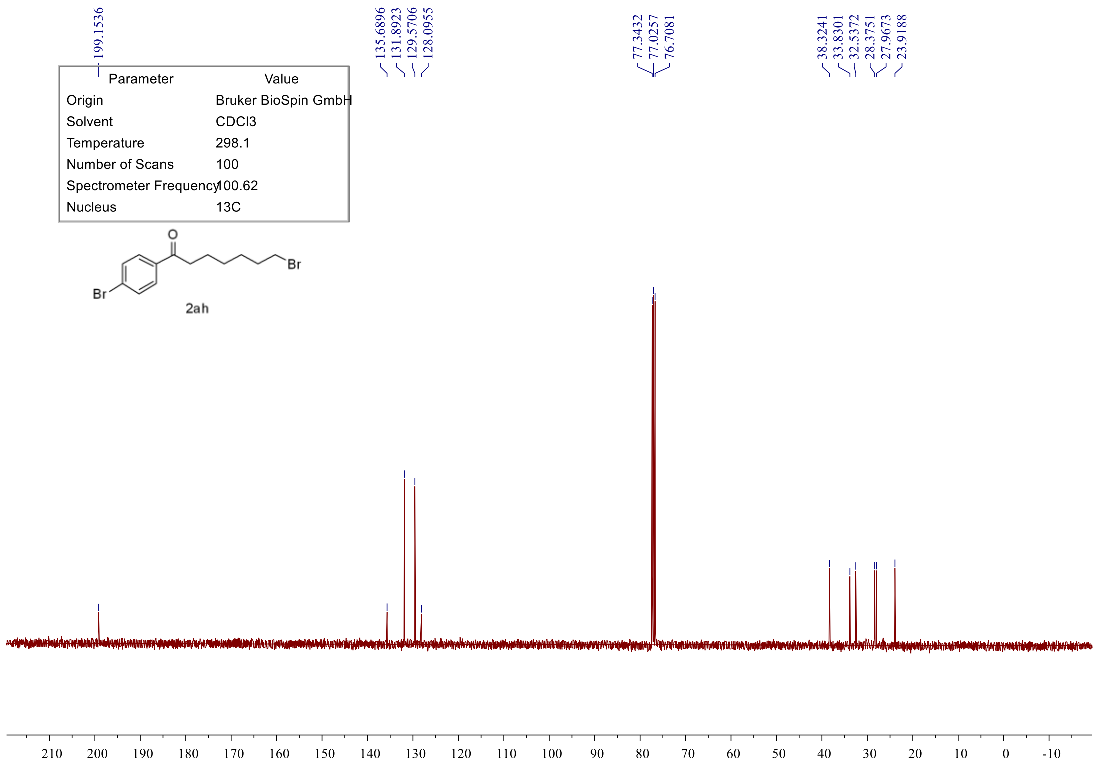

Parameter	Value
Origin	Bruker BioSpin GmbH
Solvent	CDCl3
Temperature	298.1
Number of Scans	100
Spectrometer Frequency	100.62
Nucleus	13C

2ah

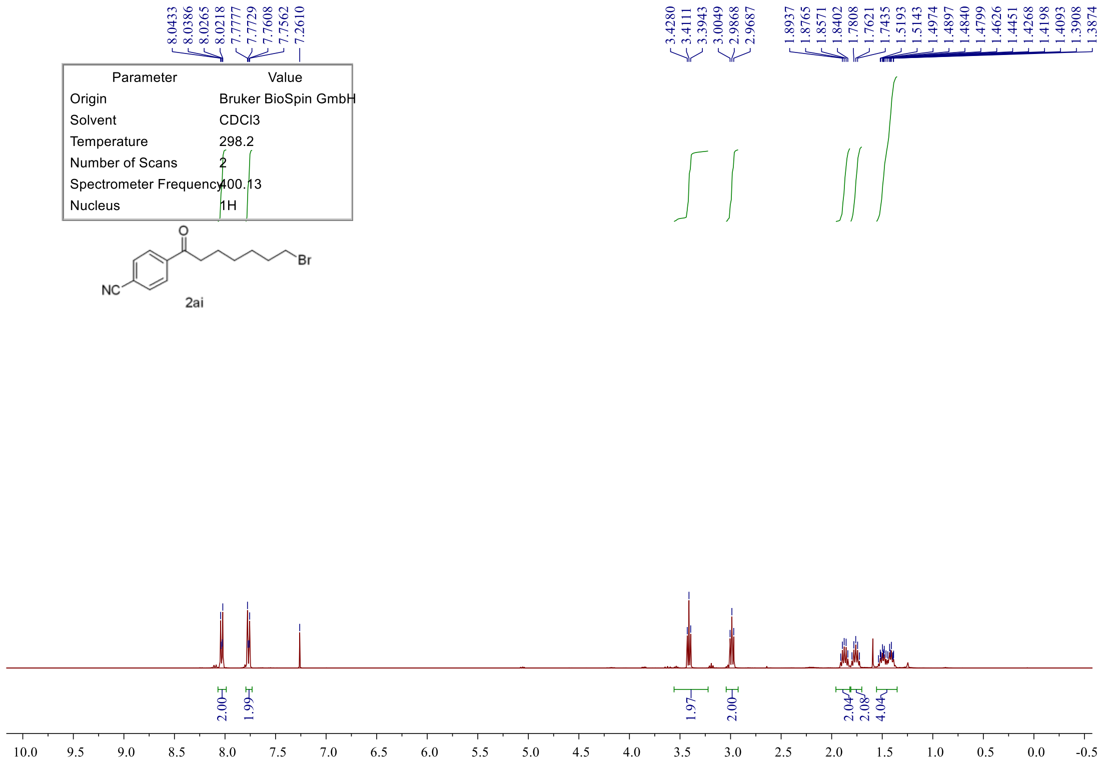

Parameter	Value
Origin	Bruker BioSpin GmbH
Solvent	CDCl3
Temperature	298.2
Number of Scans	2
Spectrometer Frequency	400.13
Nucleus	1H

2ai

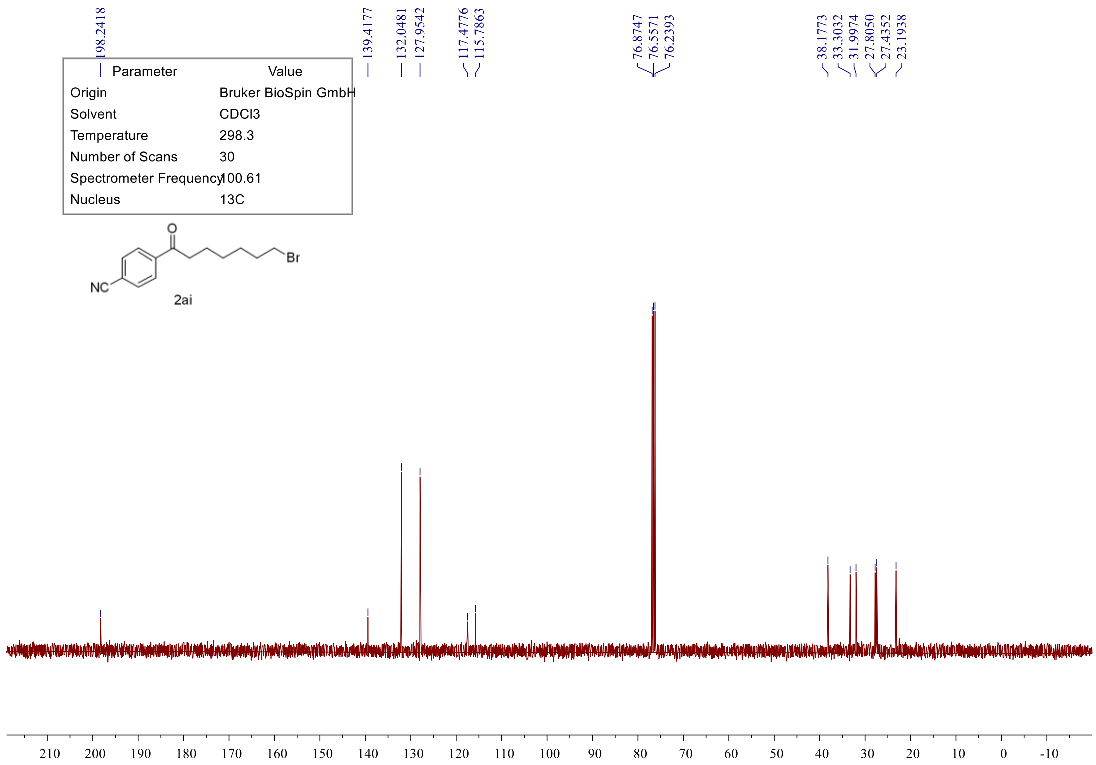
198.2418
139.4177
132.0481
127.9542
117.4776
115.7863
76.8747
76.5571
76.2393
38.1773
33.3032
31.9974
27.8050
27.4352
23.1938
Parameter Value
Origin Bruker BioSpin GmbH
Solvent CDCl3
Temperature 298.3
Number of Scans 30
Spectrometer Frequency 100.61
Nucleus 13C
O
Br
NC
2ai
210 200 190 180 170 160 150 140 130 120 110 100 90 80 70 60 50 40 30 20 10 0 -10

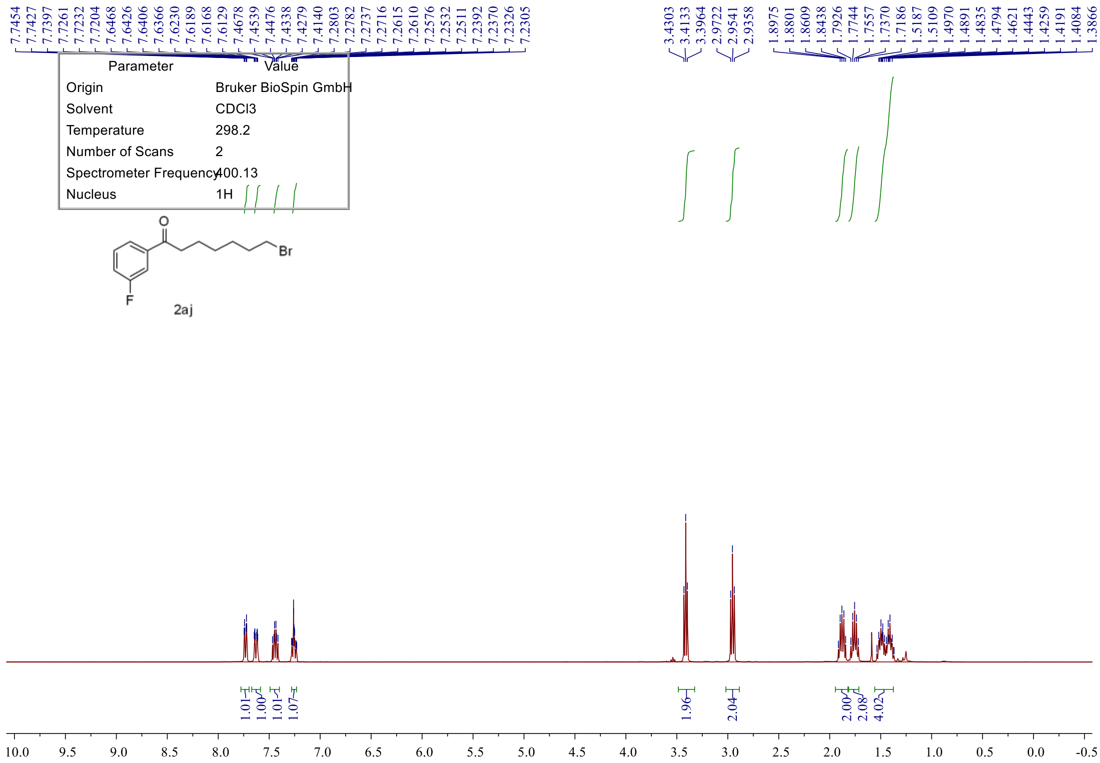
7.7454
7.7427
7.7397
7.7261
7.7232
7.7204
7.6468
7.6426
7.6406
7.6366
7.6230
7.6189
7.6168
7.6129
7.4678
7.4539
7.4476
7.4338
7.4279
7.4140
7.2803
7.2782
7.2737
7.2716
7.2615
7.2610
7.2576
7.2532
7.2511
7.2392
7.2370
7.2326
7.2305
3.4303
3.4133
3.3964
2.9722
2.9541
2.9358
1.8975
1.8801
1.8609
1.8438
1.7926
1.7744
1.7557
1.7370
1.7186
1.5187
1.5109
1.4970
1.4891
1.4835
1.4794
1.4621
1.4443
1.4259
1.4191
1.4084
1.3866
Parameter Value
Origin Bruker BioSpin GmbH
Solvent CDCl3
Temperature 298.2
Number of Scans 2
Spectrometer Frequency 400.13
Nucleus 1H
O
Br
F
2aj
1.01
1.00
1.01
1.07
1.96
2.04
2.00
2.08
4.02
10.0 9.5 9.0 8.5 8.0 7.5 7.0 6.5 6.0 5.5 5.0 4.5 4.0 3.5 3.0 2.5 2.0 1.5 1.0 0.5 0.0 -0.5

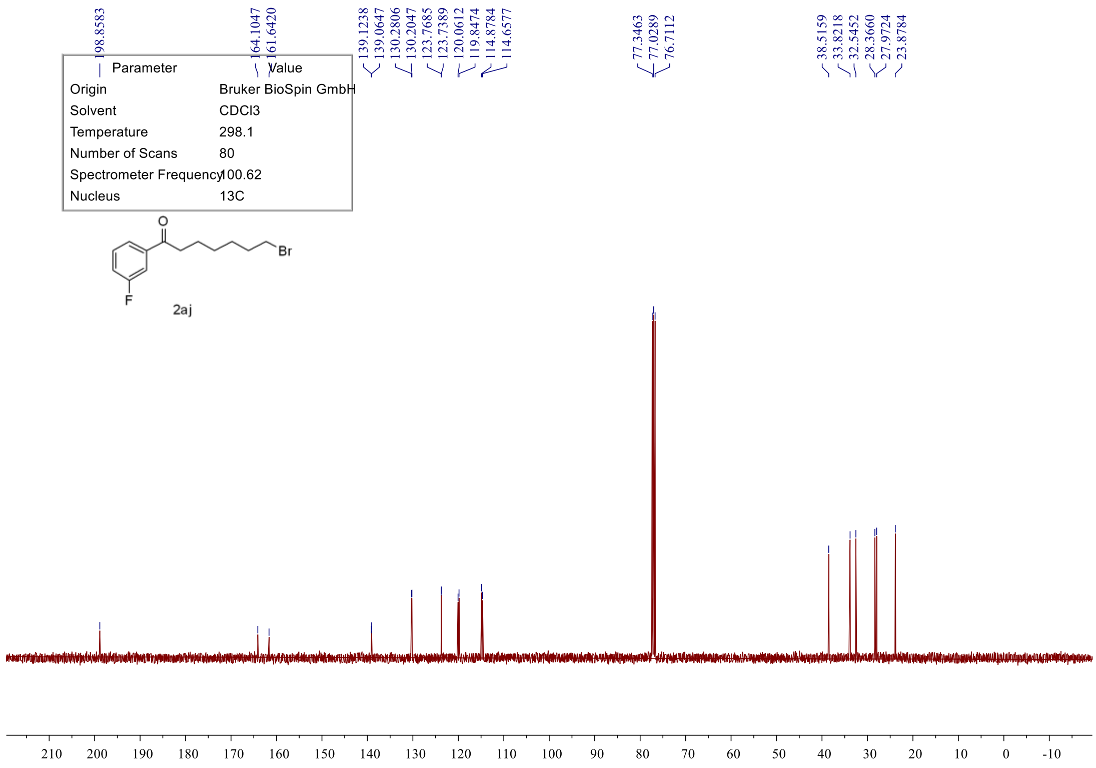

Parameter	Value
Origin	Bruker BioSpin GmbH
Solvent	CDCl3
Temperature	298.1
Number of Scans	80
Spectrometer Frequency	100.62
Nucleus	13C

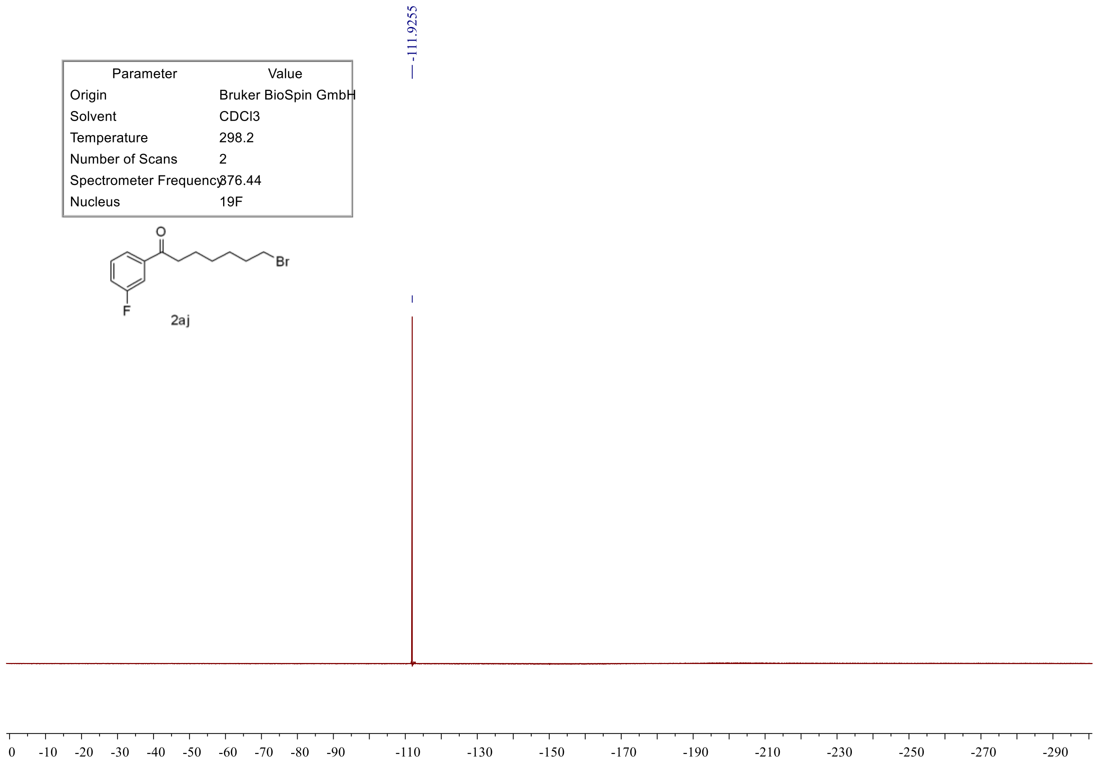

Parameter	Value
Origin	Bruker BioSpin GmbH
Solvent	CDCl3
Temperature	298.2
Number of Scans	2
Spectrometer Frequency	376.44
Nucleus	19F

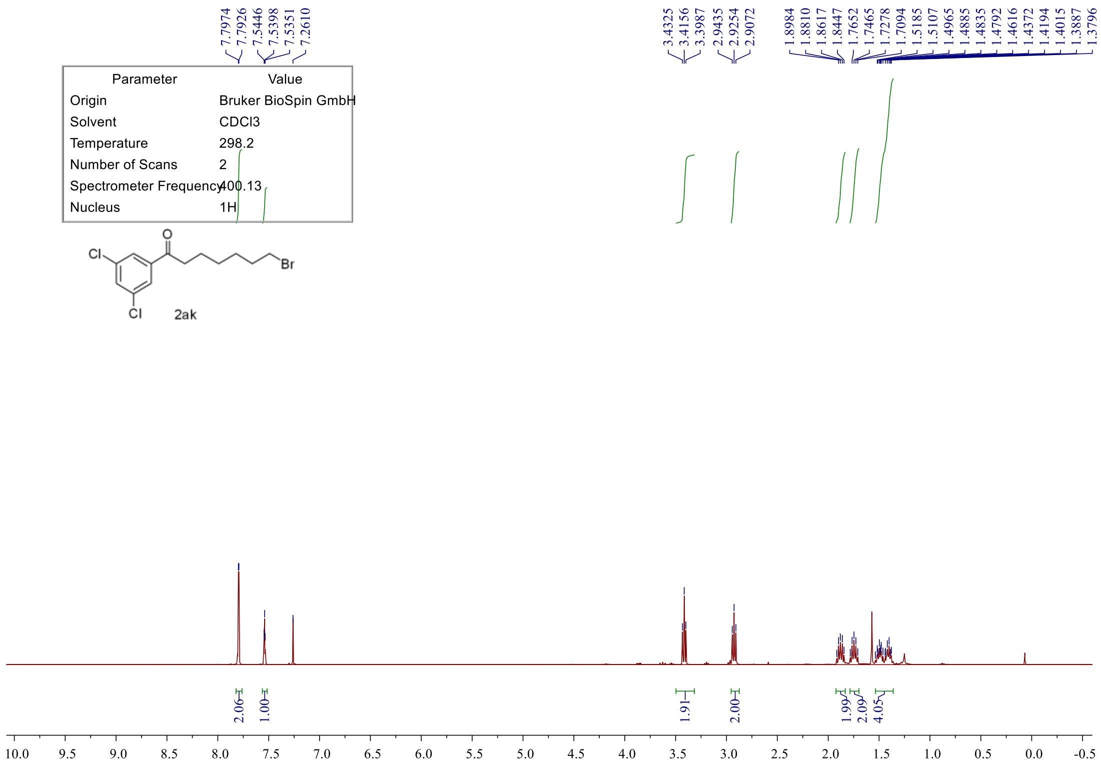
7.7974
7.7926
7.5446
7.5398
7.5351
7.2610
3.4325
3.4156
3.3987
2.9435
2.9254
2.9072
1.8984
1.8810
1.8617
1.8447
1.7652
1.7465
1.7278
1.7094
1.5185
1.5107
1.4965
1.4885
1.4835
1.4792
1.4616
1.4372
1.4194
1.4015
1.3887
1.3796
Parameter Value
Origin Bruker BioSpin GmbH
Solvent CDCl3
Temperature 298.2
Number of Scans 2
Spectrometer Frequency 400.13
Nucleus 1H
O
Cl
Br
Cl
2ak
2.06
1.00
1.91
2.00
1.99
2.09
4.05
10.0 9.5 9.0 8.5 8.0 7.5 7.0 6.5 6.0 5.5 5.0 4.5 4.0 3.5 3.0 2.5 2.0 1.5 1.0 0.5 0.0 -0.5

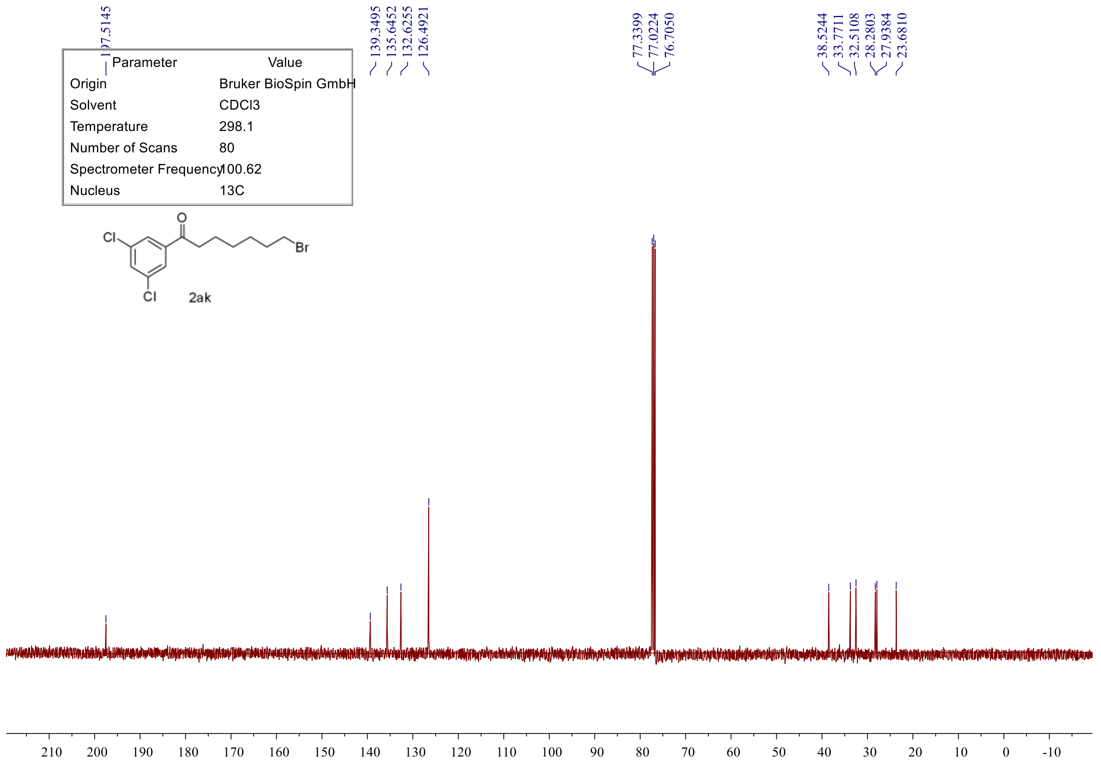
197.5145
139.3495
135.6452
132.6255
126.4921
77.3399
77.0224
76.7050
38.5244
33.7711
32.5108
28.2803
27.9384
23.6810
Parameter Value
Origin Bruker BioSpin GmbH
Solvent CDCl3
Temperature 298.1
Number of Scans 80
Spectrometer Frequency 100.62
Nucleus 13C
O
Cl
Br
Cl
2ak
210 200 190 180 170 160 150 140 130 120 110 100 90 80 70 60 50 40 30 20 10 0 -10

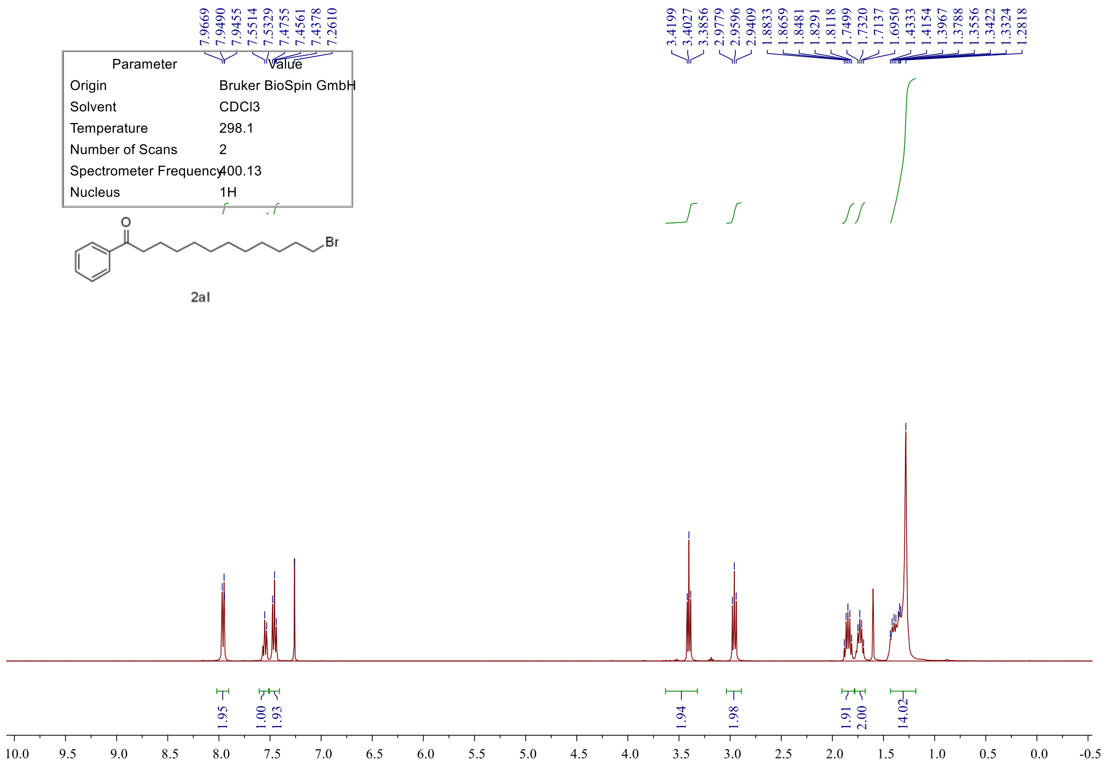
7.9669
7.9490
7.9455
7.5514
7.5329
7.4755
7.4561
7.4378
7.2610
3.4199
3.4027
3.3856
2.9779
2.9596
2.9409
1.8833
1.8659
1.8481
1.8291
1.8118
1.7499
1.7320
1.7137
1.6950
1.4333
1.4154
1.3967
1.3788
1.3556
1.3422
1.3324
1.2818
Parameter
Value
Origin
Bruker BioSpin GmbH
Solvent
CDCl3
Temperature
298.1
Number of Scans
2
Spectrometer Frequency
400.13
Nucleus
1H
Br
2al
1.95
1.00
1.93
1.94
1.98
1.91
2.00
14.02
10.0 9.5 9.0 8.5 8.0 7.5 7.0 6.5 6.0 5.5 5.0 4.5 4.0 3.5 3.0 2.5 2.0 1.5 1.0 0.5 0.0 -0.5

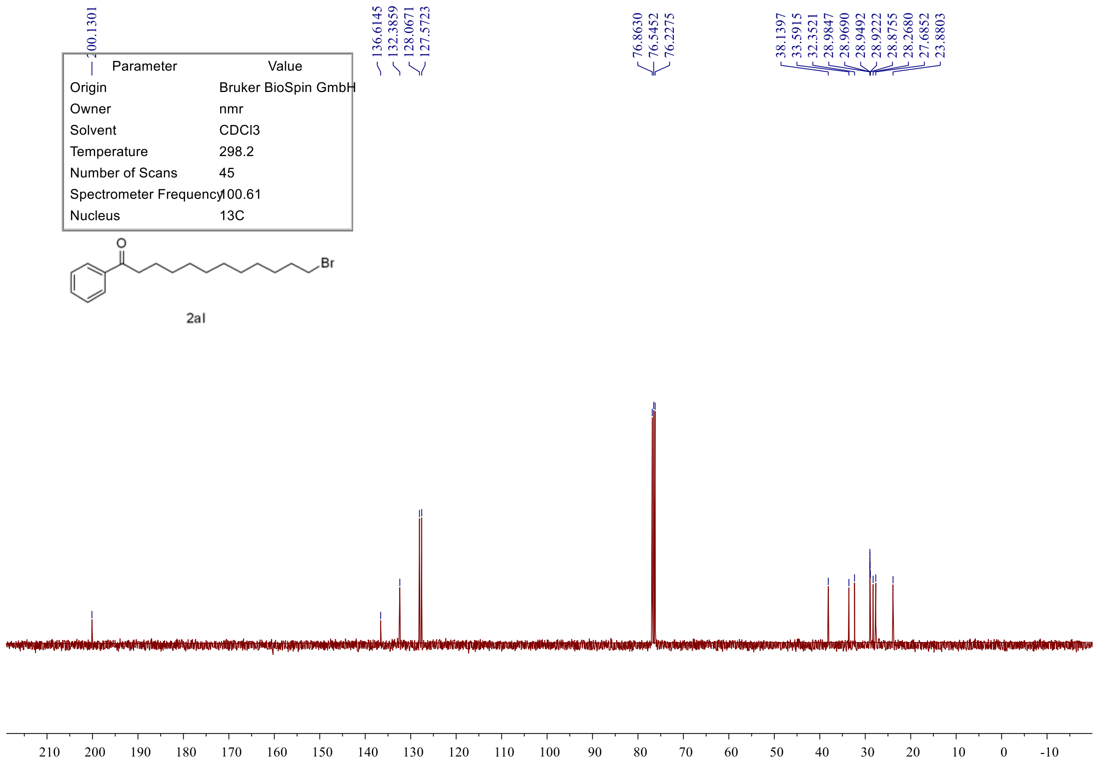
200.1301
136.6145
132.3859
128.0671
127.5723
76.8630
76.5452
76.2275
38.1397
33.5915
32.3521
28.9847
28.9690
28.9492
28.9222
28.8755
28.2680
27.6852
23.8803
Parameter
Value
Origin
Bruker BioSpin GmbH
Owner
nmr
Solvent
CDCl3
Temperature
298.2
Number of Scans
45
Spectrometer Frequency
100.61
Nucleus
13C
Br
2al
210 200 190 180 170 160 150 140 130 120 110 100 90 80 70 60 50 40 30 20 10 0 -10

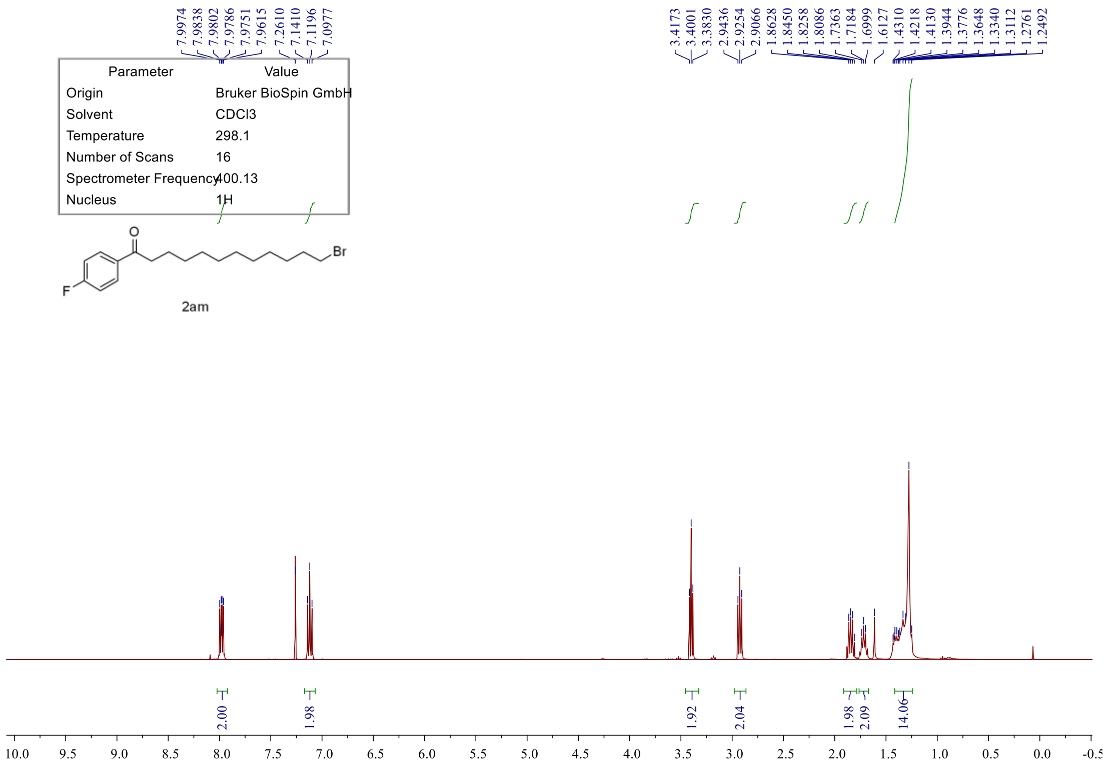

7.9974
7.9838
7.9802
7.9786
7.9751
7.9615
7.2610
7.1410
7.1196
7.0977
3.4173
3.4001
3.3830
2.9436
2.9254
2.9066
1.8628
1.8450
1.8258
1.8086
1.7363
1.7184
1.6999
1.6127
1.4310
1.4218
1.4130
1.3944
1.3776
1.3648
1.3340
1.3112
1.2761
1.2492
Parameter
Value
Origin
Bruker BioSpin GmbH
Solvent
CDCl3
Temperature
298.1
Number of Scans
16
Spectrometer Frequency
400.13
Nucleus
1H
O
F
Br
2am
2.00
1.98
1.92
2.04
1.98
2.09
14.06

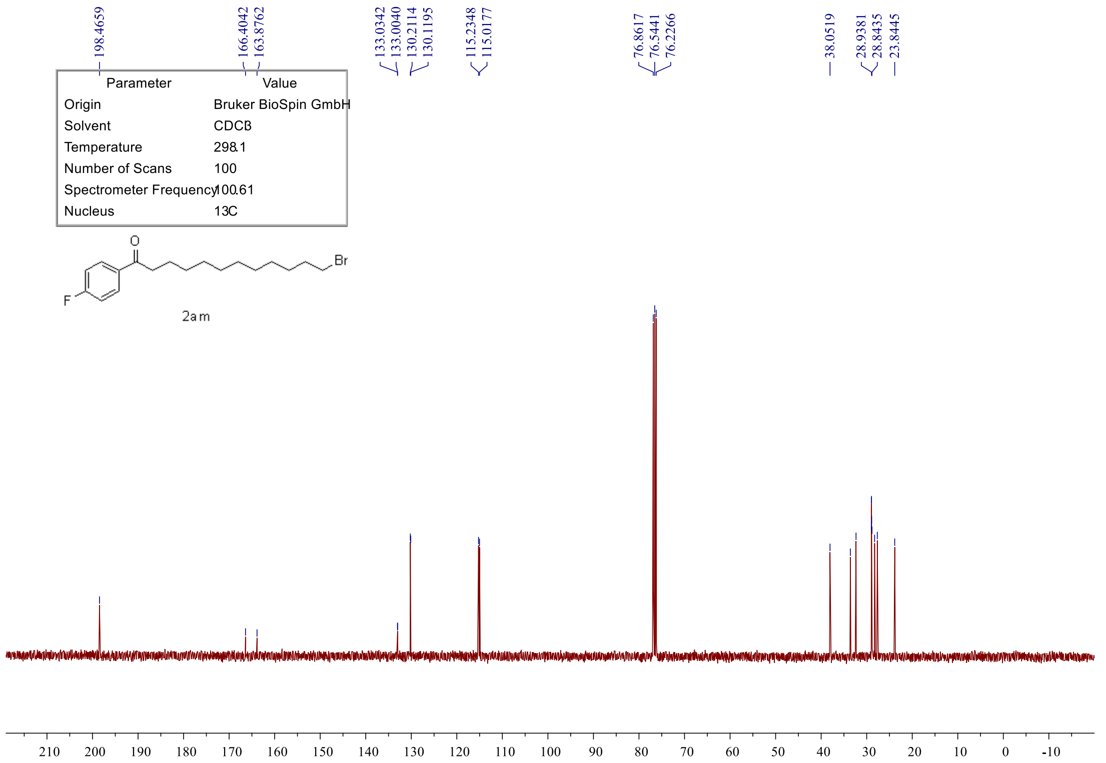

198.4659
166.4042
163.8762
133.0342
133.0040
130.2114
130.1195
115.2348
115.0177
76.8617
76.5441
76.2266
38.0519
28.9381
28.8435
23.8445
Parameter
Value
Origin
Bruker BioSpin GmbH
Solvent
CDCl3
Temperature
298.1
Number of Scans
100
Spectrometer Frequency
100.61
Nucleus
13C
O
F
Br
2am

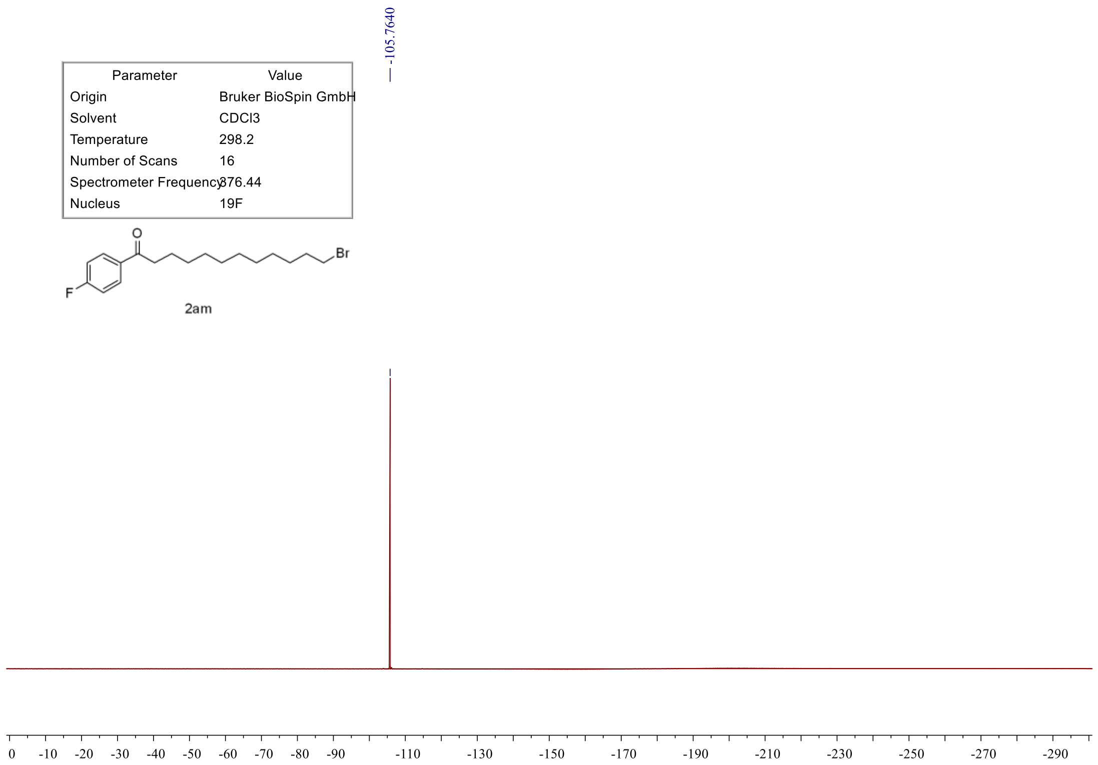
-105.7640
Parameter Value
Origin Bruker BioSpin GmbH
Solvent CDCl3
Temperature 298.2
Number of Scans 16
Spectrometer Frequency 376.44
Nucleus 19F
O
Br
F
2am
0 -10 -20 -30 -40 -50 -60 -70 -80 -90 -110 -130 -150 -170 -190 -210 -230 -250 -270 -290

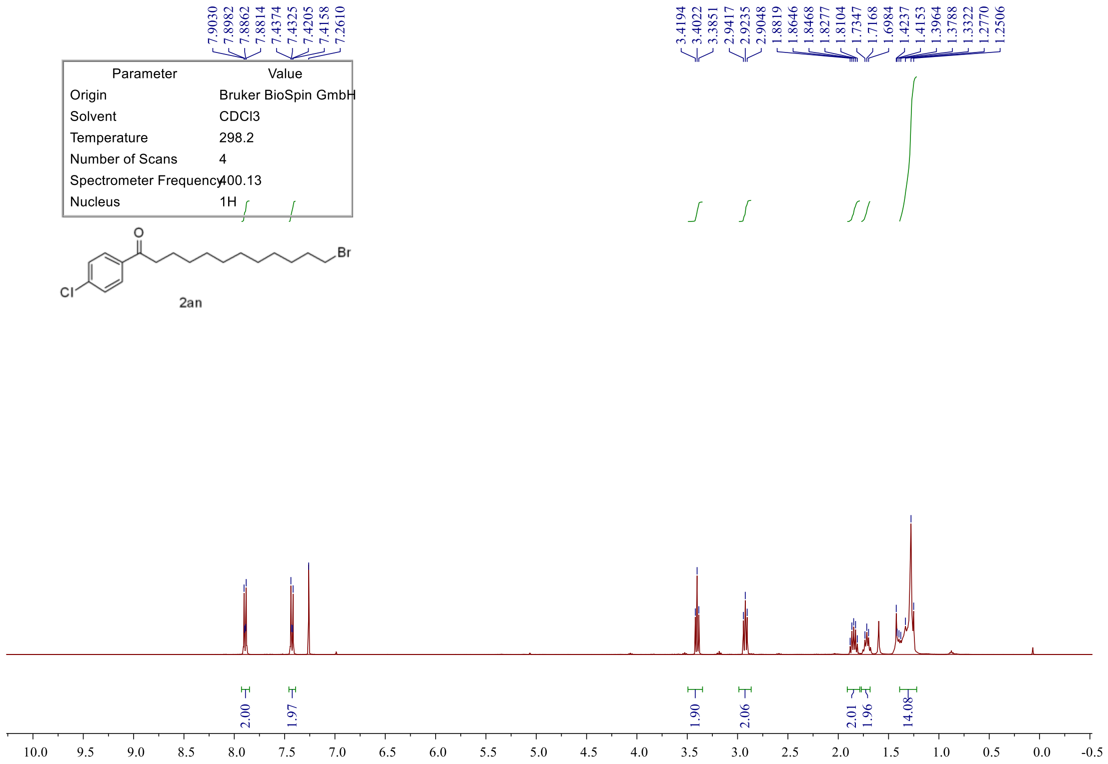
7.9030
7.8982
7.8862
7.8814
7.4374
7.4325
7.4205
7.4158
7.2610
3.4194
3.4022
3.3851
2.9417
2.9235
2.9048
1.8819
1.8646
1.8468
1.8277
1.8104
1.7347
1.7168
1.6984
1.4237
1.4153
1.3964
1.3788
1.3322
1.2770
1.2506
Parameter Value
Origin Bruker BioSpin GmbH
Solvent CDCl3
Temperature 298.2
Number of Scans 4
Spectrometer Frequency 400.13
Nucleus 1H
O
Br
Cl
2an
2.00
1.97
1.90
2.06
2.01
1.96
14.08
10.0 9.5 9.0 8.5 8.0 7.5 7.0 6.5 6.0 5.5 5.0 4.5 4.0 3.5 3.0 2.5 2.0 1.5 1.0 0.5 0.0 -0.5

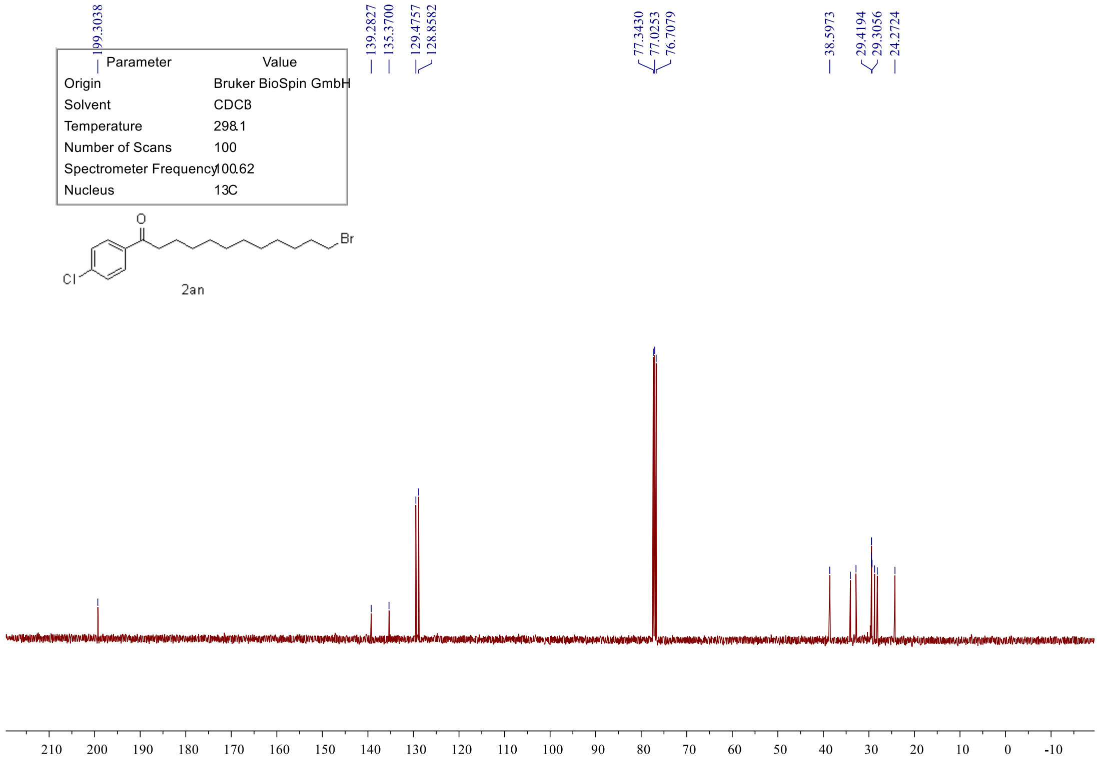

199.3038
139.2827
135.3700
129.4757
128.8582
77.3430
77.0253
76.7079
38.5973
29.4194
29.3056
24.2724
Parameter Value
Origin Bruker BioSpin GmbH
Solvent CDCl3
Temperature 298.1
Number of Scans 100
Spectrometer Frequency 100.62
Nucleus 13C
O
Br
Cl
2an
210 200 190 180 170 160 150 140 130 120 110 100 90 80 70 60 50 40 30 20 10 0 -10

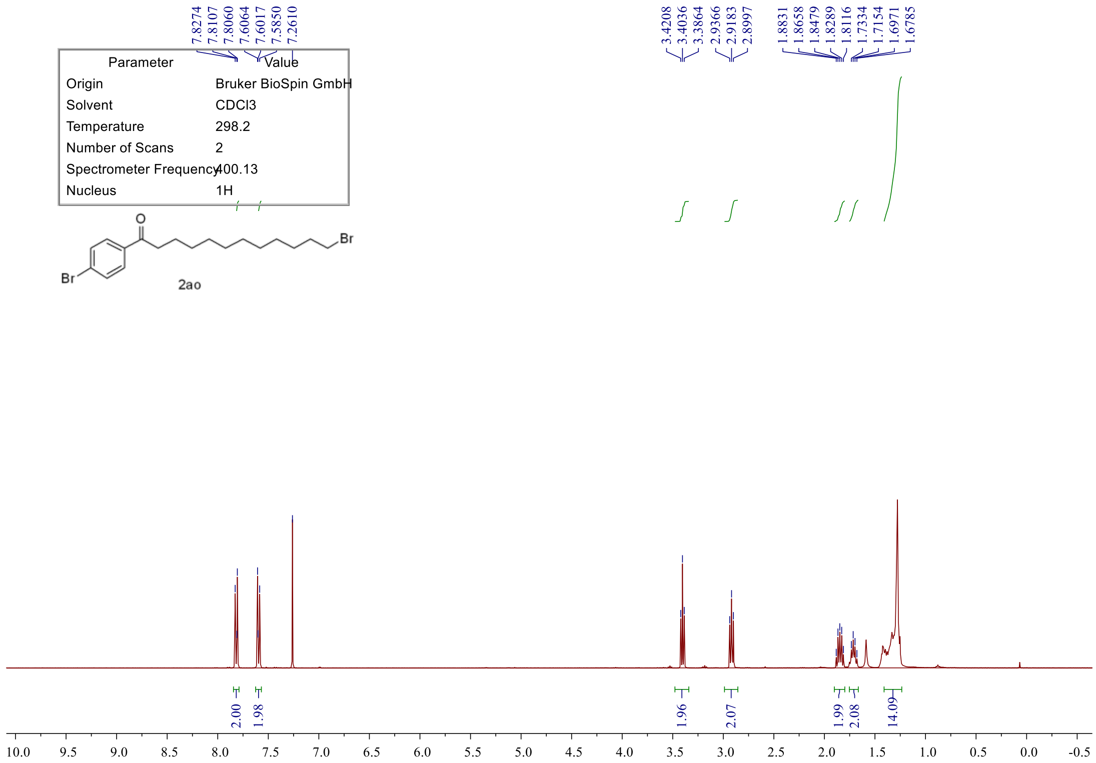

7.8274
7.8107
7.8060
7.6064
7.6017
7.5850
7.2610
3.4208
3.4036
3.3864
2.9366
2.9183
2.8997
1.8831
1.8658
1.8479
1.8289
1.8116
1.7334
1.7154
1.6971
1.6785
Parameter Value
Origin Bruker BioSpin GmbH
Solvent CDCl3
Temperature 298.2
Number of Scans 2
Spectrometer Frequency 400.13
Nucleus 1H
O
Br
Br
2ao
2.00
1.98
1.96
2.07
1.99
2.08
14.09
10.0 9.5 9.0 8.5 8.0 7.5 7.0 6.5 6.0 5.5 5.0 4.5 4.0 3.5 3.0 2.5 2.0 1.5 1.0 0.5 0.0 -0.5

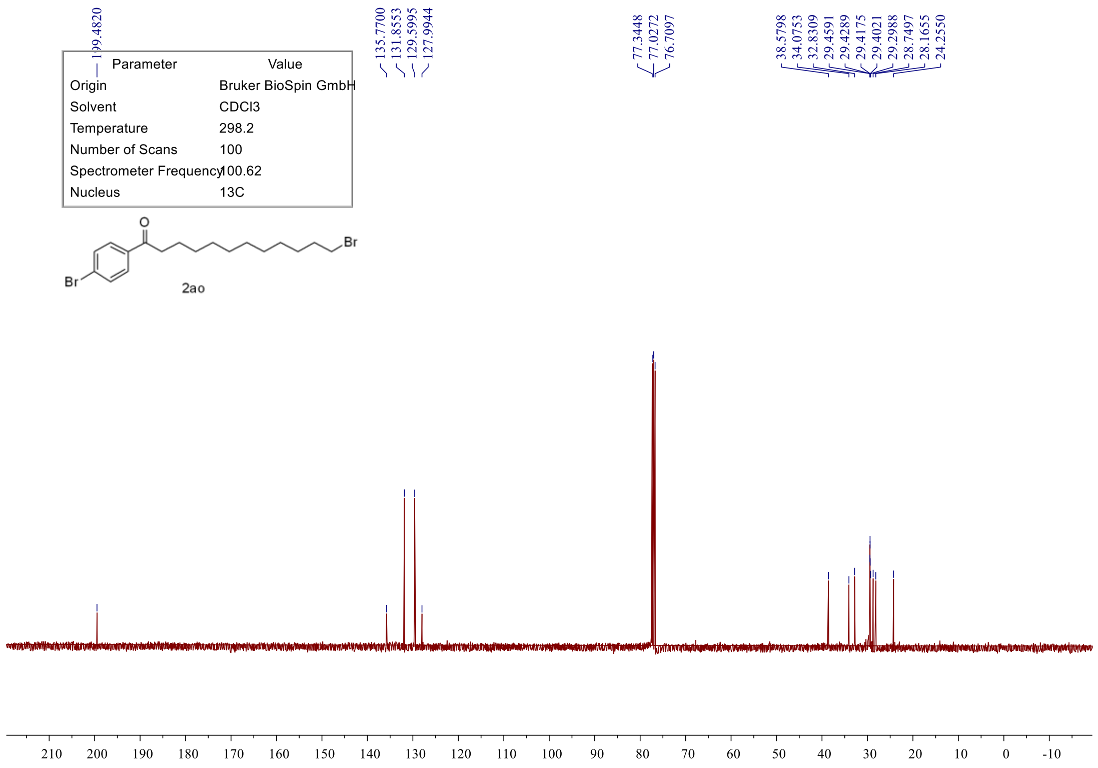
199.4820
135.7700
131.8553
129.5995
127.9944
77.3448
77.0272
76.7097
38.5798
34.0753
32.8309
29.4591
29.4289
29.4175
29.4021
29.2988
28.7497
28.1655
24.2550
Parameter Value
Origin Bruker BioSpin GmbH
Solvent CDCl3
Temperature 298.2
Number of Scans 100
Spectrometer Frequency 100.62
Nucleus 13C
O
Br
Br
2ao
210 200 190 180 170 160 150 140 130 120 110 100 90 80 70 60 50 40 30 20 10 0 -10

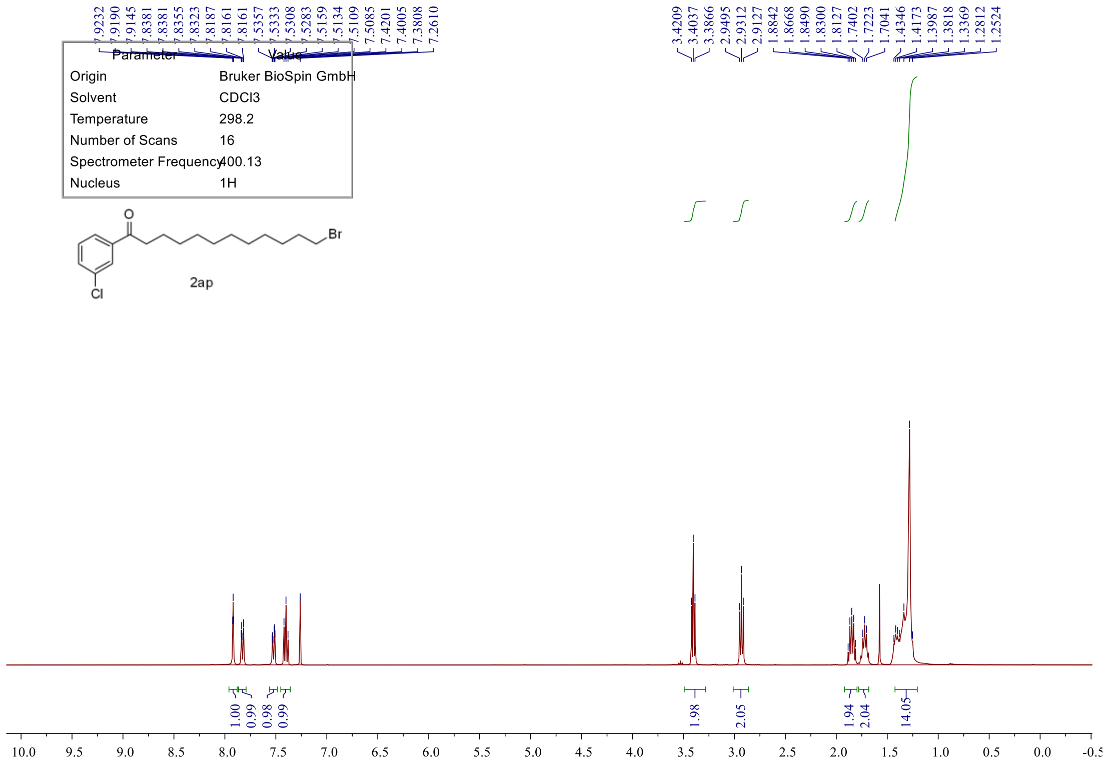
7.9232
7.9190
7.9145
7.8381
7.8381
7.8355
7.8323
7.8187
7.8161
7.8161
7.5357
7.5333
7.5308
7.5283
7.5159
7.5134
7.5109
7.5085
7.4201
7.4005
7.3808
7.2610
3.4209
3.4037
3.3866
2.9495
2.9312
2.9127
1.8842
1.8668
1.8490
1.8300
1.8127
1.7402
1.7223
1.7041
1.4346
1.4173
1.3987
1.3818
1.3369
1.2812
1.2524
Parameter Value
Origin Bruker BioSpin GmbH
Solvent CDCl3
Temperature 298.2
Number of Scans 16
Spectrometer Frequency 400.13
Nucleus 1H
O
Br
Cl
2ap
1.00
0.99
0.98
0.99
1.98
2.05
1.94
2.04
14.05
10.0 9.5 9.0 8.5 8.0 7.5 7.0 6.5 6.0 5.5 5.0 4.5 4.0 3.5 3.0 2.5 2.0 1.5 1.0 0.5 0.0 -0.5

Parameter	Value
Origin	Bruker BioSpin GmbH
Solvent	CDCl3
Temperature	298.1
Number of Scans	80
Spectrometer Frequency	100.62
Nucleus	13C

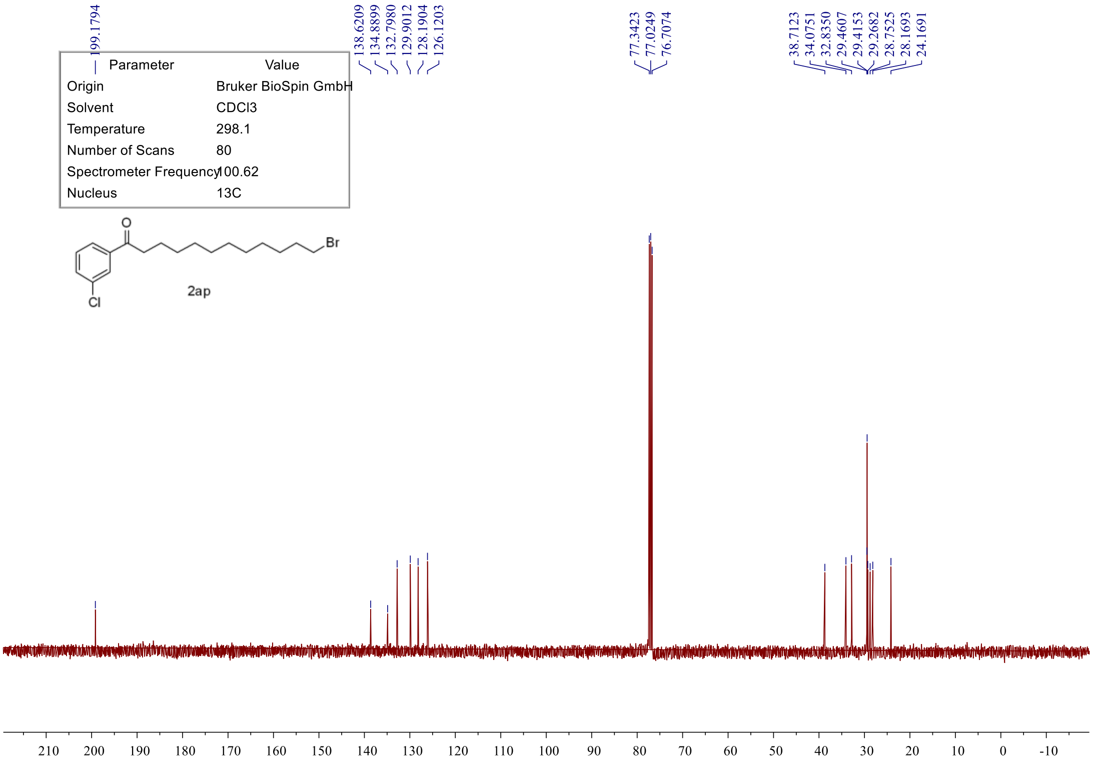

2ap

Parameter	Value
Origin	Bruker BioSpin GmbH
Solvent	CDCl3
Temperature	298.2
Number of Scans	2
Spectrometer Frequency	400.13
Nucleus	1H

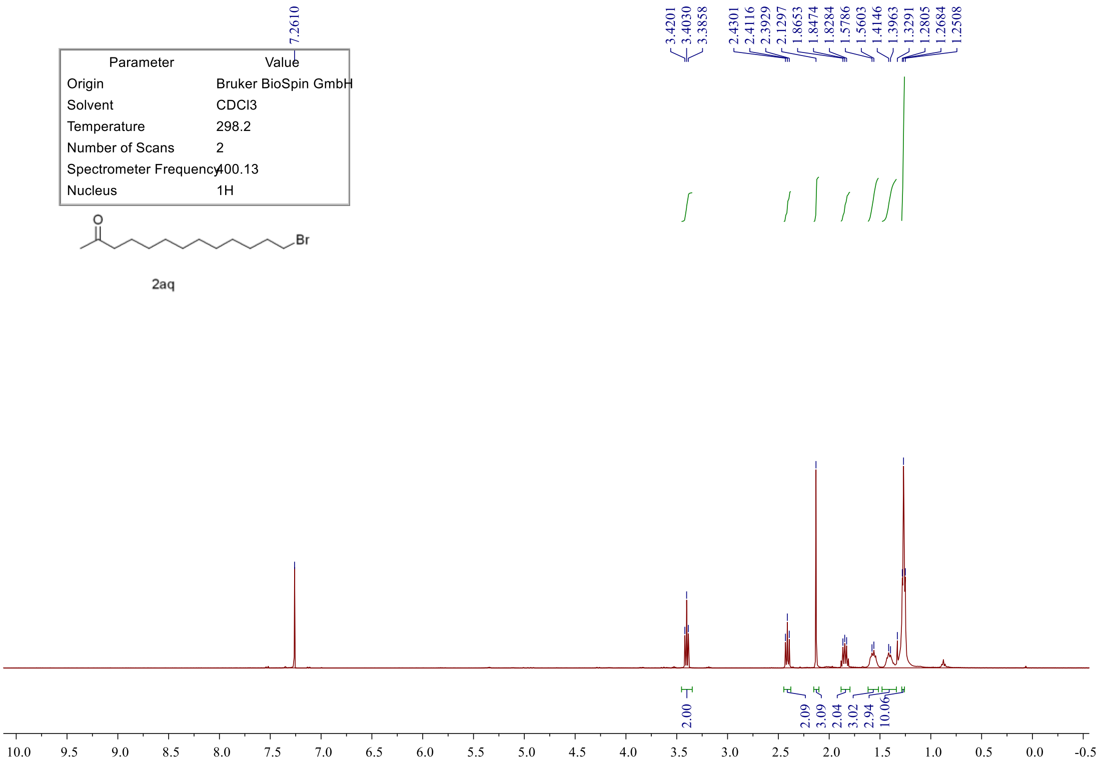

2aq

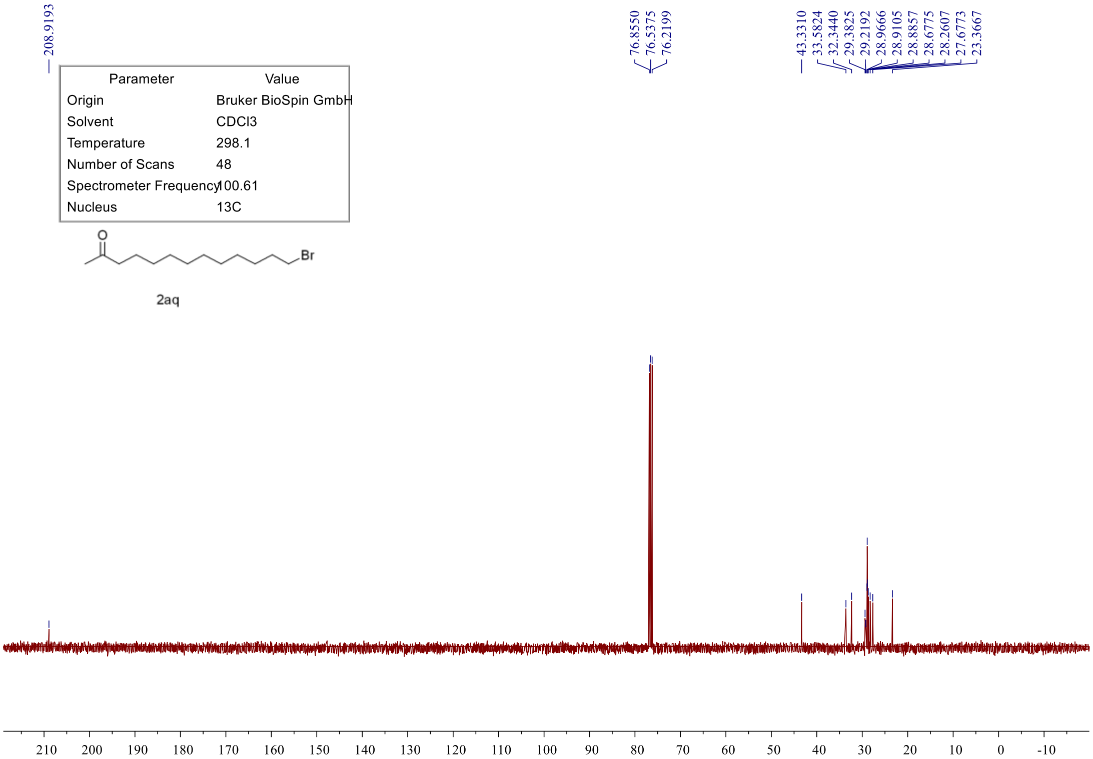
208.9193
76.8550
76.5375
76.2199
43.3310
33.5824
32.3440
29.3825
29.2192
28.9666
28.9105
28.8857
28.6775
28.2607
27.6773
23.3667
Parameter | Value
Origin | Bruker BioSpin GmbH
Solvent | CDCl3
Temperature | 298.1
Number of Scans | 48
Spectrometer Frequency | 100.61
Nucleus | 13C
O
Br
2aq
210 200 190 180 170 160 150 140 130 120 110 100 90 80 70 60 50 40 30 20 10 0 -10

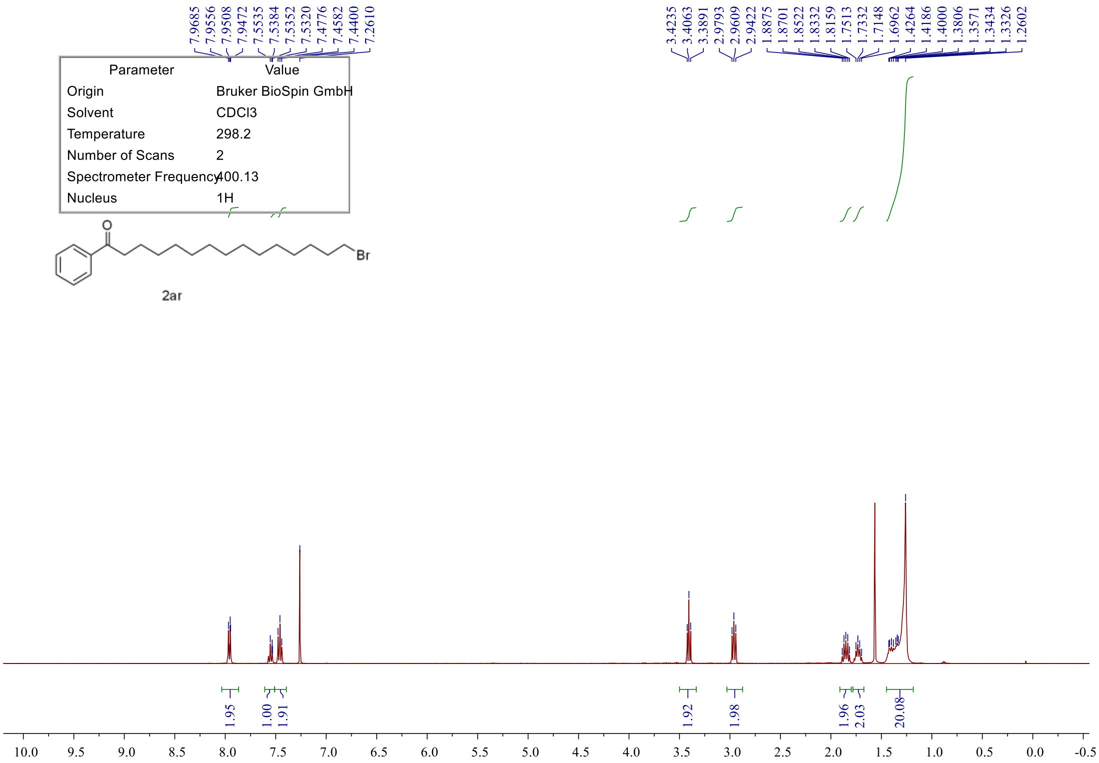
7.9685
7.9556
7.9508
7.9472
7.5535
7.5384
7.5352
7.5320
7.4776
7.4582
7.4400
7.2610
3.4235
3.4063
3.3891
2.9793
2.9609
2.9422
1.8875
1.8701
1.8522
1.8332
1.8159
1.7513
1.7332
1.7148
1.6962
1.4264
1.4186
1.4000
1.3806
1.3571
1.3434
1.3326
1.2602
Parameter | Value
Origin | Bruker BioSpin GmbH
Solvent | CDCl3
Temperature | 298.2
Number of Scans | 2
Spectrometer Frequency | 400.13
Nucleus | 1H
O
Br
2ar
1.95
1.00
1.91
1.92
1.98
1.96
2.03
20.08
10.0 9.5 9.0 8.5 8.0 7.5 7.0 6.5 6.0 5.5 5.0 4.5 4.0 3.5 3.0 2.5 2.0 1.5 1.0 0.5 0.0 -0.5

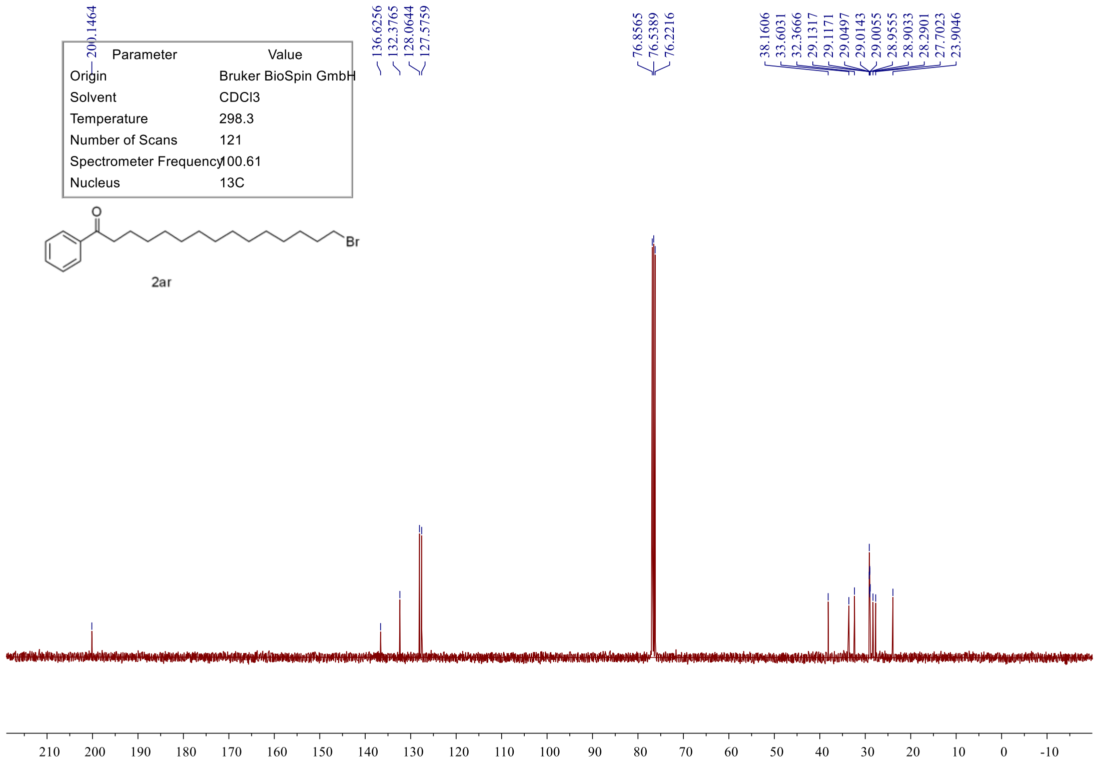
200.1464
136.6256
132.3765
128.0644
127.5759
76.8565
76.5389
76.2216
38.1606
33.6031
32.3666
29.1317
29.1171
29.0497
29.0143
29.0055
28.9555
28.9033
28.2901
27.7023
23.9046
Parameter Value
Origin Bruker BioSpin GmbH
Solvent CDCl3
Temperature 298.3
Number of Scans 121
Spectrometer Frequency 100.61
Nucleus 13C
O
Br
2ar
210 200 190 180 170 160 150 140 130 120 110 100 90 80 70 60 50 40 30 20 10 0 -10

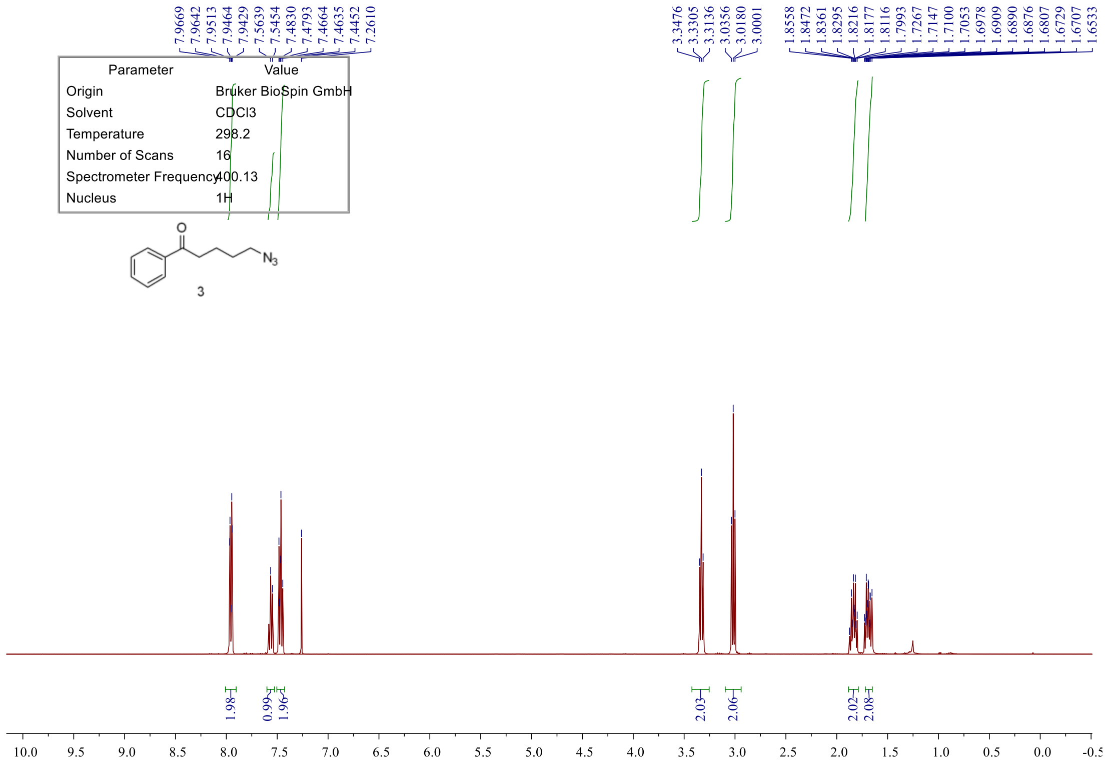
7.9669
7.9642
7.9513
7.9464
7.9429
7.5639
7.5454
7.4830
7.4793
7.4664
7.4635
7.4452
7.2610
3.3476
3.3305
3.3136
3.0356
3.0180
3.0001
1.8558
1.8472
1.8361
1.8295
1.8216
1.8177
1.8116
1.7993
1.7267
1.7147
1.7100
1.7053
1.6978
1.6909
1.6890
1.6876
1.6807
1.6729
1.6707
1.6533
Parameter Value
Origin Bruker BioSpin GmbH
Solvent CDCl3
Temperature 298.2
Number of Scans 16
Spectrometer Frequency 400.13
Nucleus 1H
O
N_3
3
1.98
0.99
1.96
2.03
2.06
2.02
2.08
10.0 9.5 9.0 8.5 8.0 7.5 7.0 6.5 6.0 5.5 5.0 4.5 4.0 3.5 3.0 2.5 2.0 1.5 1.0 0.5 0.0 -0.5

Parameter	Value
Origin	Bruker BioSpin GmbH
Solvent	CDCl3
Temperature	298.1
Number of Scans	100
Spectrometer Frequency	100.62
Nucleus	13C

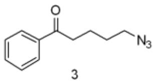

3

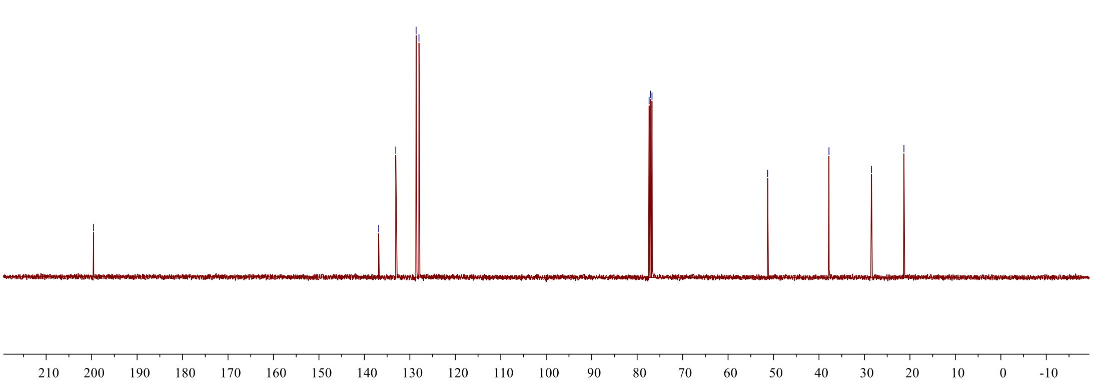

Parameter	Value
Origin	Bruker BioSpin GmbH
Solvent	CDCl3
Temperature	298.1
Number of Scans	4
Spectrometer Frequency	400.13
Nucleus	1H

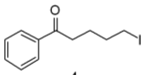

4

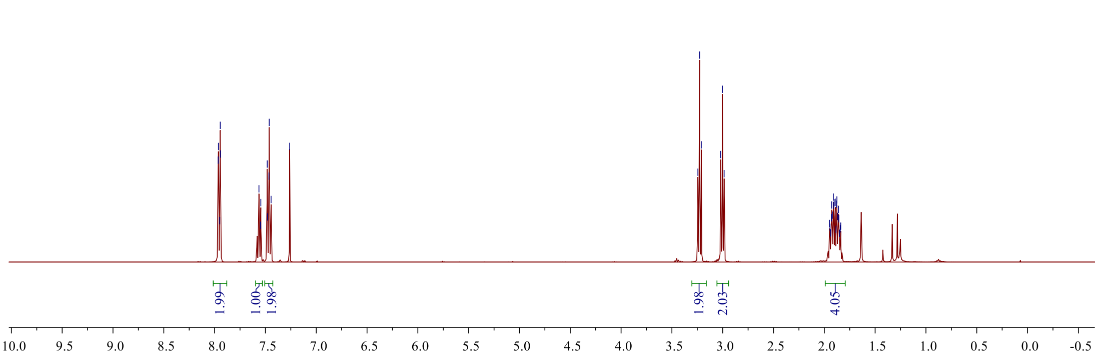

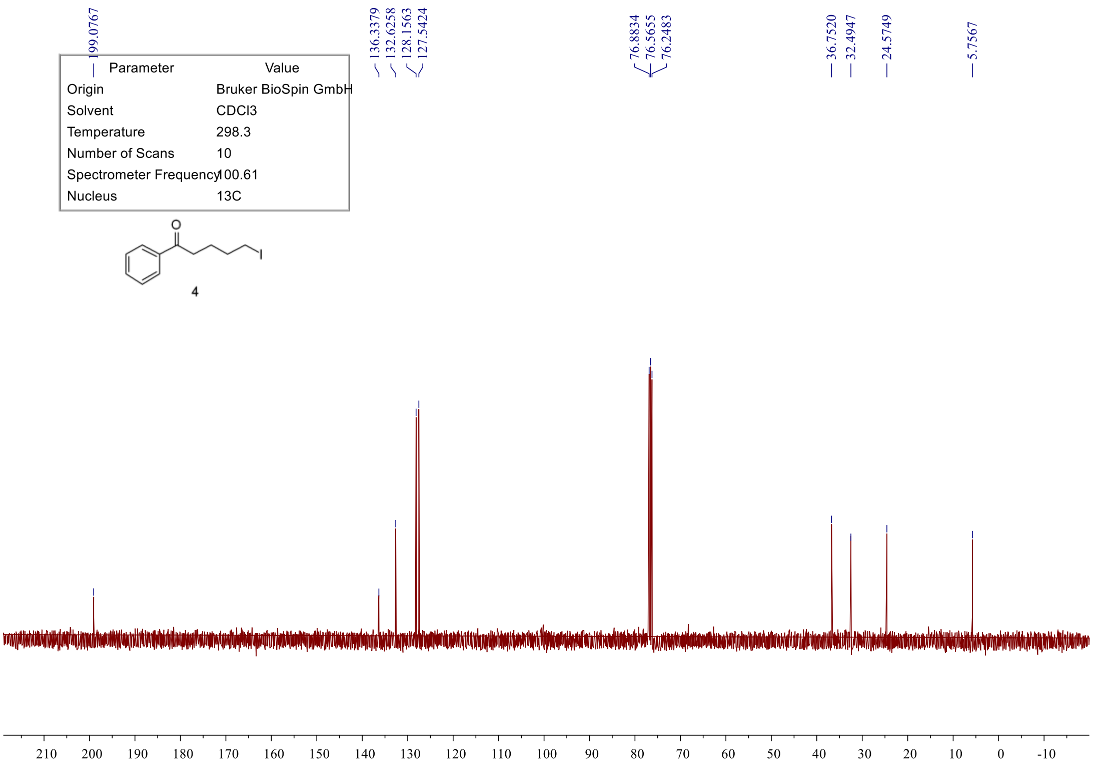
199.0767
136.3379
132.6258
128.1563
127.5424
76.8834
76.5655
76.2483
36.7520
32.4947
24.5749
5.7567
Parameter Value
Origin Bruker BioSpin GmbH
Solvent CDCl3
Temperature 298.3
Number of Scans 10
Spectrometer Frequency 100.61
Nucleus 13C
4
210 200 190 180 170 160 150 140 130 120 110 100 90 80 70 60 50 40 30 20 10 0 -10

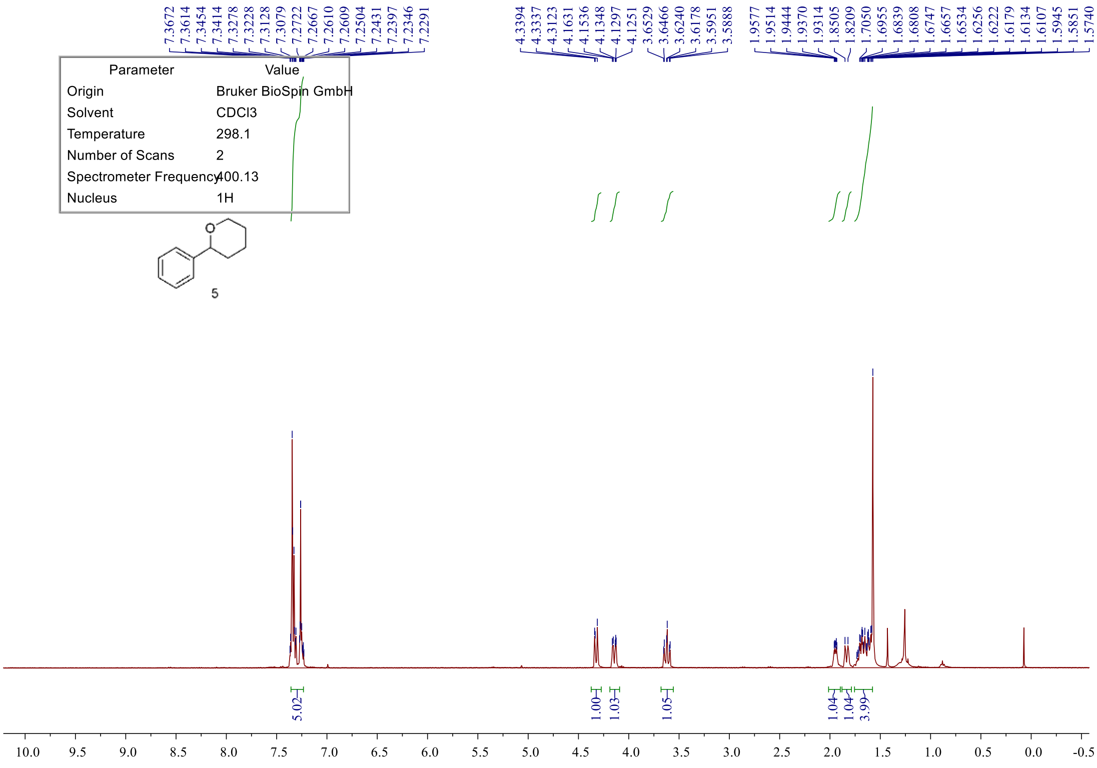
7.3672
7.3614
7.3454
7.3414
7.3278
7.3228
7.3128
7.3079
7.2722
7.2667
7.2610
7.2609
7.2504
7.2431
7.2397
7.2346
7.2291
4.3394
4.3337
4.3123
4.1631
4.1536
4.1348
4.1297
4.1251
3.6529
3.6466
3.6240
3.6178
3.5951
3.5888
1.9577
1.9514
1.9444
1.9370
1.9314
1.8505
1.8209
1.7050
1.6955
1.6839
1.6808
1.6747
1.6657
1.6534
1.6256
1.6222
1.6179
1.6134
1.6107
1.5945
1.5851
1.5740
Parameter Value
Origin Bruker BioSpin GmbH
Solvent CDCl3
Temperature 298.1
Number of Scans 2
Spectrometer Frequency 400.13
Nucleus 1H
5
5.02
1.00
1.03
1.05
1.04
1.04
3.99
10.0 9.5 9.0 8.5 8.0 7.5 7.0 6.5 6.0 5.5 5.0 4.5 4.0 3.5 3.0 2.5 2.0 1.5 1.0 0.5 0.0 -0.5

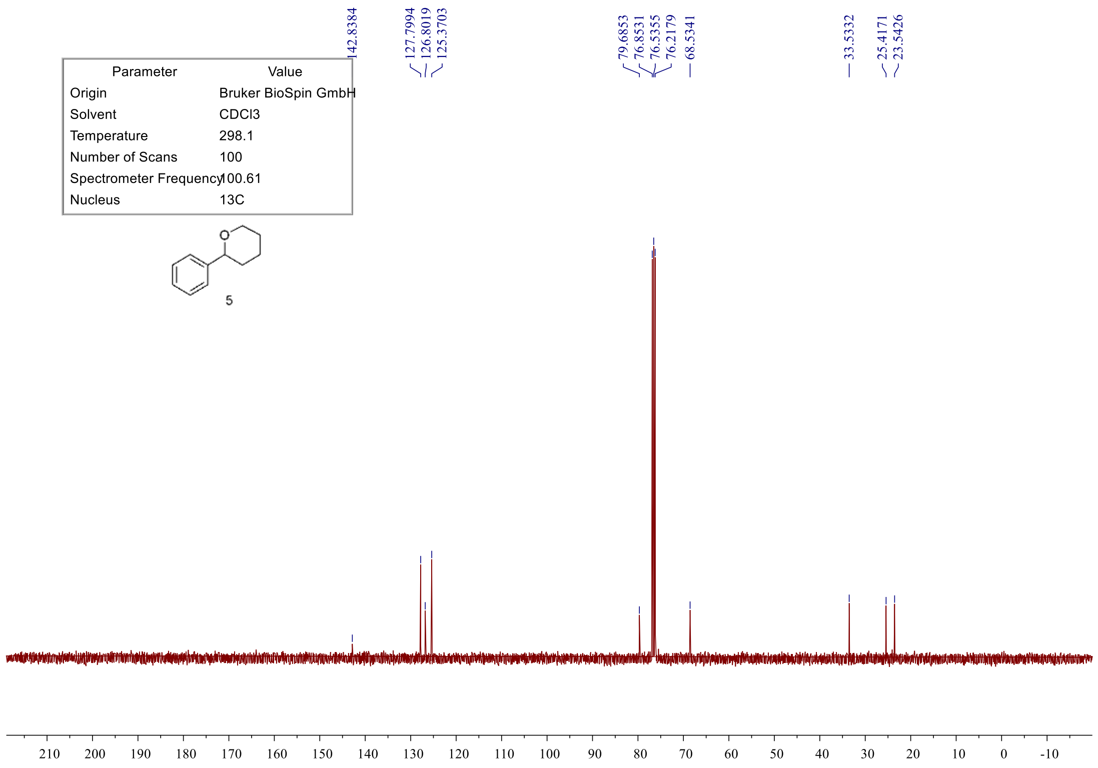
142.8384
127.7994
126.8019
125.3703
79.6853
76.8531
76.5355
76.2179
68.5341
33.5332
25.4171
23.5426
Parameter Value
Origin Bruker BioSpin GmbH
Solvent CDCl3
Temperature 298.1
Number of Scans 100
Spectrometer Frequency 100.61
Nucleus 13C
O
5
210 200 190 180 170 160 150 140 130 120 110 100 90 80 70 60 50 40 30 20 10 0 -10

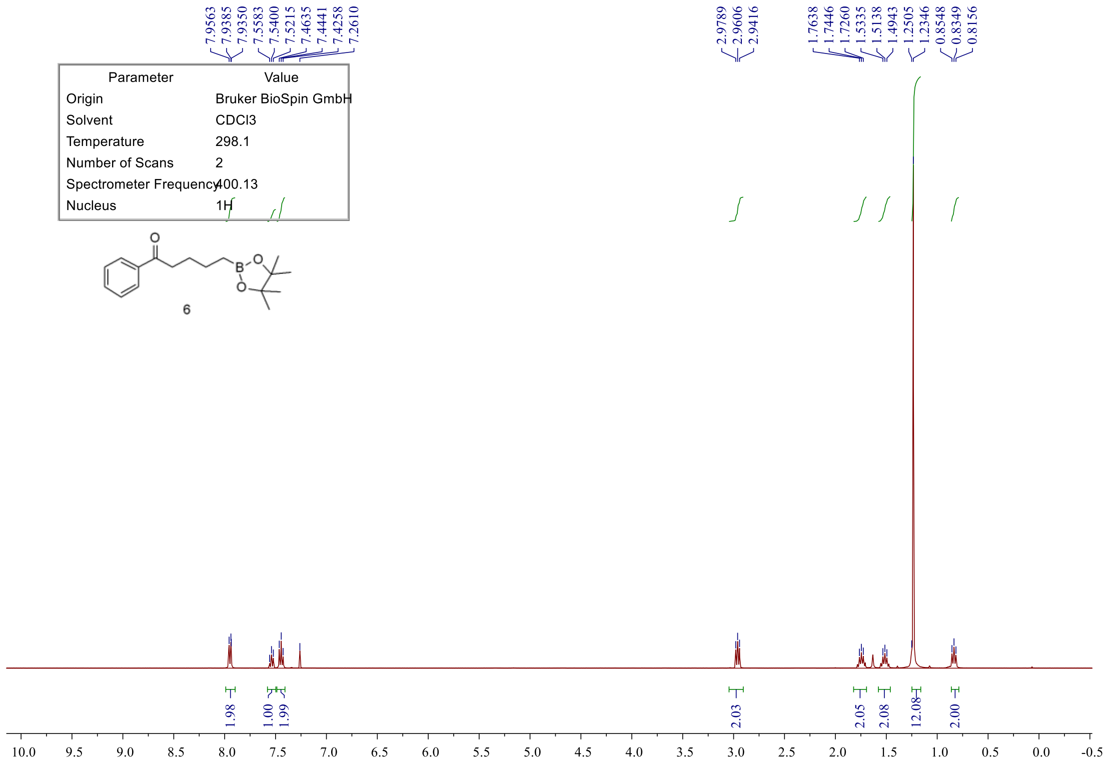
7.9563
7.9385
7.9350
7.5583
7.5400
7.5215
7.4635
7.4441
7.4258
7.2610
2.9789
2.9606
2.9416
1.7638
1.7446
1.7260
1.5335
1.5138
1.4943
1.2505
1.2346
0.8548
0.8349
0.8156
Parameter Value
Origin Bruker BioSpin GmbH
Solvent CDCl3
Temperature 298.1
Number of Scans 2
Spectrometer Frequency 400.13
Nucleus 1H
O
B
O
O
6
1.98
1.00
1.99
2.03
2.05
2.08
12.08
2.00
10.0 9.5 9.0 8.5 8.0 7.5 7.0 6.5 6.0 5.5 5.0 4.5 4.0 3.5 3.0 2.5 2.0 1.5 1.0 0.5 0.0 -0.5

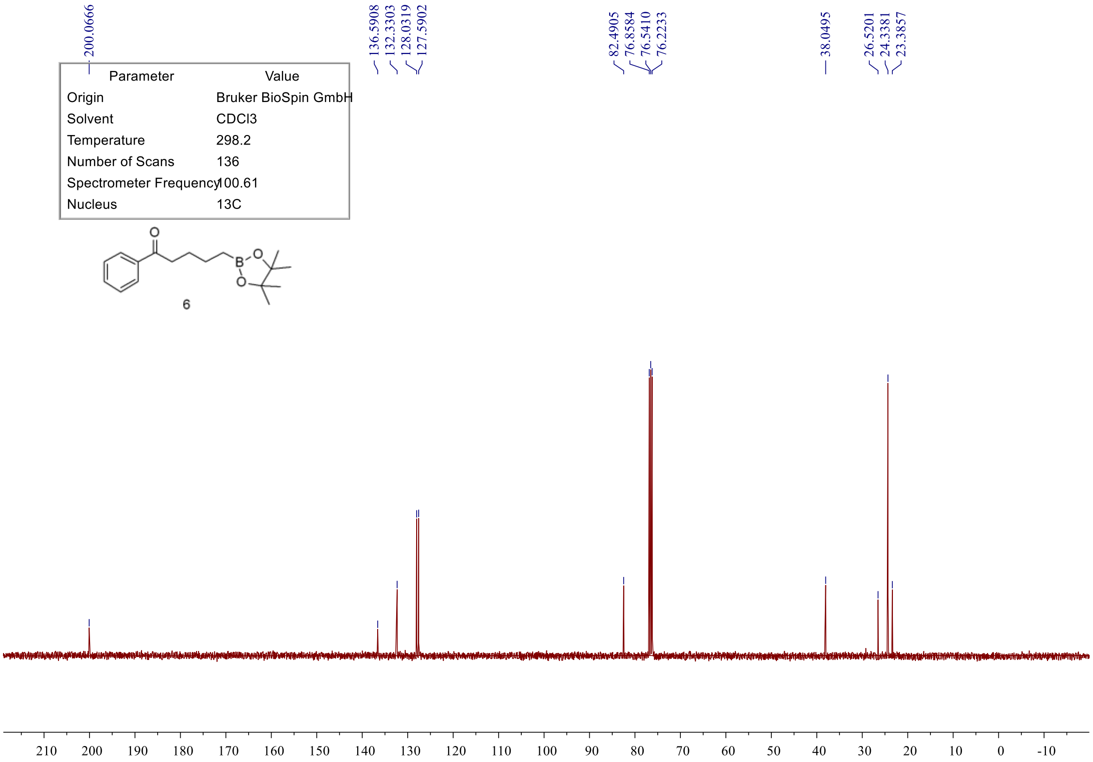

200.0666
136.5908
132.3303
128.0319
127.5902
82.4905
76.8584
76.5410
76.2233
38.0495
26.5201
24.3381
23.3857
Parameter Value
Origin Bruker BioSpin GmbH
Solvent CDCl3
Temperature 298.2
Number of Scans 136
Spectrometer Frequency 100.61
Nucleus 13C
O
B
O
O
6
210 200 190 180 170 160 150 140 130 120 110 100 90 80 70 60 50 40 30 20 10 0 -10

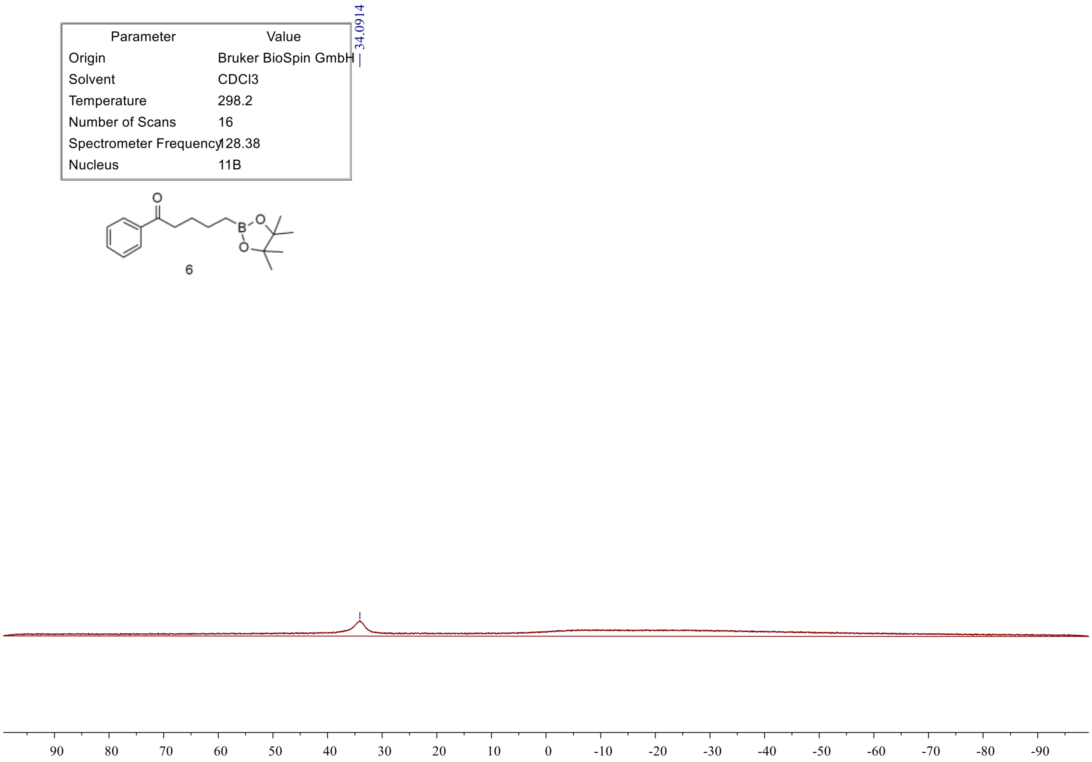

34.0914
Parameter Value
Origin Bruker BioSpin GmbH
Solvent CDCl3
Temperature 298.2
Number of Scans 16
Spectrometer Frequency 128.38
Nucleus 11B
O
B
O
O
6
90 80 70 60 50 40 30 20 10 0 -10 -20 -30 -40 -50 -60 -70 -80 -90

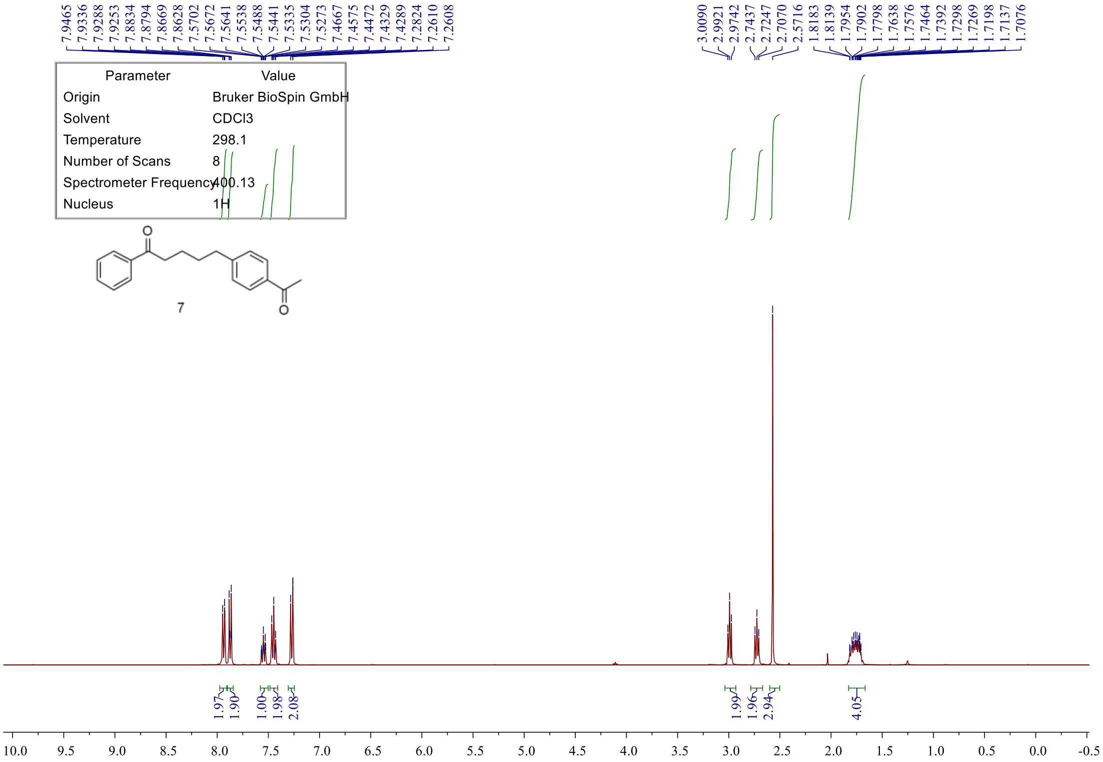
7.9465
7.9336
7.9288
7.9253
7.8834
7.8794
7.8669
7.8628
7.5702
7.5672
7.5641
7.5538
7.5488
7.5441
7.5335
7.5304
7.5273
7.4667
7.4575
7.4472
7.4329
7.4289
7.2824
7.2610
7.2608
3.0090
2.9921
2.9742
2.7437
2.7247
2.7070
2.5716
1.8183
1.8139
1.7954
1.7902
1.7798
1.7638
1.7576
1.7464
1.7392
1.7298
1.7269
1.7198
1.7137
1.7076
Parameter
Value
Origin
Bruker BioSpin GmbH
Solvent
CDCl3
Temperature
298.1
Number of Scans
8
Spectrometer Frequency
400.13
Nucleus
1H
7
1.97
1.90
1.00
1.98
2.08
1.99
1.96
2.94
4.05
10.0 9.5 9.0 8.5 8.0 7.5 7.0 6.5 6.0 5.5 5.0 4.5 4.0 3.5 3.0 2.5 2.0 1.5 1.0 0.5 0.0 -0.5

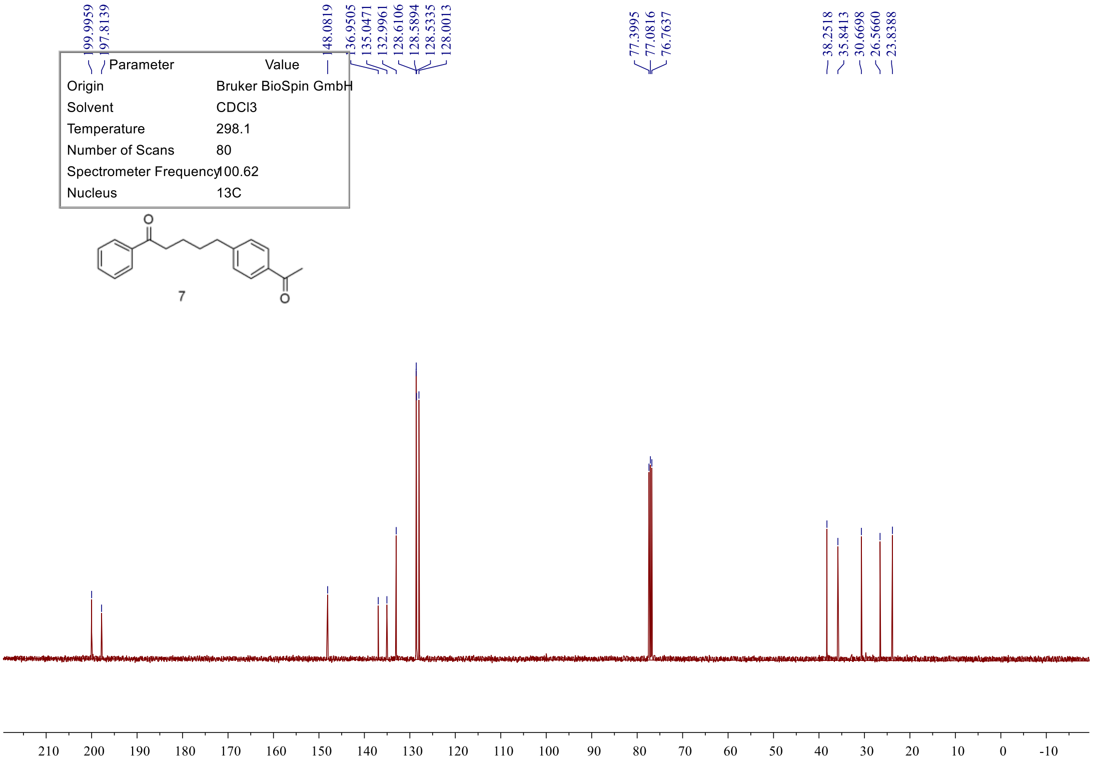
199.9959
197.8139
148.0819
136.9505
135.0471
132.9961
128.6106
128.5894
128.5335
128.0013
77.3995
77.0816
76.7637
38.2518
35.8413
30.6698
26.5660
23.8388
Parameter
Value
Origin
Bruker BioSpin GmbH
Solvent
CDCl3
Temperature
298.1
Number of Scans
80
Spectrometer Frequency
100.62
Nucleus
13C
7
210 200 190 180 170 160 150 140 130 120 110 100 90 80 70 60 50 40 30 20 10 0 -10

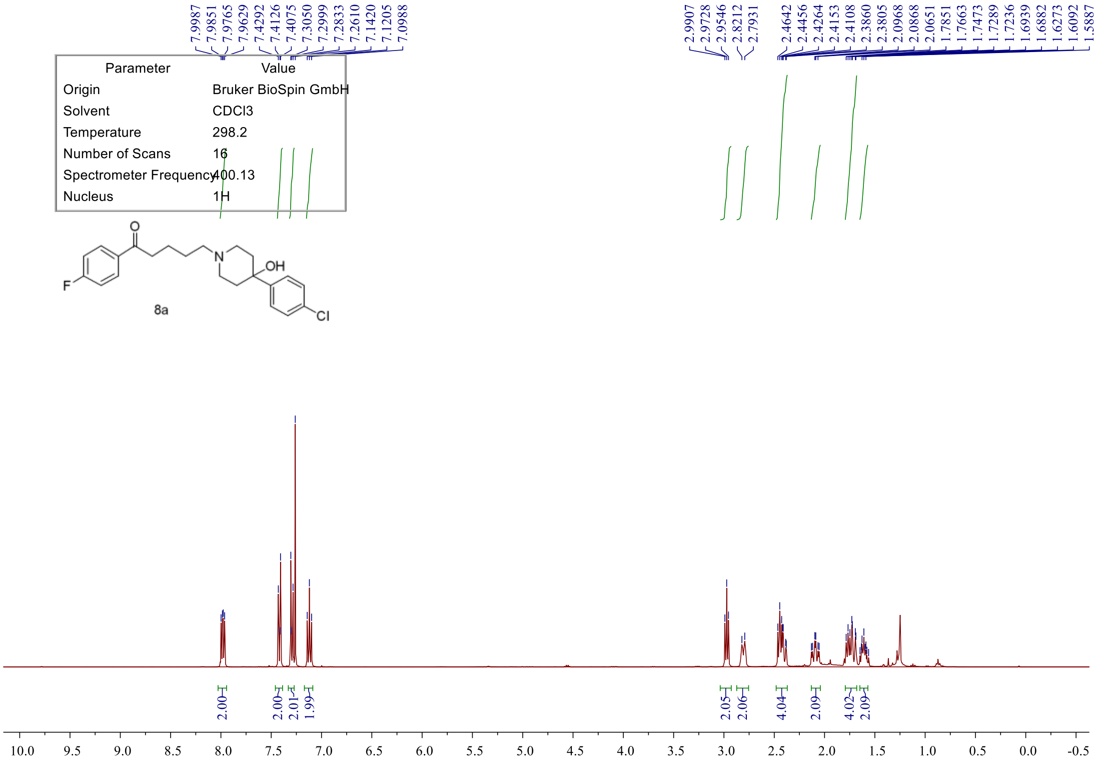
7.9987
7.9851
7.9765
7.9629
7.4292
7.4126
7.4075
7.3050
7.2999
7.2833
7.2610
7.1420
7.1205
7.0988
2.9907
2.9728
2.9546
2.8212
2.7931
2.4642
2.4456
2.4264
2.4153
2.4108
2.3860
2.3805
2.0968
2.0868
2.0651
1.7851
1.7663
1.7473
1.7289
1.7236
1.6939
1.6882
1.6273
1.6092
1.5887
Parameter Value
Origin Bruker BioSpin GmbH
Solvent CDCl3
Temperature 298.2
Number of Scans 16
Spectrometer Frequency 400.13
Nucleus 1H
8a
2.00
2.00
2.01
1.99
2.05
2.06
4.04
2.09
4.02
2.09
10.0 9.5 9.0 8.5 8.0 7.5 7.0 6.5 6.0 5.5 5.0 4.5 4.0 3.5 3.0 2.5 2.0 1.5 1.0 0.5 0.0 -0.5

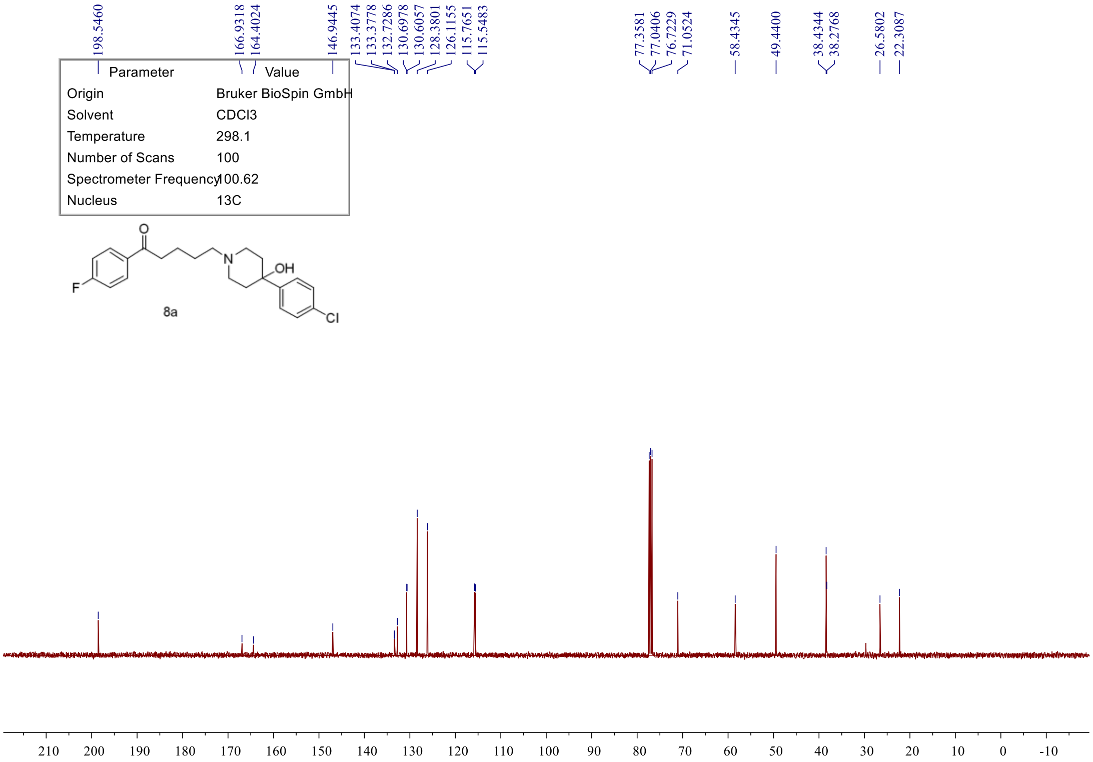
198.5460
166.9318
164.4024
146.9445
133.4074
133.3778
132.7286
130.6978
130.6057
128.3801
126.1155
115.7651
115.5483
77.3581
77.0406
76.7229
71.0524
58.4345
49.4400
38.4344
38.2768
26.5802
22.3087
Parameter Value
Origin Bruker BioSpin GmbH
Solvent CDCl3
Temperature 298.1
Number of Scans 100
Spectrometer Frequency 100.62
Nucleus 13C
8a
210 200 190 180 170 160 150 140 130 120 110 100 90 80 70 60 50 40 30 20 10 0 -10

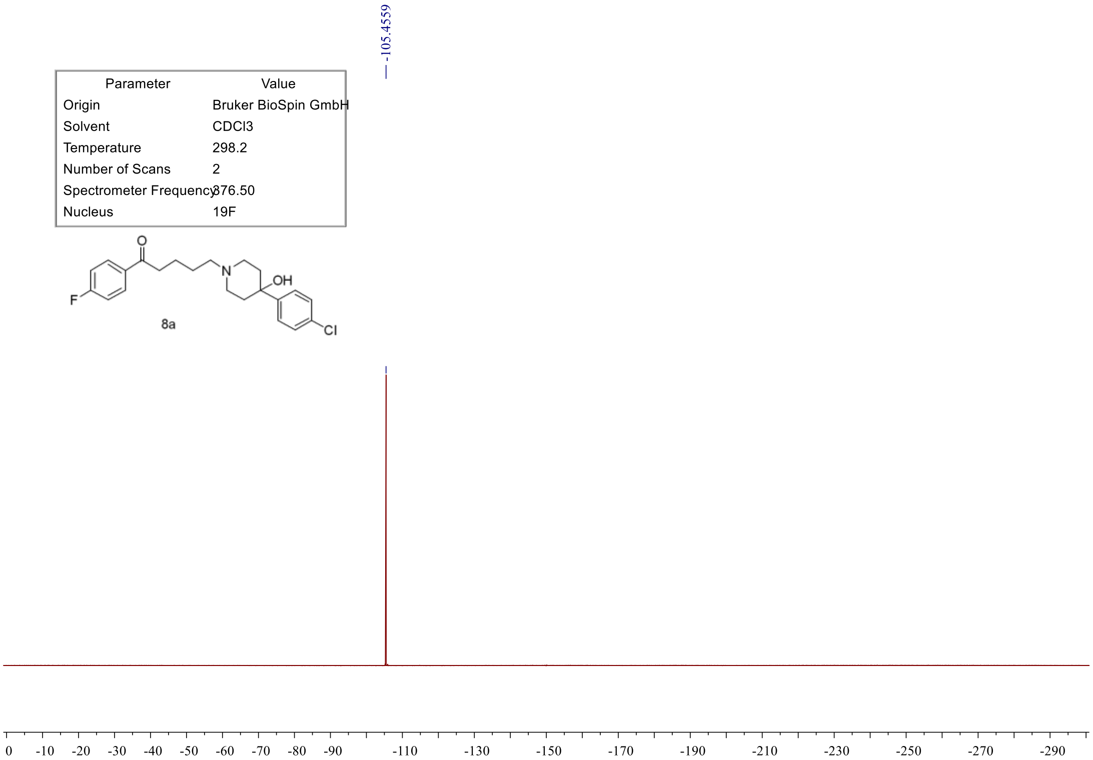

Parameter	Value
Origin	Bruker BioSpin GmbH
Solvent	CDCl3
Temperature	298.2
Number of Scans	2
Spectrometer Frequency	376.50
Nucleus	19F

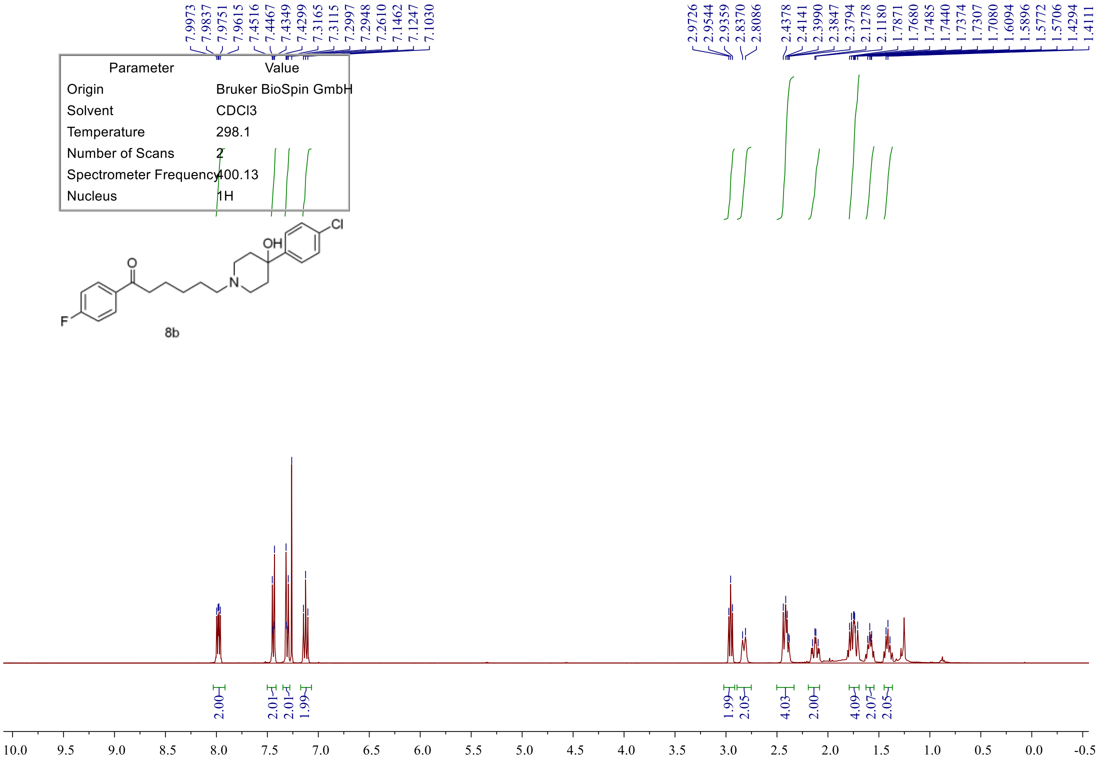

Parameter	Value
Origin	Bruker BioSpin GmbH
Solvent	CDCl3
Temperature	298.1
Number of Scans	2
Spectrometer Frequency	400.13
Nucleus	1H

Parameter	Value
Origin	Bruker BioSpin GmbH
Solvent	CDCl3
Temperature	298.2
Number of Scans	50
Spectrometer Frequency	100.61
Nucleus	13C

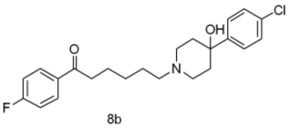

8b

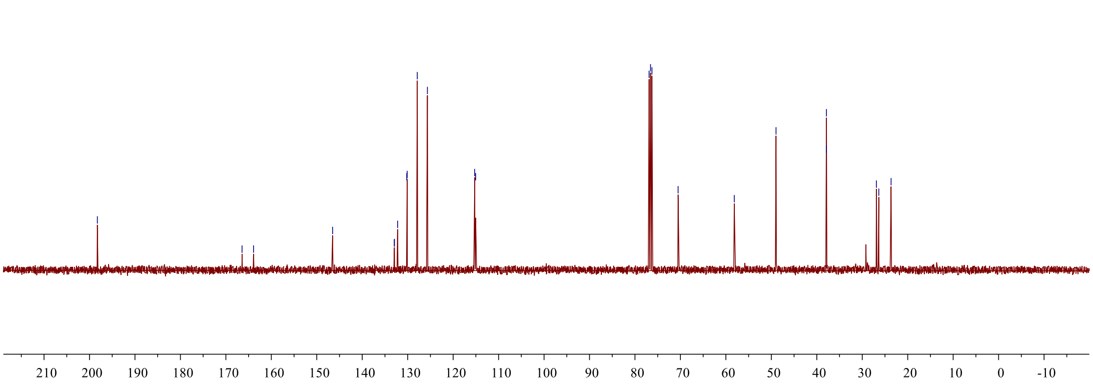

Parameter	Value
Origin	Bruker BioSpin GmbH
Solvent	CDCl3
Temperature	298.2
Number of Scans	2
Spectrometer Frequency	376.50
Nucleus	19F

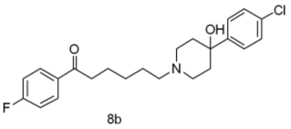

8b

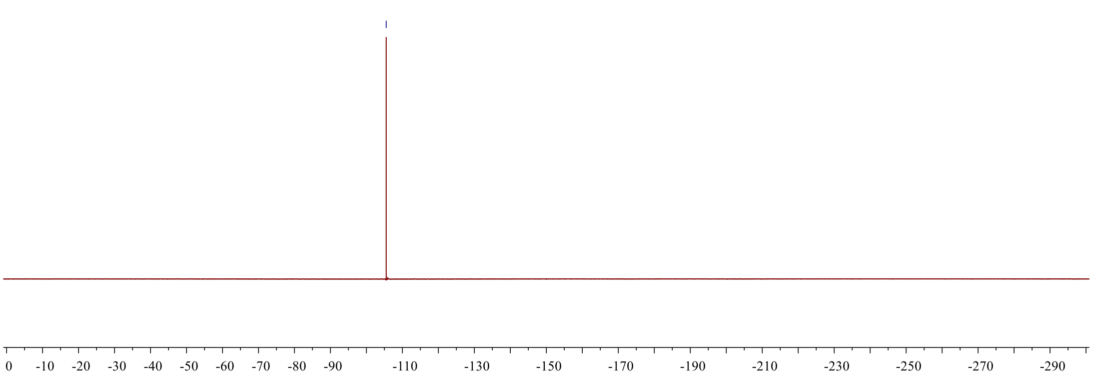

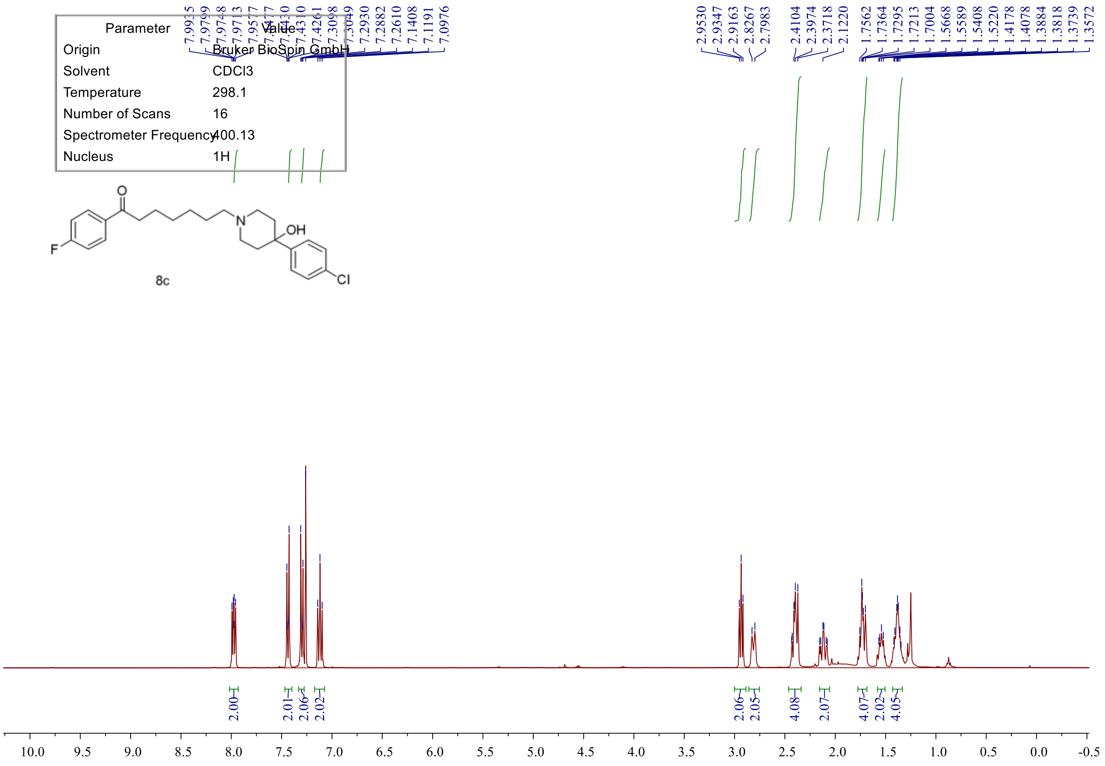
Parameter Value
Origin Bruker BioSpin GmbH
Solvent CDCl3
Temperature 298.1
Number of Scans 16
Spectrometer Frequency 400.13
Nucleus 1H
8c

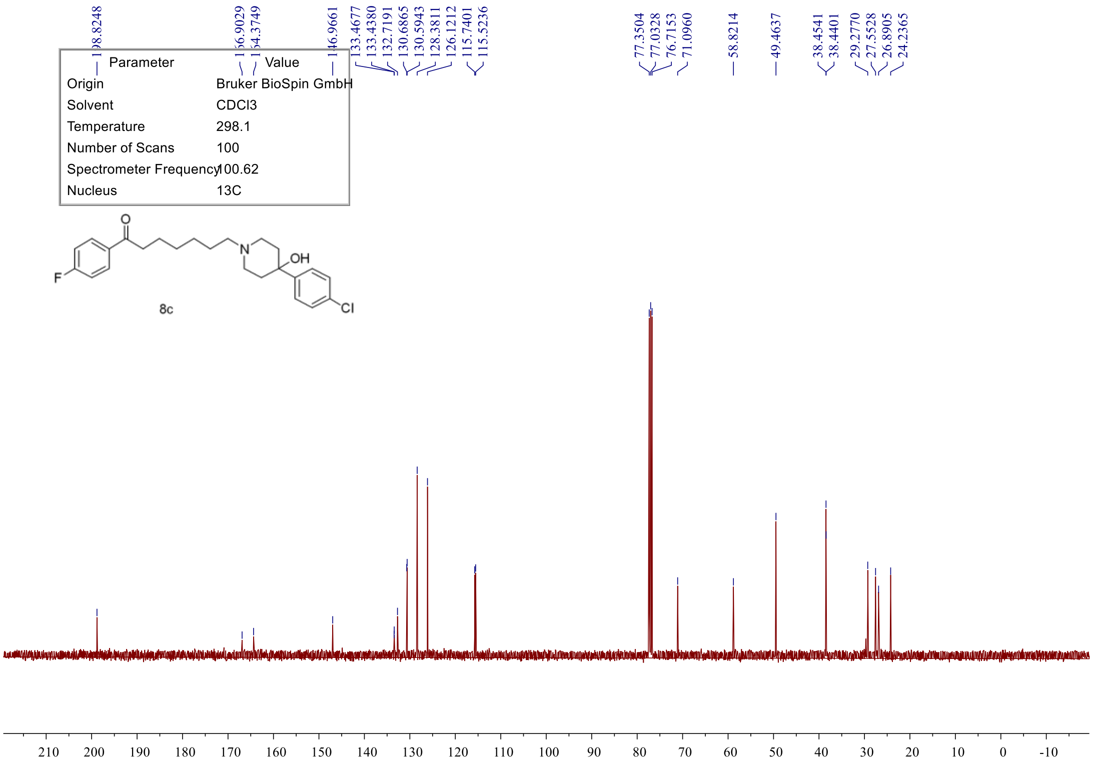
Parameter Value
Origin Bruker BioSpin GmbH
Solvent CDCl3
Temperature 298.1
Number of Scans 100
Spectrometer Frequency 100.62
Nucleus 13C
8c

Parameter	Value
Origin	Bruker BioSpin GmbH
Solvent	CDCl3
Temperature	298.2
Number of Scans	8
Spectrometer Frequency	376.50
Nucleus	19F

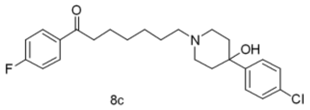

8c

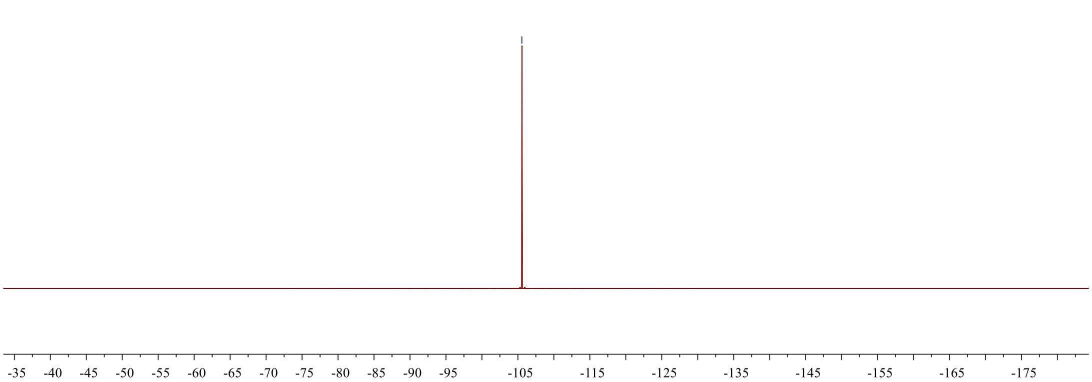

Parameter	Value
Origin	Bruker BioSpin GmbH
Solvent	CDCl3
Temperature	298.2
Number of Scans	8
Spectrometer Frequency	400.13
Nucleus	1H

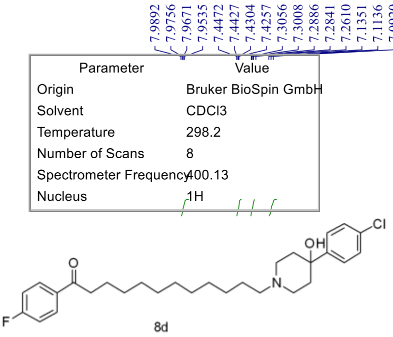

8d

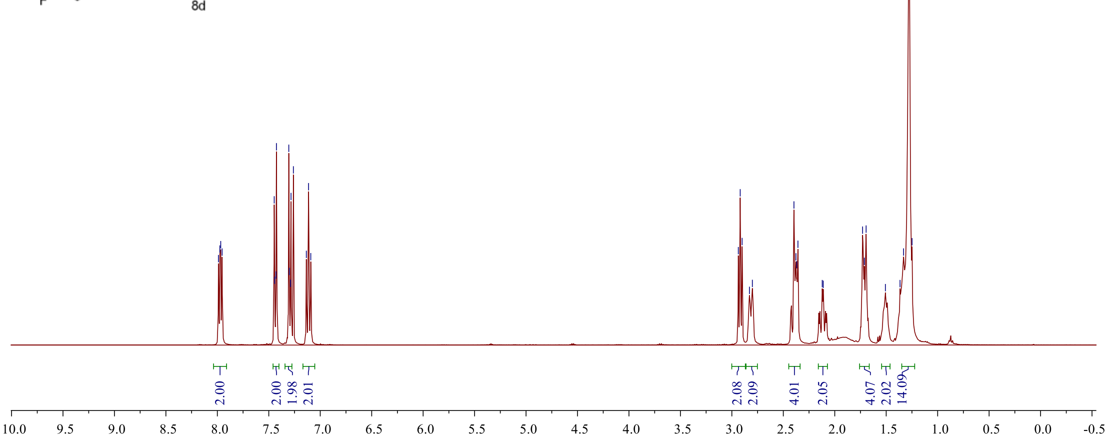

Parameter	Value
Origin	Bruker BioSpin GmbH
Solvent	CDCl3
Temperature	298.1
Number of Scans	23
Spectrometer Frequency	100.62
Nucleus	13C

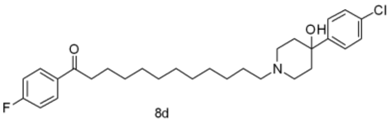

8d

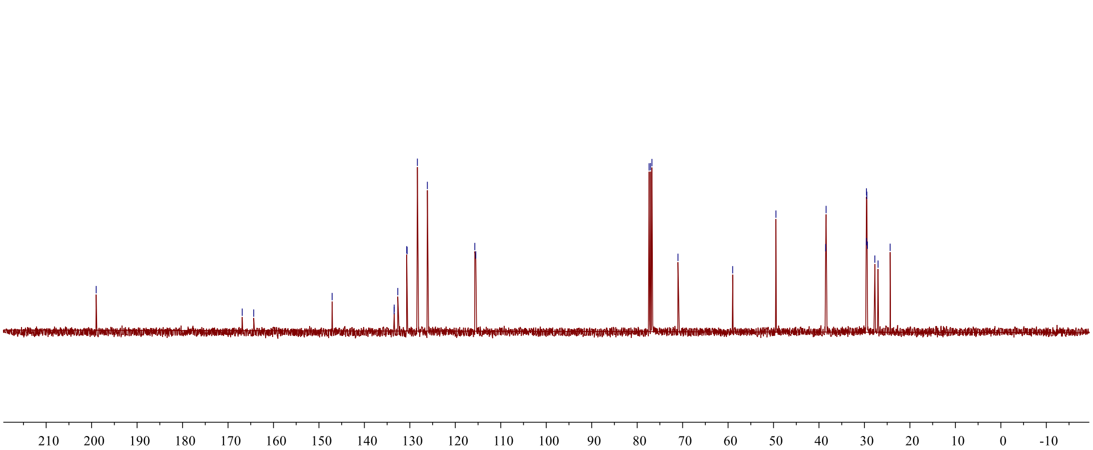

Parameter	Value
Origin	Bruker BioSpin GmbH
Solvent	CDCl3
Temperature	298.2
Number of Scans	8
Spectrometer Frequency	376.50
Nucleus	19F

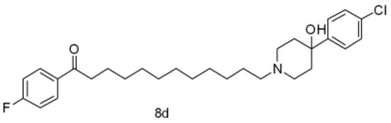

8d

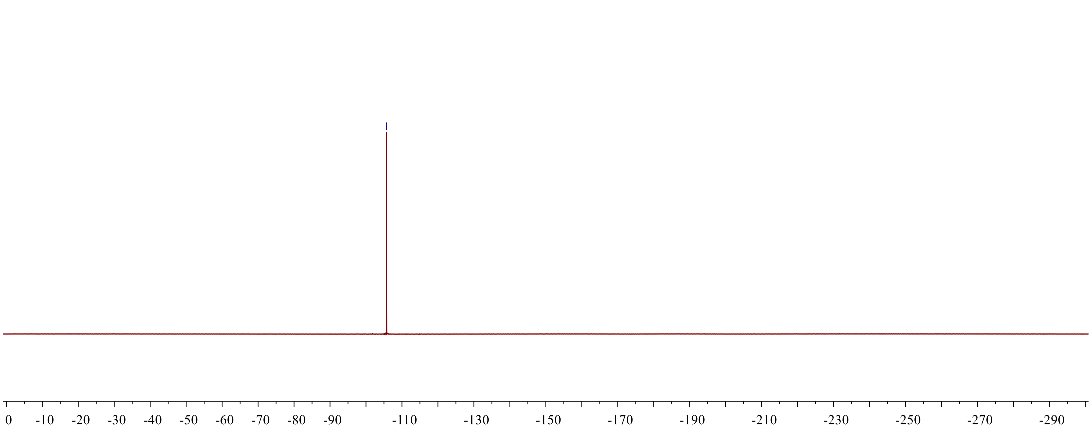

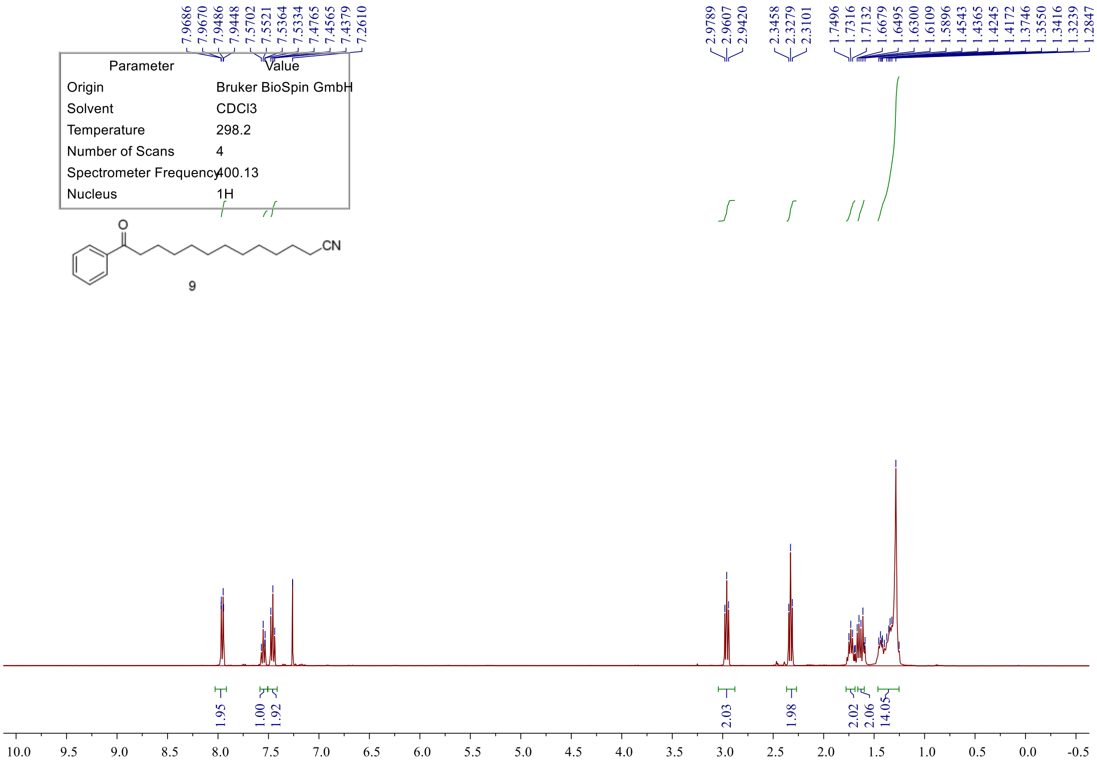
7.9686
7.9670
7.9486
7.9448
7.5702
7.5521
7.5364
7.5334
7.4765
7.4565
7.4379
7.2610
2.9789
2.9607
2.9420
2.3458
2.3279
2.3101
1.7496
1.7316
1.7132
1.6679
1.6495
1.6300
1.6109
1.5896
1.4543
1.4365
1.4245
1.4172
1.3746
1.3550
1.3416
1.3239
1.2847
Parameter Value
Origin Bruker BioSpin GmbH
Solvent CDCl3
Temperature 298.2
Number of Scans 4
Spectrometer Frequency 400.13
Nucleus 1H
O
CN
9
1.95
1.00
1.92
2.03
1.98
2.02
2.06
14.05
10.0 9.5 9.0 8.5 8.0 7.5 7.0 6.5 6.0 5.5 5.0 4.5 4.0 3.5 3.0 2.5 2.0 1.5 1.0 0.5 0.0 -0.5

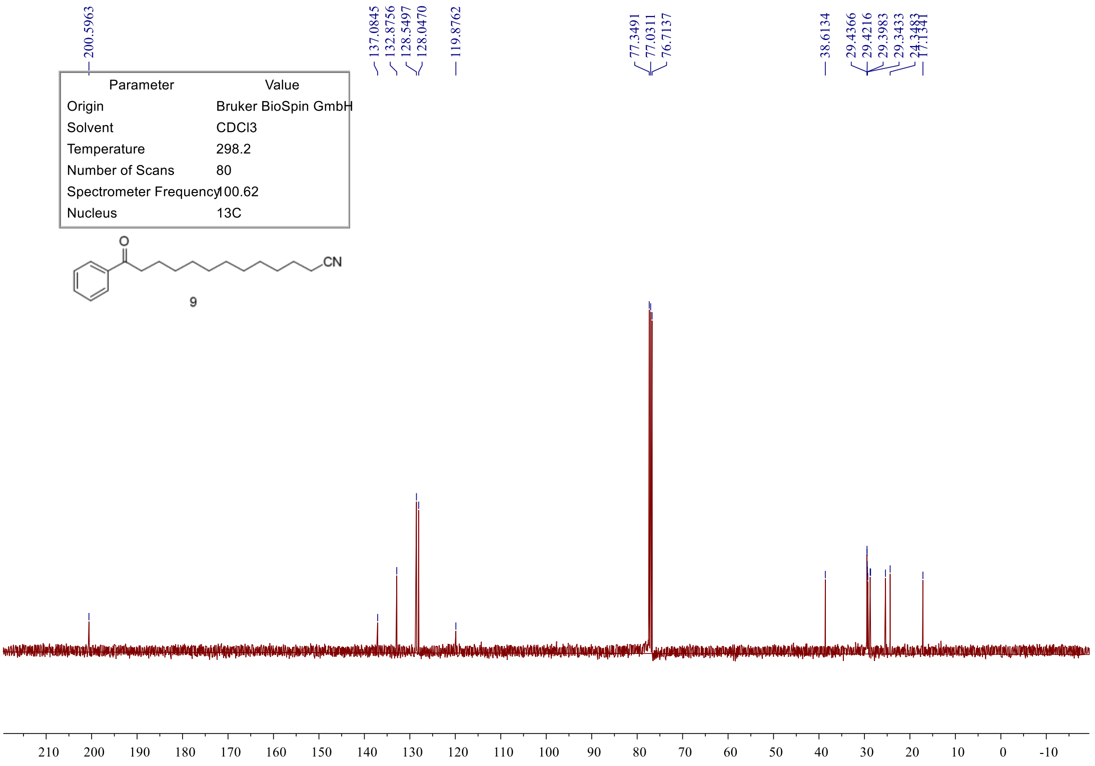
200.5963
137.0845
132.8756
128.5497
128.0470
119.8762
77.3491
77.0311
76.7137
38.6134
29.4366
29.4216
29.3983
29.3433
24.3483
17.1341
Parameter Value
Origin Bruker BioSpin GmbH
Solvent CDCl3
Temperature 298.2
Number of Scans 80
Spectrometer Frequency 100.62
Nucleus 13C
O
CN
9
210 200 190 180 170 160 150 140 130 120 110 100 90 80 70 60 50 40 30 20 10 0 -10

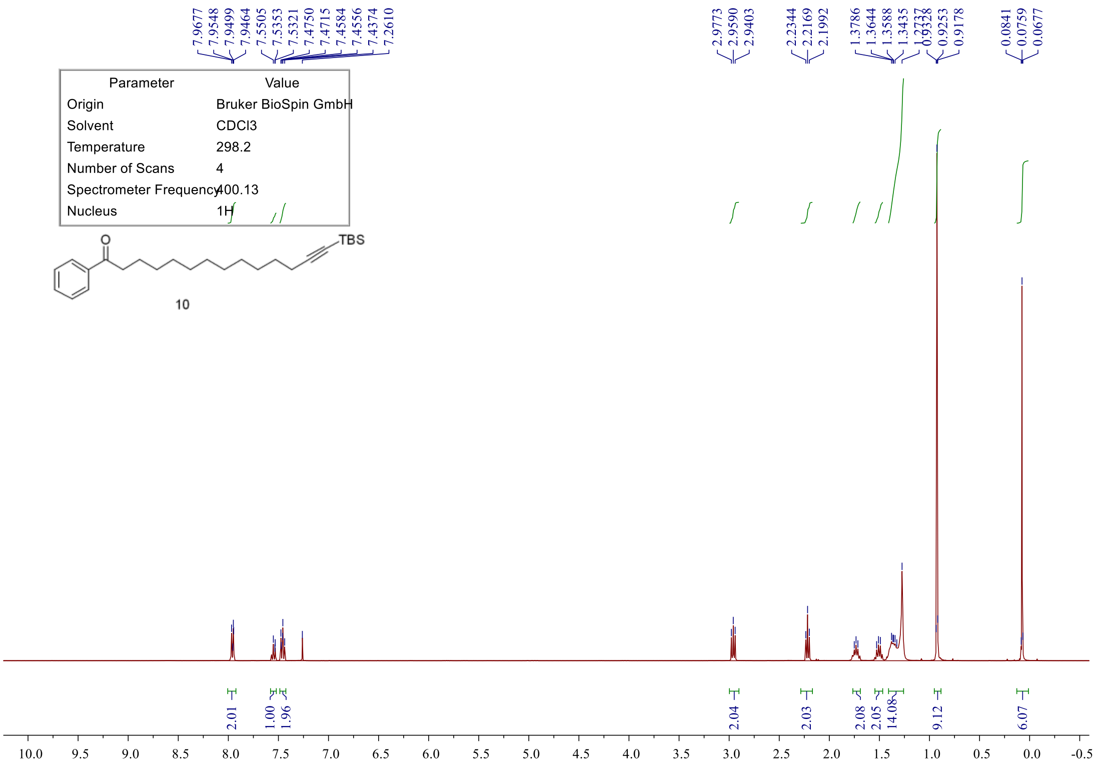
7.9677
7.9548
7.9499
7.9464
7.5505
7.5353
7.5321
7.4750
7.4715
7.4584
7.4556
7.4374
7.2610
2.9773
2.9590
2.9403
2.2344
2.2169
2.1992
1.3786
1.3644
1.3588
1.3435
0.9328
0.9253
0.9178
0.0841
0.0759
0.0677
Parameter Value
Origin Bruker BioSpin GmbH
Solvent CDCl3
Temperature 298.2
Number of Scans 4
Spectrometer Frequency 400.13
Nucleus 1H
TBS
10
2.01
1.00
1.96
2.04
2.03
2.08
2.05
14.08
9.12
6.07
10.0 9.5 9.0 8.5 8.0 7.5 7.0 6.5 6.0 5.5 5.0 4.5 4.0 3.5 3.0 2.5 2.0 1.5 1.0 0.5 0.0 -0.5

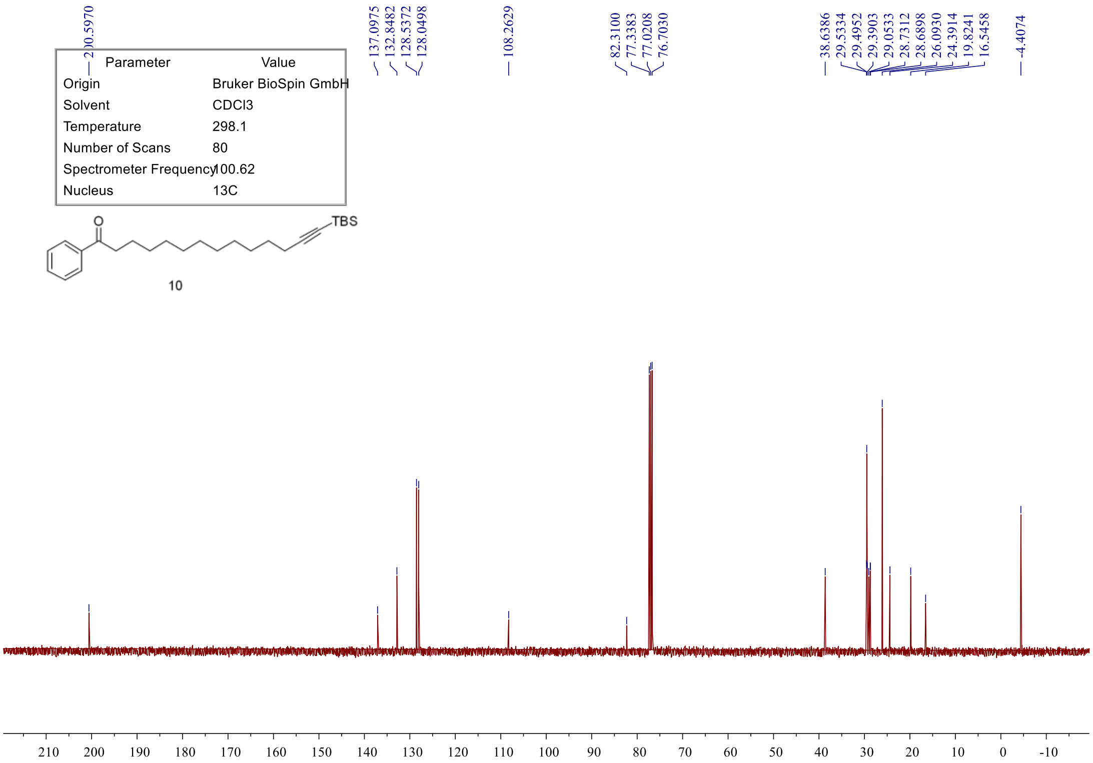
200.5970
137.0975
132.8482
128.5372
128.0498
108.2629
82.3100
77.3383
77.0208
76.7030
38.6386
29.5334
29.4952
29.3903
29.0533
28.7312
28.6898
26.0930
24.3914
19.8241
16.5458
-4.4074
Parameter Value
Origin Bruker BioSpin GmbH
Solvent CDCl3
Temperature 298.1
Number of Scans 80
Spectrometer Frequency 100.62
Nucleus 13C
TBS
10
210 200 190 180 170 160 150 140 130 120 110 100 90 80 70 60 50 40 30 20 10 0 -10

7. Cyclic voltammograms of 1a and 1k

All voltammograms were taken at room temperature using a saturated calomel (SCE) reference electrode, a mesh platinum (Pt) counter electrode, and a glassy carbon working electrode. The conditions of the experiments were the following: an acetonitrile solution of 100 mM tetrabutylammonium hexafluorophosphate (NBu_4PF_6) and 1 mM aryl alcohol, a scan rate of 0.1 V/s, and a positive initial scan direction. The reported potentials were averages over segments, and were taken at half-height of the cathodic peaks ($E_{p/2}$) of **1a**, since all oxidations were nonreversible. To convert the potentials from SCE to Fc/Fc^+ reference, 380 mV were subtracted from the measured values. The positive peaks on the return sweep of most substrates were thought to signify an ECE-type mechanism.

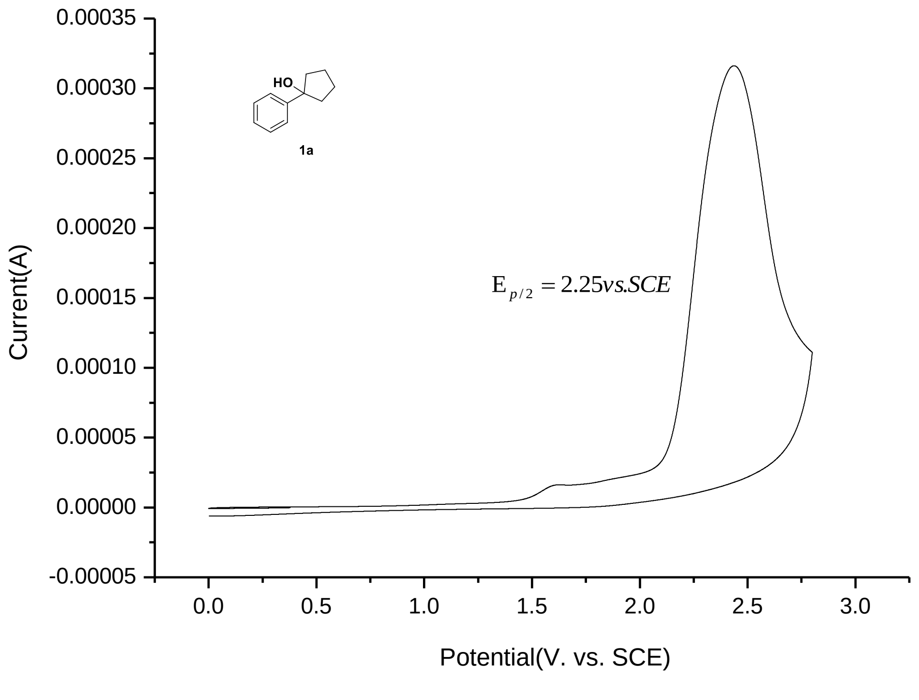

All voltammograms were taken at room temperature using a saturated calomel (SCE) reference electrode, a mesh platinum (Pt) counter electrode, and a glassy carbon working electrode. The conditions of the experiments were the following: an acetonitrile solution of 100 mM tetrabutylammonium hexafluorophosphate (NBu_4PF_6) and 1 mM aryl alcohol, a scan rate of 0.1 V/s, and a positive initial scan direction. The reported potentials were averages over segments, and were taken at half-height of the cathodic peaks ($E_{p/2}$) of **1k**, since all oxidations were nonreversible. To convert the potentials from SCE to Fc/Fc^+ reference, 380 mV were subtracted from the measured values. The positive peaks on the return sweep of most substrates were thought to signify an ECE-type mechanism.

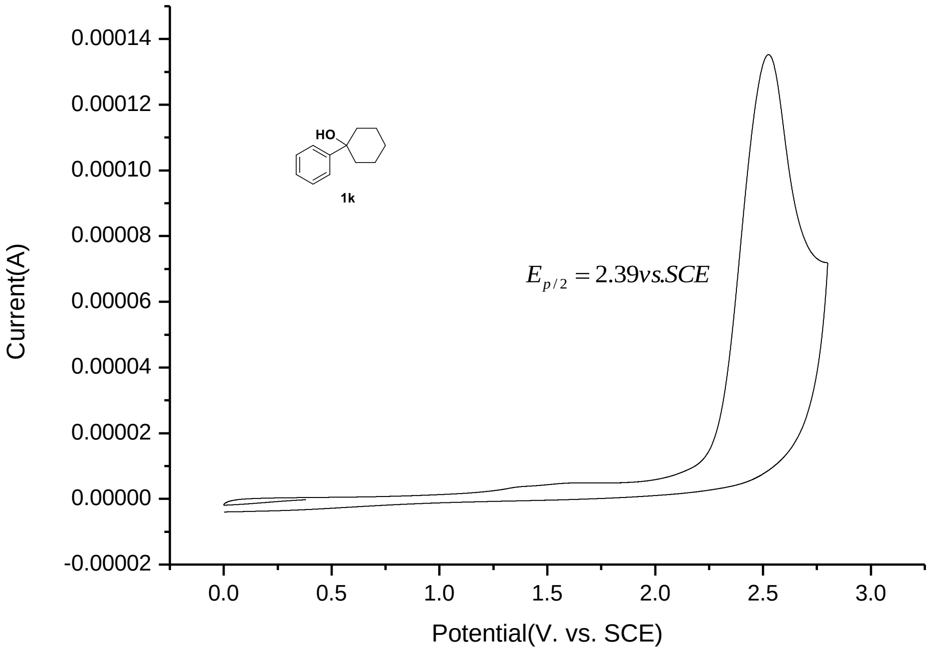

8. Emission quenching experiments (Stern–Volmer studies)

Emission intensities were recorded using a FLS980 (Edinburgh Instrument, UK) luminescence spectrophotometer. All $[Ir(ppy)_2(dtbbpy)]PF_6$ solutions were excited at 380 nm and the emission intensity was collected at 588 nm. In a typical experiment, to a $2{\cdot}10^{-5}$ M solution of $[Ir(ppy)_2(dtbbpy)]PF_6$ in CH_3CN was added the appropriate amount of NBS in a screw-top quartz cuvette. After degassing the sample with a stream of N_2 for 10 minutes, the emission of the sample was collected.

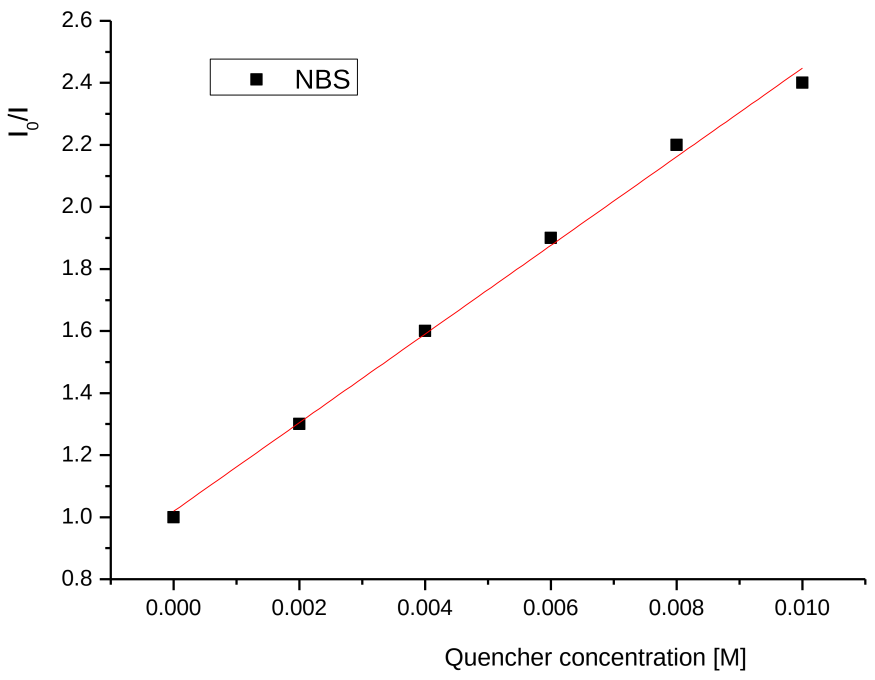

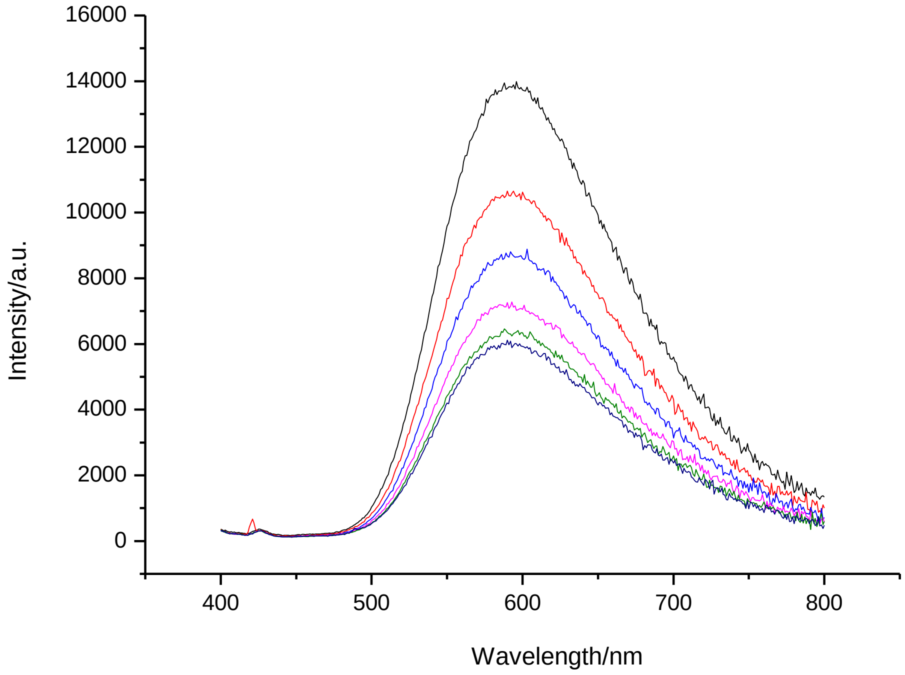
16000
14000
12000
10000
8000
6000
4000
2000
0
Intensity/a.u.
400
500
600
700
800
Wavelength/nm
